# Repositioning Lomitapide to block ZDHHC5-dependant palmitoylation on SSTR5 leads to anti-proliferation effect in preclinical pancreatic cancer models

**DOI:** 10.1038/s41420-023-01359-4

**Published:** 2023-02-11

**Authors:** Yumeng Wang, Shujie Zhang, Huiqin He, Hongyi Luo, Yannan Xia, Yuanyuan Jiang, Jingwei Jiang, Li Sun

**Affiliations:** 1 Jiangsu key lab of Drug Screening, China Pharmaceutical University, Nanjing, 210009 China; 2 Shuangyun BioMed Sci & Tech (Suzhou) Co., Ltd, Suzhou, 215028 China

**Keywords:** Targeted therapies, Pancreatic cancer

## Abstract

Palmitoylation of proteins plays important roles in various physiological processes, such as cell proliferation, inflammation, cell differentiation etc. However, inhibition of protein palmitoylation has led to few new drugs to date. ZDHHC5 serves as a key enzyme to catalyze palmitoylation on SSTR5 (a proven anti-proliferation receptor in pancreatic cells). Herein, we compare single-cell transcriptome data between pancreatic cancer tissues and normal pancreas tissues and identify that ZDHHC5 is a potential target to inhibit proliferation of pancreatic cancer cells. In addition, we report the repositioning of an orphan drug (Lomitapide) as an inhibitor of ZDHHC5, and we speculate that this inhibitor may be able to block palmitylation on SSTR5. Pharmacological blockade of ZDHHC5 with Lomitapide results in attenuated cancer cell growth and proliferation which collectively contributes to antitumor responses in vitro and in vivo. This is the first study, to our knowledge, to demonstrate the utility of a pharmacological inhibitor of ZDHHC5 in pancreatic cancer, representing a new class of palmitoylation targeted therapy and laying a framework for paradigm-shifting therapies targeting cancer cell palmitoylation.

## Introduction

Palmitoylation is the reversible attachment of the fatty acid palmitate to cysteine thiols via a thioester bond [1–3]. Protein palmitoylation is an important post-modification on expressed proteins. Palmitoylation increases the hydrophobicity of protein domains, thus contributing to protein structure, function and cellular localization [4]. In the case of G-protein coupled receptors (GPCRs), palmitoylation of a cysteine on the GPCR’s tail makes GPCR’s tail anchor to the cell membrane which significantly influences the efficiency of its coupling to G-proteins [5, 6]. Though palmitoylation of many GPCRs has been demonstrated, the enzymes responsible for this modification have been unknown. Only during the last two decade the family of ZDHHC proteins (23 proteins in mammals; characterized by a conserved zinc finger motif containing amino acid sequence DHHC) have been recognized as putative palmitoyltransferases [7–9]. However, so far it has been unclear whether any of the ZDHHC family members recognize GPCRs as substrates.

S-palmitoylation, catalyzed by the ZDHHC domain–containing protein acyl-transferases (PATs), plays important roles in immune responses, neural cell differentiation and anti-proliferation [10–14]. In the last decade, many reports focused on ZDHHC5’s palmitoylation and identified many protein substrates palmitoylated by ZDHHC5, such as STAT3 [15], SSTR5 [16], APT-1 [17], GRIP1 [18], flotillin-2 [19], delta-catenin [20], S1PR1 [21], EZH2 [22] and NOD1/NOD2 [23]. In the above reports, ZDHHC5 influences the localization of these substrates through palmitoylation hence regulates their functions. SSTR5 is activated by somatostatin 28 subsequently inhibiting cell proliferation [24]. As a protein substrate of ZDHHC5, the tail of SSTR5 is identified palmitoylation, but the function of palmitoylated SSTR5 is not known yet [16]. Previous researches report that SSTR5 agonist somatostatin analog AN-238 significantly suppresses the proliferation of pancreatic cancer cells both in vitro and in vivo [25]. Another study showed that various mutations on SSTR5 tail inhibits the anti-proliferation of SSTR5 on pancreatic cancer cells [26, 27]. In addition, clinical research showed that SSTR5 agonist pasireotide treatment patients (neuroendocrine tumor, *n* = 29) achieve 30-month overall surviva (OS) rate was 70% [28]. Taken together, we hypothesized that antagonizing ZDHHC5-mediated palmitoylation on the cytoplasmic tail of SSTR5 may represent a more efficacious approach for the treatment of pancreatic cancer.

In the present study, we performed single-cell transcriptome sequencing on pancreatic cancer patient samples and identified ZDHHC5 as a potential target for anti-proliferation of pancreatic cancer cells by silencing ZDHHC5 in cancer cells which results in dramatic antitumor effects. Furthermore, we reposition an orphan drug (Lomitapide), the first small molecule antagonist of ZDHHC5, and evaluate its use in an oncology setting. Pharmacological blockade of ZDHHC5 with Lomitapide resulted in attenuated cancer cell growth and proliferation which significantly contributed to anti-tumor responses in vitro and in mouse model in vivo.

## Results

### Single-cell sequencing of pancreatic cancer patient samples

**Fig. 1:**
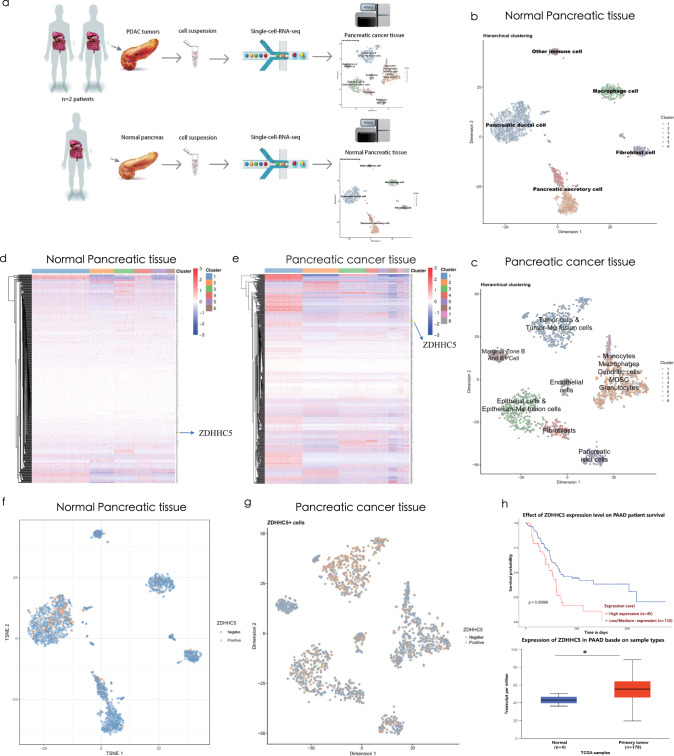
Single-cell sequencing of normal pancreatic tissue and pancreatic cancer tissue, and TCGA database analysis. **a** Flow chart of single cell sequencing. **b** Single cell sequencing of normal pancreas tissue, t-SNE analysis. **c** Single cell sequencing of tumor sample, t-SNE analysis. **d**, **f** ZDHHC5 expressing in normal pancreas tissue (heatmap and t-SNE analysis). **e**, **g** ZDHHC5 expressing in tumor sample (heatmap and t-SNE analysis). **h** TCGA public pancreatic cancer samples, the survival rate of ZDHHC5 (high expression, *n* = 45) patients and ZDHHC5 (low expression, *n* = 132) patients (upper) and the expression level of ZDHHC5 in pancreatic cancer patients (*n* = 178) or normal pancreas samples (*n* = 4).

Based on single-cell sequencing data, tumor sample and normal pancreas tissue are significantly different in terms of cell types as well as their ratio (Fig. 1a). In the tumor micro-environment, there are lots of immune cells as well as their ratio (Fig. 1b, c) such as dendritic cells, monocytes, macrophages etc. in tumor samples compared to normal pancreas tissue. Then, 639 prognostic unfavorable genes collected from THPA database (https://www.proteinatlas.org/) were plotted in terms of heatmap to identify genes expressing most in all cell types of tumor sample but not or less expressing in all cell types of normal pancreas sample. As a result, we identified ZDHHC5 as a candidate gene expressing in most of the cell types in tumor sample compared to normal pancreas sample (Fig. 1d–g). According to 177 TCGA public pancreatic cancer samples, the survival rate of ZDHHC5 (high expression, *n* = 45) patients is much lower than that of ZDHHC5 (low expression, *n* = 132) patients (Fig. 1h). Pseudotime trajectory analysis of tumor cells shows that ZDHHC5 expresses in every branch suggesting that ZDHHC5 plays important roles in the whole differentiation process of tumor cells (Supplementary Fig. 1a–c). In general, ZDHHC5 expression is associated with genes in tumor proliferation pathway (Supplementary Fig. 1d–h). Moreover, we perform correlation analysis between ZDHHC5 and the proliferative genes and find that ZDHHC5 is significantly correlated with Akt, c-Raf, MEK and ERK in tumor cells (Supplementary Fig. 2a). For non-tumor cells, ZDHHC5 is significantly correlated with Akt and c-Raf, but not with MEK and ERK (Supplementary Fig. 2b). Taken together the above results, we hypothesize that ZDHHC5 is a potential gene leading pancreatic cancer to proliferate through PI3K-Akt proliferative pathway (Supplementary Fig. 1i).

### In vitro evaluation of silencing ZDHHC5 anti-tumor effect

**Fig. 2:**
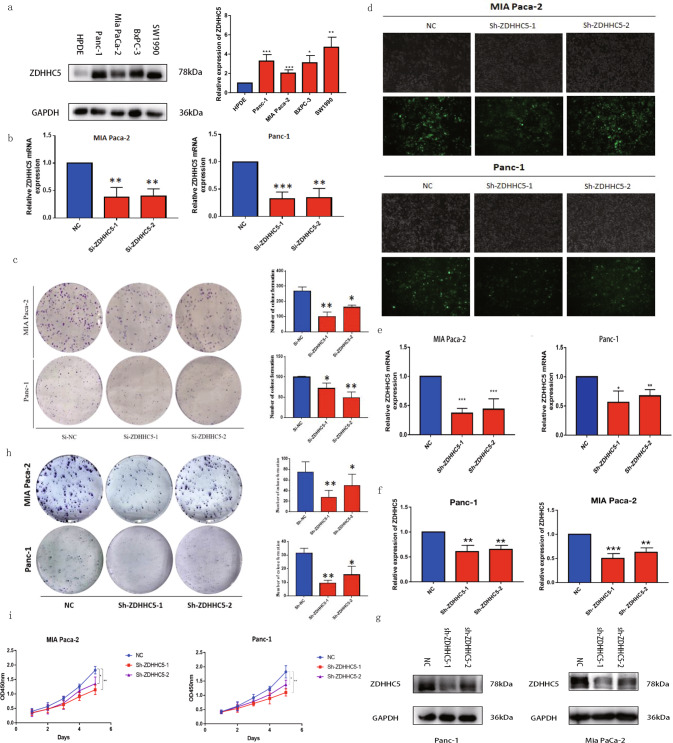
In vitro evaluation of silencing ZDHHC5 anti-tumor effect. **a** ZDHHC5 is upregulated in pancreatic cancer cell lines. Western blot analyses of ZDHHC5 expression in HPDE cell and 4 PDAC cell lines. **b** ZDHHC5 mRNA expression after transfection analyzed by RT-qPCR. **c** After transfection, colony formation assay of cell viability in MIA Paca-2 and Panc-1 cell lines after cultured 15 days. **d** Establishment of stable ZDHHC5 knockdown pancreatic cancer cell lines. MIA Paca-2 and Panc-1 cells were transduced with a lentivirus vector containing negative control ShRNA (NC) or one of two ShRNAs against ZDHHC5 (Sh-ZDHHC5-1 and Sh-ZDHHC5-2) for 48 h. Morphology of MIA Paca-2 and Panc-1 cells was captured. **e** mRNA expression of stable ZDHHC5 knockdown cell lines were analyzed by RT-PCR. **f**, **g** Knockdown efficiency on protein level was detected using Western blot. **h** After construction of stable ZDHHC5 knockdown cell lines, colony formation assay of cell viability in MIA Paca-2 and Panc-1 cell lines after cultured 15 days. **i** Transduced MIA Paca-2 and Panc-1 cells were cultured for 5 days post transduction. The absorbance at 450 nm was measured with a microplate analyzer every 24 h to assess cell growth. Data are expressed as mean ± SEM (*n* = 3) *p < 0.05, **p < 0.01, ***p < 0.001 in **a**, **b**, **c**, **e**, **f**, **h** and **i**.

We first check the expression level of ZDHHC5 in different cell lines and find that the expression level of ZDHHC5 is higher in cancer cells than that in HPDE cell (Fig. 2a). Then, we use ZDHHC5-siRNA to transfect Panc-1 and Mia PaCa-2 cell lines and find that ZDHHC5-knockdown significantly decrease cell proliferation in both cells (Fig. 2b, c). To further confirm such anti-proliferation effects, we constructed stable ZDHHC5-knockdown cell lines (Fig. 2d), and mRNA expression of ZDHHC5 was analyzed by RT-qPCR (Fig. 2e), knockdown efficiency on protein level was detected using Western blot (Fig. 2f, g). As a result, stable ZDHHC5-knockdown cell lines also show decreased proliferation ability (Fig. 2h, i).

### Evaluation of silencing ZDHHC5 in vivo

**Fig. 3:**
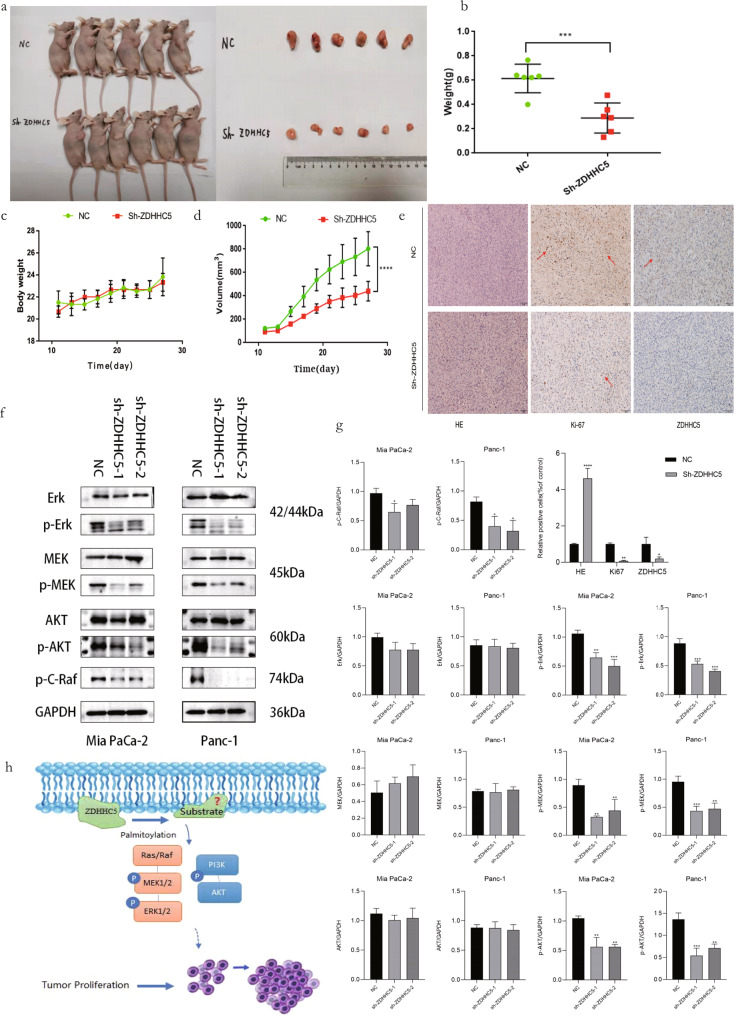
In vivo evaluation of antitumor effects of silenced ZDHHC5, and probable mechanism of PI3K-Akt pathway. **a** Representative images of the subcutaneous xenografts of different treatment groups. **b** Tumor weight of the tumor xenograft. **c** Body weight of the mice, ZDHHC5 depletion had no effect on the body weight of mice. **d** Tumor volume of the tumor xenograft. **e** IHC staining of xenografts of different treatment groups. The expression of ZDHHC5 and Ki67 in mouse pancreatic cancer tissue by immunohistochemistry. Scale bar, 50 μm. **f** Effects of ZDHHC5 knockdown on PI3K/AKT, MAPK/ERK signaling pathway. The protein expression levels of p-AKT, AKT, p-C-Raf, ERK, p-ERK, MEK, p-MEK and GAPDH were detected in MIA Paca-2 and Panc-1 cells by western blots. **g** The statistical graph of **e**, **f**. **h** We hypothesize that ZDHHC5 inhibit the proliferation of pancreatic cancer through the PI3K-Akt pathway. Data are expressed as mean ± SEM (*n* = 6) *p < 0.05, **p < 0.01, ***p < 0.001 in **b**, **c**, **d** and **g**.

We choose stable ZDHHC5-knockdown cell line (Mia PaCa-2) for xenograft experiment in nude mice. The results indicate that ZDHHC5-knockdown (Mia PaCa-2) tumor weight/volume is much smaller than those of Mia PaCa-2 (Fig. 3a–d). And we also see that expression of Ki67 of ZDHHC5-knockdown group is relatively low compared to control group (Fig. 3e). Here, we propose that ZDHHC5 is responsible for palmitoylating a membrane protein which subsequently inhibit protein phosphorylation of its downstream proteins like Akt, c-Raf, MEK and ERK (Fig. 3f–h). Based on published literatures and our above results, we hypothesize that SSTR5 palmitoylated by ZDHHC5 would downregulate its inhibitory effect leading pancreatic cancer cell’s proliferation.

### In silico modeling of ZDHHC5 substrate binding domain

**Fig. 4:**
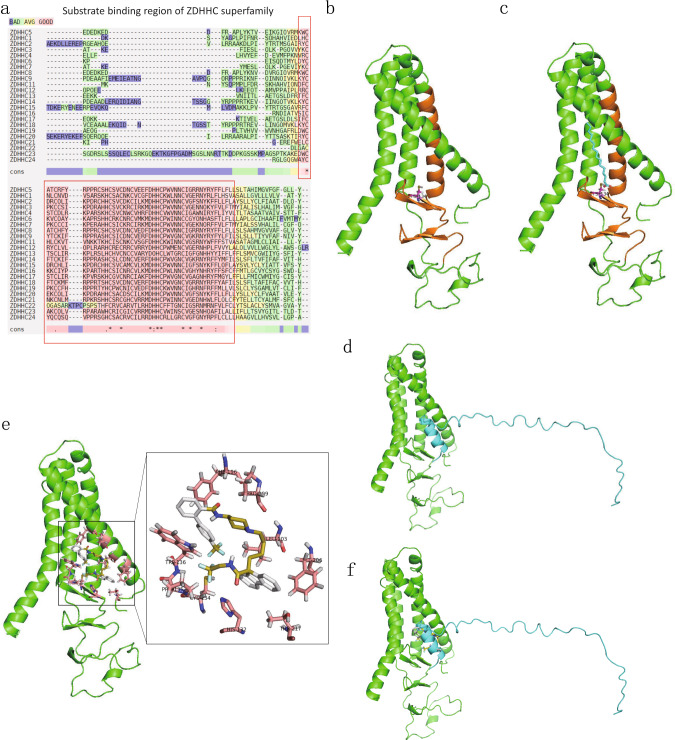
In silico modeling of ZDHHC5 substrate binding domain, molecular docking and virtual drug screening. **a** Sequence alignment of 23 ZDHHC proteins and find that ZDHHC5’s substrate binding domain (DHHC motif) is relatively conserved. **b** The 3D structure model of ZDHHC5 (1–219 aa) and predict that Cys134 is the ZDHHC5 palmitoylation site. **c** The 3D structure model of ZDHHC5 (1–219 aa)’s Cys134 binds palmitic acid. **d** The 3D structure model of SSTR5 (307–362 aa) binds to ZDHHC5 (1–219 aa) and predict that Cys134 is the palmitoylation site. **e** The predicted binding pocket of Lomitapide on ZDHHC5’s substrate binding domain is consisted of His132, CYS134, PRO135, TRP136, PHE196, PRO199, LEU203, PHE206 and THR217. **f** We hypothesize that Lomitapide competitively inhibits the 3D structural model of the substrate binding domain of SSTR5/palmitic acid binding to ZDHHC5.

At first, we align 23 ZDHHC proteins and find that ZDHHC5’s substrate binding domain (DHHC motif) is relatively conserved (Fig. 4a). Subsequently, we model the 3D structure of ZDHHC5 (1–219 aa) and predict that Cys134 is the ZDHHC5 palmitoylation site (Fig. 4b) binding palmitic acid (Fig. 4c). Furthermore, we model the 3D structure of the cytoplasmic tail of SSTR5 (307–362 aa) and perform protein-protein docking with ZDHHC5 (1–219 aa). As a result, SSTR5 (307–362 aa) binds to ZDHHC5 (1–219 aa) around Cys134 predictive palmitoylation site (Fig. 4d). Based on the above computational results, we hypothesize that: a) ZDHHC5 mediated palmitoylation of SSTR5 cytoplasmic tail is around the Cys134 of ZDHHC5; b) Repositioning a small molecule drug binding on the pocket around Cys134 of ZDHHC5 is able to block its palmitoylation reaction.

### Discover DHHC5 antagonist by virtual screening of FDA-approved drugs

**Fig. 5:**
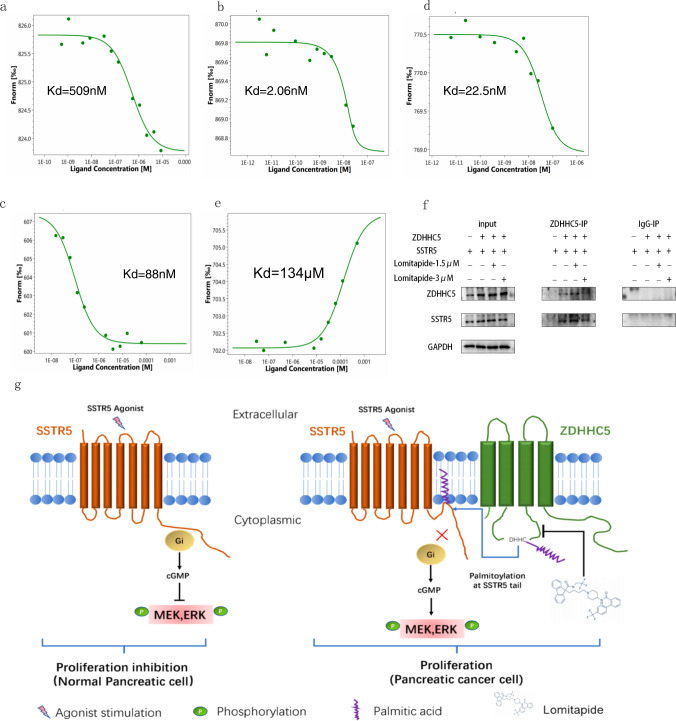
Mechanism of Lomitapide’s inhibition of palmitoylation mediated by ZDHHC5. **a** Binding of Lomitapide and ZDHHC5 substrate domain. **b** Binding of SSTR5 (307–362 aa) and ZDHHC5 substrate domain. **c** Binding of Palmitic acid and ZDHHC5 substrate domain. **d** In the presence of 25 μM Lomitapide, binding of SSTR5 (307–362 aa) and ZDHHC5 substrate domain. **e** In the presence of 25 μM Lomitapide, binding of Palmitic acid and ZDHHC5 substrate domain. **f** Co-IP assay showing that SSTR5 interacted with ZDHHC5 in HEK293T cell, and lomitapide inhibits their combination. **g** As shown in the mechanism diagram, we propose that Lomitapide inhibits pancreatic cancer proliferation by competitively binding the ZDHHC5 substrate to the binding region of SSTR5 and Palmitic acid, preventing palmitoylation.

We collect 2513 FDA-approved small molecule drugs (DRUGBANK, 2021 Dec) and perform molecular docking to the 3D model of ZDHHC5’s substrate binding domain. After virtual screening, we choose the top 5 FDA-approved drugs as candidates for subsequent experimental verification. Then, we test the Kd value between 5 candidates and ZDHHC5’s substrate binding domain and identify Lomitapide as a very potent ligand (Kd = 509 nM, Fig. 5a). The predicted binding pocket of Lomitapide on ZDHHC5’s substrate binding domain is consisted of His132, CYS134, PRO135, TRP136, PHE196, PRO199, LEU203, PHE206 and THR217 (Fig. 4e). Based on the above computational results, we hypothesize that Lomitapide binds to ZDHHC5’s substrate binding domain and it inhibits the ZDHHC5-mediated palmitoylation through competitively inhibit the interaction between ZDHHC5 and SSTR5/palmitic acid (Fig. 4f). In addition, The primary target of lomitapide is MTP (https://go.drugbank.com/drugs/DB08827). The RNA and protein expression of MTP are mainly in gastrointestinal tract and liver, but not in pancreas (https://www.proteinatlas.org/search/MTP) (Supplementary Fig. 3a–e). Further, western blotting showed that MTP protein was almost not expressed in five pancreatic cancer cell lines (SW1990, AsPC-1, BxPC-3, Mia PaCa-2, and Panc-1) compared to human hepatoma cells Hep3B and Huh-7(MTP positive controls). Therefore, we exclude the influence of lomitapide’s primary target on the subsequent studies of its antiproliferation effect on pancreatic cancer cell lines (Supplementary Fig. 3f, g).

### Mechanism of Lomitapide’s inhibition of palmitoylation mediated by ZDHHC5

Firstly, we test the Kd value of Lomitapide and ZDHHC5’s substrate binding domain and identify Lomitapide as a very potent ligand (Kd = 509 nM, Fig. 5a). Then, Kd values of SSTR5 protein fragment (cytoplasmic tail 307-364aa) and ZDHHC5’s substrate binding domain (Kd = 2.06 nM, Fig. 5b) as well as palmitic acid and ZDHHC5’s substrate binding domain (Kd = 88 nM, Fig. 5c) were determined respectively. Subsequently, we test the Kd value of SSTR5 and ZDHHC5’s substrate binding domain (Kd = 22.5 nM, Fig. 5d) as well as palmitic acid and ZDHHC5’s substrate binding domain (Kd = 134 μM, Fig. 5e) in the presence of 25 μM Lomitapide, respectively. The Kd value of SSTR5 and ZDHHC5 increase about 11-fold in the presence of 25 μM Lomitapide, while The Kd value of palmitic acid and ZDHHC5 increase more than 1000-fold in the presence of 25 μM Lomitapide. These results indicate that Lomitapide inhibits the binding of SSTR5/palmitic acid to ZDHHC5. As confirmation of these in vivo interactions, we next conducted coimmunoprecipitation (Co-IP) assays, the proteins SSTR5 and ZDHHC5 were transfected into HEK293T cells by plasmid. Co-IP assay showing that SSTR5 interacted with ZDHHC5 in HEK293T cell, and lomitapide inhibits their combination (Fig. 5f). In addition, Panc-1 cells were treated with a combination of 25 μM SSTR5 agonist (BIM-23190) and different concentrations of Lomitapide to achieve the combined effect of the two drugs (Supplementary Fig. 3h). Here, we propose the most probable mechanism of Lomitapide’s inhibition of palmitoylation mediated by ZDHHC5 is through blocking the binding between both SSTR5 and ZDHHC5 substrate binding domain as well as the binding between palmitic acid and ZDHHC5 substrate binding domain (Fig. 5g).

### In vitro evaluation of Lomitapide

**Fig. 6:**
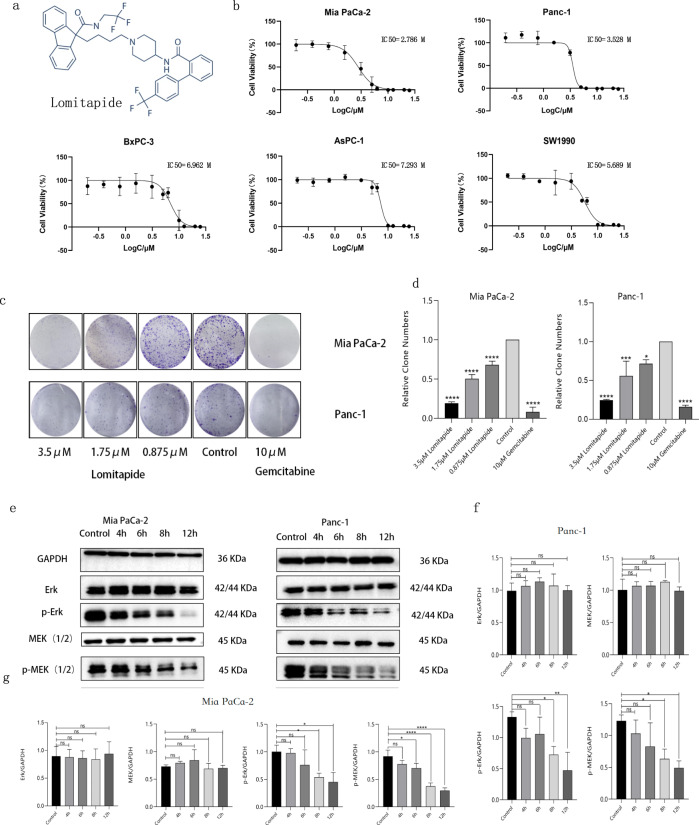
In vitro evaluation of Lomitapide. **a** Lomitapide structure. **b** The effects of Lomitapide on five pancreatic cancer cell lines were measured by MTT assay. **c**, **d** Treated Panc-1 and Mia Paca-2 cells with 3.5 μM, 1.75 μM, and 0.875 μM of Lomitapide, as well as blank group and positive control (Gemcitabine HCL, 10 μM), and found that Lomitapide significantly inhibited the colony formation of these two cell lines in a dose-dependent manner. **e**–**g** We extracted proteins from Mia PaCa-2 cells and Panc-1 cells 4 h, 6 h, 8 h and 12 h after administration respectively for Western blotting experiment which showed lomitapide presumably inhibit cell proliferation through p-MEK and p-ERK by inhibiting protein phosphorylation, and the degree of inhibition is time dependent. Data are expressed as mean ± SEM (*n* = 3) *p < 0.05, **p < 0.01, ***p < 0.001 in **b**, **d**, **f**, **g**.

We first measured the inhibition effect of Lomitapide (chemical structure, Fig. 6a) on 5 pancreatic cell lines. The IC50 values of Lomitapide on SW1990, AsPC-1, BxPC-3, Mia PaCa-2, and Panc-1 cells range from 2.786–7.293 μM, respectively (Fig. 6b). We then treated Panc-1 and Mia Paca-2 cells with 3.5 μM, 1.75 μM, and 0.875 μM of Lomitapide, and found that Lomitapide significantly inhibited the colony formation of Mia PaCa-2, and Panc-1 in a dose-dependent manner (Fig. 6c, d). Further, we extracted proteins from Mia PaCa-2 cells and Panc-1 cells 4 h, 6 h, 8 h and 12 h after Lomitapide administration respectively for Western blotting. The results show that Lomitapide inhibits cell proliferation through p-MEK and p-ERK by inhibiting protein phosphorylation as a time dependent manner. (Fig. 6e–g).

### Evaluation of Lomitapide in vivo

**Fig. 7:**
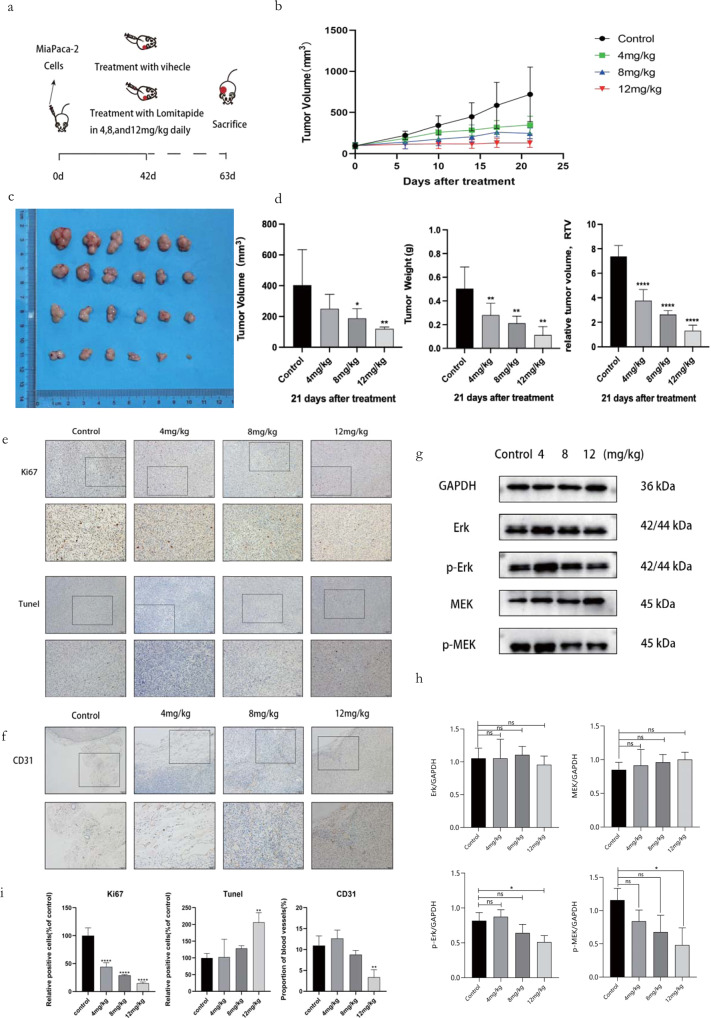
In vivo evaluation of Lomitapide. **a** Groups of athymic BALB/c nude mice were injected subcutaneously With 1 × 10^8^ Mia PaCa-2 cells and treatments were initiated 42 days later. Mice in the three treatment groups were intraperitoneally injected with lomitapide at doses of 12 mg/kg/day, 8 mg/kg/day and 4 mg/kg/day respectively, while mice in the control group were intraperitoneally injected with normal saline. On day 63, animals were sacrifificed and tumors were collected for analysis. **b** Tumor volume of the tumor xenograft. **c** Primary tumor gross appearance of tumor xenografts excised on day 39. **d** Tumor volume, weight and relative tumor volume of the tumor xenograft. **e** IHC staining detected Ki67, Tunel expression in the indicated tumors. The top row, the scale is 100 μm, the second row, the scale is 50 μm. **f** IHC staining detected CD31 expression in the indicated tumors. The top row, the scale is 100 μm, the second row, the scale is 50 μm. **g**, **h** Western blot assay showed Erk, p-Erk, MEK, P-MEK protein level in tumor tissue of mice. **i** The statistical graph of **e** and **f**. Data are expressed as mean ± SEM (*n* = 3) *p < 0.05, **p < 0.01, ***p < 0.001 in **h** and **i**. Data are expressed as mean ± SEM (*n* = 6) *p < 0.05, **p < 0.01, ***p < 0.001 in **b**, **d**.

We choose Mia PaCa-2 cell line for xenograft experiment in BALB/c Female nude mice. Mice in the treatment groups were intraperitoneally injected with Lomitapide at dosages of 12 mg/kg/day, 8 mg/kg/day and 4 mg/kg/day, respectively, while mice in the control group were intraperitoneally injected with normal saline (Fig. 7a). The results indicate that tumor volume/weight of mice in three treatment groups are much smaller than those of control group (Fig. 7b–d). And the expression of Ki67 was significantly downregulated, suggesting that Lomitapide inhibited the proliferation of pancreatic cancer, and its inhibitory effect increased in a dosage dependent manner. Tunel expression was upregulated, suggesting that Lomitapide promoted the apoptosis of pancreatic cancer cells in a dosage dependent manner (Fig. 7e). Lomitapide also has certain inhibitory effect on angiogenesis (Fig. 7f). Furthermore, tumor tissues of these four groups were collected for further Western blotting indicating that both decreased p-MEK and p-ERK (12 mg/kg group, Fig. 7g, h). In summary, we prove that pharmacological blockade of ZDHHC5 with Lomitapide resulted in attenuated cancer cell growth and proliferation.

## Discussion

The continued proliferation of cancer cells, their resistance to cell death, and their increased ability to metastasize are due to changes in the activity of intracellular signal transduction, metabolism, and gene regulation networks [29]. In both normal and cancer cells, the function of these network proteins is modulated by post-translational modifications. Hundreds of unique post-translational modifications affecting thousands of proteins have been identified in mammalian cells [30]. Lipid modification is an important post-translational modification of proteins. Lipid proteins usually have a higher affinity to non-polar structures (such as lipid bilayer), which have a significant impact on the localization, transport, interaction and stability of proteins in cells [31]. S-palmitoylation is the most common lipid modification of proteins, also known as protein palmitoylation [32], the thioesterification reaction of palmitate ester with cysteine residue is catalyzed by palmityl transferase. The family of palmityl transferases is known as the DHHC family due to the presence of a common catalytic motif.

In 2018, KO et al. [33] predicted 299 oncogenic genes through SwissPalm (Protein palmitoylation Database), among which 26% of the coding proteins may be modified by palmitoylation, such as small GTPases of RAS family as a typical example [34, 35]. In addition, it has been found that knocking down ZDHHC20 reduces the proliferation of KRAS mutant NSCLC and increases their sensitivity to PI3K inhibitors [36]. Overexpression of ZDHHC14 can also slow down the formation of xenograft tumors and induce apoptosis of cultured HEK-293T cells [37]. Palmitoylation of cell adhesion molecule C (JAM-C) can enhance the connection between NSCLC cells and inhibit their migration. Knockdown of ZDHHC7 in NSCLC reduces palmitoylation of JAM-C, thereby enhancing migration of NSCLC cells [38]. ZDHHC2 is also associated with inhibiting the metastasis of cancer cells, and is usually missing in metastatic liver cancer, colon cancer, prostate cancer, breast cancer and non-small cell lung cancer. Overexpression of ZDHHC2 can inhibit the migration and invasion of liver cancer cells [39, 40]. The above studies indicate that S-palmitoylation is closely related to the occurrence of cancer, and the dysregulation of ZDHHC family proteins promotes the development of cancer. In the present study, we performed single-cell transcriptome sequencing on pancreatic cancer patient samples and identified ZDHHC5 as a potential target for anti-proliferation of pancreatic cancer cells by silencing ZDHHC5 in cancer cells which results in dramatic antitumor effects. Based on our single-cell transcriptome sequencing data, we propose that ZDHHC5 is a potential anti-pancreatic cancer target. Furthermore, we perform in vitro and in vivo experiments to show that knockdown ZDHHC5 significantly inhibit the proliferation of pancreatic cancer cells.

SSTR5 is a proven anti-proliferation receptor in pancreatic cells, Previous studies have reported that the SSTR5 agonist somatostatin analogue AN-238 can significantly inhibit the proliferation of pancreatic cancer cells in vitro and in vivo [25]. Another study showed that various mutations in the tail of SSTR5 inhibit the anti-proliferation of SSTR5 in pancreatic cancer cells [27]. In addition, clinical research showed that SSTR5 agonist pasireotide has been applied in a Phase II clinical study to treat metastatic pancreatic cancer (Clinical Number: NCT01385956). We hypothesized that antagonizing ZDHHC5-mediated palmitoylation on the cytoplasmic tail of SSTR5 may represent a more efficacious approach for the treatment of pancreatic cancer. However, there is no approved drugs for inhibition of protein palmitoylation to date. Hence, we screen an orphan drug (Lomitapide) as a ZDHHC5 inhibitor, the first small molecule antagonist of ZDHHC5. According to the above results, we suggest that the mechanism of Lomitapide’s inhibiting pancreatic cancer cell is through inhibiting both SSTR5 and palmitic acid binding to ZDHHC5.

Lomitapide is a microsomal triglyceride transfer protein (MTP) inhibitor indicated as an adjunct to a low-fat diet and other lipid-lowering treatments which was approved by FDA in 2007. Here, we report the repositioning of Lomitapide as an inhibitor of ZDHHC5 and propose that it may be able to prevent palmitoylation on SSTR5. Pharmacological blockade of ZDHHC5 with Lomitapide results in attenuated cancer cell growth and proliferation which collectively contributes to antitumor effects in vitro and in vivo. Furthermore, we excluded the effect of Lomitapide’s original target MTP, in our study about inhibiting pancreatic cancer proliferation. In the future study of Lomitapide, clinical studies can be performed to observe Lomitapide’s clinical effects.

## Conclusions

In conclusion, we first compared single-cell transcriptome data from pancreatic cancer and normal pancreatic tissues and found that ZDHHC5 is a potential target for inhibiting pancreatic cancer cell proliferation. We evaluated the antitumor effects of silencing ZDHHC5 in vitro and in vivo, confirming that ZDHHC5 is a potential target for inhibiting pancreatic cancer cell proliferation (Figs. 1–3).

Based on the 3D model of ZDHHC5 substrate binding domain, we hypothesize that: 1) ZDHHC5 mediated palmitoylation of SSTR5 cytoplasmic tail is around the Cys134 of ZDHHC5; 2) Repositioning a small molecule drug binding on the pocket around Cys134 of ZDHHC5 is probably able to block its palmitoylation reaction (Fig. 4). Hence, we report that Lomitapide (an orphan drug) has been screened as an inhibitor of ZDHHC5, speculating that it may prevent palmitoylation on SSTR5 (Fig. 5). Pharmacological blockade of ZDHHC5 by Lomitapide results in reduced cancer cell growth and proliferation, which together contribute to antitumor effects in vitro and in vivo (Figs. 6, 7). To our knowledge, this is the first study to demonstrate the efficacy of a pharmacological inhibitor of ZDHHC5 in pancreatic cancer, representing a new class of palmitoylated targeted therapies and laying the framework for paradigm-shifting palmitoylated therapy targeting cancer cells.

## Methods

### Reagents and materials

Lomitapide (CSA No.182431-12-5, HPLC:99.37%), Gemcitabine HCL (CAS No.122111-03-9, HPLC:99.96%), palmitic acid (CSA No.57-10-3, HPLC:99.87%), BIM-23190 (Cat No:HY-P3124A, LCMS:98.82%).

Two human pancreatic cancer samples obtained from JIANGSU CANCER HOSPITAL for sc-RNA seq were collected from a woman and a man undergoing surgery for pancreatic cancer patients.

### Ethic statement

The study was conducted in accordance with the Declaration of Helsinki, and the protocol was approved by the Medical Ethics Committee of the JIANGSU CANCER HOSPITAL and performed according to institutional guidelines (No. YB M-10-09).

### Cell culture

Five pancreatic cancer cell lines (SW1990, AsPC-1, BxPC-3, Mia PaCa-2, and Panc-1) and normal HPDE cells were purchased from the Cell Repository, Chinese Academy of Sciences (Shanghai, China). AsPC-1 and BxPC-3 cells were cultured in supplemented RPMI 1640 (Gibco, Gaithersburg, MD, USA) containing 10% fetal bovine serum and antibiotics. SW1990, Mia PaCa-2 and Panc-1 cells were cultured in supplemented Dulbecco Modified Eagle’s Medium (Gibco) containing 10% fetal bovine serum and antibiotics. All cells were maintained at 37 °C/5% CO_2_.

### Single-cell transcriptome sequencing and analysis

See Online methods. In Brief, two tumor samples of pancreatic cancer patients were collected for single-cell transcriptome sequencing. In comparison, two normal pancreatic tissue single-cell transcriptome data (GSA: CRA001160, Genome Sequence Archive under project PRJCA001063) [41] were downloaded and compared with samples of pancreatic cancer patients. The cDNA/DNA/Small RNA libraries were sequenced on the Illumina sequencing platform by Guangzhou Kidio Biotechnology Co., Ltd. (Guangzhou, China). The raw reads were deposited into the NCBI Sequence Read Archive database (Accession Number: SRR19328914-SRR19328921). More detailed data and analytical methods are available from the corresponding authors upon reasonable request (Additional file 1: Supplemental materials and methods).

### Procedure for protein in silico modeling, virtual screening and protein-protein docking

The 3D model of ZDHHC5 substrate binding domain (1-219 aa) was modelled by I-TASSER under default parameters. For drug repositioning, 2513 files (sdf format) of FDA-approved small molecule drugs (DRUGBANK database, 2021 Dec) were collected as virtual screening library. Then, Autodock vina (Scripps Institute: http://vina.scripps.edu/) was used to virtually screen 2513 FDA-approved drugs targeting to ZDHHC5 substrate binding domain. The docked results with binding energy less than −8.5 kcal/mol were selected as candidates for experimental verification. Protein-protein docking was performed with Cluspro 2.0 (https://cluspro.bu.edu/login.php?redir=/home.php) for the interaction between ZDHHC5 substrate binding domain and SSTR5 cytoplasmic tail (307-362 aa).

### siRNA

siRNA (Shanghai GenePharma Co., Ltd).

ZDHHC5 siRNA#1 (Forward: 5′-CCCACAUUAUGGGUGUGUUTT-3′ Reverse 5′-AACACACCCAUAAUGUGGGTT-3′) siRNA#2 (Forward: 5′-GCUUGGAACCAGAGAGCUUTT-3′) Reverse: (5′-AAGCUCUCUGGUUCCAAGCTT-3′).

### shRNA

The shRNAs vectors targeting ZDHHC5 were purchased from OBiO Technology (Shanghai) Corp., Ltd. For the establishment of ZDHHC5 knockdown pancreatic cancer cell lines, MIA Paca-2 and Panc-1 cells were transduced with a lentivirus vector containing negative control shRNA (NC) or shRNAs against ZDHHC5 (Sh-ZDHHC5-1 and Sh-ZDHHC5-2) for 48 h. Morphology of MIA Paca-2 and Panc-1 cells was captured. mRNA expression of ZDHHC5 was analyzed by Real Time PCR, knockdown efficiency on protein level was detected using Western blot.

### RT-qPCR

RNA was extracted using Trizol (R401-01, Vazyme, Nanjing, China). Reverse transcription was performed with reverse transcription kit (R323-01, Vazyme, Nanjing, China). ZDHHC5 mRNA expression after transfection was analyzed by RT-qPCR, AceQ qPCR SYBR Green Master Mix Kit (Q341-02, Vazyme, Nanjing, China). Fluorescence quantitative PCR primer sequence as below

For GAPDH (Forward: 5′-GTCTCCTCTGACTTCAACAGCG-3′, Reverse 5′-ACCACCCTGTTGCTGTAGCCAA-3′). ZDHHC5 (Forward: 5′-AACTGTATTGGTCGCCGGAAC-3′, Reverse: 5′-AACACACCCATAATGTGGGCT-3′).

### Cell viability screen (MTT assay)

The culture medium containing 10% FBS was used to prepare a single-cell suspension, which was inoculated into a 96-well plate at a density of 2500 cells per well, with a volume of 200 μL. Cells were routinely cultured for 3 days, after which 20 μL MTT solution (prepared with 5 mg/mL PBS) was added to each well for further incubation. After 4 hours, the incubation was terminated, the supernatant was removed carefully, and centrifugation was performed for suspended cells before removal of the supernatant inside the well. Next, 100 μL dimethyl sulfoxide was added to each well, followed by shaking for 10 min in order to fully dissolve the crystals. The optical density of each well was measured at 490 nm wavelength using enzyme-linked immunosorbent assay, and the results were recorded.

### Measurement of cell proliferation (colony-formation assay)

Treated PANC-1 and Mia PaCa-2 cells were harvested and reseeded at a concentration of 2000 cells/well in 6-well plates. Cells were treated with 3.5 μM, 1.75 μM, and 0.875 μM of Lomitapide after 24 h. Gemcitabine (10 μM) was used as the positive control. After 2 weeks of treatment, the cell colonies were stained with 0.5% Crystal Violet and the colonies were counted. The total number of colonies was counted using light microscopy from more than five different fields of view.

### The growth curve

Take 20 μL and add it into the blood counting plate to count; The total amount of cells and culture medium (1500–2000 cells per well, 180 μL serum-free culture medium) for five 96-well plates were calculated, and the corresponding number of cells were added to the total culture medium amount of 96-well plates for re-suspension. After uniform blowing, the cells were placed into the 96-well plates for 12 h culture. After the cells were firmly adhered to the wall, the culture was continued, and a 96-well plate was taken out at 24, 48, 72, 96 and 120 h, and 20 μL CCK8 solution was added to each well with a determinator. The cells were placed in the incubator under dark conditions for 2 h. Remove the plate cover and put the plate into a microplate reader to detect the absorbance value at 450 nm wavelength. Data were collected for four days and growth curves were plotted using absorbance values.

### Western blotting

Cultured cells or homogenized mouse tissues were lysed in RIPA lysis buffer (Shanghai Biyuntian Biotechnology Co., LTD) containing protease inhibitors (thermo, 50913) and phosphatase inhibitors (thermo, 81660). Proteins were resolved on 10–15% and transferred to a nitrocellulose membrane using the Trans-Blot Turbo Transfer System (Bio-Rad). Membranes were blocked with 5% BSA in TBST and incubated with the primary antibody at 4 °C overnight, followed by incubation with the secondary antibody conjugated with horseradish peroxidase (HRP). The bands were visualized with enhanced chemiluminescence substrate (ThermoFisher Scientific). Primary antibodies used are as follows: antibodies against ERK1/2 (1:1000, Cell Signaling Technology, 4695 S, RRID:AB_390779), Phospho-ERK1/2 (1:1000, Cell Signaling Technology, 4370 S, RRID:AB_2315112), Phospho-AKT (1:1000, Cell Signaling Technology, 4060 S, RRID:AB_2315049), AKT (1:1000, Cell Signaling Technology, 9272 S, RRID:AB_329827), GAPDH (1:2000, Proteintech, 60004-1-IG, RRID: AB_2107436), MEK1/2 (1:1000, Cell Signaling Technology, 4694 S, RRID:AB_10695868), Phospho-MEK1/2(Ser217/221) (1:1000, Cell Signaling Technology, 9154 T, RRID:AB_2138017), Phospho-c-Raf (Ser259)(1:1000, Cell Signaling Technology, 9421 S, RRID:AB_330759), MTP (1:200, Santa Cruz Biotechnology, SC-515742), ZDHHC5 (1:1000, Proteintech, 21324-1-AP, RRID:AB_10732816), SSTR5 (1:1000, Proteintech, 66772-1-lg, RRID:AB_2882118). Immunoblotting images were obtained using the ChemiDoc Touch Imaging System (Bio-Rad) and Image Lab Touch software (Bio-Rad).

### Transfections

For plasmid transfections, HEK293T cells were seeded 3000–4000 cells/well in 6-well plates and 24 h post plating, cells were transfected with 2.5 μg of the indicated plasmid using Lipofectamine 3000 as per manufacturer’s instructions (ThermoFisher). Transfection medium was exchanged to fresh medium 8–10 h post-transfection. Cells were harvested 2–4 days post-transfection for downstream assays.

### Coimmunoprecipitation (Co-IP)

Co-IP assays were carried out using Co-Immunoprecipitation Kits of DIA.AN (Wuhan, Cat#:KM0134) following the manufacturer’s instructions. Cells were lysed using IP-lysis buffer (50 mM Tris HCl, pH 7.4, 150 mM NaCl, 1 mM EDTA, and 1%Triton X-100) at 4 °C for 30 min. After protein quantification, 40 μl cleaned magnetic beads were taken and incubated with cell lysates containing 30 μg protein at 25 °C for 15 min. After separation, the magnetic beads were cleaned with PBS for 4 times. Cell lysates were mixed with 4 μg antibody or IgG by gently rotation at 25 °C for 15 min. After separation, the magnetic beads were cleaned with PBST for 4 times. Then resuspended in 10 μl ddH2O and 10 μl 2X SDS buffer. Resuspended beads were boiled for 15 min at 100 °C, centrifuged and supernatant was processed for Western blotting as indicated.

### Microscale thermophoresis (MST) analysis

The MST analysis was performed using a NanoTemper Monolith NT.115 instrument (NanoTemper Technologies GmbH). The protein fragment of ZDHHC5 (The target protein 60-148aa was expressed using Yeast) and SSTR5 were labeled and purified using monolith TMRED Red-NHS 2nd Generation protein labeling kit (NanoTemper Technologies GmbH). Measuring the affinity of ZDHHC5 with Lomitapide: 20 nM of the labeled ZDHHC5 was mixed with Lomitapide prepared in 16 different serial concentrations (0.27 nM to 8.75 μM) at RT in 1.05*PBS. Measuring the affinity of ZDHHC5 with SSTR5: 20 nM of the labeled ZDHHC5 was mixed with unlabeled SSTR5 prepared in 16 different serial concentrations (The maximum concentration is 200 nM) at RT in 1.05*PBS. Measuring the affinity of ZDHHC5 with palmitic acid: 20 nM of the labeled ZDHHC5 was mixed with palmitic acid prepared in 16 different serial concentrations (The maximum concentration is 500 μM) at RT in 1.05*PBS. Measuring the affinity between ZDHHC5 and SSTR5 in the presence of Lomitapide: 20 nM of the labeled SSTR5 and 25 μM Lomitapide were mixed with unlabeled ZDHHC5 prepared in 16 different serial concentrations (The maximum concentration is 200 nM) at RT in 1.05*PBS. Measuring the affinity between ZDHHC5 and palmitic acid in the presence of Lomitapide: 20 nM of the labeled ZDHHC5 and 25 μM Lomitapide were mixed with unlabeled palmitic acid prepared in 16 different serial concentrations (The maximum concentration is 500 μM) at RT in 1.05*PBS. The mixtures were then loaded into standard glass capillaries (Monolith NT.115 Capillaries), After blowing evenly, the machine was tested and the Initial Fluorescence Analysis program (LED 20%, medium MST power) was used for Analysis through the MO. Control software (NanoTemper Technologies GmbH). For each set of experiments, two to four replicate MST measurements were conducted. Datasets were processed with the MO. Affinity Analysis software (NanoTemper Technologies GmbH).

### In vivo tumor study (silencing ZDHHC5)

BALB/c Female nude mice (4–5 weeks of age, 18–20 g) were purchased from Nanjing Zhongzhu Biotechnology Co., Ltd. Mice were raised in the barrier system of Animal Experiment Center of China Pharmaceutical University. Feeding temperature: 18–22 °C, humidity: about 50%. Replace sterile pad twice a week, change water every two days (sterile), and give proper feed every day (sterile). Mice were randomly divided into two groups. ZDHHC5-knockdown Mia PaCa-2 cells (1 × 10^8^cells) in 100 μL of PBS mix with 100 µL Matrigel(BD Matrigel™ Basement Membrane Matirx, Corning, 356234) were injected subcutaneously into the right root of experimental mice (*n* = 6), Model mice (*n* = 6) were injected with Mia PaCa-2 cells (1 × 10^8^cells). Subsequently, tumor size was measured with a vernier caliper every 1–2 days. When the volume of subcutaneous transplanted tumor in mice reached about 5.0 × 10^2^ mm^3^, the nude mice were sacrificed by cervical dislocation and the subcutaneous transplanted tumor was removed for further inoculation. At the end of the experiment, after weighing the mice and measuring the size of the tumor, the mice were sacrificed by removing the cervical vertebra, and the whole mice were collected and photographed. Then the tumor was completely stripped and weighed, and the tumor was collected and photographed. Local mouse tumor tissues were soaked in 4% paraformaldehyde fixative solution for paraffin embedding, followed by HE staining and immunohistochemical staining for sections. Another part of the tumor tissue was wrapped in tin foil and frozen in a refrigerator at −80 °C. All animal experimentation was conducted after approval of Ethics Committee of China Pharmaceutical University.

### In vivo tumor study (Lomitapide)

BALB/c Female nude mice (4–5 weeks of age, 18–20 g) were purchased from Hangzhou Ziyuan Laboratory Animal Technology Co., Ltd. Mice were kept in a temperature-humidity light-controlled environment and fed standard food and water. Mia PaCa-2 cells (1 × 10^8^cells) in 100 μL of PBS mix with 100 µL Matrigel (BD Matrigel™ Basement Membrane Matirx, Corning, 356234) were injected subcutaneously into the right armpit of BALB/c nude mice (*n* = 5). Subsequently, tumor size was measured with a vernier caliper every 1–2 days. When the volume of subcutaneous transplanted tumor in mice reached about 5.0 × 10^2^ mm^3^, the nude mice were sacrificed by cervical dislocation and the subcutaneous transplanted tumor was removed for further inoculation of BALB/c nude mice (*n* = 24). After the tumor was removed, it was cut open to select the tumor tissue with good growth without degeneration and necrosis, which was red and fish-like, and cut into small pieces (about 5 × 5 × 5 mm). A small incision was made under the right armpit. A small piece was taken with ophthalmic tweezers and placed under the skin within the incision. The mice were raised under pathogen-free conditions and monitored for tumor growth every week. When tumor volume averages 100 mm^3^, mice were randomly divided into four groups. Subcutaneous tumor size was measured with digital calipers at the indicated endpoints. The tumor volume was calculated using the formula: volume = (length × width^2^)/2. Mice in the three treatment groups were intraperitoneally injected with lomitapide at doses of 12 mg/kg/day, 8 mg/kg/day and 4 mg/kg/day respectively, while mice in the control group were intraperitoneally injected with normal saline. After three weeks of treatment, the mice were sacrificed and the tumor tissue was removed, weighed, and extracted for paraffin embedding. Local mouse tumor tissues were soaked in 4% paraformaldehyde fixative solution for paraffin embedding, followed by immunohistochemical staining of sections. Another part of the tumor tissue was wrapped in tin foil and frozen in a refrigerator at −80 °C. All animal experimentation was conducted after approval of Ethics Committee of China Pharmaceutical University.

### Immunohistochemistry

Immunohistochemistry has been performed as previously described using the following antibodies:

KI67 (GB111499, 1:300, Servicebio, Wuhan, China)

CD31 (GB113151, 1:500, Servicebio, Wuhan, China)

DABtunel (G1507-50, 1:5:50, Servicebio, Wuhan, China).

### Statistical analysis

Data were presented as mean ± SEM and statistical analysis were performed by GraphPad Prism8.0 software (GraphPad Software Inc., CA, USA). Unpaired Student’s *t* test was used to compare differences between two groups, while analysis of variance (ANOVA) was used for comparisons among multiple groups. The differences were considered to be statistically significant for *P*-values <0.05 (*), <0.01 (**), <0.001 (***), and <0.0001 (****).

## Supplementary information

Supplementary legends

Supplementary Figure 1

Supplementary Figure 2

Supplementary Figure 3

Supplementary file 1

Supplementary file 2

Additional Tables

Original Data File

## Data Availability

The datasets used during the current study are available from the corresponding author on reasonable request.

## Code availability

The software we use to screen drugs is Pyrx and the modeling software is I-TASSER, they are both open source software.
