# Supplementary material for: Repositioning Lomitapide to block ZDHHC5-dependant palmitoylation on SSTR5 leads to anti-proliferation effect in preclinical pancreatic cancer models
Source: Cell Death Discov. 2023 Feb 11;9:60. doi: 10.1038/s41420-023-01359-4 (PMC9922277; doi:10.1038/s41420-023-01359-4)
Supplement: Supplementary file 1 — Supplementary legends [file 41420_2023_1359_MOESM1_ESM.docx]

Supplementary figure 1 Pseudotime trajectory analysis of tumor cells,In general, ZDHHC5 expression is associated with genes in tumor proliferation pathway.

**a**-**c**: Pseudotime trajectory analysis of tumor cells shows that ZDHHC5 expresses in every branch suggesting that ZDHHC5 plays important roles in the whole differentiation process of tumor cells.

**d**-**h**: ZDHHC5 expression is associated with genes in tumor proliferation pathway.

**i**: we hypothesize thatZDHHC5 is a potential gene leading pancreatic cancer to proliferate through PI3K-Akt proliferative pathway

Supplementary figure 2 The correlation between ZDHHC5 and proliferation gene was analyzed in tumor and non-tumor cells.

**a**: we perform correlation analysis between ZDHHC5 and the proliferative genes and find that ZDHHC5 is significantly correlated with Akt, c-Raf, MEK and ERK in tumor cells

**b**: For non-tumor cells, ZDHHC5 is significantly correlated with Akt and c-Raf, but not with MEK and ERK

Supplementary figure 3 Target exclusion of MTP,and drug combination experiment.

**a**: Anatomogram of MTTP expression in human tissue

**b**: Protein expression data is shown for each of the 44 tissues.

**c**: RNA expression of MTTP in different cancers category, RNA-seq data from The Cancer Genome Atlas (TCGA)

**d**:RNA expression of MTTP in different tissues,the HPA RNA-seq tissue data is reported as nTPM (normalized protein-coding transcripts per million)

**e**: Antibody staining of standard cancer tissue samples for liver and pancreatic cancers

**f**,**g**: Western blot assay was used to detect the expression of MTP protein in two HCC cell lines (Hep3B and Huh-7) and five PDAC cell lines (SW1990, AsPC-1, BxPC-3, Mia PaCa-2, and Panc-1), Hep3B and Huh-7 were used as positive controls.

**h**:Panc-1 cells were treated with a combination of 25μM SSTR5 agonist（BIM-23190） and different concentrations of Lomitapide to achieve the combined effect of the two drugs,Jin’s formula Q value method was used to evaluate .It can be seen that Q is basically between 0.85 and 1.15,therefore, it is concluded that combination of the two drugs have addition effect.

**i**:Co-IP assay showing that SSTR5 interacted with ZDHHC5 in five PDAC cell lines ( AsPC-1, BxPC-3,SW1990, Mia PaCa-2, and Panc-1)

Data are expressed as mean±SEM (n=3) *p<0.05, **p<0.01, ***p<0.001 in **g** and **h**.
