# Supplementary figures and images for: Repositioning Lomitapide to block ZDHHC5-dependant palmitoylation on SSTR5 leads to anti-proliferation effect in preclinical pancreatic cancer models
Source: Cell Death Discov. 2023 Feb 11;9:60. doi: 10.1038/s41420-023-01359-4 (PMC9922277; doi:10.1038/s41420-023-01359-4)

Supplementary Figure 1

Pancreatic cancer tissue

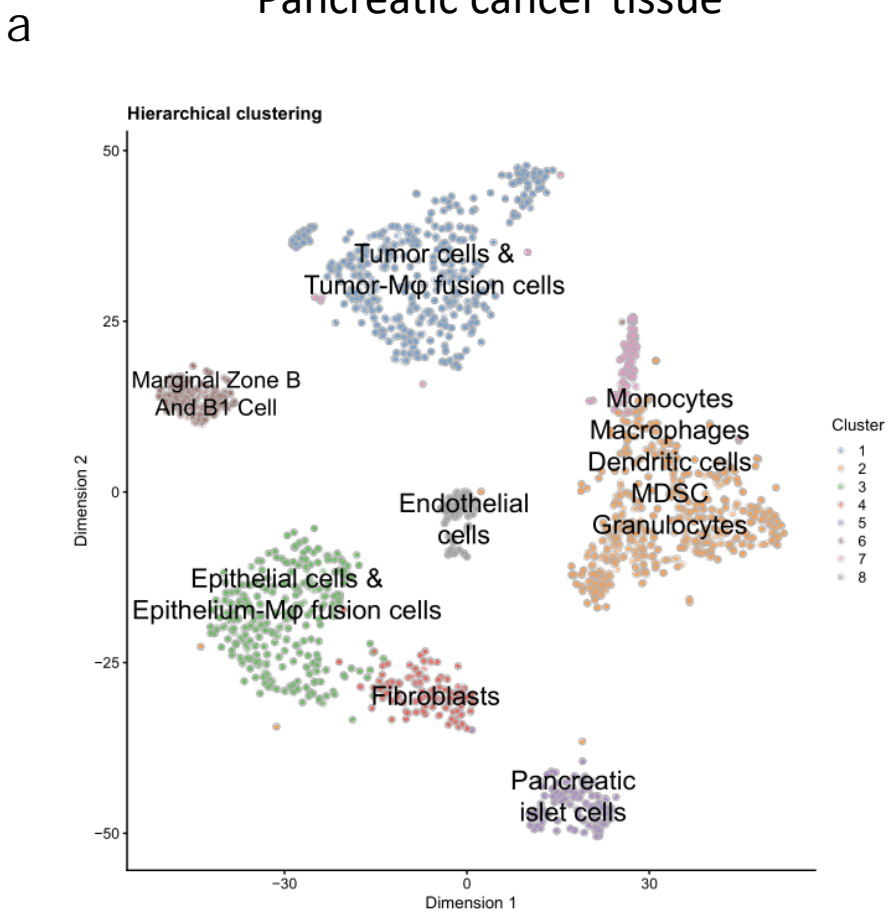

Tumor cells

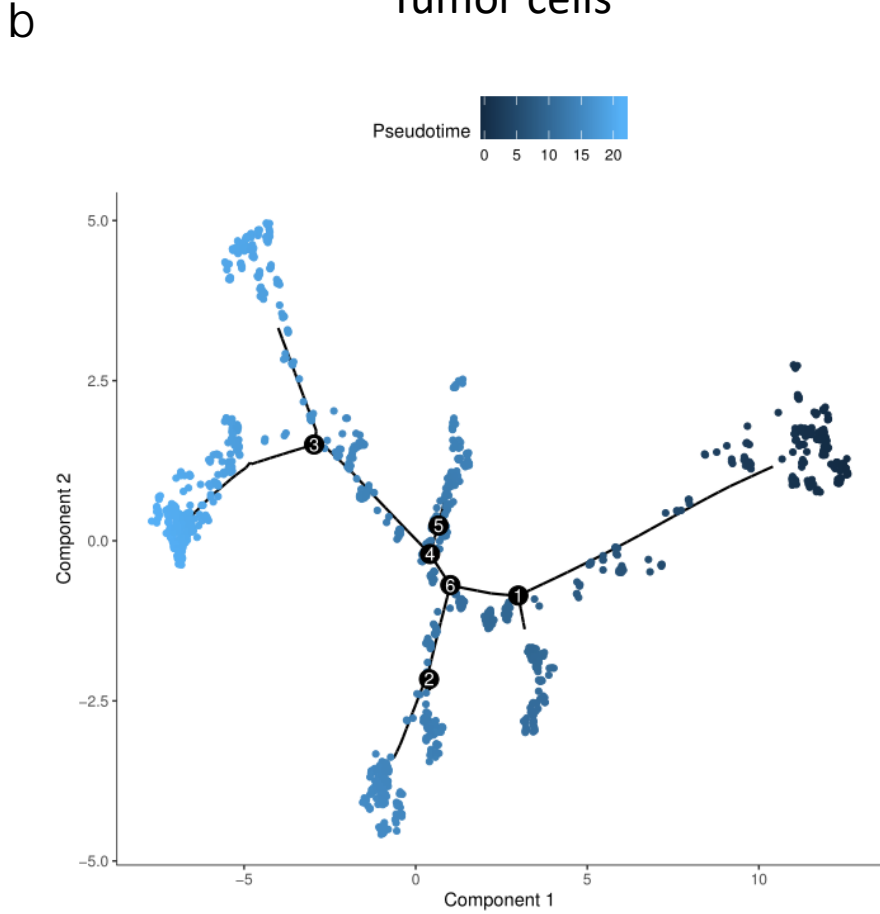

Tumor cells

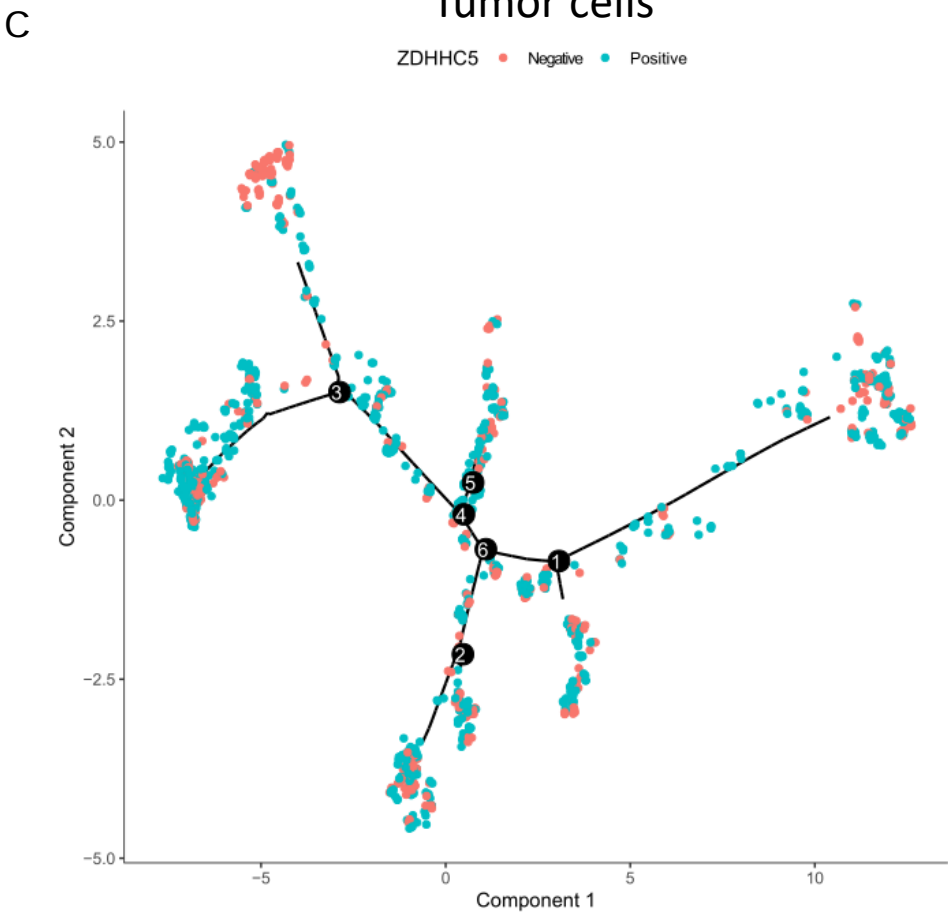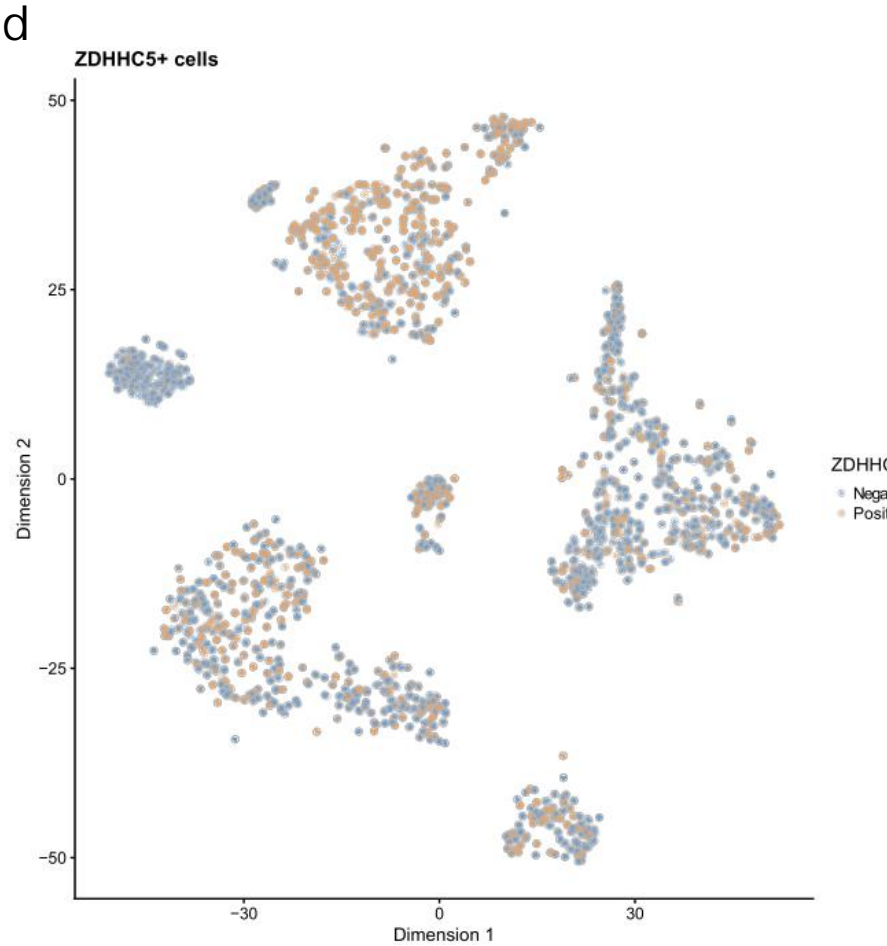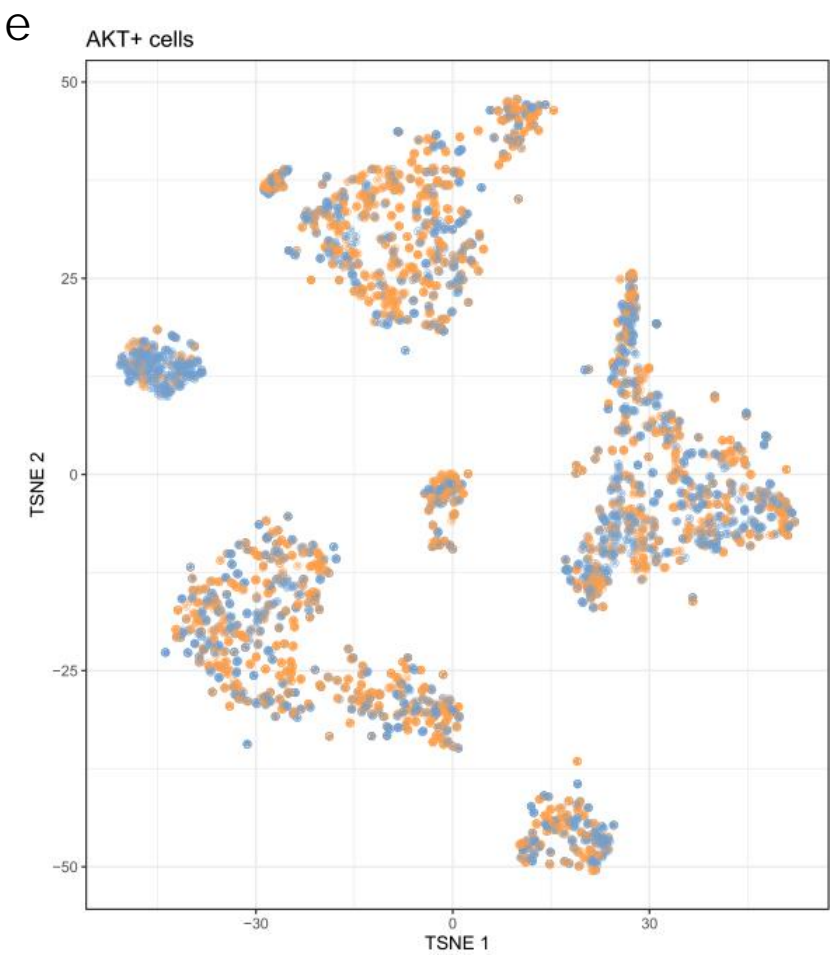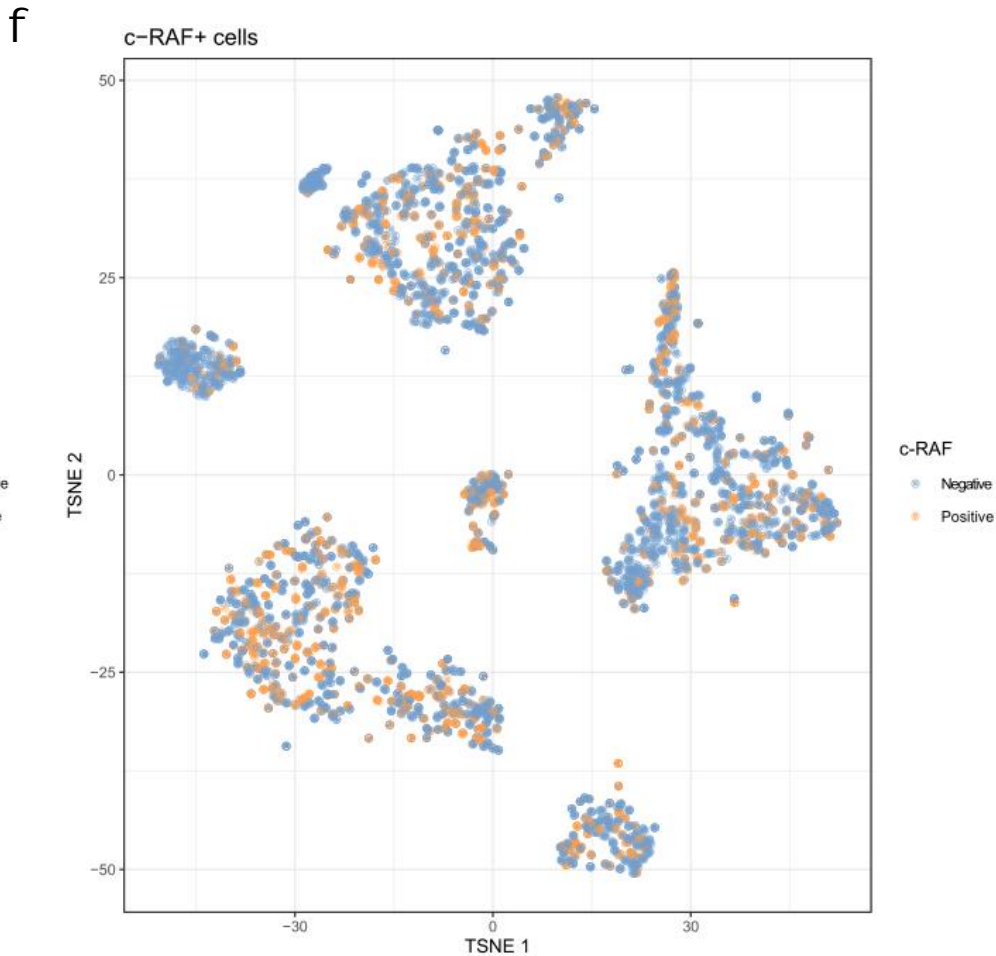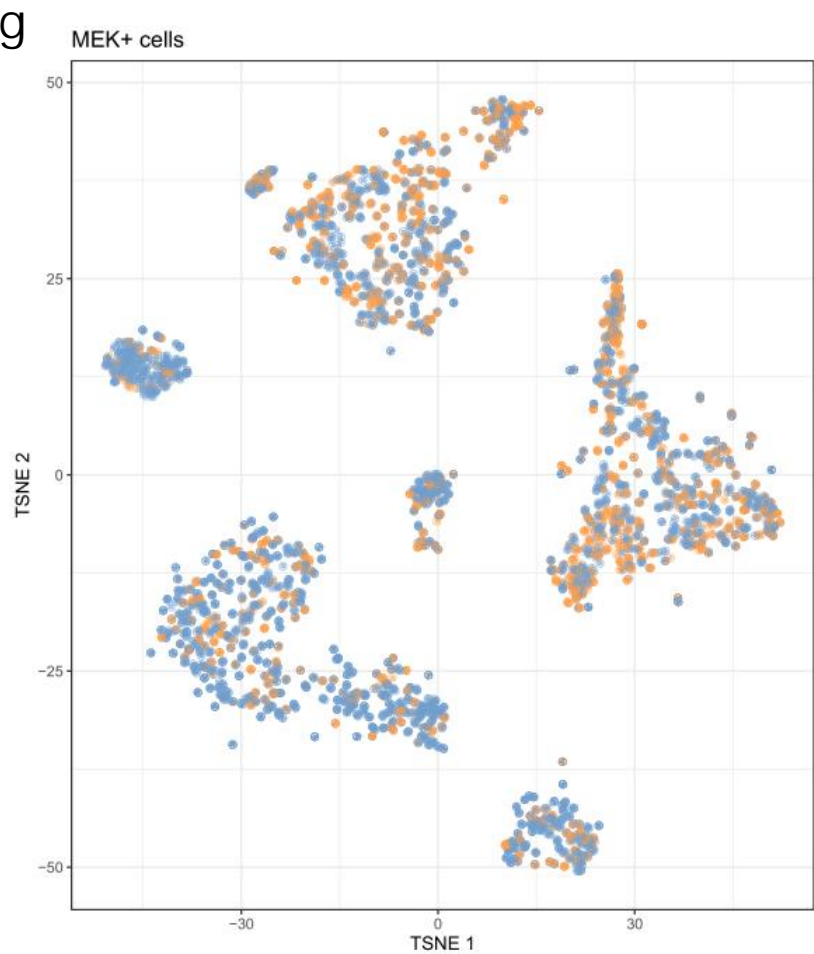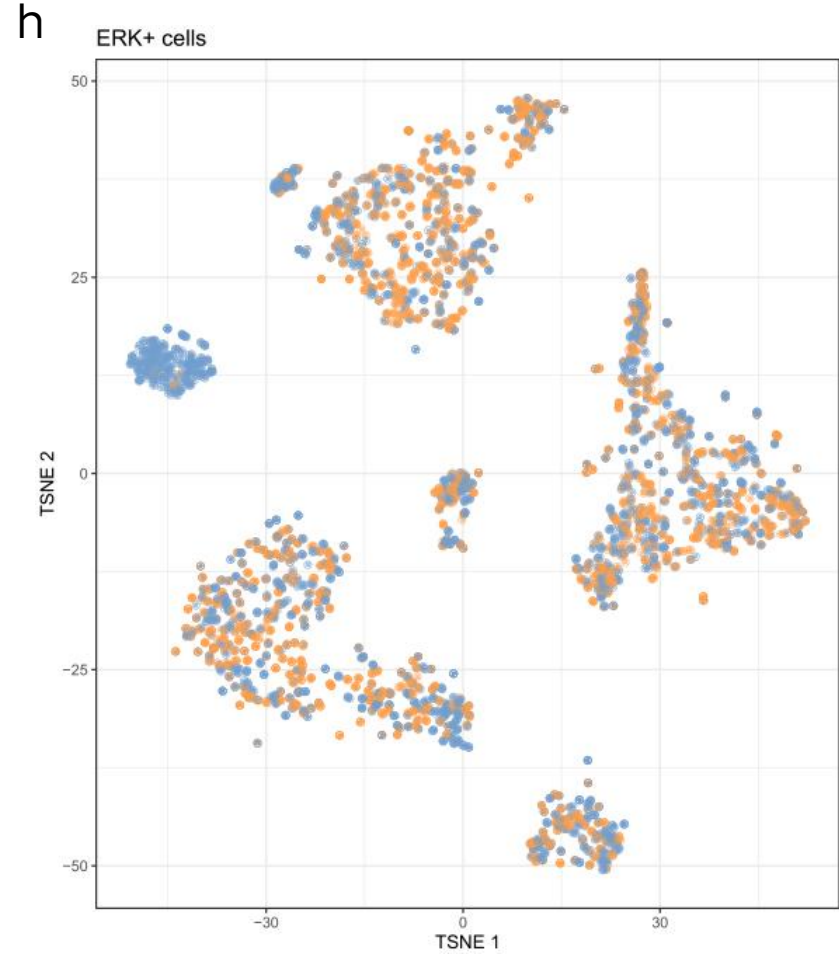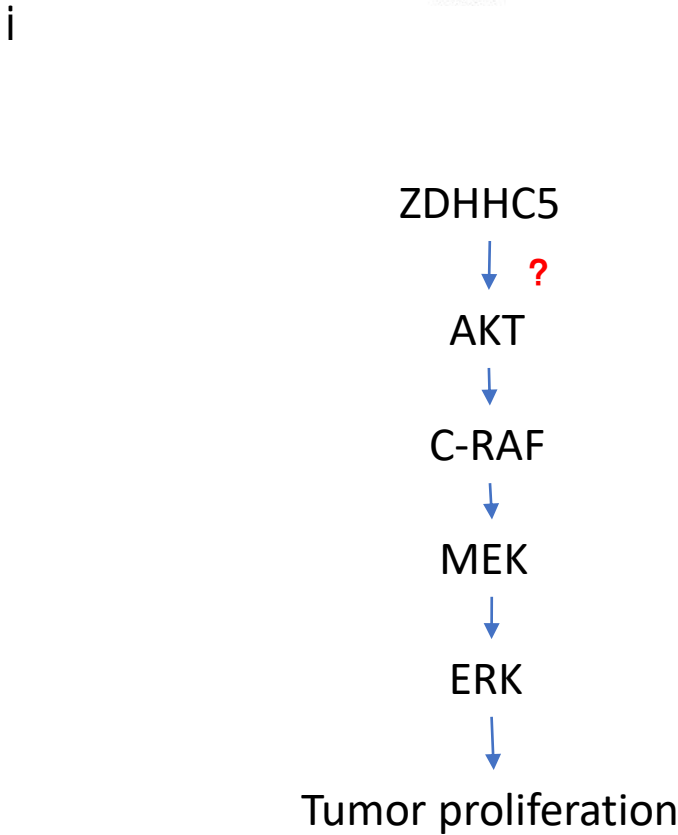

Supplement: Supplementary file 2 — Supplementary Figure 1 [file 41420_2023_1359_MOESM2_ESM.pdf]

a

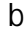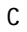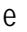

f

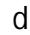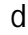

## h

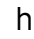

**i**

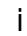

Supplement: Supplementary file 4 — Supplementary Figure 3 [file 41420_2023_1359_MOESM4_ESM.pdf]
