# Supplementary material for: Repositioning Lomitapide to block ZDHHC5-dependant palmitoylation on SSTR5 leads to anti-proliferation effect in preclinical pancreatic cancer models
Source: Cell Death Discov. 2023 Feb 11;9:60. doi: 10.1038/s41420-023-01359-4 (PMC9922277; doi:10.1038/s41420-023-01359-4)
Supplement: Supplementary file 3 — Supplementary Figure 2 [file 41420_2023_1359_MOESM3_ESM.pdf]

Supplementary Figure 2

a

Proliferative gene expression in tumor cells (cluster1)

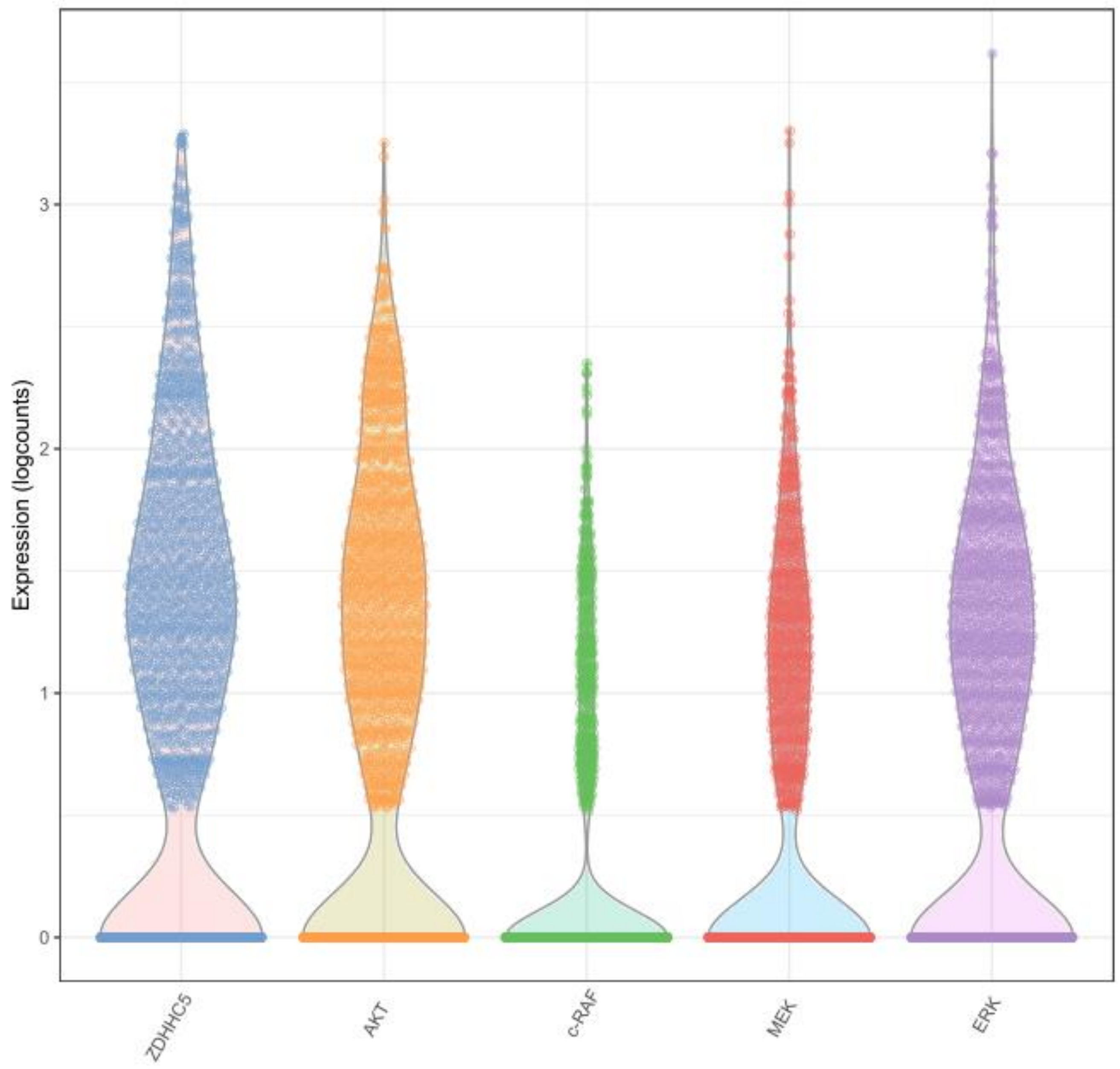

number of tumor cell(cluster1)	1183
number of tumor cell(ZDHHC5+)	798
number of tumor cell(AKT+)	744
number of tumor cell(c-RAF+)	333
number of tumor cell(MEK+)	529
number of tumor cell(ERK+)	722

gene correlation analysis in tumor cells(cluster1)	Pearson's correlation coefficient	p value
ZDHHC5 vs AKT	0.135	<0.001
ZDHHC5 vs c-RAF	0.111	<0.001
ZDHHC5 vs MEK	0.096	<0.001
ZDHHC5 vs ERK	0.139	<0.001

b

Proliferative gene expression in non-tumor cells (ZDHHC5+)

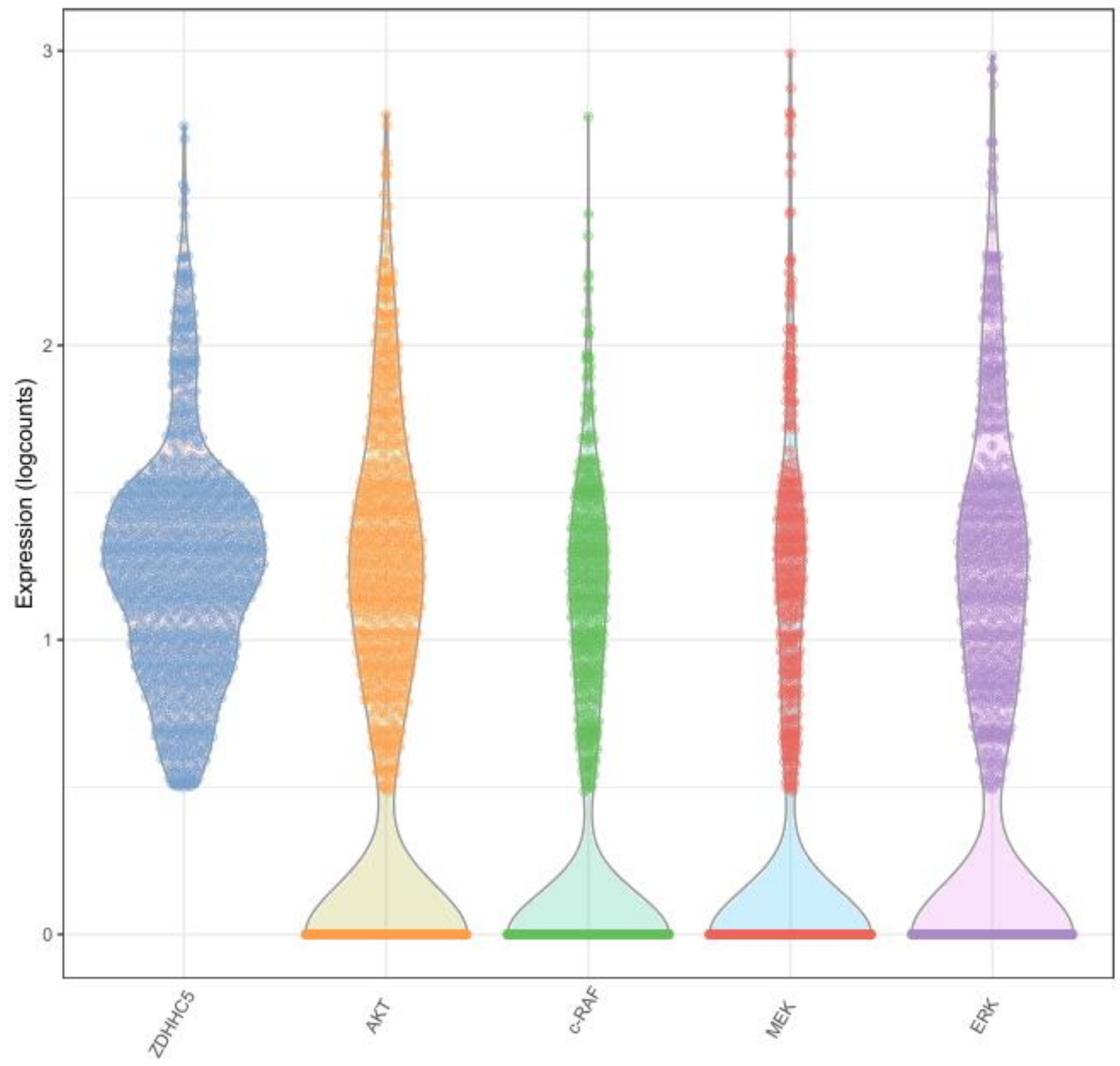

number of non-tumor cell(ZDHHC5+)	1006
number of non-tumor cell(AKT+)	550
number of non-tumor cell(c-RAF+)	391
number of non-tumor cell(MEK+)	332
number of non-tumor cell(ERK+)	542

gene corelation analysis in non-tumor cells(ZDHHC5+)	Pearson's corelation coeffecient	p value
ZDHHC5 vs AKT	0.127	<0.001
ZDHHC5 vs c-RAF	0.118	<0.001
ZDHHC5 vs MEK	0.075	>0.01
ZDHHC5 vs ERK	0.035	>0.05
