# Supplementary material for: Repositioning Lomitapide to block ZDHHC5-dependant palmitoylation on SSTR5 leads to anti-proliferation effect in preclinical pancreatic cancer models
Source: Cell Death Discov. 2023 Feb 11;9:60. doi: 10.1038/s41420-023-01359-4 (PMC9922277; doi:10.1038/s41420-023-01359-4)
Supplement: Supplementary file 5 — Supplementary file 1 [file 41420_2023_1359_MOESM5_ESM.docx]

## Additional file 1: Supplemental materials and methods. Single cell sequences data and analytical methods.

**1. Tissue dissociation and preparation of single-cell suspension**

Fresh tumor biopsies were minced into 1 mm cubic pieces, followed by enzymatic digestion in 1 mL digestion medium (HBSS containing 0.1g/mL Collagenase IV, 0.05 g/mL Neutral Protease, 0.1 g/mL Hyaluronidase and 2000 U/mL DNase I) for 75 min at 37℃.Following digesting, the digest tissues were transferred into a 15 mL tube and 3 mL of 1× HBSS was added. Then, the cells were collected by centrifugation at 100 g at 4 °C for 1 min. After centrifugation, the cells in the supernatants were collected and transferred to a new 15 mL tube. The pullete was resuspended with PBS and then passed through a 70-μm filter. The suspension was pelleted at 500g at 4 °C for 5 min with the supernatants collected before. Cells were then resuspended in 1 mL PBS, followed by lysis using Red Blood Cell Lysis Buffer, and centrifuged at 500 g at 4 °C for 5 min. The pellet was washed twice with PBS. The concentration of cencer sample was adjusted to 2×106 cells/mL while the normal sample was adjusted to 1×105 cells/mL prior to undergo sc-RNA seq. The single-cell suspension preparation was carried out by the technicist of GENEDENOVO Company.

**2. Preparation of scRNA-seq libraries**

The scRNA-seq libraries were prepared using the Chromium™ Single Cell 3' Solution which ensure that the input cells are paired with a unique barcode. The single cell was encapsulated into oil droplet together with barcoded bead and RT reagents. The final oil droplet is named GEM (Gel bead in Emulsion). Following reverse transcription, emulsions were broken and cDNA purified followed by PCR amplification (98℃ for 45 s; [98℃ for 20 s, 67℃ for 30 s, 72℃ for 1 min] x 14; 72℃ for 1 min). For gene expression library construction, the amplified cDNA was fragmented into 200~300 bp and end-repaired. The P5/P7 sequence adapters, index and sequencing binding sites Reads1/Reads2 were added at the end of each cDNA chain. The cDNA were amplified through PCR amplification to preparation of scRNA-seq libraries.

**3. Sequencing**

The scRNA libraries were sequenced on an Illumina NextSeq to a minimum sequencing depth of 20,000 reads per cell using read lengths of 26bp read 1, 8bp i7 index, 98bp read 2.

**4. Gene expression algorithms overview**

**4.1 Genome alignment**

The scRNA-seq reads were aligned to the GRCh38 reference genome and quantified using Cell Ranger count (10x Genomics, version 3.1.0). Cell Ranger uses an aligner called [STAR](https://github.com/alexdobin/STAR), which peforms splicing-aware alignment of reads to the genome. Cell Ranger then uses the transcript annotation GTF to bucket the reads into exonic, intronic, and intergenic, and by whether the reads align (confidently) to the genome. A read is exonic if at least 50% of it intersects an exon, intronic if it is non-exonic and intersects an intron, and intergenic otherwise.

**4.2 MAPQ adjustment**

For reads that align to a single exonic locus but also align to 1 or more non-exonic loci, the exonic locus is prioritized and the read is considered to be confidently mapped to the exonic locus with MAPQ 255.

**4.3 Transcriptome alignment**

Cell Ranger further aligns exonic reads to annotated transcripts, looking for compatibility. A read that is compatible with the exons of an annotated transcript, and aligned to the same strand, is considered mapped to the transcriptome. If the read is compatible with a single gene annotation, it is considered uniquely (confidently) mapped to the transcriptome. These confidently mapped reads are the only ones considered for UMI counting.

**4.4 UMI counting**

Before counting UMIs, Cell Ranger attempts to correct for sequencing errors in the UMI sequences. Reads that were confidently mapped to the transcriptome are placed into groups that share the same barcode, UMI, and gene annotation. If two groups of reads have the same barcode and gene, but their UMIs differ by a single base (i.e., are Hamming distance 1 apart), then one of the UMIs was likely introduced by a substitution error in sequencing. In this case, the UMI of the less-supported read group is corrected to the UMI with higher support.

Cell Ranger again groups the reads by barcode, UMI (possibly corrected), and gene annotation. If two or more groups of reads have the same barcode and UMI, but different gene annotations, the gene annotation with the most supporting reads is kept for UMI counting, and the other read groups are discarded. In case of a tie for maximal read support, all read groups are discarded, as the gene cannot be confidently assigned.

After these two filtering steps, each observed barcode, UMI, gene combination is recorded as a UMI count in the [unfiltered feature-barcode matrix](https://support.10xgenomics.com/single-cell-gene-expression/software/pipelines/latest/output/matrices). The number of reads supporting each counted UMI is also recorded in the [molecule info file](https://support.10xgenomics.com/single-cell-gene-expression/software/pipelines/latest/output/molecule_info).

**4.5 Calling cell barcodes**

Cell Ranger 3.1.0 introduces an improved cell-calling algorithm that is better able to identify populations of low RNA content cells, especially when low RNA content cells are mixed into a population of high RNA content cells. The algorithm has two key steps:1.It uses a cutoff based on total UMI counts of each barcode to identify cells. This step identifies the primary mode of high RNA content cells.2.Then the algorithm uses the RNA profile of each remaining barcode to determine if it is an “empty" or a cell containing partition. This second step captures low RNA content cells whose total UMI counts may be similar to empty GEMs.

In the first step, the original Cell Ranger cell calling algorithm is used to identify the primary mode of high RNA content cells, using a cutoff based on the total UMI count for each barcode. Cell Ranger takes as input the expected number of recovered cells, N. Let m be the 99th percentile of the top N barcodes by total UMI counts. All barcodes whose total UMI counts exceed m/10 are called as cells in the first pass.

In the second step, a set of barcodes with low UMI counts that likely represent ‘empty’ GEM partitions is selected. A model of the RNA profile of selected barcodes is created. This model, called the background model, is a multinomial distribution over genes. It uses [Simple Good-Turing smoothing](https://en.wikipedia.org/wiki/Good%E2%80%93Turing_frequency_estimation) to provide a non-zero model estimate for genes that were not observed in the representative empty GEM set. Finally, the RNA profile of each barcode not called as a cell in the first step is compared to the background model. Barcodes whose RNA profile strongly disagrees with the background model are added to the set of positive cell calls. This second step identifies cells that are clearly distinguishable from the profile of empty GEMs, even though they may have much lower RNA content than the largest cells in the experiment.

**4.6 Cell filtering**

All additional analyses were performed using edger, scater, scran, monocle, ggplot and pheatmap. These R packages are used to easily explore QC metrics and filter cells based on any user-defined criteria. A few QC metrics [commonly used](https://www.ncbi.nlm.nih.gov/pmc/articles/PMC4758103/) by the community include:1.The number of unique genes detected in each cell. Low-quality cells or empty droplets will often have very few genes. Cell doublets or multiplets may exhibit an aberrantly high gene count.2.Low-quality / dying cells often exhibit extensive mitochondrial contamination. So, we filter cells that have unique feature counts less than 140 and cells that have >5% mitochondrial counts.

**4.7 Normalizing the data**

After removing unwanted cells from the dataset, the next step is to normalize the data. By default, we employ a global-scaling normalization method “LogNormalize” that normalizes the feature expression measurements for each cell by the total expression, multiplies this by a scale factor (10,000 by default), and log-transforms the result.

**4.8 Identification of highly variable features (feature selection)**

We next calculate a subset of features that exhibit high cell-to-cell variation in the dataset (i.e, they are highly expressed in some cells, and lowly expressed in others). By default, we return 2,000 features per dataset. These will be used in downstream analysis, like PCA.

**4.9 Scaling the data**

We apply a linear transformation (‘scaling’) that is a standard pre-processing step prior to dimensional reduction techniques like PCA. We Shift the expression of each gene, so that the mean expression across cells is 0.And then we scale the expression of each gene, so that the variance across cells is 1.This step gives equal weight in downstream analyses, so that highly-expressed genes do not dominate.

**4.10 Principal component analysis**

Scaled data were used input into a principal component analysis (PCA) on the basis of variable genes. To overcome the extensive technical noise in any single feature for scRNA-seq data, Seurat clusters cells based on their PCA scores, with each PC essentially representing a ‘metafeature’ that combines information across a correlated feature set. The top principal components therefore represent a robust compression of the dataset. We implemented a resampling test inspired by the JackStraw procedure. We randomly permute a subset of the data (1% by default) and rerun PCA, constructing a ‘null distribution’ of feature scores, and repeat this procedure. We identify ‘significant’ PCs as those who have a strong enrichment of low p-value features. The most significant (p<1e-5) principal component (PC) was selected from the PCA analysis results for subsequent clustering and cluster analysis.

**4.11 Cluster the cells**

Seurat v3 applies a graph-based clustering approach, the distance metric which drives the clustering analysis (based on previously identified PCs) remains the same. Briefly, these methods embed cells in a graph structure - for example a S-nearest neighbor (SNN) graph, with edges drawn between cells with similar feature expression patterns, and then attempt to partition this graph into highly interconnected ‘quasi-cliques’ or ‘communities’. We first construct a SNN graph based on the euclidean distance in PCA space, and refine the edge weights between any two cells based on the shared overlap in their local neighborhoods (Jaccard similarity).To cluster the cells, we next apply modularity optimization techniques such as the Louvain algorithm (default) or SLM, to iteratively group cells together, with the goal of optimizing the standard modularity function.

**4.12 Non-linear dimensional reduction (t-SNE)**

t-SNE is done by Scran. Cells within the graph-based clusters determined above are co-localize on these dimension reduction plots. As input to the t-SNE, we use the same PCs as input to the clustering analysis.

**4.13 Pseudotime trajectory** **analysis**

Pseudotime trajectory analysis (Monocle package) was performed on tumor cell cluster to show trajectory of tumor cell differentiation. Furthermore, ZDHHC5 expression positive/negative cell were highlighted in trajectory graph.
