# Supplementary material for: Repositioning Lomitapide to block ZDHHC5-dependant palmitoylation on SSTR5 leads to anti-proliferation effect in preclinical pancreatic cancer models
Source: Cell Death Discov. 2023 Feb 11;9:60. doi: 10.1038/s41420-023-01359-4 (PMC9922277; doi:10.1038/s41420-023-01359-4)
Supplement: Supplementary file 6 — Supplementary file 2 [file 41420_2023_1359_MOESM6_ESM.pdf]

T-Coffee alignment result

MSA

The multiple sequence alignment result as produced by T-coffee.

```
T-COFFEE, Version_11.00 (Version_11.00)
Cedric Notredame
SCORE=701
*
 BAD AVG GOOD
*
ZDHHC5    :  72
ZDHHC1    :  69
ZDHHC2    :  70
ZDHHC3    :  68
ZDHHC4    :  72
ZDHHC6    :  71
ZDHHC7    :  69
ZDHHC8    :  73
ZDHHC9    :  71
ZDHHC10   :  71
ZDHHC11   :  70
ZDHHC12   :  69
ZDHHC13   :  65
ZDHHC14   :  70
ZDHHC15   :  69
ZDHHC16   :  72
ZDHHC17   :  67
ZDHHC18   :  69
ZDHHC19   :  71
ZDHHC20   :  70
ZDHHC21   :  70
ZDHHC22   :  65
ZDHHC23   :  69
ZDHHC24   :  73
cons      :  70

ZDHHC5     MPAESG-KRFK-----------PS------------------------------------------------
ZDHHC1     MYKMNI-CNK---PSN---KTAPE--------------------------K-S-------V-----------
ZDHHC2     MAPSGP-G----------------------------------------------------------------
ZDHHC3     MMLIPT-HHFRNI-ERKPEYLQPE--------------------------K---------------------
ZDHHC4     MDFLVL-FL---------------------------------------------------------------
ZDHHC6     MGTFCS-VIK--------------------------------------------------------------
ZDHHC7     MOPSGH--RLRDV-EHHPLLAEND--------------------------N---------------------
ZDHHC8     MPRSPG-TRLK-----------PA------------------------------------------------
ZDHHC9     MSVMVV-RK---------------------------------------------------------------
ZDHHC10    MSVMVV-RK---------------------------------------------------------------
ZDHHC11    MDTRSG-SOCSVTPEA---ILNNE--------------------------K-L-------------------
ZDHHC12    MAPWAL-LSP--G-----------------------------------------------------------
ZDHHC13    MEGPGLGSOCRNH-SH-----GPH------PPGFGRYGICAHENKELANAREA-------LPL---------
ZDHHC14    MPPGGG-GPMK-----------DC--------------------------E-Y-------SQIS--------
ZDHHC15    MRRGWK-MAL--------------------------------------------------------------
ZDHHC16    MRGORS-LLL-----------GPA--------------------------R-LCLRLLLLL-----------
ZDHHC17    MOREEG-FNTKMA-D------GPD------EYDTE--------------AG-C-------VPLL--------
ZDHHC18    MKDCEY-OOIS-----------PGAAPLPASPGARRPGP-----------A-A-------SPTPGPGPAPPA
ZDHHC19    MTLLTD-AT---------------------------------------------------------------
ZDHHC20    MAPWTL------------------------------------------------------------------
ZDHHC21    MGLRIH------------------------------------------------------------------
ZDHHC22    MLALRL-LNVV-------------------------------------------------------------
ZDHHC23    MTOKGS-MKPV-------------------------------------------------------------
ZDHHC24    MGQPWA-AGST---DG-----APA--------------------------Q---------------------

cons       *


ZDHHC5     ------------------------------------------------------------------------
ZDHHC1     ------WTAPAQPSGPSPELOGO-RSR--------RNGW---------------------------------
ZDHHC2     ----------------S---SAR-RR--------CRRVL---------------------------------
ZDHHC3     -------------------------CVPPPYPGPVGTMW---------------------------------
ZDHHC4     ------------------------------------------------------------------------
ZDHHC6     ----------------F---ENL-OE--------LKRLC---------------------------------
ZDHHC7     -----------YDSSSSSS-SEA-DV--------ADRVW---------------------------------
ZDHHC8     ------------------------------------------------------------------------
ZDHHC9     -------------------------KV--------TRKW---------------------------------
ZDHHC10    -------------------------KV--------TRKW---------------------------------
ZDHHC11    ------------------VLPPR-ISR--------VNGW---------------------------------
ZDHHC12    --------------------VLV-RT--------GHTVL---------------------------------
ZDHHC13    ------------------------------------------------IEDSSNCDIVKATQYGIFERCKEL
ZDHHC14    -------THSSSPMESPHKKK-K-IAA--------RRKW---------------------------------
ZDHHC15    ----------------S---GGL-RC--------CRRVL---------------------------------
ZDHHC16    ---------------------GYRRRCPPLLRGL-VORWRYGK-----------------------------
ZDHHC17    ---------------HPEEIKPQ-SHY--------NHGYGEPLGRKTHIDDYSTWDIVKATQYGIYERCREL
```

```
ZDHHC18   APAPPRWSSSGSGSGSGSGSL-G-RRP--------RRKW---------------------------------
ZDHHC19   -------------------------PL--------VKEP---------------------------------
ZDHHC20   ----------------------W-RC--------CQRVV---------------------------------
ZDHHC21   ------------------------------------------------------------------------
ZDHHC22   ------------------------------------------------------------------------
ZDHHC23   -----------------------------------KKKK---------------------------------
ZDHHC24   ------------------------------------------------------------------------

cons


ZDHHC5    ------------------------------------------------------------------------
ZDHHC1    --------------------SW--------------------------------------------------
ZDHHC2    ------------------------------------------------------------------------
ZDHHC3    --------------------FI--------------------------------------------------
ZDHHC4    -------------------------------FYLAS--VLM-------------------------------
ZDHHC6    ------------------------------------------------------------------------
ZDHHC7    --------------------FI--------------------------------------------------
ZDHHC8    ------------------------------------------------------------------------
ZDHHC9    --------------------EK--------------------------------------------------
ZDHHC10   --------------------EK--------------------------------------------------
ZDHHC11   --------------------SL--------------------------------------------------
ZDHHC12   ------------------------------------------------------------------------
ZDHHC13   VEAGYDVRQPDKENVSLLHWAAINNRLDLVKFYISKGAVVDQLGGDLNSTPLHWAIRQGHLPMVILLLQHGA
ZDHHC14   --------------------EV--------------------------------------------------
ZDHHC15   ------------------------------------------------------------------------
ZDHHC16   ------------VCLRSLLYNS--------------------------------------------------
ZDHHC17   VEAGYDVRQPDKENVTLLHWAAINNRIDLVKYYISKGAIVDQLGGDLNSTPLHWATRQGHLSMVVQLMKYGA
ZDHHC18   --------------------EV--------------------------------------------------
ZDHHC19   --------------------HP--------------------------------------------------
ZDHHC20   ------------------------------------------------------------------------
ZDHHC21   --------------------FV--------------------------------------------------
ZDHHC22   ------------------------------------------------------------------------
ZDHHC23   --------------------TE--------------------------------------------------
ZDHHC24   ------------------------------------------------------------------------

cons


ZDHHC5    ------------------------------------------------------------------------
ZDHHC1    ----PP------------------------------------------------------------------
ZDHHC2    ------------------------------------------------------------------------
ZDHHC3    ----RDG--C--------------------------------------------------------------
ZDHHC4    --------GLVL-----------ICV----------------------------------------------
ZDHHC6    ------------------------------------------------------------------------
ZDHHC7    ----RDG--C--------------------------------------------------------------
ZDHHC8    ------------------------------------------------------------------------
ZDHHC9    ----LP--GRNT----------FCCDGRVMMA----------------------------------------
ZDHHC10   ----LP--GRNT----------FCCDGRVMMA----------------------------------------
ZDHHC11   ----PL------------------------------------------------------------------
ZDHHC12   ------------------------------------------------------------------------
ZDHHC13   DPTLID--GEGF-----------SSIHLAVLF----------------------------------------
ZDHHC14   ----FP--GRNK----------FFCNGRIMMA----------------------------------------
ZDHHC15   ------------------------------------------------------------------------
ZDHHC16   ----FG--GSDTAVDAAFEPVYWLVD-NV--I----------------------------------------
ZDHHC17   DPSLID--GEGC-----------SCIHLAAOF----------------------------------------
ZDHHC18   ----FP--GRNR----------FYCGGRLMLA----------------------------------------
ZDHHC19   ----LPLVPRPW------------------------------------------------------------
ZDHHC20   ------------------------------------------------------------------------
ZDHHC21   ----VDP--H--------------------------------------------------------------
ZDHHC22   ------------------------------------------------------------------------
ZDHHC23   ----EP--ELEP----------LCCCEYIDRNGEKNHVATCLCDCQDLDEGCDRWITCKSLQPETCERIMDT
ZDHHC24   ------------------------------------------------------------------------

cons


ZDHHC5    ------------------KYVP-VSA--AA------------------------------------------
ZDHHC1    ------------------HPLO-IVA--W-------------------------------------------
ZDHHC2    ------------------YWIP-VVF--IT------------------------------------------
ZDHHC3    ------------------GIAC-A-I--VT------------------------------------------
ZDHHC4    --------------------------------C---------------------------------------
ZDHHC6    ------------------HWGP-IIA--LG------------------------------------------
ZDHHC7    ------------------GMIC-A-V--MT------------------------------------------
ZDHHC8    ------------------KYIP-VAT--AA------------------------------------------
ZDHHC9    ------------------ROKG-IFY--LT--------------------------------------L---
ZDHHC10   ------------------ROKG-IFY--LT--------------------------------------L---
ZDHHC11   ------------------HYFQ-VVT--W-------------------------------------------
ZDHHC12   ------------------TWG-------IT------------------------------------------
ZDHHC13   ------------------OHMP-IIA--YL--ISKGQSVNMTDVNGQTPLMLSAHKVIGPEPTGFLLKFNPS
ZDHHC14   ------------------ROTG-VFY--LT--------------------------------------L---
ZDHHC15   ------------------SWVP-VLV--IV------------------------------------------
ZDHHC16   ------------------RWFG-VVF--VV--------------------------------------L---
ZDHHC17   ------------------GHTS-IVA--YL--IAKGQDVDMMDQNGMTPLMWAAYRTHSVDPTRLLLTFNVS
ZDHHC18   ------------------GHGG-VFA--LT--------------------------------------L---
ZDHHC19   -------------------FLP-SLFAAFN--------------------------------------V---
ZDHHC20   ------------------GWVP-VLF--IT------------------------------------------
ZDHHC21   ------------------GWCC-M-G--LI------------------------------------------
ZDHHC22   ------------------------------------------------------------------------
ZDHHC23   ISDRLRIPWLRGAKKVNISIIPPLVL--LPVFL---------------------------------------
ZDHHC24   --------------------LP-LV-------------------------------------------L---

cons


ZDHHC5    ---------------IFLVGA------------------------------------TTLFFAF-----TCP
ZDHHC1    ---------------LLYLFF------------------------------------AVIGFGI-----LVP
```

```
ZDHHC2    ----------------LLLGW------------------------------------SYYAYAI-OLC-IVS
ZDHHC3    ----------------WFLV-------------------------------------LYAEFVVLFVM-LIP
ZDHHC4    -------SKTHSLKGLARGGA------------------------------------OIFS-----------
ZDHHC6    ----------------VIAIC------------------------------------STMAMID-SVLWYWP
ZDHHC7    ----------------WLLV-------------------------------------AYADFVVTFVM-LLP
ZDHHC8    ---------------ALLVGS------------------------------------STLFFVF-----TCP
ZDHHC9    ---------------FLILGT------------------------------------CTLFFAF-----ECR
ZDHHC10   ---------------FLILGT------------------------------------CTLFFAF-----ECR
ZDHHC11   ---------------AVFVGL------------------------------------SSATFGI-----FIP
ZDHHC12   ----------------LVL------------------------------------------FLH-DTE-L-R
ZDHHC13   LNVVDKIHQNTPLHWAVAAGN------------------------------------VNAVDKL-----LEA
ZDHHC14   ---------------VLILVT------------------------------------SGLFFAF-----DCP
ZDHHC15   ----------------LVVLW------------------------------------SYYAYVF-ELC-LVT
ZDHHC16   ---------------VIVLTG------------------------------------SIVAIAY-----LCV
ZDHHC17   VNLGDKYHKNTALHWAVLAGN------------------------------------TTVISLL-----LEA
ZDHHC18   ---------------LLILTT------------------------------------TGLFFVF-----DCP
ZDHHC19   ---------------VLLVFF------------------------------------SGLFFAF-----PCR
ZDHHC20   ----------------FVVVW------------------------------------SYYAYVV-ELC-VFT
ZDHHC21   ----------------VFVW-------------------------------------LYNIVLIPKIV-LFP
ZDHHC22   -------------APAY------------------------------FLCISLVTFVLOLFLFL-PSMREDP
ZDHHC23   ----------HVASWHFLLGVVVLTSLPVLALWYYYLTHRRKEQTLFFLSLGLFSL-GYMYYVF-LQ--EVV
ZDHHC24   ---------------TALWAA---------------------------------------AVGL-----ELA

cons


ZDHHC5    ------------------------------------------------------------------------
ZDHHC1    ------------------------------------------------------------------------
ZDHHC2    ------------------------------------------------------------------------
ZDHHC3    ------------------------------------------------------------------------
ZDHHC4    ----------------------------CIIP--ECLQRAV-------------------------------
ZDHHC6    ------------------------------------------------------------------------
ZDHHC7    ------------------------------------------------------------------------
ZDHHC8    ------------------------------------------------------------------------
ZDHHC9    ------------------------------------------------------------------------
ZDHHC10   ------------------------------------------------------------------------
ZDHHC11   ------------------------------------------------------------------------
ZDHHC12   ------------------------------------------------------------------------
ZDHHC13   GSSLDIQNVKGETPLDMALQNKNQLIIHMLKT--EAKMRAN----------QKF--RLWRWLQKCELFLLLM
ZDHHC14   ------------------------------------------------------------------------
ZDHHC15   ------------------------------------------------------------------------
ZDHHC16   ------------------------------------------------------------------------
ZDHHC17   GANVDAQNIKGESALDLAKQRKNVWMINHLQEARQAKGYDNPSFLRKLKADKEFRQKV-----------MLG
ZDHHC18   ------------------------------------------------------------------------
ZDHHC19   ------------------------------------------------------------------------
ZDHHC20   ------------------------------------------------------------------------
ZDHHC21   ------------------------------------------------------------------------
ZDHHC22   ------------------------------------------------------------------------
ZDHHC23   ------------------------------------------------------------------------
ZDHHC24   ------------------------------------------------------------------------

cons


ZDHHC5    --------------G---LSL-YVS-----------------------------P-A---------------
ZDHHC1    --------------L---LPH-HWV-----------------------------P-A---------------
ZDHHC2    --------------M---ENTG-EO------------------------------VV---------------
ZDHHC3    --------------S---RDY-VYS------------------------------II---------------
ZDHHC4    --------------------------HG------------LLHYLFHTRNHTFIV-LHLVLQGMVYTEYTWE
ZDHHC6    --------------L---HTTG-GS------------------------------VN---------------
ZDHHC7    --------------S---KDF-WYS------------------------------VV---------------
ZDHHC8    --------------W---LTR-AVS-----------------------------P-A---------------
ZDHHC9    --------------Y---LAV-OLS-----------------------------P-A---------------
ZDHHC10   --------------Y---LAV-OLS-----------------------------P-A---------------
ZDHHC11   --------------F---LPH-AWK-----------------------------Y-I---------------
ZDHHC12   --------------O---WEEQ-GE------------------------------LL---------------
ZDHHC13   LSVITMWAIGYILDF---NSD-SWLLKGCLLVTLFFLTSLFPRFLVGYKNLVYLP-TAFLLSSVFWIFMTWF
ZDHHC14   --------------Y---LAV-KIT-----------------------------P-A---------------
ZDHHC15   --------------V---LSPA-EK------------------------------VI---------------
ZDHHC16   --------------L---PLI-LRT-----------------------------Y-S---------------
ZDHHC17   TPFLVIWLVGFIADL---NID-SWLIKGLMYGGVWATVQFLSKSFFDHSMHSALP-LGIYLATKFWMYVTWF
ZDHHC18   --------------Y---LAR-KLT-----------------------------L-A---------------
ZDHHC19   --------------W---LAO-NGE-----------------------------W-A---------------
ZDHHC20   --------------IFGNEENG-KT------------------------------VV---------------
ZDHHC21   --------------H---YEEGHIP------------------------------GI---------------
ZDHHC22   --------------A---AAR-LFS-----------------------------P-----------------
ZDHHC23   --------------P---KGR--VG-----------------------------PVO---------------
ZDHHC24   --------------Y---VLV--LG-----------------------------P-G---------------

cons


ZDHHC5    ----VPIYNAI---------MF--LF-------VLA----NFSMATFMDPGI---FPRAE------------
ZDHHC1    ----GYACMGA---------IF--AG-------HLV----VHLTAVSIDPAD---ANVR-------------
ZDHHC2    ----CLMAYHL---------LF--AM-------FVWS----YWKTIFTLPMN---PSKEFHLSYAEKDLLER
ZDHHC3    ----NGIVFNL---------LA--FL-------ALAS----HCRAMLTDPGA---VPKGN------------
ZDHHC4    VFGYCOELELS-LHYLLLPYLL-LGV-------NLF----FFTLTCGTNPGI---ITKAN------------
ZDHHC6    ----FIMLIN----------WT--VM-------ILYN----YFNAMFVGPGF---VPLGW------------
ZDHHC7    ----NGVIFNC---------LA--VL-------ALSS----HLRTMLTDPGA---VPKGN------------
ZDHHC8    ----VPVYNGI---------IF--LF-------VLA----NFSMATFMDPGV---FPRAD------------
ZDHHC9    ----IPVFAAM---------LF--LF-------SMA----TLLRTSFSDPGV---IPRAL------------
ZDHHC10   ----IPVFAAM---------LF--LF-------SMA----TLLRTSFSDPGV---IPRAL------------
ZDHHC11   ----AYVVTGG---------IF--SF-------HLV----VHLIASCIDPAD---SNVRL------------
ZDHHC12   ----LPLTFLL---------LV--LG-------SLLL----YLAVSLMDPGY---VNVOP------------
ZDHHC13   ILF-FPDLAGAPFY---FSFIFSIVA-------FLY----FFYKTWATDPGF---TKASE------------
ZDHHC14   ----IPAVAGI---------LF--FF-------VMG----TLLRTSFSDPGV---LPRAT------------
ZDHHC15   ----YLILYHA---------IF--VF-------FTWT----YWKSIFTLPQQ---PNQKFHLSYTDKERYEN
```

```
ZDHHC16   ----VPRLCWHF-FYSHW--NL--IL-------IVF----HYYOAITTPPGY---PPOG-------------
ZDHHC17   FWF-WND---LNFLFIHLPFLANSVA-------LFY----NFGKSWKSDPGI---IKATE------------
ZDHHC18   ----IPIIAAI---------LF--FF-------VMS----CLLOTSFTDPGI---LPRAT------------
ZDHHC19   ----FPVITGS---------LF--VL-------TFF----SLVSLNFSDPGI---LHOGS------------
ZDHHC20   ----YLVAFHL---------FF--VM-------FVWS----YWMTIFTSPAS---PSKEFYLSNSEKERYEK
ZDHHC21   ----LIIIFYG---------IS--IF-------CLVA----LVRASITDPGR---LPENP------------
ZDHHC22   -----ALLHGA---------LF--LF-------LSANALGNYVLVIONSPD---------------------
ZDHHC23   ----LAVLTCG---------LF--LI-------LLALH------RAKKNPGY---LSNPA------------
ZDHHC24   ----PPPL--GP-LARAL--QL--ALAAFQLLNLLG----NVGLFLRSDPSIRGVMLAG-------------

cons                                                       *


ZDHHC5    --EDEDKED------------------------------DFR-A-PLYKTV--EIKGIOVRMKWCATCRFY-
ZDHHC1    ------------------------------------DK-SYAGP-LPIFNR--SOHAHVIEDLHCNLCNVD-
ZDHHC2    EPRGEAH-----------------------------QE-VLRRAA-KDLPI--YTRTMSGAIRYCDRCOLI-
ZDHHC3    --ATK---------------------------------------E-FIESL--QLK-PGOVVYKCPKCCSI-
ZDHHC4    --ELLF---------------------------------------LHVYEF---DEVMFPKNVRCSTCDLR-
ZDHHC6    --KP-------------------------------------------------EISQDTMYLOYCKVCOAY-
ZDHHC7    --ATK---------------------------------------E-YMESL--OLK-PGEVIYKCPKCCCI-
ZDHHC8    --EDEDKED------------------------------DFR-A-PLYKNV--DVRGIOVRMKWCATCHFY-
ZDHHC9    --PDEAAFIEMEIEATN----------------GAVPO-GORPP-PRIKNF--OINNOIVKLKYCYTCKIF-
ZDHHC10   --PDEAAFIEMEIEATN----------------GAVPO-GORPP-PRIKNF--OINNOIVKLKYCYTCKIF-
ZDHHC11   ------------------------------------MK-NYSQP-MPLFDR--SKHAHVIONOFCHLCKVT-
ZDHHC12   --OPOE--------------------------------------ELKEEOT--AMVPPAIPLRRCRYCLVL-
ZDHHC13   --EEKK---------------------------------------VNIITL--AETGSLDFRTFCTSCLIR-
ZDHHC14   --PDEAADLERQIDIAN----------------GTSSG-GYRPP-PRTKEV--IINGOTVKLKYCFTCKIF-
ZDHHC15   EERPEVQ-----------------------------KQ-MLVDMA-KKLPV--YTRTGSGAVRFCDRCHLI-
ZDHHC16   -------------------------------------------------------RNDIATVSICKKCIYP-
ZDHHC17   --EOKK---------------------------------------KTIVEL--AETGSLDLSIFCSTCLIR-
ZDHHC18   --VCEAAALEKQIDN-------------------TGSS-TYRPP-PRTREV--LINGOMVKLKYCFTCKMF-
ZDHHC19   --AEOG-------------------------------------P-LTVHVV--WVNHGAFRLOWCPKCCFH-
ZDHHC20   EFSOERQ-----------------------------QE-ILRRAA-RALPI--YTTSASKTIRYCEKCOLI-
ZDHHC21   --KIP---------------------------------------H-G----------EREFWELCNKCNLM-
ZDHHC22   ------------------------------------------------------------DLGACOGASARK
ZDHHC23   --SGDRSLSSSQLECLSRKGQEKTKGFPGADMSGSLNNRTTKDD-PKGSSKMPAGSPTKAKEDWCAKCOLV-
ZDHHC24   -------------------------------------------------------RGLGQGWAYCYQCQSQ-

cons                                                                      *  .


ZDHHC5    ---RPPRCSHCSVCDNCVEEFDHHCPWVNNCIGRRNYRYFFLFLLSLTAHIMGVFGF-GL---LYV-L----
ZDHHC1    ---VSARSKHCSACNKCVCGFDHHCKWLNNCVGERNYRLFLHSVASALLGVLLLVLV--A---TYV-F----
ZDHHC2    ---KPDRCHHCSVCDKCILKMDHHCPWVNNCVGFSNYKFFLLFLAYSLLYCLFIAAT-DL---OYF-I----
ZDHHC3    ---KPDRAHHCSVCKRCIRKMDHHCPWVNNCVGENNOKYFVLFTMYIALISLHALIM-VGF--HFLH-----
ZDHHC4    ---KPARSKHCSVCNWCVHRFDHHCVWVNNCIGAWNIRYFLIYVLTLTASAATVAIV-ST---TFL-VHLVV
ZDHHC6    ---KAPRSHHCRKCNRCVMKMDHHCPWINNCCGYONHASFTLFLLLAPLGCIHAAFIFVMT--MYTOL----
ZDHHC7    ---KPERAHHCSICKRCIRKMDHHCPWVNNCVGEKNORFFVLFTMYIALSSVHALIL-CGF--OFIS-----
ZDHHC8    ---RPPRCSHCSVCDNCVEDFDHHCPWVNNCIGRRNYRYFFLFLLSLSAHMVGVVAF-GL---VYV-L----
ZDHHC9    ---RPPRASHCSICDNCVERFDHHCPWVGNCVGKRNYRYFYLFILSLSLLTIYVFAF-NI---VYV-A----
ZDHHC10   ---RPPRASHCSICDNCVERFDHHCPWVGNCVGKRNYRYFYLFILSLSLLTIYVFAF-NI---VYV-A----
ZDHHC11   ---VNKKTKHCISCNKCVSGFDHHCKWINNCVGSRNYWFFFSTVASATAGMLCLIAI--L---LYV-L----
ZDHHC12   ---OPLRARHCRECRRCVRRYDHHCPWMENCVGERNHPLFVVYLALOLVVLLWGLYL-AWSGLRFF------
ZDHHC13   ---KPLRSLHCHVCNCCVARYDOHCLWTGRCIGFGNHHYYIFFLFFLSMVCGWIIYG-SF---IYL-S----
ZDHHC14   ---RPPRASHCSLCDNCVERFDHHCPWVGNCVGKRNYRFFYMFILSLSFLTVFIFAF-VI---THV-I----
ZDHHC15   ---KPDRCHHCSVCAMCVLKMDHHCPWVNNCIGFSNYKFFLOFLAYSVLYCLYIATT-VF---SYF-I----
ZDHHC16   ---KPARTHHCSICNRCVLKMDHHCPWLNNCVGHYNHRYFFSFCFFMTLGCVYCSYG-SW---DLF-R----
ZDHHC17   ---KPVRSKHCGVCNRCIAKFDHHCPWVGNCVGAGNHRYFMGYLFFLLFMICWMIYG-CI---SYW-G----
ZDHHC18   ---RPPRTSHCSVCDNCVERFDHHCPWVGNCVGRRNYRFFYAFILSLSFLTAFIFAC-VV---THL-T----
ZDHHC19   ---RPPRTYHCPWCNICVEDFDHHCKWVNNCIGHRNFRFFMLLVLSLCLYSGAMLVT-CL---IFL-V----
ZDHHC20   ---KPDRAHHCSACDSCILKMDHHCPWVNNCVGFSNYKFFLLFLLYSLLYCLFVAAT-VL---EYF-I----
ZDHHC21   ---RPKRSHHCSRCGHCVRRMDHHCPWINNCVGEDNHWLFLOLCFYTELLTCYALMF-SFC--HYYYF----
ZDHHC22   TPCPSPSTHFCRVCARVTLRHDHHCFFTGNCIGSRNMRNFVLFCLYTSLACLYSMVA-GV---AYI-S----
ZDHHC23   ---RPARAWHCRICGICVRRMDHHCVWINSCVGESNHOAFILALLIFLLTSVYGITL-TL---DTI-C----
ZDHHC24   ---VPPRSGHCSACRVCILRRDHHCRLLGRCVGFGNYRPFLCLLLHAAGVLLHVSVL-LG---PAL-S----

cons               .*  *       *:**     * *  *   :


ZDHHC5    --YHI---------------E---ELS------------GVRT-AV-----------------TM----AV-
ZDHHC1    --VEFFV-NPM-RLRTNRHFE---VLK-NHTDVWFVFLPAAPV-ET------QAP---AILA-LA----ALL
ZDHHC2    --KF--------------WTN---GLP-D--------TO----AKF-----------------HI----MF-
ZDHHC3    ---C-FE---------EDWTKCS-SFS-P--------PT----TVI-----------------LL----IL-
ZDHHC4    MSDLYOE--TY--IDDLGHL----HVMDT--------VFLIOY-LF-----------------LTFPRIVF-
ZDHHC6    --YHRLS---------FGWNTVKIDMS-A--------ARRDPLPIV-----------------PF----GL-
ZDHHC7    ---C-VR---------GQWTECS-DFS-P--------PI----TVI-----------------LL----IF-
ZDHHC8    --NHA---------------E---GLG------------AAHT-TI-----------------TM----AV-
ZDHHC9    --LKSLK---------IGFLE---TLK------------ETPG-TV-----------------LE----VL-
ZDHHC10   --LKSLK---------IGFLE---TLK------------ETPG-TV-----------------LE----VL-
ZDHHC11   --VOYLV-NPG-VLRTDPRYE---DVK-NM-NTWLLFLPLFPV-OV------QTL---IVVI-IG----MLV
ZDHHC12   --OP--------------WGO---WLR-S--------S-----GLL-----------------FA----TF-
ZDHHC13   --SHCAT--TF--KE-DGLWT---YLNQI--------VACSPW-VL-----------------YI----LM-
ZDHHC14   --LRSQQ---------TGFLN---ALK------------DSPA-SV-----------------LE----AV-
ZDHHC15   --KY--------------WRG---ELP-S--------VR----SKF-----------------HV----LF-
ZDHHC16   --EAYAAIEKMKQLD-KNKLO---AVA-N------QTYHOTPP-PTFSF--RERM---THKS-LV----YL-
ZDHHC17   --LHCET--TY--TK-DGFWT---YITQI--------ATCSPW-MF-----------------WM----FL-
ZDHHC18   --LRAQG---------SNFLS---TLK------------ETPA-SV-----------------LE----LV-
ZDHHC19   --RTT-------------------HLP------------FSTD-KA-----------------IA----IV-
ZDHHC20   --KF--------------WTN---ELT-D--------TR----AKF-----------------HV----LF-
ZDHHC21   --LP-LK---------KRNLDLF-VFR-H--------EL----A-------------------IM----RL-
ZDHHC22   --AVL-S---------ISFAH---PLA---------FLTLLPT-SI------SOFFSGAVLGSEM----FV-
ZDHHC23   --RD--R---------SVF-------T---------ALFYCPG-VY------ANY---------S----SA-
ZDHHC24   --AL---LRAHTPLH-MAA------------------LLLLPW-LML-LTGRVSL---AQFA-LA----FV-

cons
```

```
ZDHHC5     --MCV--------AG-LFF-I----PVAGLTGFHVVL-VARGRTTNEOVTGKFRG-----------------
ZDHHC1     ILLGL--------LS-T---A----LLGHLLCFHIYL-MWHKLTTYEYIVOHRPPOEAK--------G---V
ZDHHC2     --LFF--------AAAMFS-V----SLSSLFGYHCWL-VSKNKSTLEAFRSPVFRH----------------
ZDHHC3     --LCF--------EGLLFL-I----FTSVMFGTOVHS-ICTDETGIEOLKKEERRW------------A---
ZDHHC4     -MLGF--------VV-VLS-F----LLGGYLLFVLYL-AATNOTTNEWYRGDWAWCORCPLVAWPPSA-E-P
ZDHHC6     --AAFATTLFALGLALGTT-I----AVGMLFFIOMKI-ILRNKTSIESWIEEKAKDRIQ--------YYQLD
ZDHHC7     --LCL--------EGLLFF-T----FTAVMFGTOIHS-ICNDETEIERLKSEKPTW------------E---
ZDHHC8     --MCV--------AG-LFF-I----PVIGLTGFHVVL-VTRGRTTNEOVTGKFRG-----------------
ZDHHC9     --ICF--------FT-L---W----SVVGLTGFHTFL-VALNOTTNEDIKGSWTGKNR--------------
ZDHHC10    --ICF--------FT-L---W----SVVGLTGFHTFL-VALNOTTNEDIKGSWTGKNR--------------
ZDHHC11    LLLDF--------LG-L---V----HLGOLLIFHIYL-KAKKMTTFEYLINNRKEESSK--------H---Q
ZDHHC12    ---LL--------L-SLFS-L----VASLLLVSHLYL-VASNTTTWEFISSHRIAY------------LR--
ZDHHC13    -LATF--------HF-----S----WSTFLLLNOLFOIAFLGLTSHERISLOKOSKHM--------KQ-T-L
ZDHHC14    --VCF--------FS-V---W----SIVGLSGFHTYL-ISSNOTTNEDIKGSWSNKRG--------------
ZDHHC15    --LLF--------VACMFF-V----SLVILFGYHCWL-VSRNKTTLEAFCTPVFTS----------------
ZDHHC16    WFLCS--------SV-A---L----ALGALTVWHAVL-ISRGETSIERHINKKERRRLQ--------A-K-G
ZDHHC17    -NSVF--------HF-----M----WVAVLLMCOMYOISCLGITTNERMNARRY-KHF--------KV-T-T
ZDHHC18    --ICF--------FS-I---W----SILGLSGFHTYL-VASNLTTNEDIKGSWSSKRGG-------------
ZDHHC19    --VAV--------SAAGLL-V----PLSLLLLIOALS-VSSADRTYK---GKCRH-LQ--------------
ZDHHC20    --LFF--------VSAMFF-I----SVLSLFSYHCWL-VGKNRTTIESFRAPTFSY----------------
ZDHHC21    --AAF--------MGITML-V----GITGLFYTOLIG-IITDTTSIEKMSNCCEDI------------S---
ZDHHC22    --ILM--------LY-LWFAIGL--ACAGFCCHOLLL-ILRGOTRHOVRKGVAVR-----------------
ZDHHC23    --LSF--------TC-VWYSVIITAGMAYIFLIOLIN-ISYNVTEREVOOAL--------------------
ZDHHC24    TDTCV--------AG-A---L----LCGAGLLFHGML-LLRGQTTWEWARGQ--------------------

cons                                                      :


ZDHHC5     ----GVNPF---TNGC-CNNVS----------------------------------------------RV--
ZDHHC1     HRELESCPP---KMRP-IOEMEFYMRTFRHMRPEPPGQAGPAAVNAKHSRPASPDPTPGRRDCAGPPVOVEW
ZDHHC2     --GTDKNGF---SLGF-SKNMR----------------------------------------------OV--
ZDHHC3     ----KKTK---------WMNMK----------------------------------------------AV--
ZDHHC4     --OVHRNIH---SHGL-RSNLO----------------------------------------------EI--
ZDHHC6     --EVFVFPY---DMGSRWRNFK----------------------------------------------OV--
ZDHHC7     ----RRLR---------WEGMK----------------------------------------------SV--
ZDHHC8     ----GVNPF---TRGC-CGNVE----------------------------------------------HV--
ZDHHC9     ----VONPY---SHGNIVKNCC----------------------------------------------EV--
ZDHHC10    ----VONPY---SHGNIVKNCC----------------------------------------------EV--
ZDHHC11    --AVRKDPYVQMDKGV-LOO--------------GAGALGSSA---------------------------QG
ZDHHC12    --ORPSNPF---DRGL-TRNLA----------------------------------------------HF--
ZDHHC13    --SLRKTPY---NLGF-MONLA----------------------------------------------DF--
ZDHHC14    --KENYNPY---SYGNIFTNCC----------------------------------------------VA--
ZDHHC15    --GPEKNGF---NLGF-IKNIO----------------------------------------------OV--
ZDHHC16    --RVFRNPY---NYGC-LDNWK----------------------------------------------VF--
ZDHHC17    --TSIESPF---NHGC-VRNII----------------------------------------------DF--
ZDHHC18    --EASVNPY---SHKSIITNCC----------------------------------------------AV--
ZDHHC19    ----GYNPF---DOGC-ASNWY----------------------------------------------LT--
ZDHHC20    --GPDGNGF---SLGC-SKNWR----------------------------------------------OV--
ZDHHC21    ---RPRKPW--------OOTFS----------------------------------------------EV--
ZDHHC22    -----ARP-------W-RKNLO----------------------------------------------EV--
ZDHHC23    ---------------------R----------------------------------------------OK--
ZDHHC24    ------HSY---DLGP-CHNLQ----------------------------------------------AA--

cons


ZDHHC5     ----------------L-----CSSP-APRYLGR-----------PKKEKT-IVIRPPFLRPEVSDGO----
ZDHHC1     DRKKPLPWRSPLLLLAM-----WGPOAPPCLCRK---------------------------------R--G-
ZDHHC2     ----------------F-----GDEK--KYWLLPIFSSLGDGCSFP---------------TCLV-------
ZDHHC3     ----------------F-----GHPF-SLGWASPFAT-----------------------------------
ZDHHC4     ----------------FL----------PA------------------------------------------
ZDHHC6     ----------------F-----TWS----------GVPEGDGLEWP---------------VREGCHQ--YS
ZDHHC7     ----------------F-----GGPP-SLLWMNPFVGFR--FRRLP--------------------------
ZDHHC8     ----------------L-----CSPL-APRYVVE-----------PPRLPLAVSLKPPFLRPELLDRA--AP
ZDHHC9     ----------------L-----CGPL-PPSVLDR---------------------------------R--GI
ZDHHC10    ----------------L-----CGPL-PPSVLDR---------------------------------R--GI
ZDHHC11    VKAKS--SLLI-HKHL--------CHFCT-SVNO---------------------------------D--G-
ZDHHC12    ----------------F-----CGWP-SGSWETLWAE-----------------------------------
ZDHHC13    ----------------FQ-CGCFGLV-KPCVVDW---------------------------------TSQYT
ZDHHC14    ----------------L-----CGPI-SPSLIDR---------------------------------R--GY
ZDHHC15    ----------------F-----GDKK--KFWLIPIGSSPGDGHSFP--------------------------
ZDHHC16    ----------------L-----GVDT-GRHWLTR------------------------------------VL
ZDHHC17    ----------------FE-FRCCGLF-RPVIVDW---------------------------------TRQYT
ZDHHC18    ----------------L-----CGPL-PPSLIDR---------------------------------R--GF
ZDHHC19    ----------------I-----CAPL-GPKYMAE---------------------------------A--VQ
ZDHHC20    ----------------F-----GDEK--KYWLLPIFSSLGDGCSFP---------------TRLV-------
ZDHHC21    ----------------F-----GTRW-KILWFIPFRQR------QP--------------------------
ZDHHC22    ----------------FGKRWLLGLL-VPMFNVG--------------------------------------
ZDHHC23    ----------------TGRRLLCGLI-VDTGLLG--------------------------------------
ZDHHC24    ----------------L-----GPRW-ALVWLWP------------------------------------FL

cons


ZDHHC5     ITVKIMDNGIQGELRRTKSKGSLEITE----SQSADAEPPPPPKPDLSRYTGLRTHLGLAT-----------
ZDHHC1     ------------------------------------------------------------------------
ZDHHC2     --------------------------NQDPEQA------STPA----------------GL-----------
ZDHHC3     ------------------------------------------------------------------------
ZDHHC4     ------------------------------------------------------------------------
ZDHHC6     LT------------------------IEQLKQK--------------------------AD-----------
ZDHHC7     ------------------------------------------------------------------------
ZDHHC8     LKVKLSDNGLKAGLGRSKSKGSLDRLD----EKPLDLGPPLPPKIEAGTFSSDLQTPRPGS-----------
ZDHHC9     LPLE----------------------E----SG------SRPP----------------ST-----------
ZDHHC10    LPLE----------------------E----SG------SRPP----------------ST-----------
ZDHHC11    ------------------------------------------------------------------------
ZDHHC12    ------------------------------------------------------------------------
ZDHHC13    MVF---------------------------------------------------------------------
```

```
ZDHHC14   IQPD----------------------T----PQ------PAAP----------------SNGITMYGATQSQ
ZDHHC15   ------------------------------------------------------------------------
ZDHHC16   LP------------------------S---------------------------------------------
ZDHHC17   IEY---------------------------------------------------------------------
ZDHHC18   VQSD----------------------T----VL------P--------------------------------
ZDHHC19   LQ----------------------------------------------------------------------
ZDHHC20   --------------------------GMDPEQA------SVTN----------------QN-----------
ZDHHC21   ------------------------------------------------------------------------
ZDHHC22   ------------------------------------------------------------------------
ZDHHC23   ------------------------------------------------------------------------
ZDHHC24   --------------------------A---------------------------------------------

cons


ZDHHC5    -----------------------------N-----E------DSSL-------LAKDSPPTPTMYKYRPGYS
ZDHHC1    ------------------------------------------------------------------------
ZDHHC2    -------NSTAK----NLENHQFPA------------KPLRESQS-------HLLTDSQ-SWT---------
ZDHHC3    --------------------------------------PDQGKA----------------------------
ZDHHC4    ---------------------------------------------------------FP-------------
ZDHHC6    -------KRVRSVRYKVIEDYSGACCPLNKGIKTFFTSPCTE-EPRIQLQKGEFILATR-GLRY--------
ZDHHC7    ------------------------T------------RPRKG------------------------------
ZDHHC8    -----------------------------A-----E------SALS-------VQRTSPPTPAMYKFRPAFP
ZDHHC9    -----------------------------Q-----E------TSSS-------LLPQSP-APTE--------
ZDHHC10   -----------------------------Q-----E------TSSS-------LLPQSP-APTE--------
ZDHHC11   ------------------------------------------------------------------------
ZDHHC12   --------------------------------------EEEE------------------------------
ZDHHC13   ---------------------------------------------------------HP-------------
ZDHHC14   SDMCDQDQCIQSTKFVL------------Q-----A------AATP-------LLQSEP-SLTS--------
ZDHHC15   ------------------------M------------RSMNESQN-------PLLANEE-TWE---------
ZDHHC16   ---------------------------------------------------------SH-L-----------
ZDHHC17   ---------------------------------------------------------DQ-------------
ZDHHC18   --------------------------------------------SP-------IRSDEP-ACR---------
ZDHHC19   ------------------------------------------------------------------------
ZDHHC20   -------EYARS----SGSNQPFPI------------KPLSESKN-------RLLDSES-QWL---------
ZDHHC21   ------------------------L------------------R----------------------------
ZDHHC22   ------------------------------------------------------------------------
ZDHHC23   ------------------------------------------------------------------------
ZDHHC24   ---------------------------------------------------------SP-L-----------

cons


ZDHHC5    SSSTSAA---MPHSS--------SAKLSRGDSLKEPTSIAESSRHP---SYRSEPSLEPES-----------
ZDHHC1    ------------------------------------------------------------------------
ZDHHC2    ---------------------------------------------ESSIN----------------------
ZDHHC3    ------------------------------------------------------------------------
ZDHHC4    ------------------------------------------------------------------------
ZDHHC6    --------------------------------------WLYGDKIL---DDS--------------------
ZDHHC7    ------------------------------------------------------------------------
ZDHHC8    TGP---K---VPFCGPGEQVPGPDSLTLGDDSIRSL-------------DFVSEPSLDLPDYGPGGLHAAYP
ZDHHC9    --------------------------------------HLNSNEMP---EDSST---------PE-------
ZDHHC10   --------------------------------------HLNSNEMP---EDSST---------PE-------
ZDHHC11   -------------------------------------------------D----------------------
ZDHHC12   ------------------------------------------------------------------------
ZDHHC13   ------------------------------------------------------------------------
ZDHHC14   ------DELHLPGK------------------------PGL--GTP---CAS------------LT------
ZDHHC15   ---------------------------------------------DNEDD----------------------
ZDHHC16   ---------------------------------------------P---HGN--------------------
ZDHHC17   ------------------------------------------------------------------------
ZDHHC18   -------------------------------------------AKP---DAS--------------------
ZDHHC19   -----------------------------------------RVVGP---DWTSMPNLHPPMS-PS-------
ZDHHC20   ---------------------------------------------ENGAE----------------------
ZDHHC21   ------------------------------------------------------------------------
ZDHHC22   ---------------------------------------------------S--------------------
ZDHHC23   ------------------------------------------------------------------------
ZDHHC24   ---------------------------------------------P----GD--------------------

cons


ZDHHC5    ------FRSPTFGKSFHFDPLSSGSRSSSLKSAQGTGFELGQLQSIRSEGTTSTSYKSL----ANQTRNGSL
ZDHHC1    ------------------------------------------------------------------------
ZDHHC2    ------------------------------------------------------------------------
ZDHHC3    ------------------------------------------------------------------------
ZDHHC4    ------------------------------------------------------------------------
ZDHHC6    ------------------------------------------------------------------------
ZDHHC7    ------------------------------------------------------------------------
ZDHHC8    PSPPLSA----------SDAFSGALRSLSLKASSRRGGDHVALQPLRSEGGPPTPHRSIFAPHALPNRNGSL
ZDHHC9    ------------------------------------------------------------------------
ZDHHC10   ------------------------------------------------------------------------
ZDHHC11   ------------------------------------------------------------------------
ZDHHC12   ------------------------------------------------------------------------
ZDHHC13   ------------------------------------------------------------------------
ZDHHC14   ------------------------------------------------------------------------
ZDHHC15   ------------------------------------------------------------------------
ZDHHC16   ------------------------------------------------------------------------
ZDHHC17   ------------------------------------------------------------------------
ZDHHC18   ------------------------------------------------------------------------
ZDHHC19   ------------------------------------------------------------------------
ZDHHC20   ------------------------------------------------------------------------
ZDHHC21   ------------------------------------------------------------------------
ZDHHC22   ------------------------------------------------------------------------
ZDHHC23   ------------------------------------------------------------------------
ZDHHC24   ------------------------------------------------------------------------

cons
```

```
ZDHHC5     SYDSLLTPSDSPDFESVQAGPEPDPPLGYTSPFLSARLAQQREAERHPRLVPT-GPTHREPSPVRYDNLSRH
ZDHHC1     ---------------------------------RGACIK------------------------CER------
ZDHHC2     ------------------------------------------------------------------------
ZDHHC3     ------------------------------------------------------------------------
ZDHHC4     ------------------------------------------------------------------------
ZDHHC6     ------------------------------------------------------------------------
ZDHHC7     ------------------------------------------------------------------------
ZDHHC8     SYDSLLNPG-SPGGHACPAHPAVG-VAGYHSPYLHPGATGDPPRPLPRSFSPVLGPRPREPSPVRYDNLSRT
ZDHHC9     ------------------------------------------------------------------------
ZDHHC10    ------------------------------------------------------------------------
ZDHHC11    --------------STAREGDEDPCPSALGAKARNSRLI------------------------CRRL-----
ZDHHC12    ------------------------------------------------------------------------
ZDHHC13    ------------------------------------------------------------------------
ZDHHC14    ----------------------LG-----------------PPTP-----------------PASMPNLAEA
ZDHHC15    ------------------------------------------------------------------------
ZDHHC16    ------------------------------------------------------------------------
ZDHHC17    ------------------------------------------------------------------------
ZDHHC18    ------------------------------------------------------------------------
ZDHHC19    ------------------------------------------------------------------------
ZDHHC20    ------------------------------------------------------------------------
ZDHHC21    ------------------------------------------------------------------------
ZDHHC22    ------------------------------------------------------------------------
ZDHHC23    ------------------------------------------------------------------------
ZDHHC24    ------------------------------------------------------------------------

cons


ZDHHC5     IVASLQ-ERE------------------KLLRQSPPLPGREEE-PGLGDSGIQSTPG---------------
ZDHHC1     -----LRPR---IRRRGLGPPAAAPARRR-------------------------IPR---------------
ZDHHC2     -------------------------------------------------------PGK--------------
ZDHHC3     ------------------------------------------------------------------------
ZDHHC4     ------------------------------------------------------------------------
ZDHHC6     --------------------------------------------------FI---EGV--------------
ZDHHC7     ------------------------------------------------------------------------
ZDHHC8     IMASIQ-ERKDREER------------ERLLRSQ--------ADSLFGDSGVYDAPSSYSLQQASVLSEGPR
ZDHHC9     ------------------------------------------------------------------------
ZDHHC10    ------------------------------------------------------------------------
ZDHHC11    --CQFS---------TRVHPDGGSMAQEA--D---------------------DAPS---------------
ZDHHC12    ------------------------------------------------------------------------
ZDHHC13    ------------------------------------------------------------------------
ZDHHC14    TLADVM-PRKDEHMG------------HQ-------------------------------------------
ZDHHC15    -------------------------------------------------------NQD--------------
ZDHHC16    ---------------------GMSW-----------------------------------------------
ZDHHC17    ------------------------------------------------------------------------
ZDHHC18    ------------------------------------------------------------------------
ZDHHC19    ------------------------------------------------------------------------
ZDHHC20    -------------------------------------------------------EGI--------------
ZDHHC21    ------------------------------------------------------------------------
ZDHHC22    ------------------------------------------------------------------------
ZDHHC23    ------------------------------------------------------------------------
ZDHHC24    ---------------------GITF-----------------------------------------------

cons


ZDHHC5     -----------------------------------------SGHAPRTSSSSDDSKRS-----PL-----G-
ZDHHC1     ----------------------TPA------LCTPLALPAPTTRRRQSPWTRFQWRRRAWAAPLW-------
ZDHHC2     ---------------------------------C-----K------------------------A-------
ZDHHC3     ------------------------------------------------------------------------
ZDHHC4     ---------------------------------------------------------------CH-------
ZDHHC6     ---------------------------------S-----RIRGWFPRKCVEKC----------PC-------
ZDHHC7     ------------------------------------------------------------------------
ZDHHC8     GPALRYGSRDDLVAGPGFGGARNPALQTSLSS-----LSSSVSRAPRTSSSSLQADQASSNA-PGPRPSSGS
ZDHHC9     -------------------------------E-----MPPPE-------------------P-PE-------
ZDHHC10    -------------------------------E-----MPPPE-------------------P-PE-------
ZDHHC11    ----------------------I----------STLGLQQET------------------TEPMK-------
ZDHHC12    ------------------------------------------------------------------------
ZDHHC13    ---------------------------------------------------------------AR-------
ZDHHC14    -------------------------------F-----LTPDE-------------------A-PS-------
ZDHHC15    ---------------------------------Y-----P------------------------E-------
ZDHHC16    -----------------------------------------E-------------------P-P--------
ZDHHC17    ---------------------------------------------------------------IS-------
ZDHHC18    ------------------------------------------------------------------------
ZDHHC19    -------------------------------A-----LNPPA-------------------P-TS-------
ZDHHC20    ---------------------------------V-----K------------------------S-------
ZDHHC21    ------------------------------------------------------------------------
ZDHHC22    ------------------------------------------------------------------------
ZDHHC23    ------------------------------------------------------------------------
ZDHHC24    -----------------------------------------Q-------------------T-T--------

cons


ZDHHC5     -KT--------PLGRPAVPRFGKPDGLRGRGVGSPEP---GPTAPYL--GRSMSYSSQKAQPGVSETEEVAL
ZDHHC1     ------------------------------------P-------PRG--AG-------------ADSPR---
ZDHHC2     ------------------------------------GMS-N---PAL--T--------------MENE----
ZDHHC3     -------------------------------------DPY-----QY--V--------------V-------
ZDHHC4     ----------------------------------------------E--R--------------K-------
ZDHHC6     ------------------------------------DAE-T----DQ--A--------------PEGEK---
ZDHHC7     --------------------------------------GP-----EF--S--------------V-------
ZDHHC8     HRSPARQGLPSPPGTPHSPSYAGPKAVAFIHTDLPEP---PPS---L--T--------------VQRDH---
ZDHHC9     ------------------------------------P-------PQE--A--------------AEAEK---
ZDHHC10    ------------------------------------P-------PQE--A--------------AEAEK---
ZDHHC11    ---------------------------------------------T---DS-------------AESED---
```

```
ZDHHC12   -------------------------------------GS------SP--A--------------V-------
ZDHHC13   ----------------------------------------------E--K--------------V-------
ZDHHC14   ------------------------------------P-------PRL--L--------------AAGSP---
ZDHHC15   ------------------------------------GSS------SL--A--------------VETE----
ZDHHC16   --------------------------------------------PWV--T--------------AHSAS---
ZDHHC17   ----------------------------------------------G--S--------------G-------
ZDHHC18   ------------------------------------------------------------------------
ZDHHC19   ------------------------------------G-------SLO--S--------------REGTP---
ZDHHC20   ------------------------------------GTNNH---VTV--A--------------IE------
ZDHHC21   -------------------------------------VPY-----HFANH--------------V-------
ZDHHC22   ---------------------------------------------ES--S--------------KQQD----
ZDHHC23   ------------------------------------------------------------------------
ZDHHC24   --------------------------------------------AD---V--------------GHTAS---

cons


ZDHHC5    QPLLTPKDEVQLKTTYSKSNGQPKSLGSASPGPGQP-PLSSPTR-GGVKK--V-----------SGVGGTTY
ZDHHC1    -------------------------------------------WR--G-----------------RR--VRP
ZDHHC2    ------------------------------------------------------------------------
ZDHHC3    ------------------------------------------------------------------------
ZDHHC4    ------------------------------------------------------------------------
ZDHHC6    ------------------------------------------------K-----------------------
ZDHHC7    ------------------------------------------------------------------------
ZDHHC8    ---------PQLKTPPSKLNGQSPGLARLGPATGPPGPSASPTRHTLVKK--V-----------SGVGGTTY
ZDHHC9    ------------------------------------------------------------------------
ZDHHC10   ------------------------------------------------------------------------
ZDHHC11   ------------------------------------------------------------------------
ZDHHC12   ------------------------------------------------------------------------
ZDHHC13   ------------------------------------------------------------------------
ZDHHC14   -------------------------------L-----------AHS--RTMHVLGLASQDSLHEDSVRGLVK
ZDHHC15   ------------------------------------------------------------------------
ZDHHC16   ------------------------------------------------------------------------
ZDHHC17   ------------------------------------------------------------------------
ZDHHC18   -----------------------------------------------------------------MVGGH--
ZDHHC19   --------------------------------------------------------------------GAW-
ZDHHC20   ------------------------------------------------------------------------
ZDHHC21   ------------------------------------------------------------------------
ZDHHC22   ------------------------------------------------------------------------
ZDHHC23   ------------------------------------------------------------------------
ZDHHC24   ------------------------------------------------------------------------

cons


ZDHHC5    EISV
ZDHHC1    PFS-
ZDHHC2    ---T
ZDHHC3    ----
ZDHHC4    KQ-E
ZDHHC6    N--R
ZDHHC7    ----
ZDHHC8    EISV
ZDHHC9    ----
ZDHHC10   ----
ZDHHC11   ----
ZDHHC12   ----
ZDHHC13   LRSV
ZDHHC14   LSSV
ZDHHC15   ---T
ZDHHC16   VMAV
ZDHHC17   YQLV
ZDHHC18   ---P
ZDHHC19   ----
ZDHHC20   ---N
ZDHHC21   ----
ZDHHC22   ---K
ZDHHC23   ----
ZDHHC24   ----

cons
```

Citation

Please cite this result referring the papers at this link.

Result files

9 output files - download them all

Input(s)	Input sequences (9KB)
System	Command line (216 B) Log file (246KB)
Tree	dnd file (654 B)
Multiple Alignment	score_html file (265KB) clustalw_aln file (51KB) fasta_aln file (41KB) score_ascii file (49KB) phylip file (50KB)

Copy to your Dropbox

Send results

Forward this result to other online tools.

Core/TCS	Evaluates your Alignment indicating the local reliability
ProtoGene	Turning amino acid alignments into bona fide CDS nucleotide alignments
MSA hub	MyHits: a new interactive resource for protein annotation and domain identification
JalView	Open this alignment in the Jalview viewer
ESPript	ESPript server renders sequence similarities and secondary structure information from aligned sequences

Info

Some information about this alignment job

Mode: T-Coffee
Request ID: 909d452c
Created at: 09 Dec 2021, 05:12 (CET)
Elapsed time: 1 min 18 sec
Expiration at: 19 Dec

Command Line

This is the command line used to execute your alignment. You can use it as reference to run this alignment on your desktop.

```
t_coffee -in=ZDHHC1-24.fasta -mode=regular -output=score_html clustalw_aln fasta_aln score_ascii phylip -
maxnseq=150 -maxlen=10000 -case=upper -seqnos=off -outorder=input -run_name=result -multi_core=4 -quiet=stdout
```

Replay

Change some input parameters and resubmit this alignment clicking here.

Are you a T-Coffee guru? You may want to use the full featured T-Coffee command line options.

Feedback

Give us feedback about T-coffee web server

Are you satisfied with this result? Do you like this alignment server? If so recommend it using Google+1 or Facebook.

For suggestions, questions or any problem send an email to tcoffee@googlegroups.com

T-Coffee Server is hosted by the **Centre for Genomic Regulation** (CRG) of Barcelona - Powered by nextflow
