# Supplementary material for: Repositioning Lomitapide to block ZDHHC5-dependant palmitoylation on SSTR5 leads to anti-proliferation effect in preclinical pancreatic cancer models
Source: Cell Death Discov. 2023 Feb 11;9:60. doi: 10.1038/s41420-023-01359-4 (PMC9922277; doi:10.1038/s41420-023-01359-4)
Supplement: Supplementary file 7 — Additional Tables [file 41420_2023_1359_MOESM7_ESM.docx]

**Table S1. Characteristics of human pancreatic cancer patients (n = 2)**

| Sample No. | Cancer Type | Gender | Age | T | N | M | Pathological Grade |
| --- | --- | --- | --- | --- | --- | --- | --- |
| 1 | Pancreatic cancer | Female | 54 | T2 | N2 | M0 | Ⅲ |
| 2 | Pancreatic cancer | Male | 65 | T2 | N1 | M1 | Ⅲ |

Both pancreatic cancer tissue samples were collected from Jiangsu Cancer Hospital.
