# Supplementary material for: Repositioning Lomitapide to block ZDHHC5-dependant palmitoylation on SSTR5 leads to anti-proliferation effect in preclinical pancreatic cancer models
Source: Cell Death Discov. 2023 Feb 11;9:60. doi: 10.1038/s41420-023-01359-4 (PMC9922277; doi:10.1038/s41420-023-01359-4)
Supplement: Supplementary file 8 — Original Data File [file 41420_2023_1359_MOESM8_ESM.pdf]

Miapaca-2

MEK
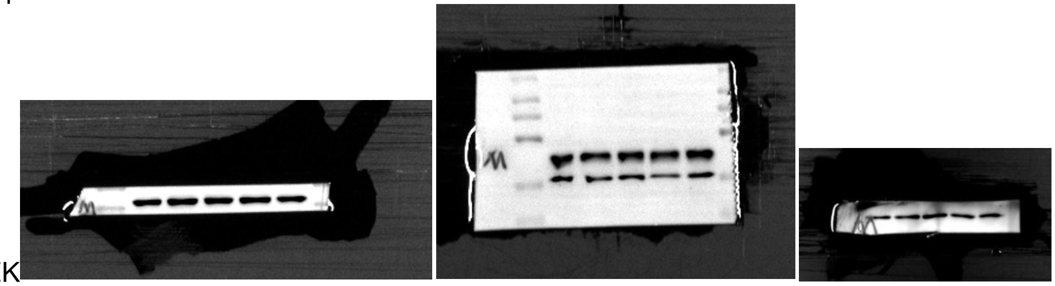

PERK
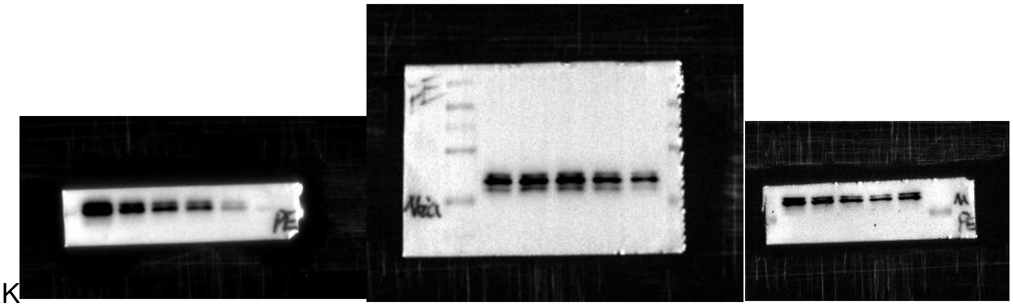

PMEK
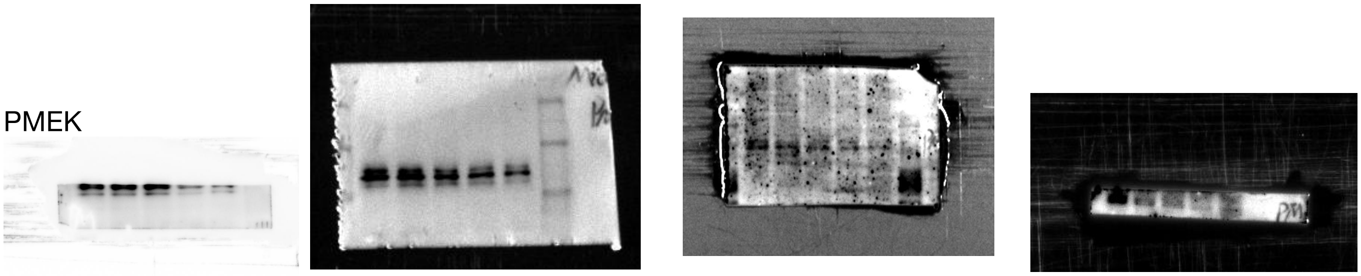

ERK
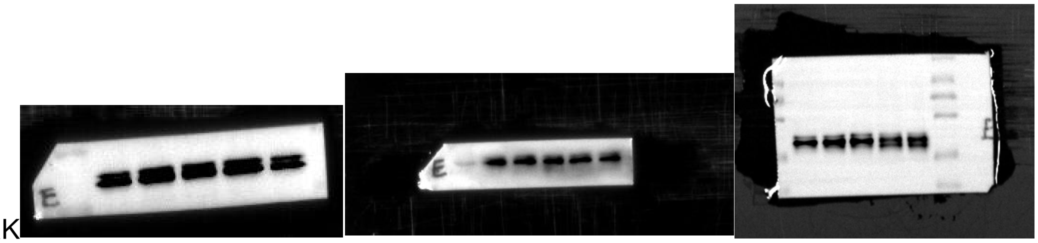

GAPDH
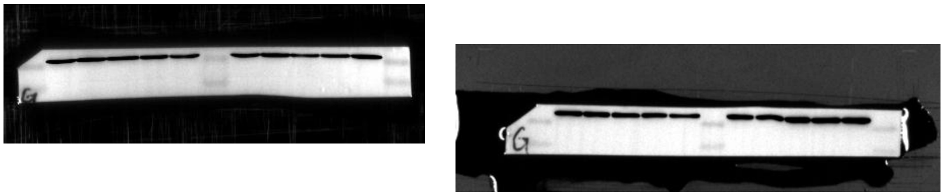

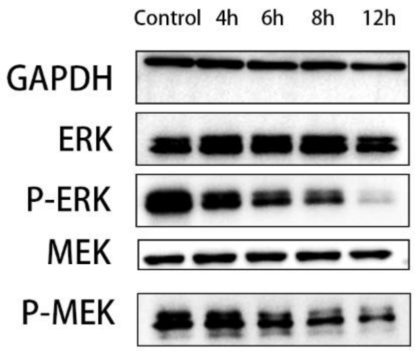

Panc-1

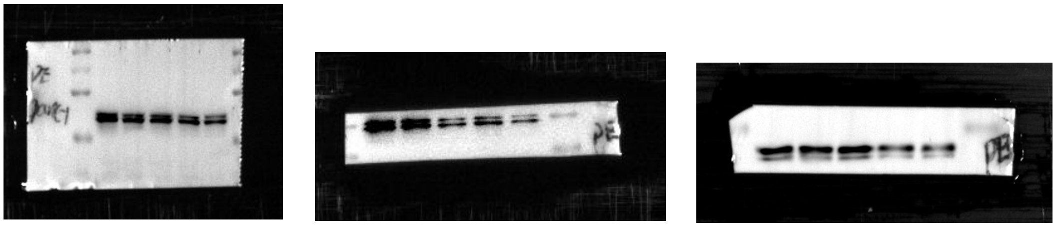

PERK

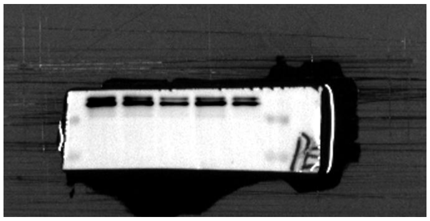

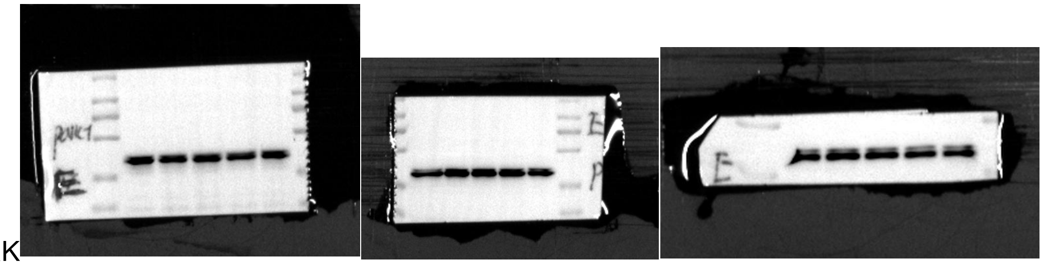

ERK

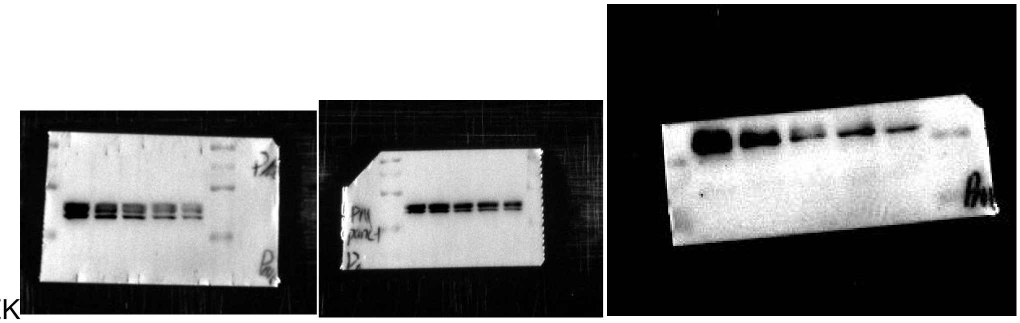

PMEK

MEK

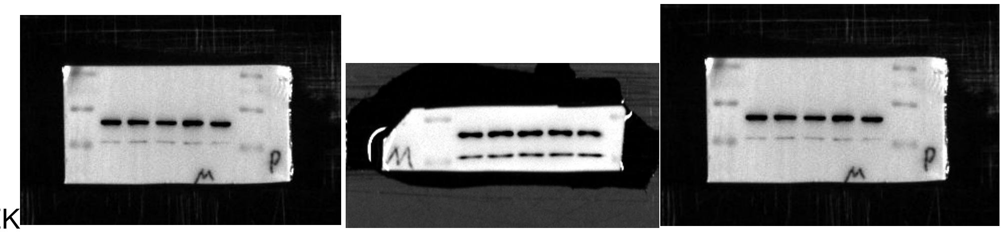

GAPDH

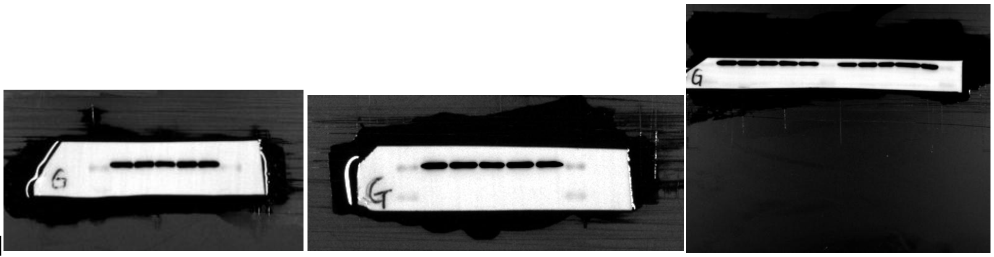

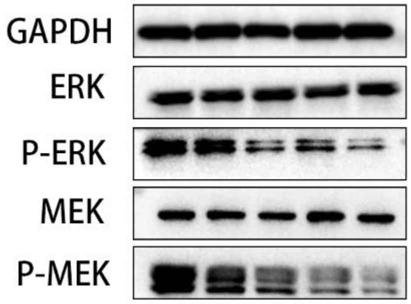

动物 WB

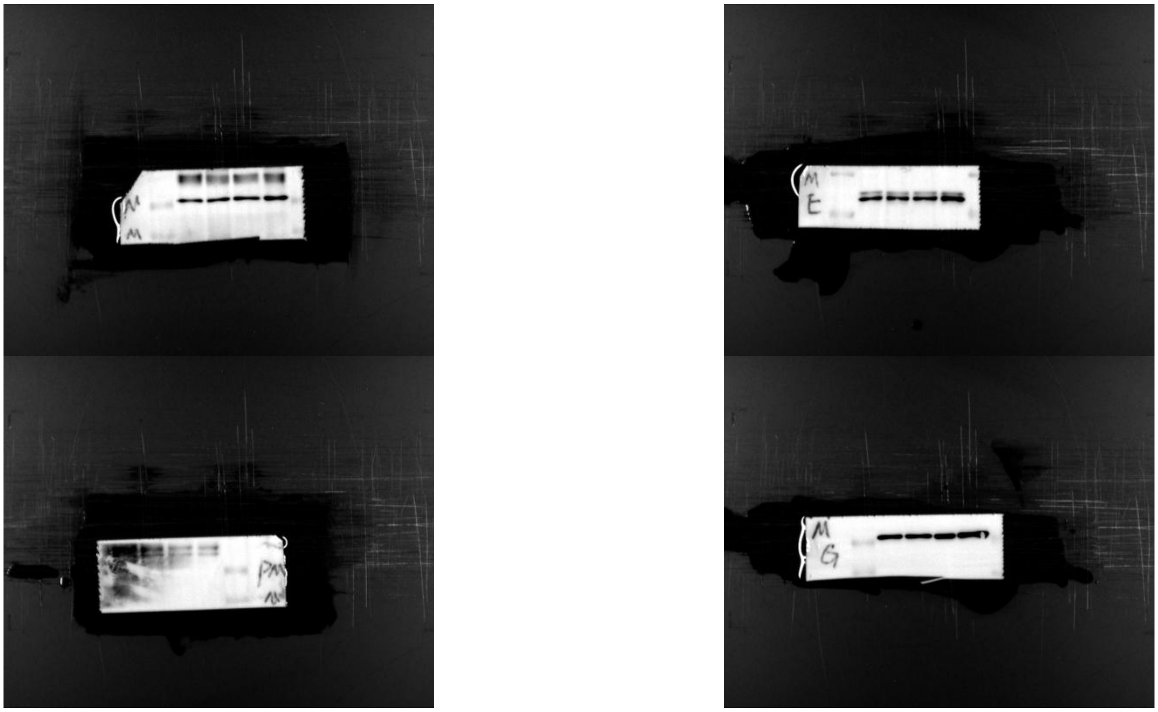

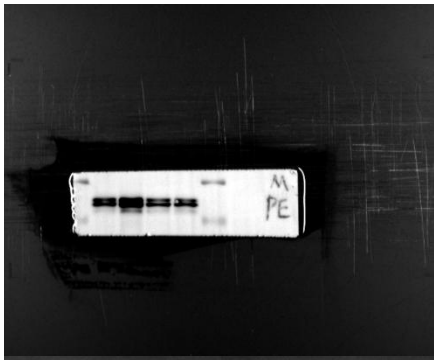
M
PE
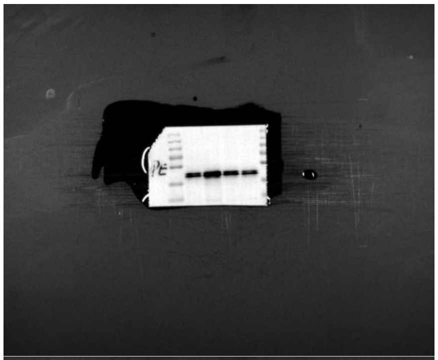
PE
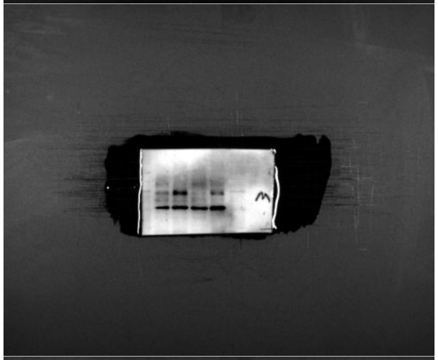
m
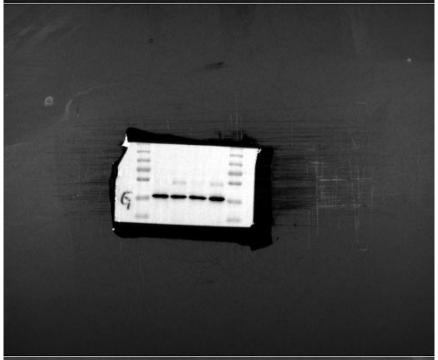
G
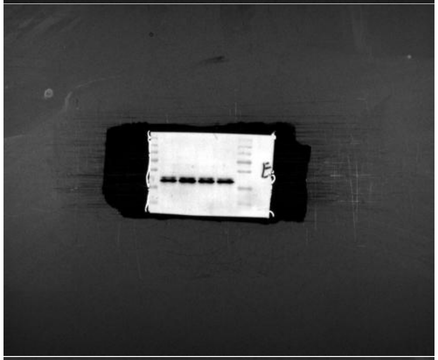
E
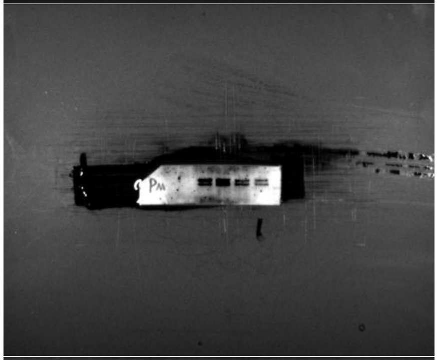
Pm
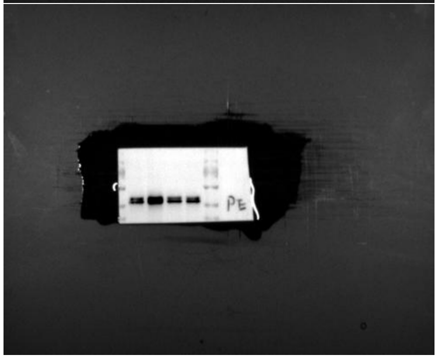
PE
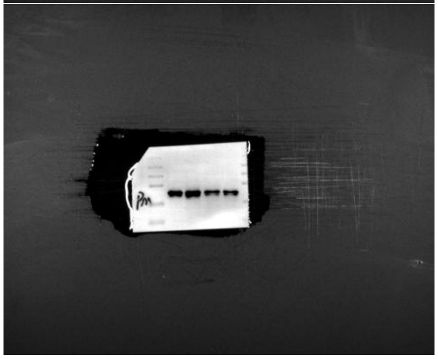
Pm

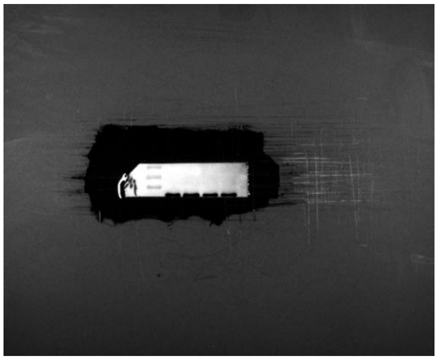

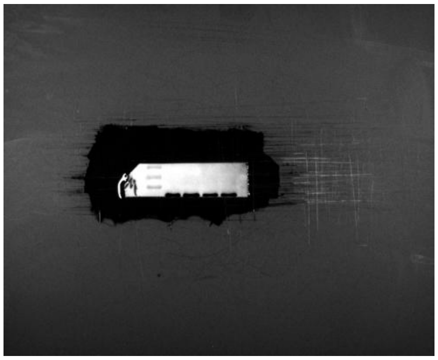

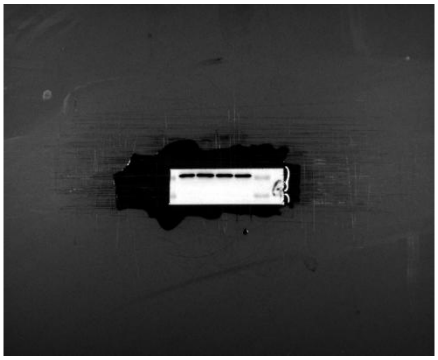

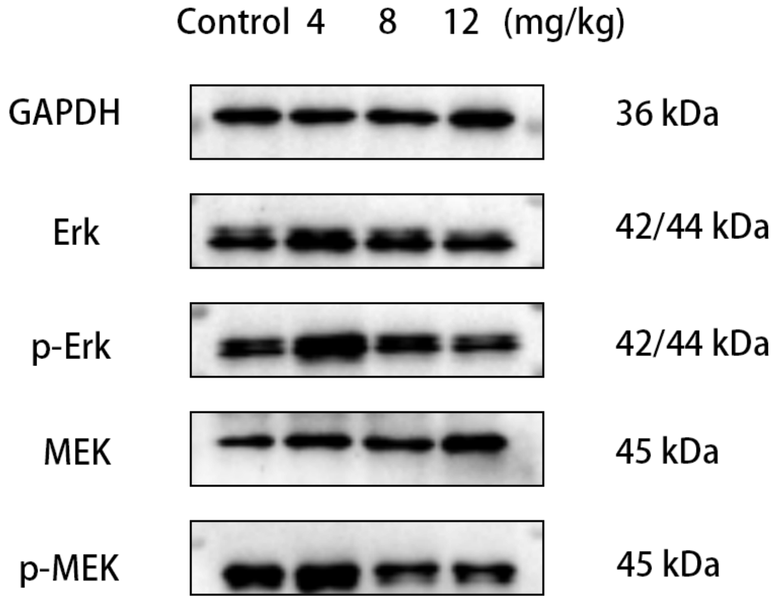
Control 4 8 12 (mg/kg)
GAPDH
36 kDa
Erk
42/44 kDa
p-Erk
42/44 kDa
MEK
45 kDa
p-MEK
45 kDa

MEK

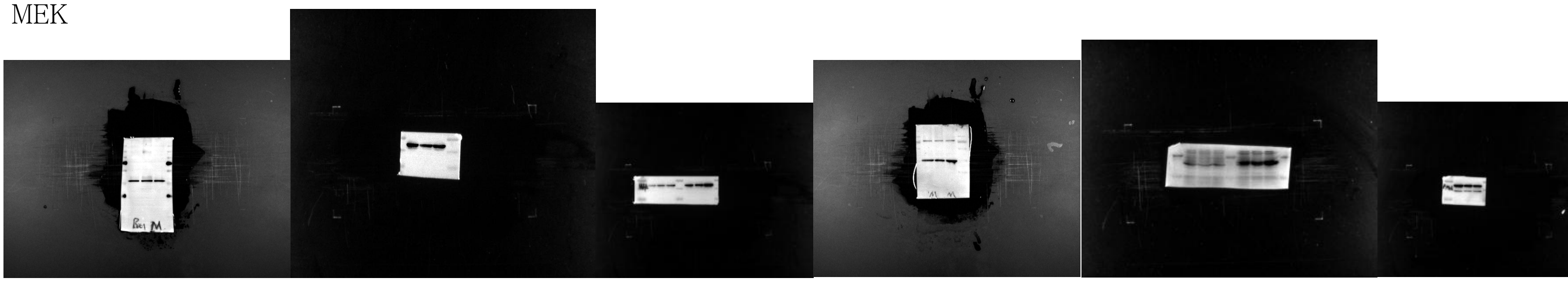

P-Craf

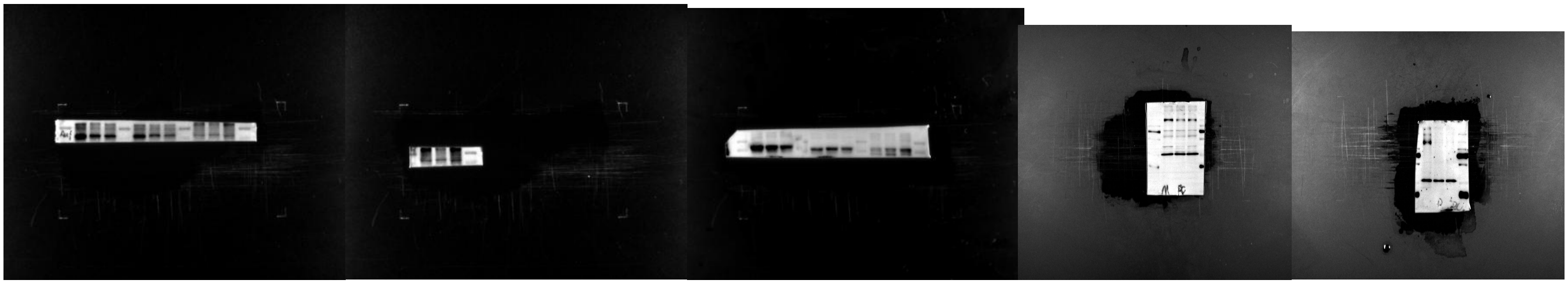

Perk

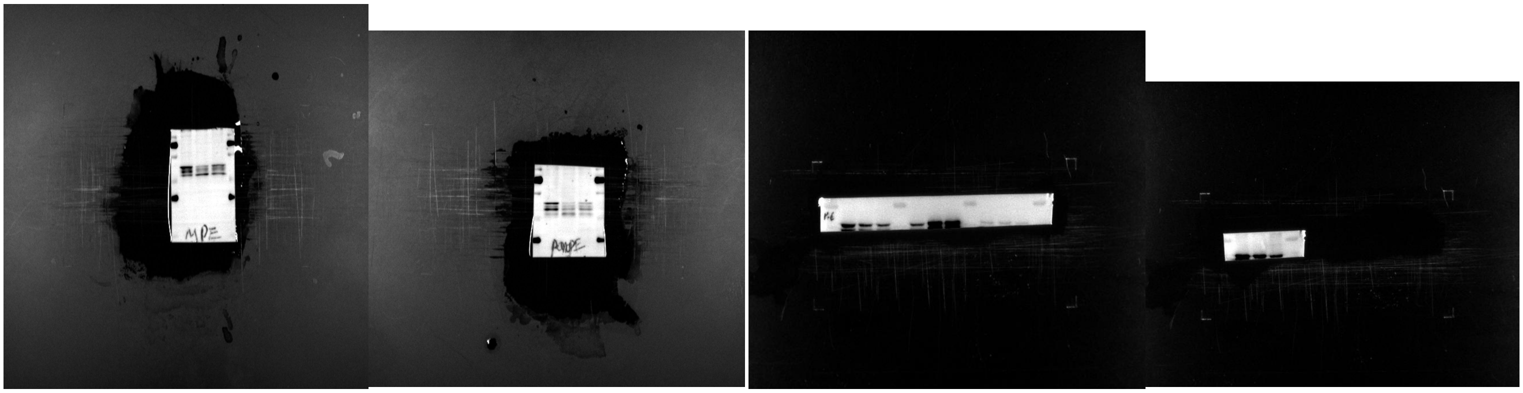

Pakt

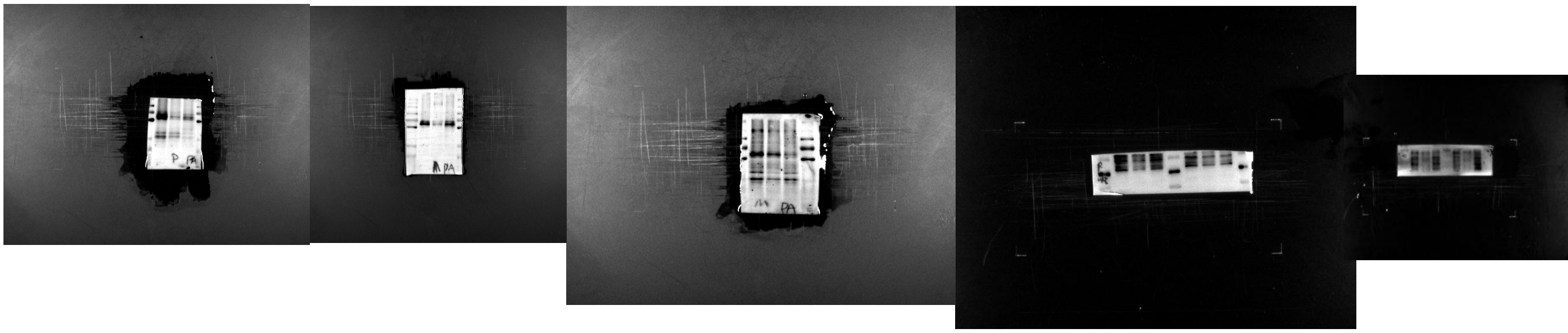

Akt

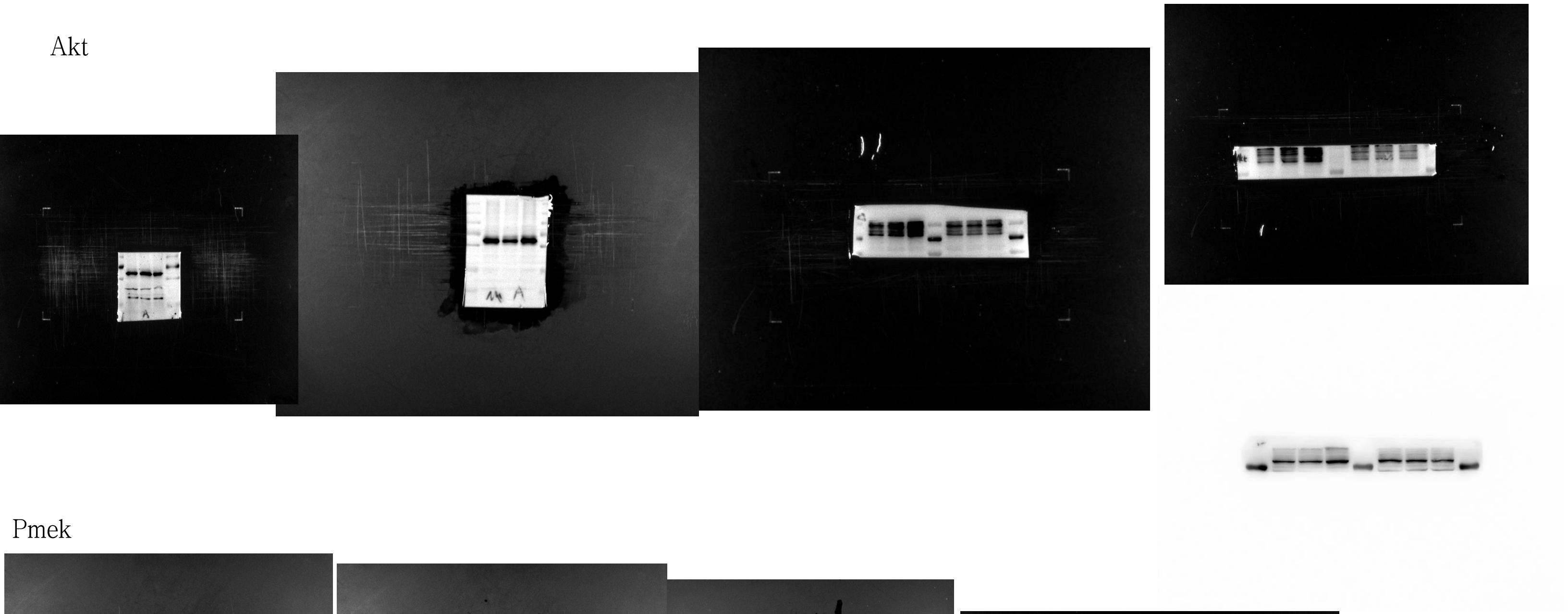

Pmek

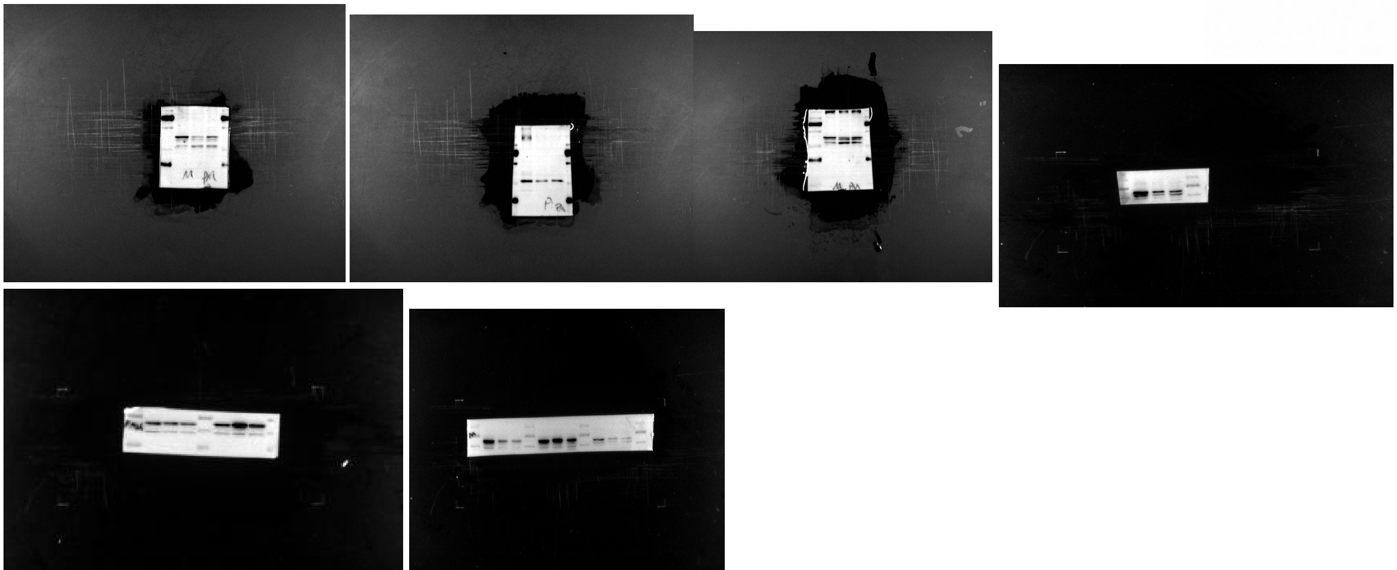

Erk

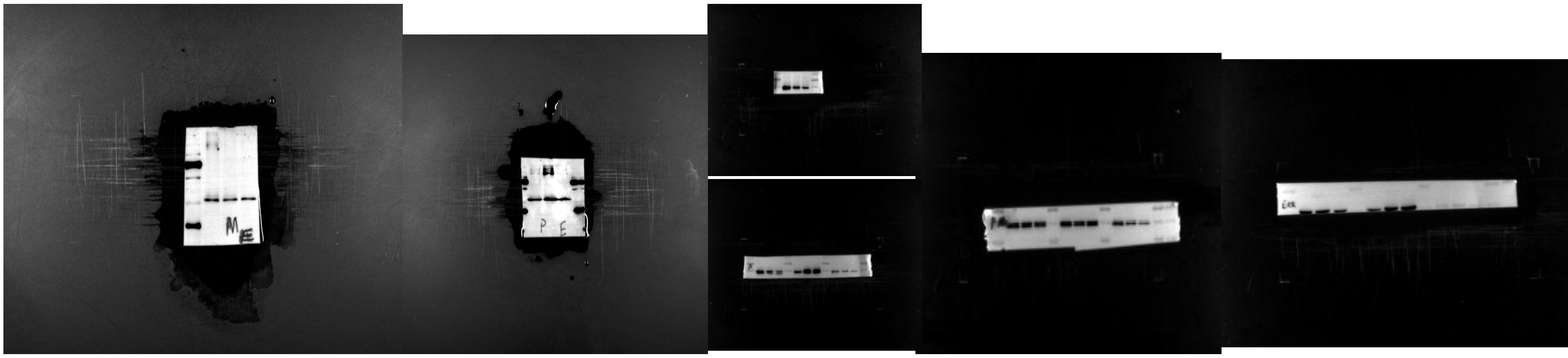

GAPDH

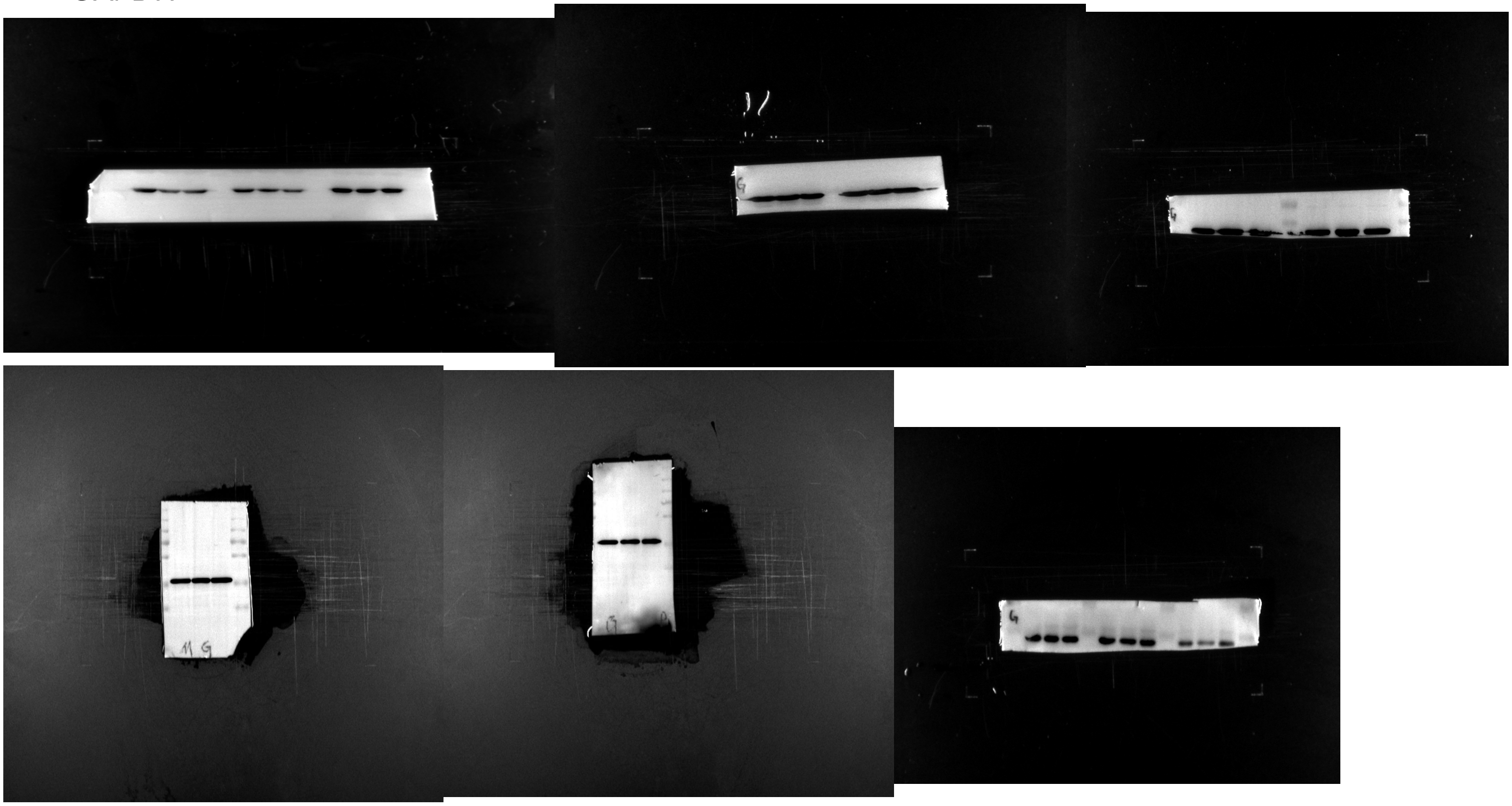

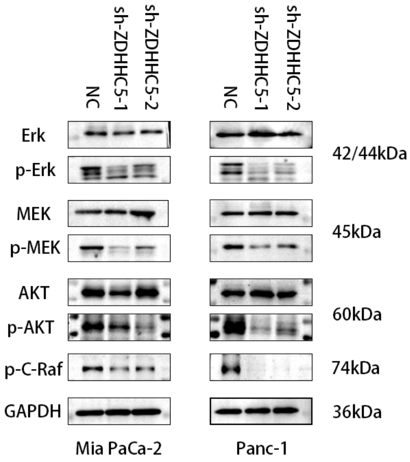
NC
sh-ZDHHC5-1
sh-ZDHHC5-2
NC
sh-ZDHHC5-1
sh-ZDHHC5-2
Erk
p-Erk
42/44kDa
MEK
p-MEK
45kDa
AKT
p-AKT
60kDa
p-C-Raf
74kDa
GAPDH
36kDa
Mia PaCa-2
Panc-1

MTP

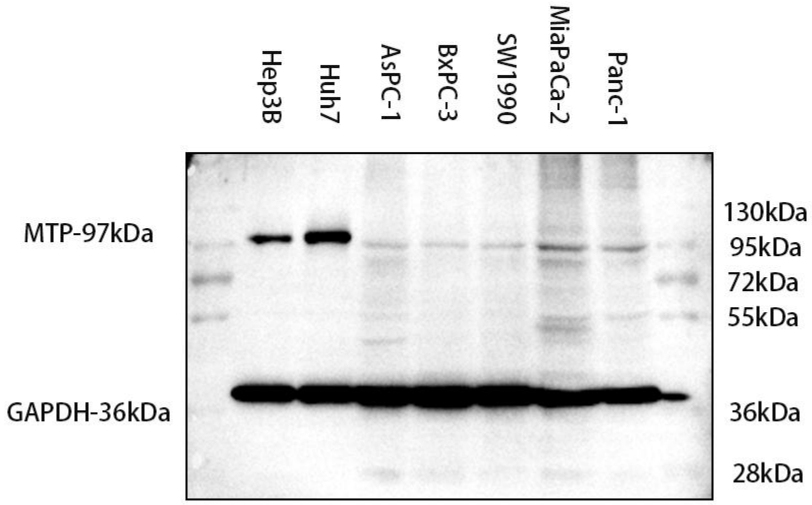
Hep3B
Huh7
AsPC-1
BxPC-3
SW1990
MiaPaCa-2
Panc-1
MTP-97kDa
GAPDH-36kDa
130kDa
95kDa
72kDa
55kDa
36kDa
28kDa

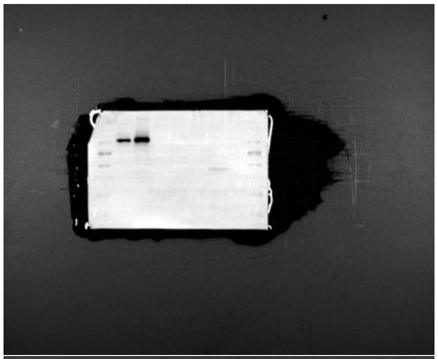
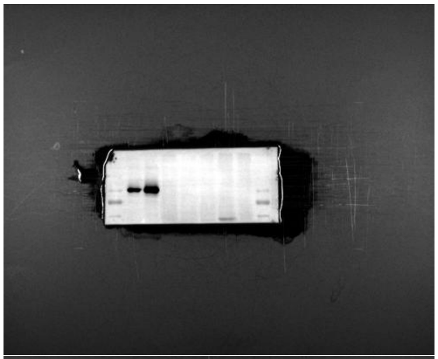
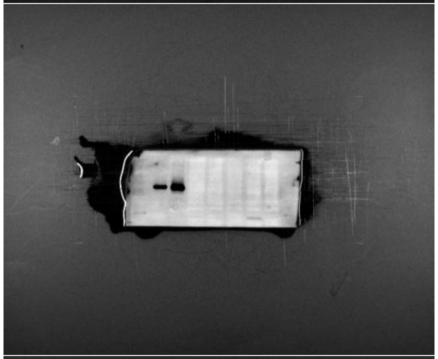
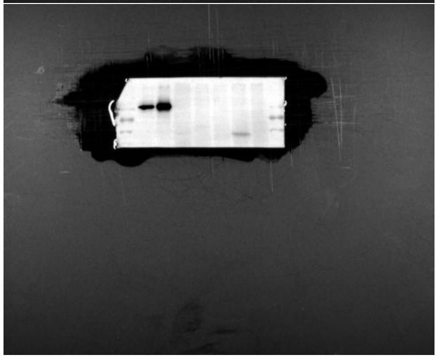
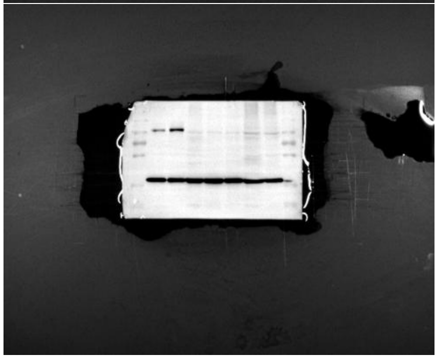

GAPDH

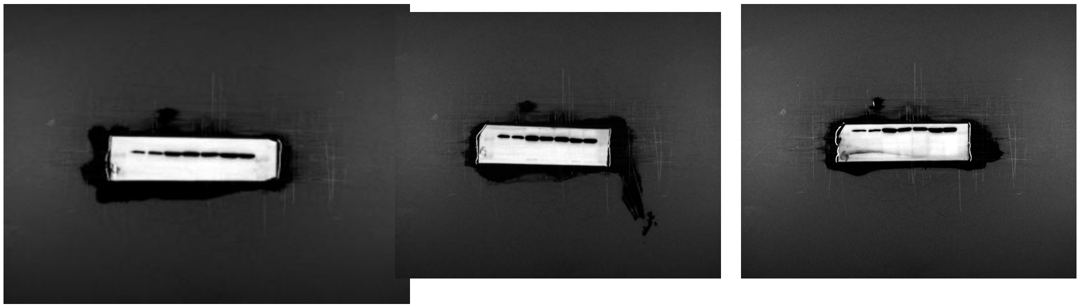

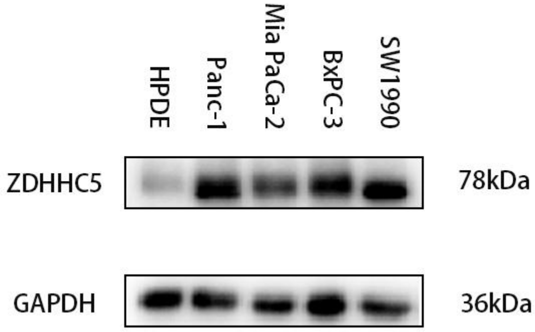

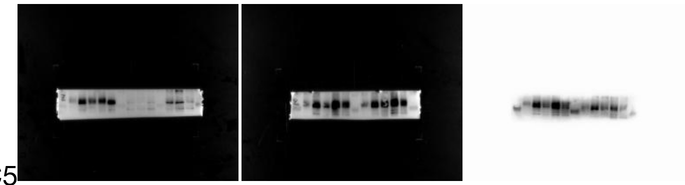

ZDHHC5

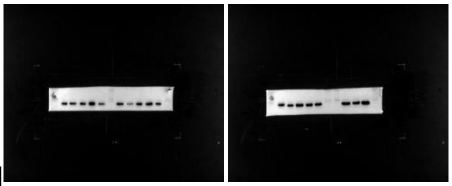

GAPDH

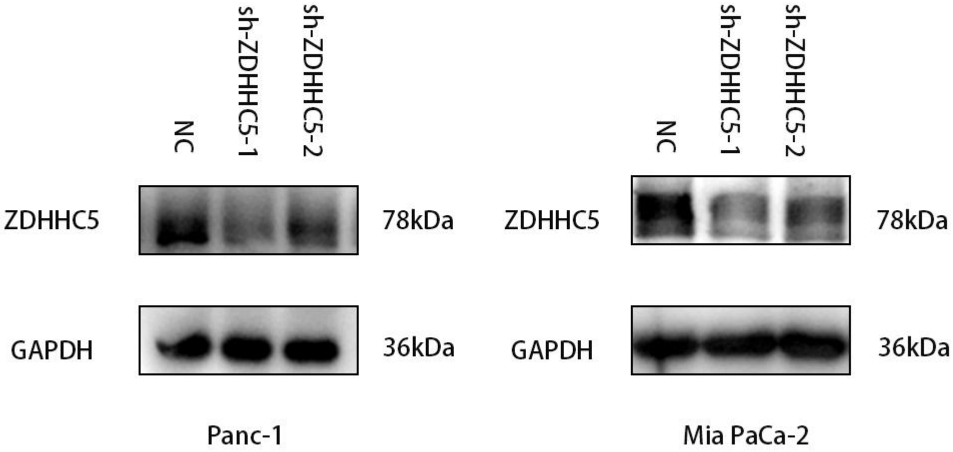

ZDHHC5

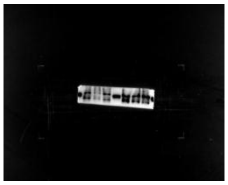
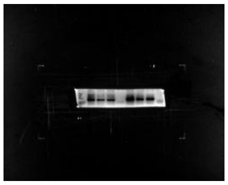
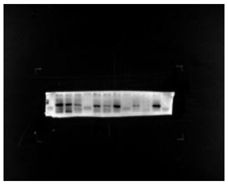
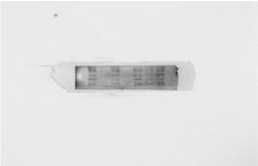
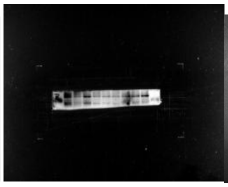
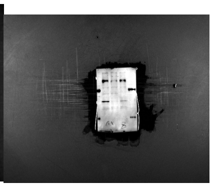
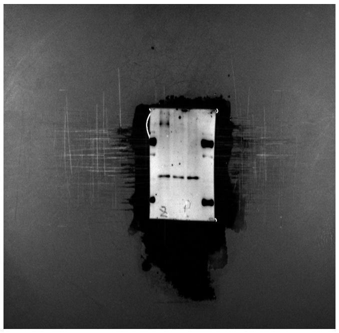

GAPDH

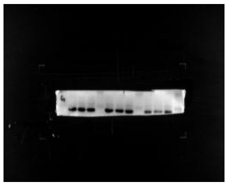
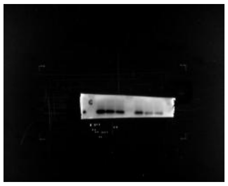
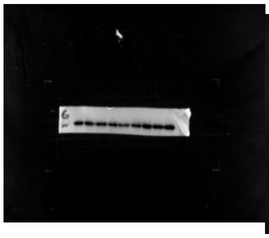
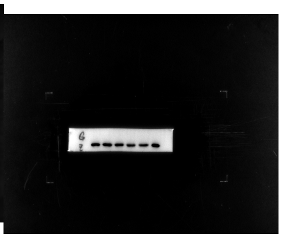

COIP

Input

SSTR5

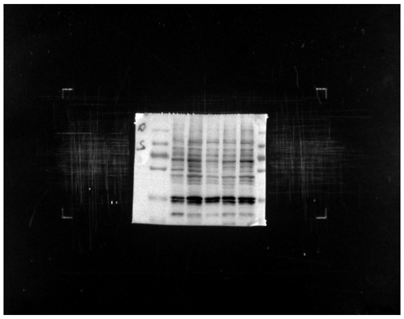

GAPDH

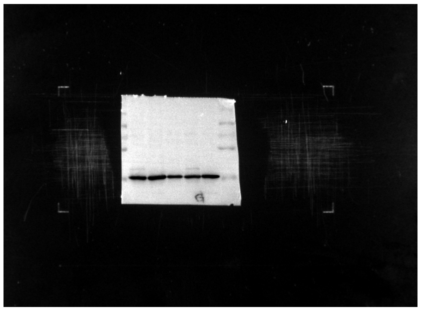

GAPDH-ZDHHC5-SSTR5

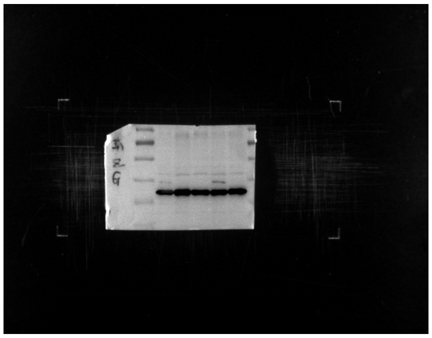

GAPDH

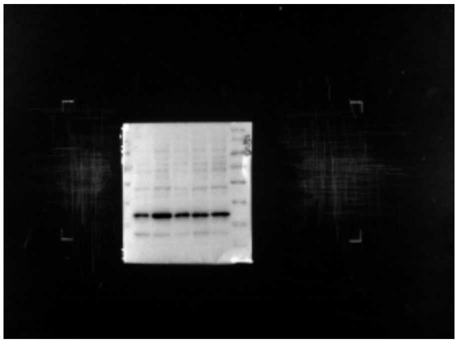

ZDHHC5-SSTR5

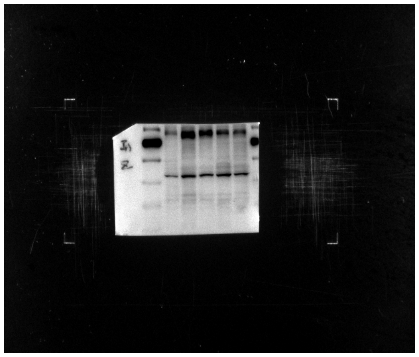

ZDHHC5-SSTR5

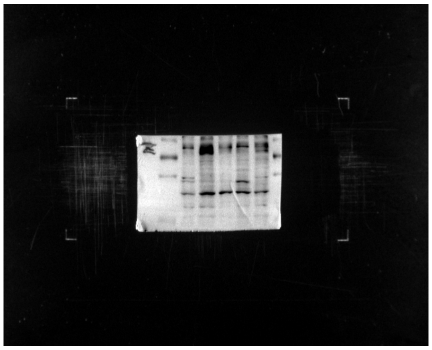

ZDHHC5-SSTR5

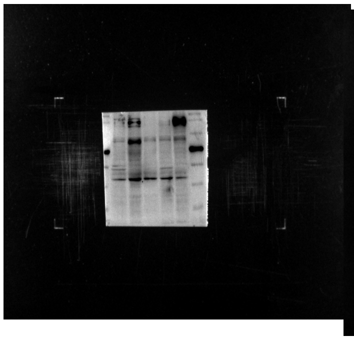

GAPDH-ZDHHC5-SSTR5

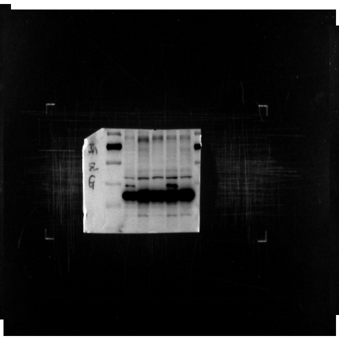

GAPDH-SSTR5

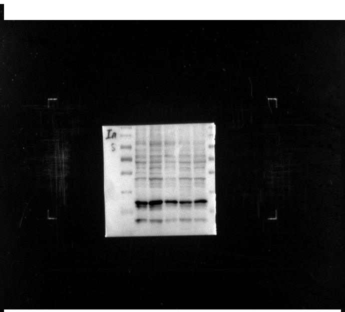

GAPDH-SSTR5

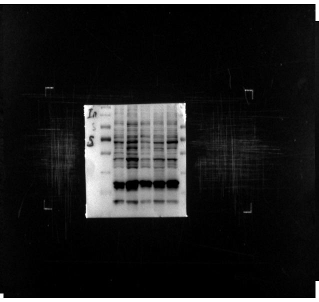

ZDHHC5-SSTR5

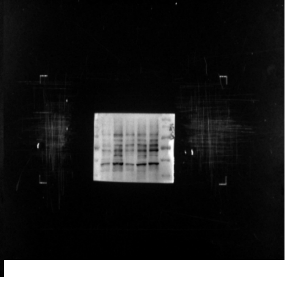

coip-ZDHHC5 , SSTR5

ZDHHC5

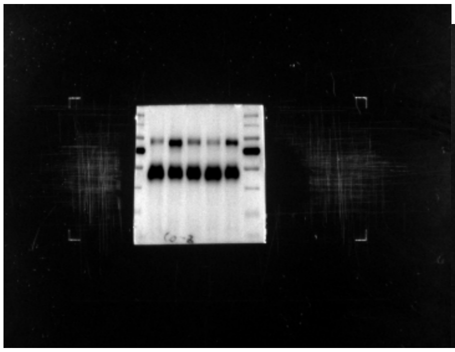

ZDHHC5

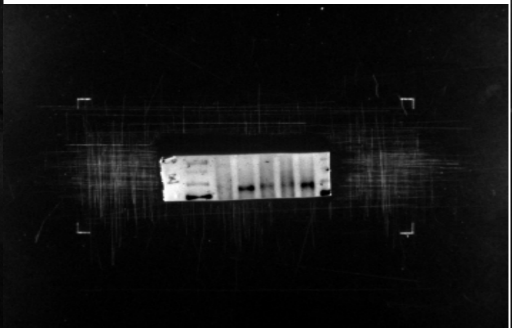

SSTR5

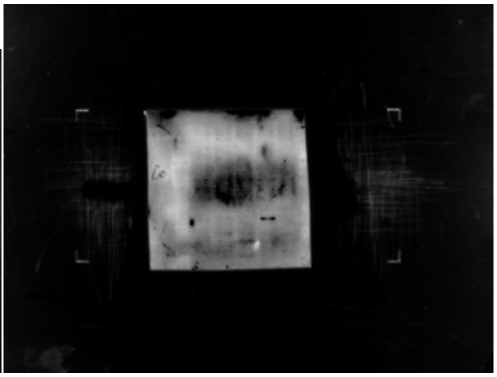

SSTR5

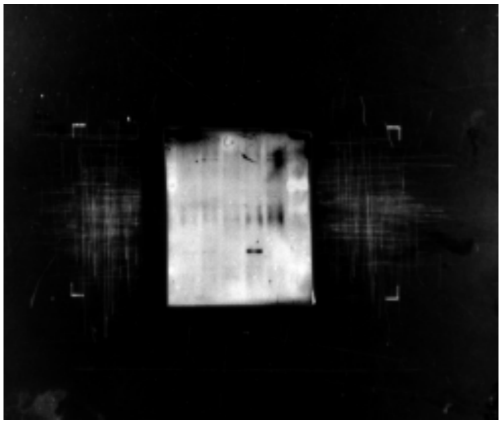

ZDHHC5-SSTR5

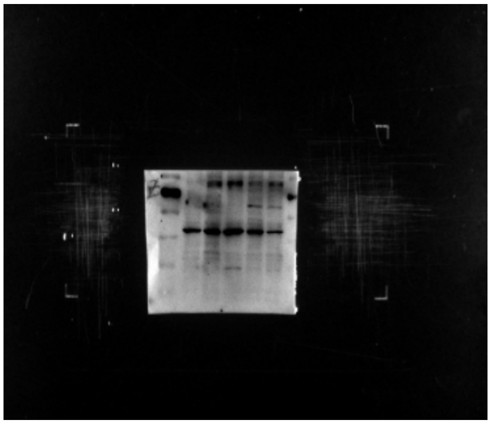

IgG

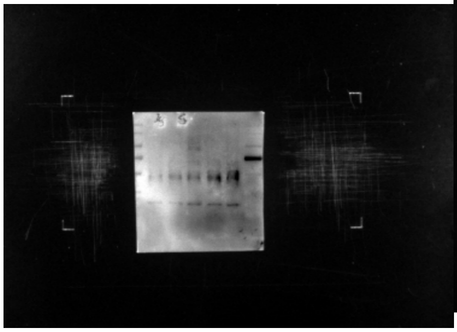

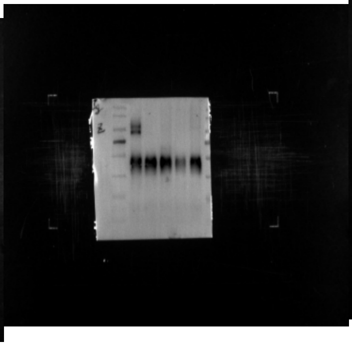

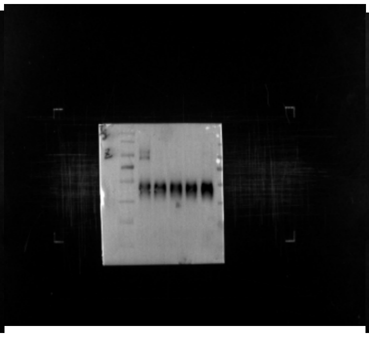

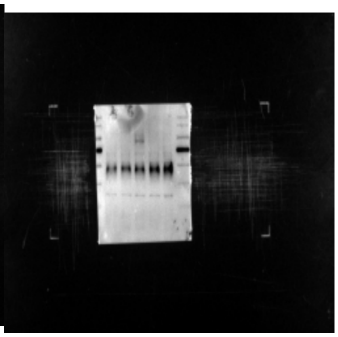

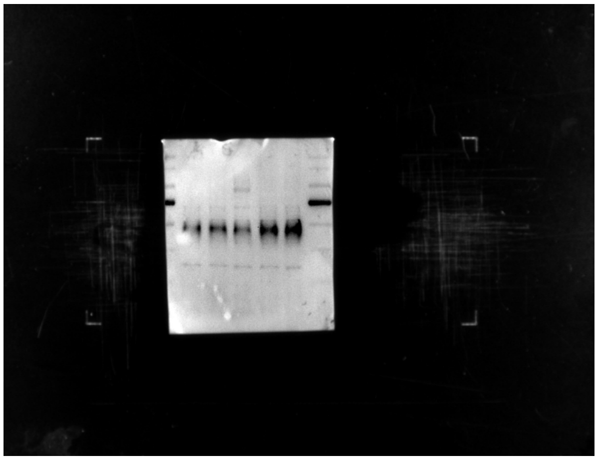

Input

ZDHHC5-SSTR5

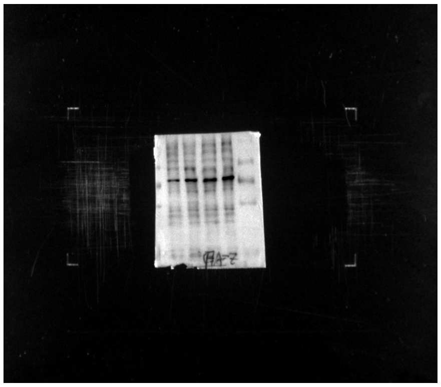

SSTR5

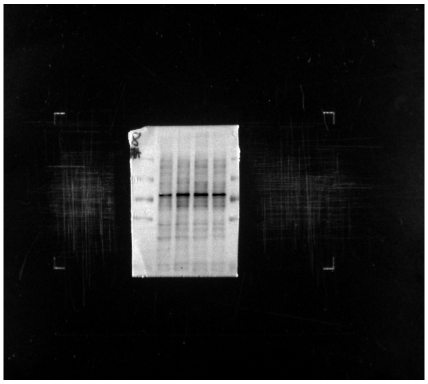

GAPDH-ZDHHC5-SSTR5

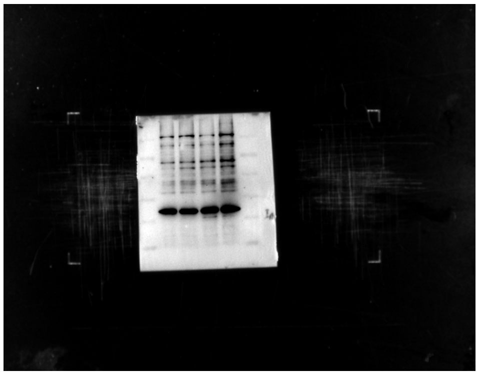

ZDHHC5-SSTR5

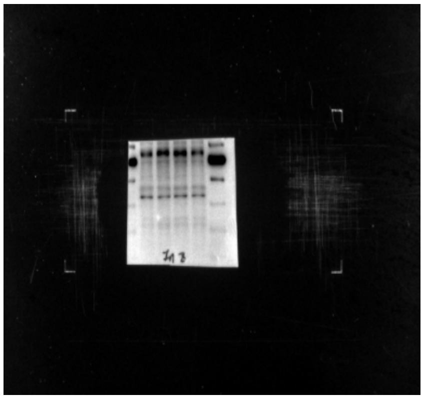

GAPDG-ZDHHC5-SSTR5

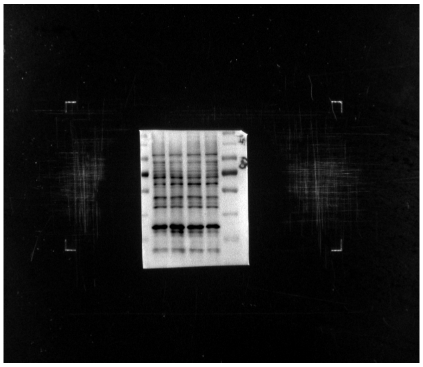

GAPDH

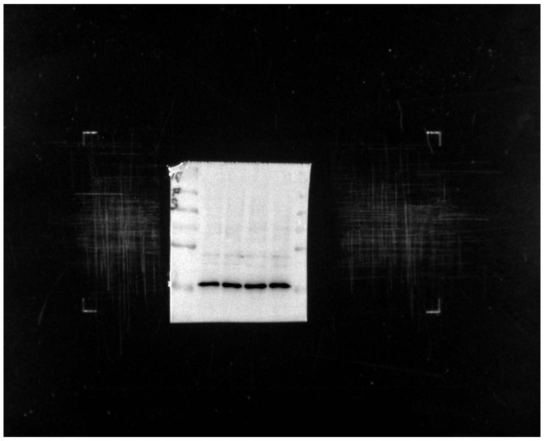

GAPDG-SSTR5

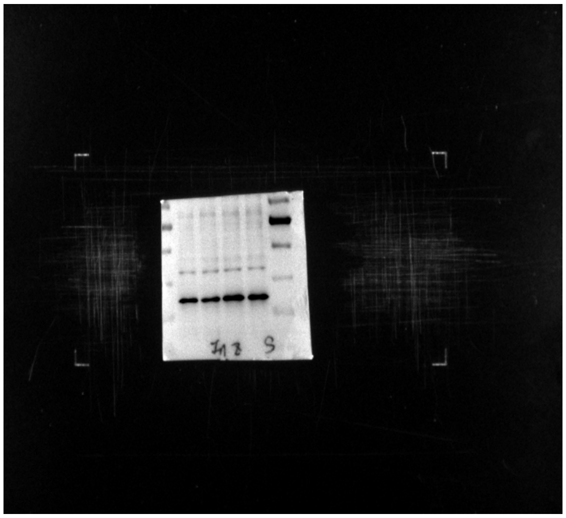

GAPDH

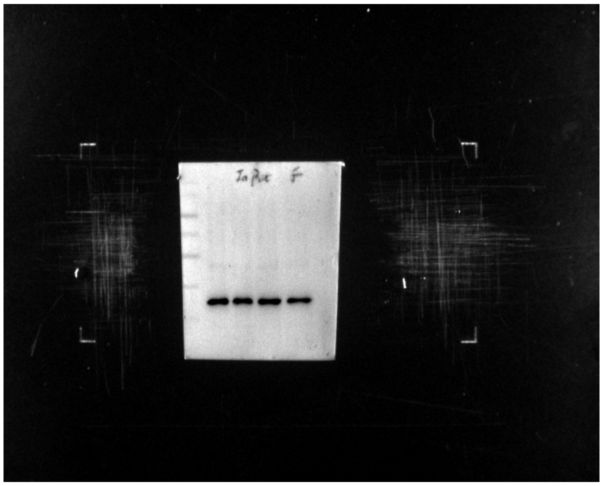

GAPDH-SSTR5

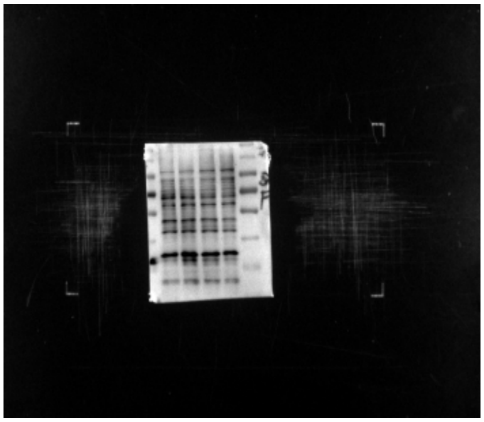

GAPDH-SSTR5-ZDHHC5

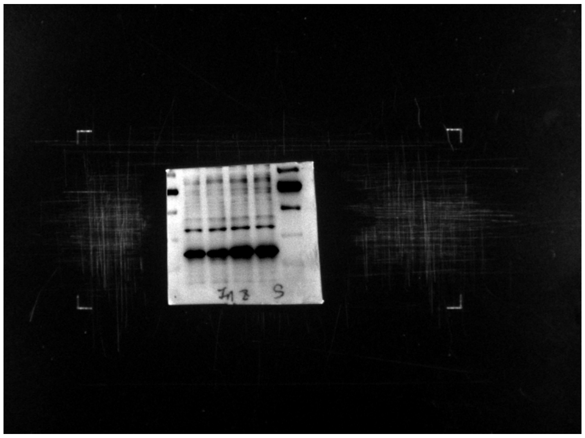

GAPDH

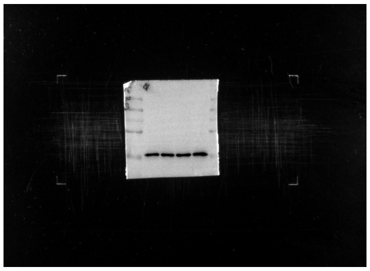

coip-ZDNNC5 ,SSTR5

SSTR5-ZDHHC5

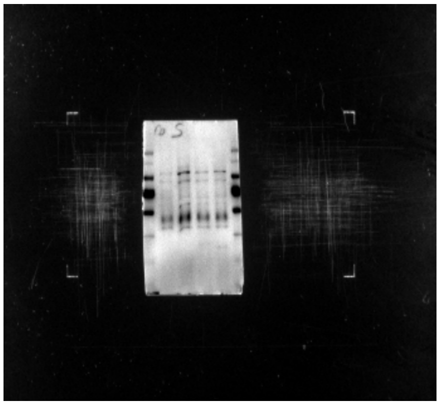

SSTR5-ZDHHC5

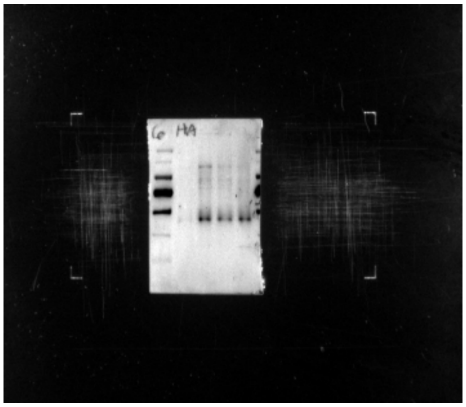

ZDHHC5

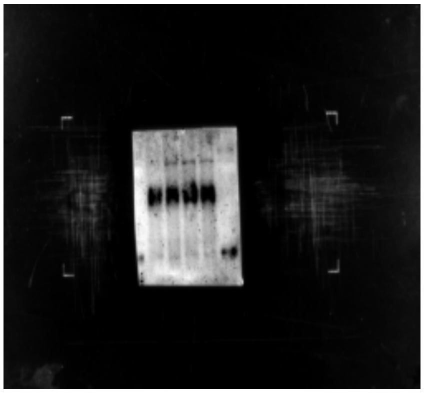

ZDHHC5

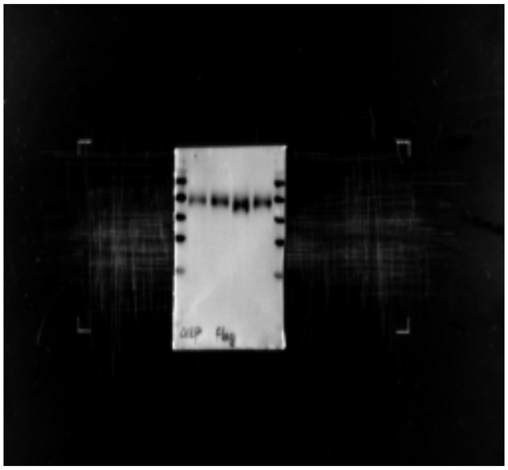

SSTR5-ZDHHC5

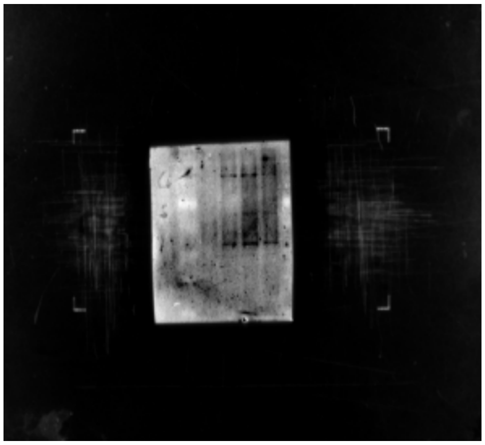

ZDHHC5

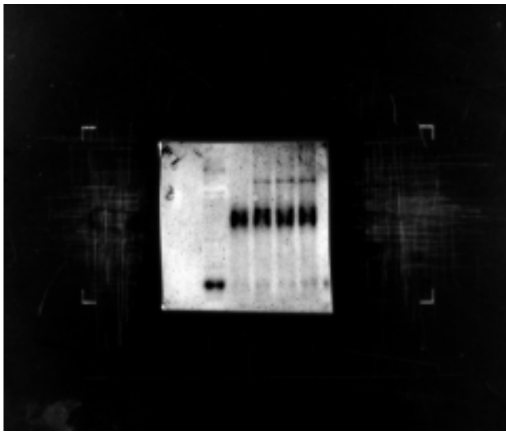

IgG

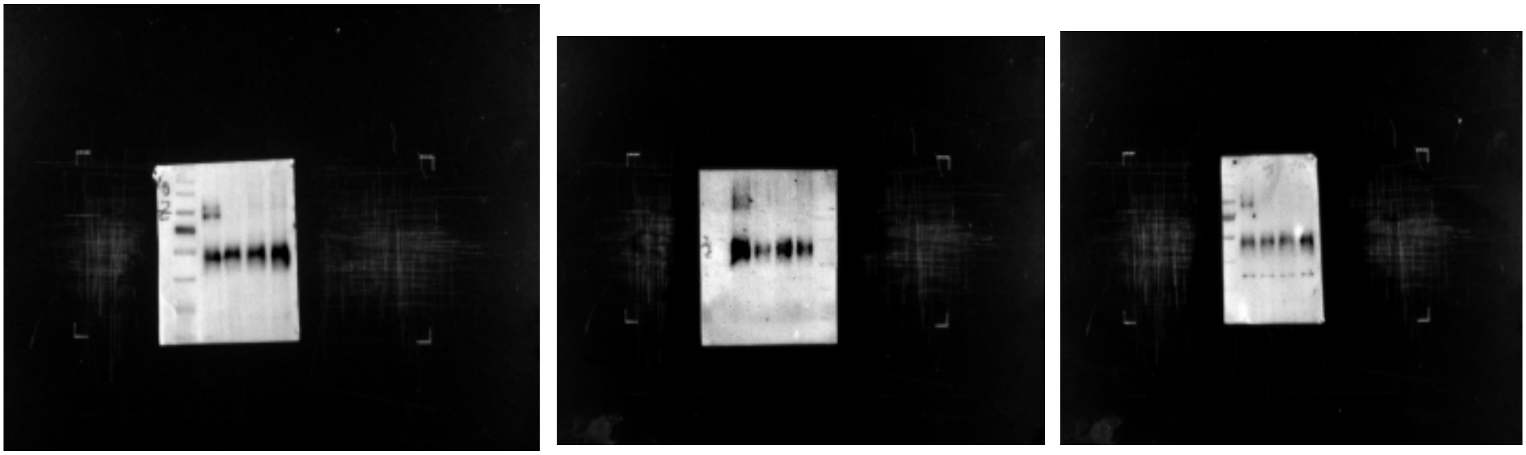

_x0010__x0010_
_x0012_

_x0010_

_x0007_

_x0007_
_x0007_ _x0007_

_x0007_ _x0007_

_x0007_ _x0007__x0007__x0007_ _x0007_ _x0007__x0007__x0007_
_x0007_
_x0007_
_x0007__x0007_
_x0007_
_x0007_

_x0007_

_x0010__x0010_

_x0012_

_x0007_

_x0010_

_x0010_ _x0010_

_x0007__x0010__x0010_

_x0010_

_x0012_

_x0010__x0010_

_x0010_
_x0010__x0016__x0010__x0012__x0012__x0012_

_x0012_

_x0012_

_x0010__x0012_

_x0012__x0010__x0010_

_x0012_

_x0012_

_x0007_

_x0007_
_x0007_

_x0007_ _x0007_

_x0007_
_x0007__x0007_
_x0007__x0007_
_x0007__x0007_

_x0007_
_x0007__x0007__x0007_ _x0007__x0007__x0007__x0007__x0007__x0007__x0
007_
_x0007_
_x0007__x0007_
_x0007__x0007__x0007_

_x0007_

_x0007_

_x0007__x0007_

_x0007_

_x0007_

_x0010_

_x0012_

_x0010__x0010_

_x0010__x0012__x0010_

_x0010_

_x0010__x0012_

_x0012_

_x0010__x0010__x0010_

_x0010_ _x0010_

_x0010_

_x0007_

_x0007__x0007_

_x0007_

_x0007_

_x0007_
_x0007__x0007__x0007__x0007__x0007__x0007_
_x0007__x0007_

_x0007_

_x0007_

_x0007_

_x0007__x0007__x0007_ _x0007_

_x0007__x0007_

_x0007_

_x0007_

_x0010_

_x0010_

_x0010_

_x0010_

_x0010_

_x0010_

_x0010__x0010_
_x0012_

_x0012__x0012_

_x0010__x0012_

_x0012__x0012_

_x0010__x0010_

_x0010_

_x0010_
_x0007_

_x0007_
_x0007_

_x0007_

_x0007__x0007__x0007_
_x0007_ _x0007_
_x0007__x0007_
_x0007__x0007__x0007_
_x0007__x0007_ _x0007_ _x0007_ _x0007_ _x0007__x0007_
_x0007_ _x0007_

_x0007_
_x0007__x0007__x0007_
_x0007__x0007_

_x0010_

_x0010_

_x0010_

_x0010__x0012_

_x0010__x0010_

_x0012__x0012_ _x0012_

_x0019__x0012__x0010__x0010_

_x0010_

_x0010_

_x0016__x0010__x0010_

_x0010_

_x0010__x0012_

_x0010_

_x0007_

_x0007_

_x0007__x0007_
_x0007__x0007__x0007_ _x0007__x0007_
_x0007_
_x0007_

_x0007__x0007__x0007__x0007__x0007_ _x0007_ _x0007__
x0007__x0007_ _x0007_
_x0007_ _x0007__x0007_ _x0007_
_x0007__x0007__x0007_
_x0007_

_x0007_

_x0007_

_x0010_

_x0007__x0007_

_x0010_

_x0012_

_x0010_

_x0010_

_x0016__x0012__x0010__x0010__x0010__x0010__x0016__x0012__x0010_

_x0016_
_x0010_

_x0012__x0016_

_x0012_

_x0010__x0010_

_x0010_

_x0010__x0012_

_x0010__x0010_
_x0010_

_x0012_

_x0010__x0012_

_x0010_

_x0010__x0010__x0012__x0010__x0010__x0012__x0016_

_x0010_

_x0010__x0012_

_x0010__x0012__x0019_

_x0010_

_x0007_

_x0007_

_x0007_

_x0007_

_x0007_
_x0007_
_x0007_

_x0007__x0007__x0007_ _x0007_ _x0007__x0007__x0007_ _x0007_

_x0007_ _x0007_ _x0007_ _x0007_ _x0007_
_x0007_
_x0007_ _x0007_

_x0007_
_x0007_ _x0007_
_x0007_

_x0007_

_x0007_ _x0007__x0010_

_x0010_

_x0010_

_x0012_

_x0010_

_x0012_

_x0010_

_x0012__x0012__x0010__x0016__x0012__x0012__x0012__x0012_

_x0016_

_x0010__x0010__x0012_

_x0007_

_x0007_

_x0007__x0007_

_x0010_

_x0007__x0007_

_x0007_ _x0007_ _x0007_

_x0007__x0007_

_x0007_ _x0007_
_x0007__x0007_ _x0007__x0007_ _x0007_ _x0007_
_x0007_
_x0007__x0007__x0007_ _x0007_

_x0010_

_x0007_ _x0007_
_x0007_
_x0007_
_x0007_

_x0010_

_x0010_

_x0010_

_x0012_

_x0010__x0010_

_x0010_

_x0010_

_x0010_

_x0012__x0010__x0012__x0016__x0012_

_x0010_

_x0007_

_x0007_

_x0010_

_x0007_

_x0010__x0016_

_x0010_

_x0010_

_x0010_

_x0012__x0016__x0016__x0012_

_x0010__x0010__x0019__x0016__x0012__x0010_

_x0010_

_x0010_

_x0012_

_x0012_

_x0012_

_x0007_
_x0007_

_x0007_

_x0007_ _x0007__x0007_ _x0007__x0007_ _x0007_ _x0007__x0007_
_x0007_
_x0007_
_x0007_
_x0007_

_x0007__x0007__x0007__x0007__x0007_
_x0007_ _x0007__x0007_

_x0007_

_x0012_

_x0012_

_x0012_

_x0012__x0012_

_x0012__x0010_

_x0012__x0010_

_x0010_
_x0010__x0012_

_x0012_

_x0010__x0019__x0019_

_x0010__x0010__x0012_

_x0007_ _x0007_

_x0007_

_x0007__x0007__x0007_

_x0007_
_x0007_

_x0007_ _x0007__x0007__x0007_ _x0007_ _x0007_
_x0007_
_x0007__x0007_
_x0007_ _x0007_

_x0007_

_x0007_

_x0007_

_x0010_

_x0007_

_x0012_

_x0010_

_x0010__x0010__x0010_

_x0010_

_x0010_

_x0010_

_x0012_

_x0010_

_x0007_

_x0012__x0012__x0016_

_x0016__x0012__x0016__x0010__x0012_

_x0012__x0016_

_x0010_

_x0007_ _x0007_

_x0007_ _x0007_

_x0007_

_x0007_
_x0007_
_x0007_
_x0007_ _x0007_ _x0007_

_x0007_ _x0007__x0007_ _x0007_

_x0007_

_x0007_
_x0007__x0007_

_x0007_

_x0007_ _x0007_

_x0012_

_x0010_

_x0010_

_x0012_

_x0010_

_x0010__x0010_

_x0012_

_x0012__x0012__x0010_

_x0010_

_x0010__x0012__x0019__x0019__x0016_
_x0016__x0016__x0010_

_x0010_

_x0010__x0012_

_x0016__x0010_

_x0010__x0010_

_x0012_

_x0007_

_x0007_ _x0007_
_x0007__x0007_

_x0007_

_x0007_
_x0007__x0007__x0007__x0007__x0007_ _x0007__x0007_ _x0007__x0007_ _x0
007_ _x0007_ _x0007_
_x0007_ _x0007_
_x0007_
_x0007_

_x0007_

_x0010_

_x0010_

_x0010_

_x0010_

_x0010_

_x0010_

_x0010_

_x0010_
_x0010__x0012_

_x0010_

_x0012_

_x0010_

_x0010__x0010_

_x0016_

_x0010__x0016__x0010_

_x0016__x0012__x0016__x0010_

_x0012__x0010_

_x0010_

_x0012_

_x0010_

_x0012_

_x0007_

_x0007_

_x0007_

_x0007_

_x0007_

_x0007_

_x0007_

_x0007__x0007_
_x0007__x0007__x0007__x0007__x0007__x0007_ _x0007_

_x0007__x0007_ _x0007_

_x0007_

_x0010_

_x0012_

_x0010_
_x0010_

_x0010_
_x0010_

_x0010__x0010_

_x0007_

_x0010_

_x0010_

_x0010_

_x0010__x0012_

_x0010_

_x0010_

_x0012__x0010__x0012_

_x0012__x0010_

_x0007_

_x0007_
_x0007__x0010_

_x0007_

_x0007_

_x0007_ _x0007__x0007_ _x0007__x0007__x0007__x0007__x0007__x0007_
_x0007_
_x0007__x0007__x0
007_ _x0007__x0007__x0007__x0007_
_x0007__x0007__x0007_
_x0007_

_x0007_

_x0007_

_x0007_

_x0007__x0007_

_x0007_
_x0007_

_x0010__x0012__x0010__x0012_

_x0010_

_x0010_
_x0010__x0010__x0010_

_x0010_

_x0012__x0010__x0012__x0012_

_x0010__x0012_

_x0010_

_x0010_

_x0016__x0012__x0016__x0019_

_x0010__x0016__x0016_

_x0010__x0012_

_x0010__x0012_

_x0010__x0012__x0012__x0010_

_x0012__x0010__x0010_

_x0010_

_x0007_

_x0007_

_x0007_

_x0007_ _x0007__x0007_ _x0007_ _x0007_

_x0007__x0007__x0007_ _x0007_ _x0007_ _x0007__x0007_
_x0007_
_x0007_ _x0007__x0007_ _x0007__x0007_
_x0007_
_x0007_

_x0007_

_x0007_

_x0007_

_x0007_

_x0007_

_x0010_

_x0010__x0007_

_x0010_

_x0012_

_x0012_

_x0010_

_x0012_

_x0010_

_x0012_

_x0012__x0016__x0010__x0012__x0012_

_x0012_

_x0010_

_x0010__x0010__x0010_
_x0010_

_x0010_

_x0010_

_x0012__x0012_

_x0012_

_x0007__x0007_ _x0007_

_x0007_

_x0007_

_x0007__x0007_

_x0007_ _x0007__x0007__x0007_
_x0007_ _x0007
__x0007__x0007__x0007__x0007__x0007_

_x0007__x0007__x0007_

_x0007_

_x0007_

_x0010_

_x0007_
_x0007_

_x0007_

_x0007_

_x0012_

_x0010_

_x0010_

_x0010_

_x0010__x0010__x0010_

_x0010_
_x0010_

_x0010__x0012__x0016__x0019__x0019__x0019_
_x0012_

_x0010__x0012_

_x0012_

_x0016__x0010_

_x0012_-_x0010_

_x0012_

_x0010_

_x0007_

_x0007_

_x0007_

_x0007__x0007__x0007__x0007__x0007_

_x0007__x0007_ _x0007__x0007__x0007_ _x0007_ _x0007_ _x0007_
_x0007_
_x0007__x0007__x0007__x0007_
_x0007_

_x0012_

_x0010_

_x0007_

_x0010_

_x0010_

_x0012__x0010_

_x0016_

_x0016_

_x0010_

_x0012__x0012__x0016__x0019__x0012__x0010__x0012__x0019__x0019__x0010__x0012_

_x0012__x0012__x0012__x0016__x0012_

_x0012_

_x0010__x0012_

_x0012__x0019_

_x0010__x0012__x0012_

_x0010_

_x0007_

_x0007__x0007__x0007_

_x0007__x0007_ _x0007_ _x0007_ _x0007_ _x0007_ _x0007_
_x0007_ %_x0007_
_x0007_
_x0007_ _x0007__x0007_ _x0007_ _x0007__x0007_
_x0007_

_x0007__x0007_
_x0007_

_x0007_

_x0007__x0010_

_x0007_ _x0007_

_x0010_

_x0010__x0012_

_x0010_

_x0010_

_x0012_

_x0010__x0016__x0016_

_x0010__x0010__x0012_

_x0010__x0010_
_x0012_

_x001D_

_x0010_

_x0010_

_x0010__x0012_

_x0012__x0012_

_x0007_

_x0007_

_x0007__x0007__x0007__x0007_ _x0007__x0007__x0007__x0007_ _x0007_

_x0007_

_x0007__x0007_ _x0007_ _x0007__x0007_ _x0007_
&_x0010_ _x0007_
_x0007__x0007_ _x0007__x0007_
_x0007__x0007_ _x0007_

_x0007__x0007__x0007_

_x0007_

_x0007__x0007_

_x0010_

_x0010_

_x0010_

_x0010_

_x0012_

_x0010_

_x0010__x0010_

_x0010_
_x0012_ _x0010_

_x0010__x0010_

_x0010_

_x0010__x0010_

_x0010_
_x0010_

_x0010__x0012__x0012__x0010__x0010__x0019__x0012_

_x0010_

_x0016__x0010__x0010__x0016_

_x0010_

_x0010_

_x0010__x0010_

_x0010_

_x0010_

_x0010_

_x0010_

_x0007_

	_x0007_	
_x0007_	_x0007_	_x0007_
_x0007_		

_x0007_
_x0007_

_x0007_
_x0007_ _x0007__x0007__x0007_
_x0007__x0007__x0007__x0007__x0007_ _x0007_
_x0007_

_x0007_ _x0007__x0007__x0007_
_x0007__x0007__x0007_

_x0007_ _x0007__x0007_

_x0010_

_x0010_

_x0010_

_x0010_

_x0012_

_x0012_

_x0010_

_x0016_

_x0010_

_x0012__x0010_

_x0012_
_x0016_

_x0010__x0012_

_x0010_

_x0010_
_x0010_

_x0012__x0007_

_x0007_

_x0007__x0007__x0007__x0007_

_x0007__x0010_ _x0007__x0007_
_x0007_ _x0007_ _x0007_

_x0007_ _x0007__x0007_ _x0007_
_x0007_
_x0007__x0007_ _x0007_ _x0007_
_x0007__x0007_

_x0007_

_x0007_
_x0007_

_x0007_
_x0007_

_x0007_

_x0010_

_x0007_

_x0007_

_x0010__x0010_

_x0010_

_x0010__x0012_

_x0010__x0010__x0010_

_x0012_

_x0012__x0012__x0010__x0012__x0019__x0016_

_x0010__x0012__x0010__x0016__x0010__x0019_
_x0010_

_x0010_

_x0010_

_x0010_

_x0007_

_x0010_

_x0010_

_x0007_

_x0007_

_x0007__x0007_
_x0007_
_x0007_

_x0007_ _x0007_ _x0007_

_x0007__x0007_ _x0007_

_x0007__x0007__x0007_

_x0007_

_x0007_ _x0007__x0007__x0007_
_x0007_ _x0007__x0007_

_x0007_ _x0007_

_x0007_

_x0007_
_x0007_
_x0007_

_x0007__x0007_

_x0010_

_x0007_

_x0010_

_x0012_

_x0010_

_x0012_

_x0012__x0010_

_x0012_

_x0012_
_x0012__x0010_
_x0010__x0010__x0010__x0010_

_x0016__x0012_

_x0012__x0012_

_x0012__x0012__x0012__x0012_

_x0012_

_x0010_

_x0010_

_x0007_

_x0007_

_x0007__x0007_ _x0007_
_x0007_

_x0007__x0007_
_x0007__x0007__x0007_

_x0007_

_x0007__x0007__x0007_ _x0007__x0007__x0007_
_x0007_

_x0007_
_x0007__x0007_ _x0007_ _x0007_ _x0007_
_x0007__x0007__x0007_ _x0007_ _x0007_ _x0007_
_x0007_ _x0007_ _x0007_
_x0007_ _x0007_ _x0007_ _x0007_
_x0007_

_x0007_

_x0007_

_x0007_

_x0012_

_x0010__x0007_

_x0012_

_x0012_

_x0012_

_x0010_

_x0010__x0012_

_x0010_

_x0016__x0016_

_x0010__x0012__x0010__x0010_

_x0010__x0012_

_x0012__x0010__x0012_
_x0012__x0010__x0010_

_x0016__x0010_

_x0012_

_x0010_

_x0012_

_x0007_
_x0012__x0007_

_x0007_

_x0007_
_x0007_

_x0010__x0007_
_x0007_ _x0007_ _x0007_

_x0007__x0007_ _x0007__x0007__x0007_
_x0007_
_x0007_ _x0007_ _x0007__x0007__x0007_

_x0007_ _x0007_
_x0007_ _x0007_

_x0007_

_x0010__x0007_

_x0010__x0007__x0007_

_x0007_

_x0012_
_x0010_

_x0010__x0010_

_x0016_

_x0012_

_x0010_

_x0010__x0019__x0019__x0016__x0016__x0019__x0016__x0012__x0012_

_x0012__x0012_

_x0012_
_x0010_

_x0010_

_x0012_

_x0010_

_x0012_

_x0007_ _x0007_

_x0007_

_x0007_
_x0007_
_x0007_
_x0007__x0007_
_x0007_
_x0007_
_x0007_
_x0007_ _x0007__x0007_ _x0007__x0007_ _x0007_ _x0007__x0007__x0007_ _x0007
__x0007__x0012_

_x000

7_ _x0007__x0007__x0007__x0007_ _x0007_

_x0010_

_x0010__x0010_

_x0010_

_x0012_

_x0012__x0010_

_x0010_

_x0010_

_x0010__x0010_
_x0016__x0016__x0012_

_x0010__x0019_

_x0012__x0016__x0016_

_x0010_

_x0010__x0010_
_x0010__x0010_

_x0010_

_x0010_

_x0007_

_x0007_ _x0007_ _x0007_

_x0007__x0007_

_x0007_

_x0007_

_x0007_
_x0007__x0007_

_x0007_ _x0007__x0007_ _x0007_ _x0007_ _x0007__x0007_ _x0007_

_x0007_ _x0007__x0007__x0007__x0007_ _x0007__x0007_

_x0007__x0007_

_x0007_

_x0012_

_x0010_

_x0010_

_x0010_

_x0010__x0010_

_x0010__x0010_

_x0012_

_x0010__x0012__x0012_

_x0010_

_x0010_

_x0012__x0016__x0019__x0019__x0012_

_x0016__x0012__x0016__x0016__x0016_

_x0010_

_x0010__x0012_
_x0010_

_x0012_

_x0012_

_x0010_

_x0007_

_x0007__x0007__x0007_

_x0007__x0007__x0007_

_x0007_

_x0007_

_x0007_

_x0007__x0007_

_x0007__x0007_ _x0007_ _x0007_ _x0007__x0007_
_x0007_
_x0007_ _x0007_ _x0007_ _x0007_ _x000
7_ _x0007_
_x0007__x0007__x0007__x0007_
_x0007__x0007__x0007__x0007__x0007_

_x0007__x0007_
_x0007_

_x0007_ _x0007__x0007__x0007_

_x0007__x0007__x0007_

_x0010_

_x0010_

_x0007__x0010_
_x0010__x0012__x0016_

_x0010_

_x0010_

_x0010_

_x0010_

_x0012__x0012_

_x0012_

_x0016_
_x0016__x0019__x0016__x0016_

_x0012__x0012_
_x0019__x0012__x0019__x0016_

_x0012__x0012_

_x0010__x0010_

_x0007_

_x0007_

_x0007_

_x0007_

_x0012_

_x0007_

_x0007_
_x0007_

_x0007__x0007__x0007_
_x0007__x0007_ _x0007__x0007_
_x0007_
_x0007_ _x0007__x0007_
_x0007__x0007__x0007__x0007__x0007_ _x0007__x0007_

_x0007_

_x0007__x0007__x0007__x0007_

_x0007_

_x0010_

_x0007_

_x0010_

_x0010__x0010_

_x0010__x0012_

_x0010_

_x0010_

_x0012_

_x0016_

_x0010_

_x0010__x0010_

_x0012_

_x0012__x0012__x0010__x0010__x0019__x0016__x0012__x0016__x0016__x0012_

_x0012__x0010__x0010_

_x0010_

_x0010_

_x0010_

_x0007_

_x0016__x0019_

_x0007__x0007_
_x0007_

_x0007_ _x0007__x0007__x0007__x0007__x0007_ _x0007__x0007_

_x0007_ _x0007_ _x0007_ _x0007__x0007_
_x0007_ _x0007_
_x0007__x0007__x0007__x0007__x0007_
_x0007__x0007__x0007_
_x0007_

_x0007_

_x0007_ _x0007_

_x0007_

_x0010_

_x0010_
_x0010__x0012_
_x0010_

_x0010__x0010_

_x0012_
_x0010__x0007__x0010_

_x0010_

_x0010_
_x0010_
_x0012_

_x0010_

_x0010__x0010_

_x0010__x0016__x0012__x0016__x0019__x0016__x0010__x0012__x0012__x0016__x0010__x0010
__x0019__x0010__x0010__x0016__x0012__x0010_

_x0010_

_x0010_

_x0007_

_x0010_

_x0007__x0007_

_x0007_

_x0007_ _x0007_

_x0007__x0007_
_x0007__x0007_ _x0007__x0007_
_x0007__x0007__x0007__x0007__x0007__x0007_

_x0007__x0007_ _x0007_ _x0007__x0007_ _x0007_ _x0007_
_x0007_ _x0007_ _x000
7_ _x0007__x0007__x0007__x0007__x0007_
_x0007_

_x0007_

_x0010_

_x0010_

_x0010_
_x0016_

_x0010_

_x0012__x0007_

_x0010_

_x0010_

_x0012__x0010__x0010__x0012_

_x0016__x001D_-_x0016__x0016_

_x0012__x0016__x0016__x0010__x0016_
_x0016__x0010__x0012_
_x0010_

_x0012_

_x0010_

_x0010__x0012_

_x0012_

_x0010_

_x0012_

_x0007_

_x0007_
_x0007_
_x0007__x0007_

_x0007_

_x0007__x0007_

_x0007_ _x0007_

_x0016__x0007_ _x0007_ _x0007__x0007_ _x0007_ _x0007_ _x0007_
_x0007__x0007_ _x0007_ _x0007__x0007_
_x0007_ _x0007__x0007_ _x0007__x0007_

_x0007__x0007__x0007__x0007__x0007__x0007_

_x0007__x0007__x0007_

_x0007_

_x0010__x0007_

_x0012_

_x0012__x0010__x0012_
_x0010_

_x0016_

_x0012_

_x0010_

_x0010__x0010_

_x0010_

_x0010_

_x0010_

_x0010_

_x0010__x0012__x0012_

_x0012_

_x0012__x0016_

_x001D__x001D__x0010__x0016__x0019__x0016__x0010__x0010__x0016__x0010__x0016__x0012__x0016__x0012__x0010__x0010__x0012__x0012__x0019__x0012_

_x0012_

_x0010_

_x0007__x0010_

_x0007_

_x0007_

_x0010_

_x0007_

_x0007__x0007_

_x0007__x0007__x0007__x0007__x0007__x0007_ _x0007__x0007_

_x0010__x0007__x0007__x0007__x0007_ _x0007_ _x0007_ _x0007_
_x0007_
_x0007__x0007__x0007_
_x0007__x0007_
_x0007_

_x0007_

_x0007_ _x0007_

_x0007_

_x0010_

_x0010__x0010__x0010__x0012__x0010_

_x0010_

_x0010_

_x0012__x0010_

_x0012__x0010_

_x0010__x0010_

-_x0019__x0019__x0012__x0010_

_x0010__x0019__x0010__x0012__x0016_

_x0010__x0012__x0016__x0019__x0019__x0012_

_x0010_

_x0012_

_x0007_

_x0010_

_x0007_

_x0007_
_x0007_

_x0007__x0007__x0007__x0007__x0007__x0007__x0007__x0007__x0007_

_x0007_ _x0007__x0007__x0007__x0007_ _x0007_ _x0007__x0007_ _x0007_
_x0007_ _x0007_ _x0007_
_x0007__x0007_
_x0007__x0007_
_x0007_ _x0007__x0007_

_x0007_
_x0007_ _x0007_

_x0007_
_x0010_

_x0007_

_x0007_

_x0010__x0010_

_x0010_

_x0012__x0010_

_x0010__x0010__x0010__x0012__x0012_

_x0010__x0012_

_x0016__x0010__x0010__x0010_

_x0010__x0010__x0010__x0012__x0010_

_x0016__x0010__x0010__x0010__x0010__x0010__x0010_

_x0019__x0016__x0016__x0016_-_x0016_

_x0019_

_x0010__x0012__x0010__x0010__x0016_

_x0012_

_x0010__x0019_

_x0010__x0010_

_x0019__x0010__x0010_

_x0007_

_x0007_

_x0007_

_x0007_ _x0007_

_x0007_
_x0007__x0007_
_x0007__x0007_

_x0007_ _x0007_ _x0007__x0007_
_x0007_ _x0007_ _x0
007_ _x0007__x0007_
_x0007_

_x0007__x0007_
_x0007__x0007_

_x0007__x0007_
_x0007_

_x0007__x0007__x0007__x0007_

_x0007_

_x0007_

_x0007_

_x0010_

_x0012__x0010_

_x0010_

_x0010_

_x0010_

_x0016_

_x0010__x0012_

_x0019__x0010_

_x0012_

_x0019__x0012__x0019__x001D__x001D__x0019_
_x0012__x0016__x0012_

_x0012__x0012__x0012__x0016__x0012_

_x0010__x0012_

_x0012__x0010__x0019__x0010__x0012_
_x0010__x0012_

_x0012_-_x0010_

_x0010_

_x0007_

_x0012__x0010_

_x0007_

_x0007_

_x0007_

_x0007__x0007__x0007_
_x0007__x0007__x0007__x0007_
_x0007_
_x0007_

_x0007_ _x0007_ _x0007_
_x0007_ _x0007__x0007_ _x0007__x0007_

_x0007__x0007_
_x0007__x0007_

_x0007_

_x0010_

_x0012_ _x0007_
_x0010_

_x0010_

_x0010_

_x0010_

_x0012_

_x0010_

_x0010_

_x0012_

_x0010__x0010__x0010__x0010__x0010__x0010_

_x0012_

_x0010__x0016_

_x0016__x0016_

_x0012_

_x0019__x0010__x0016_

_x0012_

_x0010_

_x0010__x0010_

_x0010_

_x0010_

_x0010_

_x0010__x0012_

_x0012_

_x0010_

_x0010_

_x0007__x0007_

_x0007__x0007__x0007_
_x0007__x0007_

_x0007_ _x0007_

_x0007_ _x0007_

_x0007__x0007__x0007_ _x0007_ _x0007_ _x0007_
_x0007_ _x0007_
_x0007_
_x0007__x0007__x0007__x0007__x0007__x0007_

_x0007_
_x0007__x0007_

_x0007__x0007__x0007_ _x0007_

_x0007__x0007_

_x0012_

_x0010_

_x0012_

_x0010_

_x0010__x0010_

_x0012__x0010__x0010__x0010_
_x0010__x0016__x0012__x0012_

-_x0019__x0012__x0012__x0010__x0012__x0012__x0016__x0012__x0012_

_x0010_

_x0012__x0010_

_x0010_

_x0010_

_x0012_

_x0016__x0010_

_x0012_

_x0007_

_x0007__x0007_

_x0007_

_x0007_

_x0007__x0007__x0007_

_x0007__x0007_
_x0007__x0007__x0007_ _x0007_ _x0007__x0007_ _x0007_
_x0007_ _x0007_ _x0007_
_x0007__x0007_
_x0007__x0007_ _x0007_ _x0007_
_x0007_

_x0007_

_x0007_ _x0007_

_x0007_

_x0007_

_x0012_

_x0010__x0010_

_x0010_

_x0010_

_x0010__x0012_

_x0010__x0012__x0010__x0012_

_x0010__x0012__x0016__x0012__x0019__x0016_
_x0012__x0016__x0016__x0012__x0010__x0010__x0016_

_x0012_

_x0012_

_x0010__x0010__x0016_

_x0010__x0010_

_x0010__x0010_

_x0007_

_x0010_

_x0007_

_x0007__x0007_

_x0007_

_x0007__x0007__x0007_ _x0007__x0007__x0007_

_x0007_ _x0007__x0007_ _x0007_ _x0007_ _x0007__x0007_ _x0007_

_x0007_ _x0007_ _x0007_ _x0007_ _x0007_
_x0007__x0007__x0007_ _x0007_ _x0007__x0007_

_x0007_

_x0007__x0007__x0007__x0007_

_x0007_

_x0007_

_x0012_

_x0012_
_x0010_

_x0010_

_x0012_

_x0010_
_x0010_

_x0012_

_x0016__x0010_

_x0010__x0010__x0012__x0010__x0016__x0012__x0016_"+&_x0016__x0016__x0019__x0012__x0
012__x0016__x0016__x0010__x0016__x0016__x0010__x0016_

_x0010_

_x0007_

_x0012_

_x0007_
_x0007_

_x0007_

_x0007_
_x0007__x0007_ _x0007_
_x0007__x0007_ _x0007__x0007__x0007__x0007_
_x0007_ _x0007_ _x0007_ _x0007_ _x0007_

_x0007_ _x0007__x0007_
_x0007_ _x0007_ _x0007_ _x0
007__x0007_ _x0007_ _x0007__x0007_
_x0007__x0007_ _x0007__x0007_

_x0007__x0007__x0007_

_x0007_

_x0010_

_x0012__x0012_

_x0010__x0012_
_x0010_

_x0012__x0012_

_x0010_

_x0012_

_x0016__x0016__x0010__x0016__x001D_$.'_x0019__x0012__x0016__x0010__x0016__x0012__x00
10__x0012_

_x0016__x0016__x0012__x0012__x0016_

_x0010__x0010_

_x0010__x0016_

_x0012_

_x0012__x0010_

_x0010_
_x0010_

_x0007_

_x0007_

_x0010_

_x0010_

_x0007__x0010_

_x0007_
_x0007__x0007__x0007_

_x0007_ _x0007__x0007__x0007_ _x0007_ _x0007_ _x0016_-
_x0007_

_x0007__x0007__x0007__x0007__x0007_
_x0007__x0007_

_x0007_
_x0007__x0007_

_x0007_

_x0010_

_x0010__x0010_

_x0012_

_x0016__x0016__x0016_

_x001D__x0016__x0019_%3"!_x0016__x0016__x0016__x0016__x0012_

_x0016__x0016_
_x0016__x0012__x0016_

_x0010_

_x0012_

_x0010_

_x0010__x0010__x0010_

_x0010_

_x0007_

_x0007_

_x0007_

_x0007_
_x0007_

_x0007__x0007_
_x0007__x0007_

_x0007_

_x0007__x0007_ _x0007_ _x0007_ _x0007_ _x0007_ _x0007_

_x0007_ _x0007_
_x0010_

_x0007_ _x0007_
_x0007_
_x0007_ _x0007__x0007_
_x0007_
_x0007__x0007__x0007__x0007__x0007_

_x0007_

_x0007__x0007_

_x0007_

_x0007_

_x0010_

_x0010__x0010_

_x0010__x0012__x0012__x0010_

_x0012__x0010_

_x0012__x0012_
_x0012__x0012_

_x0012_
_x0010__x0012__x0012_

_x0012__x0016_

_x0012__x001D_)% _x0016__x0019__x001D__x0019__x0019_

_x0012__x0016__x0010__x0012__x0010__x0012_

_x0010_

_x0010_

_x0012__x0010__x0010_

_x0012_

_x0010_

_x0010_

_x0007_

_x0007_

_x0007_

_x0007__x0007__x0007__x0007__x0007_

_x0007__x0007__x0007__x0007_

_x0007__x0007_

_x0007_
_x0007_

_x0007_ _x0007_ _x0007_
_x0007_ _x0007__x0007__x0007_ _x0007__x0007__x0007_ _x0007__x
0007_ _x0007_

_x0007_ _x0007_

_x0007_

_x0007_
_x0007_

_x0010__x0010_

_x0012__x0012_

_x0010_

_x0012_

_x0010_

_x0010_

_x0010_

_x0010_

_x0012__x0012__x0012__x0010__x0010__x0010_

_x0012__x0010_

_x0016_

_x0016__x0016__x0016__x001D__x0019_--_x0019__x0019__x0016__x0012__x0016__x0019_

_x0012__x0010_
_x0019__x0012_

_x0010__x0010_

_x0012_

_x0010_

_x0010_

_x0012_

_x0007_

_x0010_

_x0007_

_x0007_
_x0007_

_x0007_
_x0007_
_x0007__x0007__x0007__x0007_ _x0007__x0007__x0007_
_x0007__x0007_ _x0007_ _x0007__x0007_
_x0007_ _x0007_ _x0007_ _x0007_ _x0007_
_x0007_ _x0007__x0007_ _x0007__x0007_ _x0007_

_x0007__x0007_

_x0007_

_x0012_

_x0010__x0010__x0012_

_x0010__x0012__x0010__x0012_

_x0010_

_x0010__x0010__x0012__x0012_

_x0010_

_x0012__x0012__x0012__x0016__x0016__x0019_-_x001D__x001D__x0019__x001D_--_x0016__x0019__x0019_

_x0012__x0016__x0010__x0010__x0012__x0016_

_x0012_

_x0010_

_x0012_

_x0010__x0010_

_x0010_

_x0010_

_x0007_
_x0007_

_x0007__x0007_ _x0007_ _x0007__x0007_ _x0007_ _x0007_ _x0007_
_x0007_ _x0007_ _x0007_
_x0007_ _x0007_ _x0007_ _x0007_ _x0007_
_x0007_
_x0007_

_x0007__x0007_

_x0007_

_x0012_

_x0010_

_x0016__x0010__x0012_

_x0012_

_x0016__x0019__x0012__x0016__x0012_

_x0010__x0012__x0010__x0016__x0016__x0010__x001D__x001D__x0019_!_x0019__x001D__x001
9__x0010__x0012__x0019__x0019__x0016__x0016__x0010_

_x0010__x0012_
_x0010__x0012_

_x0010_

_x0016_

_x0012_

_x0007_

_x0007_

_x0007_
_x0007_ _x0007_ _x0007__x0007_
_x0007_

_x0007__x0007__x0007__x0007_
_x0007_
_x0007_ _x0007_ _x0007_ _x00
07_ _x0007__x0007__x0007_ _x0007_
_x0007__x0007__x0007__x0007__x0007_

_x0007_

_x0007_
_x0007__x0007_ _x0007_ _x0007_

_x0007_

_x0007_

_x0010_

_x0010_

_x0010_

_x0012_

_x0010__x0010__x0010__x0010_

_x0010_

_x0010_

_x0010__x0016__x0012__x0016__x0016__x0012__x0012__x0016__x0016__x0012__x0019_!_x0010
__x0016_-_x001D__x0016__x001D_
_x0019__x001D__x0016__x0019__x0016__x0012__x0010__x0016_

_x0012__x0010__x0010_

_x0012__x0012__x0012_

_x0010_

_x0010_

_x0010__x0010_

_x0012_

_x0007__x0010_

_x0010_

_x0007_
_x0007_
_x0007_
_x0007_ _x0007_ _x0007_
_x0007__x0007_ _x0007__x0007_ _x0007_
_x0007_ _x0007_
_x0007__x0007__x0007__x0007_

_x0007__x0007_
_x0007__x0007__x0007__x0007_
_x0007_

_x0010_

_x0010__x0010_

_x0010__x0010_

_x0012_

_x0012_
_x0012__x0016__x0012_

_x0019_

_x0016__x0016__x0012__x0012__x0010__x0016__x0019__x0016__x0016__x001D_--_x0016__x0010__x0019_

_x0012__x0019__x0016__x0016_
_x0016__x0012__x0010_

_x0012_

_x0012_
_x0012_

_x0010__x0010__x0010_

_x0010__x0012_

_x0012_

_x0010_

_x0007_

_x0007__x0007__x0007_
_x0007_

_x0007_

_x0007_ _x0007__x0007_
_x0007_ _x0007__x0007_
_x0007__x0007__x0007_ _x0007_
_x0007__x0007_ _x0007__x0007_ _x0007_ _x0007__x0007__x0007__x0007_

_x0007_

_x0007__x0007_

_x0007_ _x0007_

_x0010_

_x0010__x0010_

_x0010__x0010_

_x0010_

_x0010_

_x0010__x0019__x0010_

_x0012__x0012__x0016__x0012__x0016__x0019_

_x0010__x0016_ _x0019__x0019_-_x0019__x0016__x0019__x0019_

_x0012__x0012__x0016_

_x0010__x0010__x0012_

_x0012_

_x0010__x0012__x0010_

_x0012_

_x0010_

_x0007_
_x0007_ _x0007__x0007_
_x0007__x0007__x0007_
_x0007_
_x0007_
_x0007_ _x0010__x0007__x0007_ _x0007__x0007_

_x0007_

_x0007_

_x0007_

_x0010_

_x0010__x0010_

_x0010__x0012__x0016_

_x0012__x0016__x0019_

_x0010__x0012__x0012__x0019_

_x0010_

_x0016__x0016_-_x0019__x0016__x0016__x0019__x0016__x0016__x0019_

_x0012_

_x0012__x0016_

_x0012__x0019_

_x0012_
_x0010_

_x0010_

_x0012_

_x0010_

_x0007_

_x0010__x0007_

_x0007_

_x0007_

_x0007_
_x0007__x0007__x0007__x0007_
_x0007__x0007_
_x0007__x0007_
_x0007__x0007__x0007__x0007__x0007__x0007__x0007_ _x0007_ _x0007__x0007_

_x0007__x0007_ _x0007_
_x0007__x0007_ _x0007__x0007_ _x0007__x0007_

_x0007__x0007_

_x0007_
_x0007_

_x0007__x0007_

_x0012_

_x0007_

_x0010__x0010_

_x0010__x0010_

_x0012_

_x0010_

_x0016_

_x0010_

_x0010_

_x0012__x0016_

_x0012_

_x0012_
_x0010__x0012__x0016_

_x0016__x0016__x0016__x0012__x001D_"-"_x001D__x0012__x0019__x0012__x0016__x0016_

_x0012__x0016_

_x0016__x0012__x0010_

_x0016__x0016__x0012__x0016__x0012__x0012__x0010_

_x0010_

_x0012__x0010_

_x0012_

_x0012_

_x0007_

_x0010__x0012_

_x0007_

_x0007__x0007_
_x0007_

_x0007__x0007__x0007__x0007__x0007__x0007__x0007__x0007_
_x0007_ _x0007__x0007_ _x0007_ _x0007_ _x0007_

_x0007_

_x0007__x0007_
_x0007_

_x0007__x0007__x0007__x0007_

_x0007_ _x0007_ _x0007_

_x0007_

_x0010_

_x0007_
_x0010__x0010_
_x0010_

_x0012_

_x0010__x0016_

_x0012_

_x0016_

_x0012__x0016__x0019__x0012__x0016__x0010__x0012_

_x0019_-_x0019_-_x0012__x001D__x0016__x0019__x0016__x0012__x0012__x0010__x0016__x0012__x0016__x0010__x0010_

_x0010_

_x0012_

_x0010__x0016__x0012_

_x0012_ _x0012__x0012_

_x0012_

_x0010_
_x0012_

_x0007__x0007__x0007__x0007_ _x0007_ _x0007__x0007__x0007__x0007_
_x0007__x0007__x0007__x0007_ _x0007_ _x0007_ _x0007_ _x0007_
_x0007_ _x0007_ _x0007_ _x0007_
_x0007_ _x0007_
_x0010_

_x0007_

_x0007__x0007_
_x0007_

_x0007__x0007_

_x0010_

_x0010__x0012__x0010_

_x0010__x0010_

_x0012__x0016__x0012__x0012__x0012__x0010__x0012_

_x0012__x0016__x0012__x0010__x0012_

_x0012__x0016__x0012__x0016__x0012_-_x0019_
_x001D__x0016__x0019__x0019__x001D__x0016__x0016_

_x0012__x0016__x0016__x0019_

_x0012__x0010_
_x0010__x0012_

_x0012_

_x0010_

_x0010_

_x0012_

_x0007_

_x0007_

_x0010_

_x0010_

_x0007_

_x0007_

_x0007_

_x0007_
_x0007_

_x0007__x0007__x0007_

_x0007_

_x0007__x0007__x0007_

_x0007__x0007__x0007_ _x0007_ _x0007_ _x0007__x0007_ _x0007_

_x0007__x0007_ _x0007_ _x0007_

_x0007_ _x0007_

_x0007__x0007__x0007__x0007_

_x0007_

_x0007__x0007__x0007_

_x0007__x0010_

_x0007_

_x0007_

_x0016__x0012_

_x0012__x0012__x0012_

_x0010_

_x0012_
_x0010__x0012_

_x0012_

_x0010_

_x0010_

_x0007_

_x0007_

_x0007__x0007_ _x0007_ _x0007__x0007_ _x0007__x0007_ _x0007_
_x0007__x0007_ _x0007_
_x0007__x0007__x0007_ _x0007__x0007__x0007__x0007__x0007__x0007__x0007_
_x0007_ _x0007_ _x0007_
_x0007_ _x0007_ _x0007_

_x0007__x0007_
_x0007_
_x0007__x0007__x0007__x0007_ _x0007_

_x0007_

_x0007_

_x0007__x0007_

_x0012_

_x0010__x0007_

_x0012__x0010_

_x0010_

_x0012_
_x0010_

_x0016_

_x0010_

_x0012__x0016_

_x0010__x0012__x0016__x0016__x0012__x0019__x0019__x0019__x0019__x0019__x0016_!_x0019
__x0019__x0012_-_x001D__x0019__x0019_-_x0016__x0019_

_x0012__x0016__x0012__x0012_

_x0010_

_x0016_
_x0010_

_x0012_

_x0010_

_x0010_

_x0012__x0010__x0010_
_x0010__x0010_

_x0010_

_x0010__x0010_

_x0010__x0016_

_x0012_

_x0012__x0012__x0016__x0012__x0016_

_x0010__x0012__x0016__x0012__x0016__x0019__x001D__x001D__x0019_-_x0016__x0019_

_x0010__x0016_

_x0012__x0010__x0012__x0012__x0010_

_x0016_

_x0010_

_x0010_

_x0010_

_x0010_

_x0010_

_x0010_

_x0007__x0007_

_x0007_

_x0007_

_x0007__x0007__x0007__x0007__x0007__x0007_
_x0007_
_x0007__x0007__x0007_ _x0007__x0007__x0007_ _x0007_ _x0007_
_x0007__x0007__x0007_ _x0007_ _x0007_
_x0007_ _x0007__x0007_ _x0007__x0007__x0007__x0007_
_x0007__x0007_

_x0007__x0007__x0007_

_x0007_

_x0007_

_x0010_

_x0010_

_x0012_

_x0010_

_x0010__x0010__x0016_

_x0010__x0012_

_x0010_

_x0012__x0016__x0012__x0016_

_x0012__x0019__x0019__x0010__x0019__x0019__x0016_--_x0012__x0010_

_x0010_

_x0010_

_x0007_

_x0010_

_x0010_

_x0010_

_x0007_

_x0007__x0007__x0007_

_x0007_

_x0007_ _x0007__x0007__x0007_ _x0007_ _x0007_ _x0007_ _x0007_ _x0007_
_x0007_ _x0007_ _x0007_
_x0007_ _x0007_
_x0007__x0007_
_x0007__x0007_ _x0007__x0007_ _x0007_
_x0007_

_x0012_

_x0010_

_x0010__x0016_

_x0012_

_x0012__x0016__x0010_

_x0012__x0010__x0012_

_x0016__x0019__x001D__x0016__x0016_--_x0016_-_x0019_

_x0012__x0016__x0010__x0012_

_x0012__x0012_

_x0016_

_x0016_

_x0010_

_x0010_

_x0007__x0007_

_x0007_

_x0012_

_x0007_ _x0007_

_x0007__x0007__x0007__x0007__x0007__x0007_ _x0007_
_x0007_ _x0007__x0007__x0007__x0007__x0007_

_x0007_

_x0007_ _x0007_

_x0007_ _x0007__x0007__x0007__x0007__x0007__x0007__x0007_ _x0007_

_x0007_ _x0007__x0007__x0007__x0007_

_x0007_ _x0007_

_x0007_

_x0007_
_x0007__x0007_

_x0010_

_x0012_

_x0010_

_x0010_

_x0012_

_x0012_

_x0012_

_x0010_
_x0012_

_x0016__x0010__x0012__x0012_

_x0012__x0010_

_x0019__x0016__x0016__x0010__x0019__x0019__x0012__x0019_

--_x0019__x0019__x0016__x0016__x0019__x0012__x0019__x0010__x0010__x0010_

_x0016__x0016__x0010__x0019__x0019__x0010__x0012_

_x0010_

_x0010_

_x0012_

_x0010_

_x0010_
_x0010_

_x0010_

_x0007_

_x0007_

_x0007__x0007__x0007_
_x0007__x0007_

_x0007_ _x0007__x0007_
_x0007_ _x0007__x0007_ _x0007__x0007_ _x0007_
_x0007_
_x0007_ _x0007_ _x0007_ _x0007_
_x0007_
_x0007_ _x0007__x0007_ _x0007_

_x0007_
_x0007_

_x0007_
_x0010_

_x0007_

_x0012_

_x0010_

_x0012_
_x0010_

_x0012_ _x0010__x0010_

_x0012__x0016__x0010__x0012__x0016__x0012__x0010__x0016_

_x0010__x001D__x001D_
_x001D__x0010__x0016__x0016__x0012__x001D__x0019__x0019__x0019__x0019__x0016__x0012
_
_x0012_

_x0019__x0012_

_x0010__x0016__x0016__x0016__x0019__x0012__x0012_

_x0010_
_x0012_

_x0012_

_x0010_

_x0010_

_x0012__x0012_

_x0007_
_x0007_

_x0007_ _x0007__x0007_

_x0007_

_x0007_ _x0007__x0007__x0007_
_x0007_

_x0007__x0007_
_x0007__x0007__x0007__x0007__x0007__x0007__x0007_ _x0007_
_x0010_ _x0007__x0007_ _x0007__x0007_ _x0007_
_x0007__x0007_ _x0007__x0007_ _x0007_ _x0007__x0007_ _x0007__x0007__x0007_

_x0007_

_x0007_

_x0007__x0007_

_x0007_

_x0010_

_x0012__x0010_

_x0010__x0012_
_x0010_

_x0010_

_x0012_

_x0016__x0012__x0012__x0010_

_x0012__x0019__x0016__x0019__x0012__x0019__x001D__x0019_"_x0019_

_x0016__x0012__x0016__x0019__x0012__x0016__x0016__x0012__x0010_

_x0012__x0012__x0010__x0012__x0010__x0010__x0016_

_x0010_

_x0010_

_x0007_
_x0010__x0007__x0007_
_x0007_

_x0007_

_x0007_ _x0007__x0007_

_x0007_ _x0007_ _x0007_
_x0007_

_x0007__x0007__x0007_ _x0007_ _x0007__x0007_
_x0007_ _x0007_ _x0007_ _x0007_ _x0007_ _x0007_
_x0007__x0007_ _x0007__x0007_

_x0007_

_x0007_
_x0007_

_x0007_

_x0010_

_x0012_

_x0010_

_x0012__x0010__x0012_

_x0010_

_x0012_

_x0012_

_x0012_

_x0010__x0012_

_x0012__x0012__x0010__x0012__x0010_

_x0019__x0016__x0019_
_x0019__x0019__x0016__x001D_!_x0016__x0016__x0019__x0016__x0016__x001D__x0019__x0019__x0010__x0016_

_x0012__x0012_

_x0012_

_x0010__x0010__x0010_
_x0012__x0012_

_x0010_

_x0012_

_x0007_

_x0007_
_x0007_ _x0007__x0007__x0007_
_x0007_

_x0007__x0007__x0007__x0007__x0007__x0007__x0007__x0007__x0007__x0007_
_x0007__x0007_
_x0007_
_x0007_ _x0007__x0007_
_x0007__x0007__x0007__x0007__x0007__x0007__x0007__x0007_

_x0010__x0007_

_x0010_

_x0010_

_x0012__x0010_

_x0010__x0010__x0010__x001D__x0010_

_x0010__x0010_

_x0010__x0019_

_x0012__x0016__x0012__x0019__x0016__x0010_-!_x0016_-_x0019__x0019__x001D__x0019__x0019__x0010__x0019__x0016__x0016__x0010__x0019__x0016_

_x0010__x0016__x0016__x0012_

_x0016__x0010__x0010__x0016__x0012_

_x0010__x0010_

_x0010_

_x0010__x0010_

_x0007_
_x0010_

_x0010__x0007_

_x0007__x0007_ _x0007_ _x0007__x0007_ _x0007_

_x0007_
_x0007__x0007_ _x0007__x0012_
_x0007__x0007__x0007__x0007_
_x0007__x0007__x0007_

_x0007_ _x0007_
_x0007_ _x0007__x0007_ _x0007_ _x0007_ _x0007__x0007_

_x0007_

_x0007_ _x0007__x0007_

_x0010_

_x0010_

_x0012_
_x0010__x0010_

_x0012_

_x0016_

_x0010_

_x0016_

_x0016__x0012__x0012__x0016_

_x0012__x0019_-_x001D__x001D__x001D_!_x0016__x0016__x0019__x0019__x0016__x0016__x0012__x0012__x0016__x0016__x0016_

_x0012_

_x0010_

_x0010__x0010_

_x0007_

_x0007_ _x0007__x0007_
_x0007_
_x0007__x0007_
_x0007_

_x0007_
_x0007__x0007__x0007__x0007_ _x0007_ _x0
007_ _x0007_
_x0007_

_x0007_
_x0007_ _x0007_

_x0007_

_x0007_
_x0007__x0007_

_x0010_

_x0012__x0010_

_x0012__x0010__x0012__x0016__x0016_-_x0016_

_x0016_

_x0016__x0016__x0016__x001D__x001D__x001D__x0016__x0019__x0012__x0016__x0010__x0010__x0010__x0016__x0016__x0010__x0012__x0012__x0010_

_x0012_

_x0010_
_x0010__x0010_

_x0007__x0007_
_x0007_

_x0007__x0007_
_x0007_

_x0007_

_x0007_
_x0007_

_x0007_ _x0007__x0007__x0007_ _x0007__x0007_
_x0007_ _x0007__x0007__x0007__x0007__x0007_ _x0007_
_x0007__x0007__x0007_ _x0007_ _x0007__x0007_

_x0007_ _x0007__x0007__x0007__x0007__x0007_
_x0007__x0007__x0007_

_x0010__x0007_
_x0007_
_x0007_

_x0010_

_x0007_

_x0012_

_x0010_

_x0010__x0010_

_x0016__x0012_

_x0010_

_x0012_

_x0016__x0019__x0019__x0019__x0016__x0016__x0019__x0016__x0019__x0016__x0016__x0019
_-_x001D_&_x0019_

_x0012__x0016__x0012__x0012_
_x0012__x0010__x0010__x0010__x0010__x0010_

_x0010_
_x0010_

_x0010_

_x0007_

_x0007__x0007_

_x0007_

_x0007__x0007_

_x0007__x0007__x0007__x0007_
_x0007_

_x0007_

_x0007__x0007_
_x0007__x0007__x0007_ _x0007__x0007_ _x0007_
_x0007_ _x0007__x0007__x0007_
_x0007__x0007_ _x0007_ _x0007__x0007__x0007__x0007__x0007__x0007__x0007_
_x0007_ _x0007__x0007__x0007__x0007_
_x0007_

_x0010_

_x0012_
_x0012__x0012__x0010_

_x0010__x0012_

_x0012__x0019__x0016__x0019__x0019__x001D__x001D__x0019__x0019__x0016__x0016__x0019
__x001D_" _x001D__x0016_-_x0019__x0016__x0019__x0019__x0016__x0016__x0019_-_x0019_

_x0016__x0012__x0012__x0012_

_x0010_

_x0012_

_x0012_

_x0010_

_x0010_

_x0010_

_x0010_

_x0007__x0007_
_x0007__x0012_

_x0007__x0007_

_x0007_

_x0007_
_x0007_

_x0007__x0007_

_x0007_ _x0007_ _x0007__x0007_ _x0007_ _x0007_
_x0007_ _x0007_ _x0007_ _x0007_ _x0007_ _x0007_ _x0007_
_x0007_ _x0007_ _x0007_ _x0007_
_x0007_ _x0007__x0007_ _x0007__x0007_
_x0007__x0007__x0007_
_x0007__x0007__x0007__x0007__x0007__x0007__x0007_ _x0007_
_x0007_
_x0007_

_x0010_

_x0010_

_x0010_

_x0019__x0012__x0012__x0010__x0010__x0012__x0016__x0016_

_x0012__x0019__x0016__x0019__x0016__x0019__x0019__x0016__x0016__x0019__x0019__x001D_ _x0019__x001D_#-_x0019__x0019__x0019__x0019__x0019__x0019__x001D__x0019_

_x001D_-_x001D__x0016_

_x0016__x0010__x0012_

_x0010__x0010_

_x0012_

_x0012_

_x0010_

_x0007_
_x0010_

_x0007_

_x0007__x0007_ _x0007_

_x0007_

_x0007_
_x0007_ _x0007_

_x0007__x0007_
_x0007__x0007_ _x0007_ _x0007__x0007_
_x0007_ _x0007_ _x0007__x0007_ _x0007_
_x0007__x0007_ _x0007_
_x0007__x0007_
_x0007__x0007__x0007_

_x0007_

_x0007_

_x0007_

_x0007__x0010_

_x0007_

_x0010__x0010_

_x0010_

_x0010_
_x0010__x0010_

_x0010_

_x0012_

_x0016__x0016_

_x0016__x0019__x0010_

_x0012__x0012__x0019__x0016__x0010__x0016__x0012__x0016_-_x0016_ _x001D_
_x0019__x0019__x0019_!_x0016__x0016__x0019__x0010__x0012__x0012__x0019__x0010__x0010
__x0010__x0019_

_x0010_

_x0010_

_x0007_

_x0007_

_x0007_

_x0007_

_x0007__x0007_

_x0007__x0007_

_x0007_ _x0007__x0007_ _x0007__x0007__x0007_

_x0007__x0007__x0007__x0007__x0007__x0007__x0007__x0007__x0007__x0007__x0007__x0007
__x0007_ _x0007_
_x0007_ _x0007__x0007__x0007__x0007_

_x0007__x0007_

_x0007_

_x0007_

_x0010_

_x0010_
_x0007_

_x0010__x0012__x0010__x0010_

_x0012_

_x0016__x0012__x0019__x0019__x0019__x0019__x0012__x0019__x0016__x0016__x0016__x001D
_-_x0019__x0016__x001D_ _x0012__x0016__x0012__x0016__x0012_

_x0010__x0010__x0012_

_x0007_

_x0007_

_x0007_

_x0007_

_x0007_

_x0007_
_x0007__x0007__x0007__x0007__x0007_

_x0007__x0007_ _x0007__x0007__x0007__x0007_ _x0007__x0007__x0007_
_x0007_ _x0007_ _x0007__x0007_ _x0007_
_x0007_ _x0007__x0007__x0007_ _x0007_

_x0007_

_x0007_ _x0007__x0007_
_x0007_

_x0007_

"

_x0010_

_x0010_

_x0010_

_x0010__x0010__x0012__x0012__x0010__x0012_

_x0012_

_x0010_
_x0010__x0016__x0019__x0012__x0012__x0012__x0016__x0016__x0019__x0012_
_x0019__x0019__x0019_ !-_x0016__x0012__x0012__x0012__x0016__x0016__x0012_
_x0012__x0010__x0010__x0012_

_x0010_

_x0007_

_x0007_

_x0007__x0007_ _x0007_

_x0007_ _x0007_

_x0007_ _x0007__x0007_

_x0007_
_x0007__x0007__x0007__x0007_
_x0007_ _x0007__x0007__x0007_
_x0007_ _x0007__x0007_ _x0007__x0007_ _x0007_
_x0007_ _x0007_ _x0007_ _x0007_ _x0007_
_x0007_ _x0007__x0007__x0007__x0007_
_x0007_ _x0007__x0007_

_x0007_

_x0016_#%

_x0010_

_x0012__x0010__x0016_

_x0010_

_x0010_
_x0012_

_x0010_
_x0012_

_x0012__x0016__x0012__x0010__x0016__x0016__x0010__x0016__x0019_!-_x0019__x0016__x0019_-
_x0019__x0019__x0019_
_x0016__x0019__x0012__x0012__x0016__x0016__x0019__x0016__x0016_

_x0010__x0012__x0016_

_x0010_

_x0012__x0010_

_x0012__x0010_

_x0010_ _x0007_

_x0007__x0007_

_x0007__x0007_

_x0007__x0007__x0007__x0007__x0007__x0007_ _x0007_
_x0007_
_x0007__x0007__x0007_ _x0007_
_x0007_ _x0007_ _x0007_
_x0007_ _x0007_ _x0007__x0007_ _x0007__x0007__x0007_

_x0007_ _x0007_
_x0007_
_x0007_
_x0007_

_x0007_

_x0007_

_x0019__x0019_

_x0012_

_x0010_

_x0010_

_x0010_

_x0012_

_x0010__x0012__x0016_
_x0012__x001D_

_x0019__x001D__x0016__x0016__x0016__x001D_--_x0016_-#$_x0019__x0016__x001D__x0012__
x0016__x001D__x0012_

_x0012_

_x0010__x0012__x0010__x0010__x0012_

_x0012_

_x0010_

_x0007_

_x0007_

_x0007_

_x0007_ _x0007__x0007_

_x0007__x0007__x0007__x0007_

_x0007__x0007__x0007_ _x0007_ _x0007__x0007_ _x0007_ _x0007_

_x0007_ _x0007_ _x0007__x0007__x0007_

_x0007_ _x0007_ _x0007_ _x0007__x0007__x0007_

_x0007_
_x0007_ _x0007_

_x0010_

_x0010__x0010__x0012_

_x0010__x0012_

_x0012__x0010_

_x0010_

_x0010__x0012__x0010__x0012_

_x0016__x0016__x0019__x0016__x0016__x0012__x0012__x0012__x0016__x001D_-_x0016_-_x001D_+! _x0019__x001D__x0019_-_x0010__x0012__x0010_

_x0016_

_x0016__x0012_

_x0010__x0016__x0010__x0012_
_x0012_

_x0010_

_x0012_

_x0010_

_x0007_

_x0007_

_x0007_

_x0007_

_x0007_

_x0007__x0007__x0007_

_x0007_ _x0007__x0007__x0007_ _x0007__x0007_
_x0007_ _x0007__x0007
__x0007__x0007__x0007_

_x0007__x0007_

_x0007_

_x0007_

_x0010_

_x0010_

_x0010__x0012_

_x0012_

_x0012_

_x0007__x0010__x0012__x0016__x0012_

_x0016__x0010_

_x0019__x001D__x0019_-_x0016__x001D__x001D__x001D__x0016__x0019__x0019__x001D_!$$_x001D__x0016__x0019_-_x0019__x0012__x0019_

_x0012__x0012_

_x0010_
_x0012_

_x0012_

_x0010_

_x0010_
_x0010_

_x0010_

_x0010_

_x0010_

_x0007_

_x0007_
_x0007__x0007_

_x0007_ _x0007_

_x0007_
_x0007_
_x0007__x0007__x0007_

_x0007_
_x0007_ _x0007__x0007_ _x0007_
_x0007__x0007_ _x0007_ _x0012_ _x0007__x0007_
_x0007_
_x0007__x0007_
_x0007__x0007_ _x0007_
_x0007_

_x0007_

_x0007__x0007_

_x0007_

_x0012__x0007_

_x0010_

_x0012_

_x0012__x0010__x0012_

_x0012_

_x0012__x0012__x0012_

_x0019_-_x0019__x0016__x0016__x001D__x0012__x001D_-_x001D__x001D_-_x0016_!_x001D__x0016__x0016__x0019_ _x0012_

_x0016_

_x0016_

_x0012__x0012_

_x0010_

_x0010_

_x0007__x0007__x0007_

_x0007_

_x0007__x0007_ _x0007__x0007_ _x0007_ _x0007_ _x0007__x0007_
_x0007_

_x0010_
_x0007_ _x0007_ _x0007_
_x0007_ _x0007_ _x0007__x0007__x0007__x0007__x0007_ _x0007_

_x0007_

_x0007_
_x0007__x0007_

_x0010_

_x0012_

_x0010_
_x0010__x0010_

_x0010__x0010_

_x0010__x0012__x0012__x0010__x0010_

_x0010__x0016__x0016__x0010__x0012__x0016__x0016__x0012__x0016__x0019__x0012__x0012__x0016__x0019__x001D_-_x0019__x0016__x0019_"%_x0019__x0019__x0019_-" _x001D_-_x001D__x001D__x0019__x0016__x0016__x0016_

_x0010__x0012__x0010_
_x0010__x0010__x0010__x0012__x0010_

_x0010_

_x0010_

_x0012_

_x0010__x0012__x0010_

_x0012_

_x0007_

_x0007_
_x0007__x0007_ _x0007__x0007__x0007_
_x0007__x0007_

_x0007__x0007__x0007__x0007__x0007_

_x0007_

_x0007__x0007__x0007__x0007_ _x0007_ _x0007__x0007__x0007_
 _x0007_ _x0007_

_x0007__x0007__x0016_

_x0007__x0007_ _x0007_ _x0007_
_x0007_ _x0007__x0007__x0007__x0007_ _x0007_ _x0007_

_x0007_

_x0007_

_x0007_

_x0007__x0007_

_x0007__x0007_

_x0010_

_x0007__x0007_

_x0010_

_x0010_

_x0012_

_x0010_

_x0012_

_x0012__x0016__x0010__x0019__x0016_-_x0016__x0019__x0016__x0016__x001D__x0019_"
_x001D_! --_x0019__x0016__x0019__x0019__x0016_

_x0016__x0016__x0012__x0016__x0012_

_x0012__x0012_

_x0010_

_x0012_
_x0010_

_x0012_

_x0010_

_x0007_

_x0007__x0007_

_x0007__x0007__x0007_

_x0007_

_x0007_

_x0007_

_x0007__x0007_
_x0007_

_x0012__x0010_

_x0007_

_x0012_

_x0010__x0012__x0012_

_x0012_

_x0016__x0010__x0012__x0012__x0010__x0010__x0012__x0010__x0012__x0016__x0012__x0019__x0016__x0019__x0016_-_x0016__x001D__x001D_!"_x001D_!!"_x0019__x001D__x0019_-_x0019__x0016__x0016__x0019__x0010_

_x0010_

_x0010__x0010_

_x0012__x0010__x0012_

_x0010_

_x0012_

_x0010_

_x0010_

_x0007_

_x0007_
_x0007_ _x0007__x0007_

_x0007_

_x0007_ _x0007__x0007__x0007_ _x0007_

_x0007_ _x0007_ _x0007_

_x0007_ _x0007_ _x0007_ _x0007__
x0007_ _x0007_ _x0007_ _x0007__x0007__x0007__x0007__x0007__x0007__x0007_

_x0007__x0007__x0007_
_x0007_

_x0007__x0007_
_x0007_

_x0007_

_x0007_ _x0007_

_x0007_ _x0007_

_x0010_

_x0010_

_x0010_
_x0010_

_x0012__x0012__x0010__x0012__x0010_

_x0010__x0016__x0012__x0016__x0010__x0016_

_x0010__x0012__x0016__x0016__x0016__x0010__x0016__x0016__x001D__x001D__x0019__x0019
_- ---_x0019_--_x0019_-_x0016__x0010_
_x0010__x0012__x0012_

_x0010__x0010__x0012__x0019_
_x0010_
_x0010_

_x0010__x0012_

_x0016__x0010_

_x0010_

_x0012_

_x0010_

_x0007_

_x0007_
_x0007_

_x0007__x0007__x0007_

_x0007_

_x0007__x0007__x0007__x0007__x0007__x0007__x0007_
_x0007_ _x0007_ _x0007_
_x0007__x0007__x0007_ _x0007__x0007__x0007_
_x0007__x0007_ _x0007_
_x0007_ _x0007_

_x0007__x0007_

_x0007_

_x0007__x0007_

_x0010_

_x0012__x0012__x0012__x0010_

_x0016_

_x001D_

_x0010_

_x0016_

_x0012__x0012__x0016__x0016__x0012__x0012__x0012__x0019__x0019_-_x001D_-"_x0019__x0019__x0019__x001D__x0019__x0019__x001D__x0016__x0019__x0016__x0012_
_x0012__x0010__x0016__x0010_

_x0010_

_x0010_

_x0007_

_x0007__x0007__x0007_ _x0007_ _x0007__x0007__x0007__x0007_
_x0007_
_x0007__x0007_ _x0007_ _x0007_
_x0007_
_x0007__x0007__x0007__x0007_ _x0007__x0007__x0007_
_x0010_
_x0007_ _x0007_ _x0007_ _x0007_ _x0007_ _x0007__x0007_
_x0007_ _x0007__x0007__x0007__x0007__x0007_

_x0007_

_x0010__x0010_

_x0012_

_x0012_
_x0010_

_x0010__x0012__x0016__x0010__x0012_

_x0016__x0012__x0019__x0019__x0016__x0016__x0016__x0016_-_x0019__x0016_-_x001D__x001D__x0016_ -_x0019__x0019__x0012__x0019__x0019__x0016__x0016__x0016_

_x0019__x0012_

_x0012__x0010__x0012__x0012_

_x0010_

_x0016_

_x0012_

_x0010_
_x0010__x0010_
_x0012_

_x0007_

_x0007_

_x0007_

_x0007__x0007_
_x0007__x0007_ _x0007_ _x0007__x0007_

_x0007__x0007__x0007__x0007__x0007__x0007__x0007__x0007_ _x0007__x0007__x0007__x0007_ _x0007_ _x0007_ _x0007_ _x0007__x0007_
_x0007_ _x0007_
_x0007_
_x0007_

_x0007_

_x0007_

_x0007_

_x0007_

_x0007_

_x0007__x0007_

_x0010_

_x0010_

_x0012_

_x0010__x0012_

_x0016__x0010__x0010_

_x0016_

_x0012__x0012__x0010__x0019__x0016__x001D__x0016__x0019_-_x001D__x0019__x0016__x0016_

_x0016__x0016__x0019__x0012_

_x0010__x0016__x0012_

_x0010__x0010_

_x0012_

_x0010_

_x0007_

_x0007_
_x0007__x0007__x0007_

_x0007__x0007__x0007__x0007__x0007__x0007_ _x0007_
_x0007_ _x0007_

_x0007_ _x0007_ _x0007_ _x0007_ _x0007_ _x00
07_
_x0007_
_x0007__x0007__x0007_ _x0007_

_x0007_ _x0007_

_x0007_ _x0007_

_x0007__x0007__x0007__x0007_

_x0010__x0012__x0010_

_x0010_

_x0012_

_x0010_

_x0010_

_x0016__x0012__x0016__x0019__x0019__x0010__x0010__x0012_

_x0010_

_x0019__x0019__x0019__x0019__x0019__x0016__x0016__x0019__x001D__x0019__x0016_-_x001
2__x0016__x0012__x0012_ _x0019__x0019__x0010__x0016__x0012_

_x0012_

_x0010__x0012_

_x0012_

_x0010_

_x0012_

_x0012_

_x0007_
_x0007_

_x0007_

_x0007__x0007_

_x0007__x0007_ _x0007__x0007__x0007__x0007_
_x0007_ _x0007_ _x0007_
_x0007_ _x0007_
_x0007__x0007_
_x0007__x0007__x0007_
_x0007__x0007__x0007_

_x0010_

_x0010_

_x0010_

_x0007_

_x0012__x0012__x0016_

_x0010__x0010_

_x0010_

_x0012__x0012__x0016_
_x0012__x0016__x0012__x0010__x0010__x0019__x0019__x0016__x0019__x0012__x001D__x0010
__x0016__x0012__x0016__x0010__x0016__x0016__x0019__x0012__x0012__x0019_

_x0016__x0012_

_x0012_

_x0010__x0010__x0012_

_x0010__x0010_

_x0012__x0010_

_x0007_

_x0007__x0007_ _x0007_ _x0007__x0007_ _x0007__x0007__x0007__x0007__x0007_
_x0007_ _x0007__x0007_ _x0007_ _x0007__x0007_
_x0007__x0007__x0007_ _x0007_ _x0007__x00
07__x0007__x0007_ _x0007__x0007__x0007_
_x0007_ _x0007_
_x0007__x0007__x0007__x0007__x0007_

_x0007_

_x0010_

_x0007_

_x0010_

_x0010__x0012_

_x0010__x0010__x0012_

_x0010__x0012__x0012__x0010_

_x0016__x0012_

_x0012__x0016__x0019__x001D__x0016__x0019__x0019_-_x0016__x001D__x0019__x001D__x0010__x0016__x0012__x0012_
_x0012__x0016__x0012__x0019__x0016__x0010__x0016__x0012__x0016__x0016_
_x0012_

_x0010__x0010_

_x0012_

_x0012_

_x0010_

_x0007_

_x0007_

_x0007_

_x0007_

_x0007__x0007_

_x0007__x0007__x0007__x0007_ _x0007__x0007__x0007_ _x0007_ _x0007_ _x0007_ _x0007_ _x0007_ _x0007_
_x0007__x0007_ _x0007_ _x0007_ _x0007_

_x0007_

_x0007_

_x0007_

_x0010_

_x0012_
_x0010_
_x0010__x0010__x0012_

_x0010_

_x0012__x0010_

_x0010__x0019__x0016__x0012__x0019_ _x0019_-!_x0019__x0012_

_x0016__x0016__x0012__x0012__x0019__x0010__x0010_

_x0010__x0019__x0016__x0019__x0019__x0016__x0019__x0016__x0016__x0019__x0016__x0012
__x0010_
_x0016_

_x0010__x0010_
_x0010__x0010__x0010_

_x0012_

_x0010_

_x0007_
_x0007_ _x0007_

_x0007__x0007__x0007__x0007__x0007__x0007__x0007_
_x0007__x0007__x0007_
_x0007__x0007__x0007__x0007__x0007__x0007__x0007__x0007__x0007_ _x0007_
_x0007_ _x0007_ _x0007__x0007_
_x0007_
_x0007_ _x0007_ _x0007_

_x0007__x0007__x0007_

_x0007_

_x0007_

_x0007_

_x0012_

_x0010_

_x0010__x0012_

_x0010_

_x0010__x0012__x0012__x0012__x0016__x0016__x0016_--_x0019__x0016__x0012__x0019__x0016__x0016__x0016__x0016__x0019__x001D__x0019__x0019__x0016__x001D__x001D__x0019_-_x0016__x0012_

_x0010__x0012__x0012__x0016__x0012__x0019__x0012__x0016__x0010__x0010__x0016__x0012
__x0010__x0012_

_x0016_

_x0010_

_x0010_

_x0010_

_x0007_

_x0007_

_x0007__x0007_

_x0007_
_x0007__x0007__x0007__x0007__x0007__x0007_
_x0007__x0007__x0007__x0007__x0007__x0007__x0007_ _x0007__x0007_ _x0007__x0007_
_x0007_ _x0007_ _x0007_
_x0007_ _x0007_ _x0007_ _x0007_

_x0007__x0007__x0007__x0007__x0007__x0007__x0007_

_x0007__x0007_

_x0007_

_x0007_
_x0007_
_x0010_

_x0007_

_x0007_

_x0007_

_x0010_

_x0012__x0010_

_x0012__x0010_

_x0012__x0012_

_x0019__x0016__x0016__x0016__x0016__x0019__x0019__x0019__x0016__x0016__x0016__x0019__x0012__x0019__x0019__x0012__x0019__x0016__x0016__x001D_-_x0016__x0012_

_x0019__x0012__x0012__x0016__x0019__x001D_

_x0010__x0010__x0010_
_x0016_

_x0010__x0012_

_x0010_

_x0010_

_x0010_

_x0007_

_x0010_
_x0007_
_x0007__x0007_ _x0007__x0007_

_x0007__x0007__x0007__x0007_ _x0007__x0007__x0007_ _x0007__x0007__x0007__x0007
__x0007_
_x0007_ _x0007_ _x0007_ _x0007_ _x00
07_ _x0007_ _x0007__x0007_
_x0007__x0007__x0007__x0007_ _x0007__x0007_

_x0007_

_x0010__x0010_

_x0010__x0012_
_x0016__x0010_

_x0012_-_x0016__x001D__x0016__x0019_--_x0012__x0016__x0012__x0016__x0016__x0019_

_x0019__x0019_-_x0019__x0019__x0016__x0019__x0010__x0012__x0016__x0019__x0012__x0016
_

_x0016__x0016__x0012__x0010_

_x0010__x0012_

_x0010_

_x0010_

_x0010_

_x0012__x0012_

_x0007__x0010_

_x0007_

_x0007_
_x0007__x0007_ _x0007__x0007_

_x0007__x0007_ _x0007_ _x0007_ _x0007_ _x0007_
_x0007_ _x0007_
_x0007__x0007__x0007_
_x0007__x0007__x0007_

_x0007__x0007__x0007__x0007__x0007_
_x0007_

_x0012__x0007_

_x0007_

_x0010_

_x0010_

_x0012_
_x0010__x0012_

_x0010__x0016__x0019__x0019__x0016_

_x0010__x0010__x0010__x0019__x0016__x0016__x0016__x001D__x0012__x0019__x0012__x0010__x0016__x0016__x0010__x0012__x0012__x0012__x0016_

_x0012__x0012_

_x0016_
_x0012_

_x0010_

_x0010_

_x0010_
_x0012_

_x0010__x0007_
_x0007_
_x0007__x0007_
_x0007__x0007__x0007__x0007_
_x0007__x0007__x0007__x0007__x0007_
_x0007_ _x0007_ _x0007
__x0007__x0007__x0007__x0007_ _x0007__x0007_ _x0007__x0007__x0007_
_x0007_
_x0007_
_x0007_
_x0007_

_x0010__x0012_

_x0007_

_x0007__x0007__x0007_

_x0010_

_x0010_

_x0019__x0010__x0010__x0019__x0010__x0012_
_x0012_-_x0019__x0016__x0012_

_x0016__x0019__x0019__x0012_-_x0019__x0019__x0016__x0019__x0019__x0019__x0019_-_x001D_-_x0012__x0019__x0019__x0016__x0016__x0010__x0012__x0019__x0012__x0019__x0019_

_x0010__x0016_

_x0012__x0016_

_x0012_

_x0010_

_x0010_

_x0010_

_x0010_
_x0012_

_x0007_

_x0010_

_x0007_

_x0007_ _x0007__x0007_

_x0007_
_x0007_ _x0007__x0007__x0007__x0007_ _x0007__x0007__x0007__x0007_
_x0007_ _x0007__x0007_
_x0007_ _x0007__x0007_ _x0007__x0007__x0007_
_x0007_ _x0007__x0007__x0007__x0007_ _x0007_

_x0007__x0007_
_x0007_
_x0010_

_x0007_

_x0010__x0012__x0012_

_x0010_

_x0010__x0012__x0012__x0010__x0010__x0012__x0016_-_x0019__x001D__x0016__x0012__x001D__x0019__x0019_"-_x0016__x001D__x001D__x0016__x0016__x001D__x0016__x0019__x0016__x0019__x0012__x0019_

_x0016_

_x0016__x0010_
_x0012__x0012__x0012_

_x0010_

_x0010_

_x0010_

_x0007_

_x0010_

_x0007_

_x0007_

_x0007_ _x0007_

_x0007_ _x0007_
_x0007_ _x0007_ _x0007_ _x0007_ _x0007_

_x0007_ _x0007_ _x0007_ _x0007__x0007_

_x0007_
_x0007_

_x0007__x0007__x0007_

_x0007_

_x0007_

_x0007__x0007_ _x0007_ _x0007_

_x0012_

_x0012__x0010_

_x0010__x0012_
_x0010_

_x0012__x0012_

_x0012__x0012__x0012__x0019__x0016__x0016__x0019__x0019__x0019__x001D_
_x0019__x0019_"_x001D__x0016_"_x0016__x0016__x001D__x001D__x0019__x0019_

_x0016__x0010__x0012_

_x0010__x0012__x0012_

_x0010_

_x0010__x0010_

_x0010__x0010__x0010_

_x0010_

_x0010_

_x0010_
_x0007__x0007_
_x0007_

_x0007__x0007__x0007_ _x0007__x0007__x0007__x0007__x0007__x0007__x0007__x0007_

_x0007__x0007_ _x0007_
_x0007_ _x0007_ _x0007_ _x0007__x0007_
_x0007__x0007_ _x0007_

_x0007_

_x0007_
_x0007_

_x0007__x0007__x0007__x0007_ _x0007_

_x0010_
_x0012_

_x0012__x0010__x0010_

_x0016_

_x0010__x0012__x0019__x0019__x0016__x0016__x0016__x0019__x0019_-_x0016_-_x0016__x001
D__x0019__x001D_ -_x0016__x001D_ _x0016__x0016__x0016__x0019__x0019__x0010__x0012_
_x0012__x0010_

_x0010__x0010__x0010_

_x0012_

_x0010__x0010_

_x0012_

_x0012_

_x0010_

_x0012_

_x0007_

_x0010_

_x0007_ _x0007_
_x0007_

_x0007_
_x0007__x0007_
_x0007_
_x0007_

_x0007_ _x0007_ _x0007_ _x0007_ _x0007__x0007_
_x0007__x0007_
_x0007_
_x0007_ _x0007_ _x0007__x0007__x0007__x0007__x000
7_
_x0007__x0007__x0007__x0007_
_x0007__x0007_

_x0007_

_x0007_

_x0010_

_x0010__x0016__x0007_

_x0007_

_x0012_

_x0012__x0012_

_x0012_

_x0012__x0010__x0012__x0012_

_x0016__x0016__x0019__x0019__x0016__x0012__x0012__x0019__x001D_!_x001D__x001D_-_x0019__x0016__x0019_--_x0012__x0016__x0019__x0016__x0012_

_x0012__x0016_

_x0012_

_x0007_

_x0007_

_x0007__x0007__x0007_ _x0007_
_x0007__x0007__x0007_

_x0007__x0007__x0007_

_x0007__x0007_ _x0007_ _x0007__x0007_ _x0007_ _x0007__x0007_ _x0
007_ _x0007_ _x0007__x0007_

_x0007__x0007_ _x0007_

_x0007_

_x0007_

_x0012__x0019_

_x0007_
_x0012_

_x0012__x0010__x0010_

_x0010__x0010__x0012_

_x0012__x0010_

_x0016__x0016__x0019__x0019__x0016__x0019__x0016__x0019__x001D_-_x0019__x0019__x0019__x0019__x0016__x0016__x0019__x0016__x0019__x0019__x0012__x0019__x001D__x0016__x0016__x0012__x0016_

_x0016__x0012_

_x0012__x0010_

_x0010__x0007_

_x0007__x0007__x0007_
_x0007_ _x0007__x0007__x0007_ _x0007__x0007_
_x0007_ _x0007_

_x0007_ _x0007_ _x0007_

_x0007__x0007_

_x0007_
_x0007_
_x0007_ _x0007__x0007_

_x0007__x0007__x0007__x0007_

_x0016_

_x0007_

_x0007_
_x0012__x0010_

_x0016__x0012__x0016__x0016_!_x0012__x0016__x0019__x0016_%!"-_x0019__x0019__x0016_

_x0016__x0016__x0016__x0016_
_x0016__x0016_
_x0010__x0012_

_x0010_

_x0010_

_x0010_

_x0016_

_x0007_
_x0007_

_x0007__x0007_ _x0007__x0007_ _x0007_

_x0007_
_x0007__x0007__x0007_

_x0007__x0007_ _x0007__x0007__x0007_ _x0007__x0007_
_x0007_
_x0007_
_x0007__x0007__x0007__x0007_
_x0007__x0007_

_x0007_
_x0007__x0007_

_x0007__x0007_

_x0010_

_x0010_

_x0010__x0010__x0012_

_x0010__x0010_

_x0012_

_x0016_!_x001D_
_x001D__x001D__x001D__x0019__x001D__x001D__x0016__x0016__x0010__x0016__x0016__x001
9__x0012__x0010__x0019__x0019__x0010__x0010__x0012__x0012_

_x0012_

_x0012__x0012__x0012_

_x0012_

_x0007__x0007__x0007_

_x0010_

_x0007__x0007_ _x0007__x0007_
_x0007__x0007__x0007__x0007__x0007_

_x0007__x0007__x0007_ _x0007_ _x0007_ _x0007_ _x0007__x0007_
_x0007_ _x0007_ _x0007_ _x0007__x0007_
_x0007_ _x0007__x0007__x0007__x0007__x0007__x0007_ _x0007__x0007_

_x0007_
_x0007_
_x0007__x0007_

_x0007__x0007_
_x0007_
_x0007_
_x0007_
_x0007__x0007_ _x0012__x0010__x0012__x0010__x0010_

_x0012__x0010_

_x0012__x0010__x0010__x0012_

_x0019__x0012__x0016__x0016__x0019_-_x0016__x001D__x0019_!_x0019__x001D__x0016__x001D__x0012__x0016__x001D__x0019__x0016_

_x0012__x0010__x0010_

_x0012_

_x0010_

_x0007_
_x0007__x0007_

_x0007__x0007__x0007__x0007_

_x0007_
_x0007__x0007__x0007__x0007__x0007_
_x0007__x0007_ _x0007_ _x0007_ _x0007_ _x0007_
_x0007_

_x0007__x0007__x0007__x0007_
_x0007_ _x0007_

_x0007_
_x0007__x0007_ _x0007_

_x0010_
_x0012_

_x0012_

_x0012__x0010__x0010__x0016__x001D__x0016__x0019__x0010__x0019__x001D__x0016__x0016_ _x0019__x0016__x0019__x0016_ _x0019__x0012__x0019__x0019_

_x0012__x0010_

_x0012__x0012__x0012__x0010__x0010_

_x0012_

_x0016__x0010_
_x0010_
_x0010_

_x0007_

_x0010_

_x0007_

_x0007_
_x0007__x0007_

_x0007__x0007__x0007__x0007_
_x0007_ _x0007_ _x0007_ _x0007_ _x
0007_ _x0007__x0007__x0007_ _x0007_ _x0007__x0007_
_x0007__x0007__x0007_ _x0007_

_x0007_ _x0007_
_x0007__x0007__x0007_

_x0007_

_x0007__x0007_

_x0012__x0010__x0012__x0012__x0010_
_x0012__x0012__x0016__x0010_

_x0016_

_x0010__x0012__x0016__x0012__x0010__x0012__x0019__x0016__x0016__x0019__x0019__x0019__x0016__x001D_--_x0016__x001D__x001D_-"_x001D__x0016__x001D__x0016__x001D__x0019__x0016__x001D__x0010__x0010__x0019__x0016_

_x0012_

_x0012__x0012_

_x0010_

_x0012__x0010__x0012__x0010__x0010_

_x0012__x0016__x0012__x0012__x0016__x0016__x0016__x0019_-_x0016__x001D_-_x001D__x00
19_-_x0016__x0019__x0012__x001D_ -_x0019__x0019_-_x0019__x0016__x0019__x0010__x0010_

_x0016__x0010__x0012_

_x0010_
_x0012_

_x0010_

_x0010_

_x0010__x0010_

_x0012__x0010_

_x0010_

_x0007_ _x0007__x0007_

_x0007_
_x0007__x0007__x0007__x0007__x0007__x0007__x0007__x0007__x0007__x0007_ _x0007__
x0007__x0007_
_x0007__x0007_ _x0007_
_x0007_ _x0007_ _x0007_ _x0007_ _x0007_
_x0007__x0007_ _x0007_ _x0007__x0007__x0007__x0007__x0007_ _x0007_ _x0007_

_x0007__x0007__x0007__x0007_ _x0007_ _x0007_

_x0007_

_x0012__x0012__x0012__x0010_

_x0012__x0010__x0012__x0012__x0010_

_x0010__x0012__x0012__x0019__x0016__x001D_-$_x0016__x0016__x0016_
_x001D__x0016__x0019__x0019__x0016_$ -
"_x0019__x0016__x0016__x0016__x001D__x001D__x0012__x0016__x0010__x0012__x0012__x0012_
_x0019_

_x0012_

_x0010__x0012_

_x0010_

_x0010_

_x0007_
_x0007_ _x0007_ _x0007__x0007__x0007__x0007__x0007_

_x0007_
_x0007__x0007_
_x0007_
_x0007__x0007__x0007_
_x0007__x0007__x0007_ _x0007__x0007_ _x0007_
_x0007__x0007_
_x0007__x0007__x0007__x0007_ _x0007__x0007__x0007_ _x0007_
_x0007__x0007__x0007_
_x0007__x0007__x0007__x0007__x0007__x0007_ _x0007_
_x0007_

_x0007_

_x0007__x0010_

_x0010_

_x0010_

_x0019__x0012_

_x0010__x0012__x0012__x0016_-.
_x0019__x0019__x0016__x0010__x0012_!#$_x0019__x0019__x0012__x0019__x0012__x0010_

_x0012__x0012__x0012__x0012_

_x0010_

_x0010_

_x0007_

_x0007_ _x0007__x0007_
_x0007_
_x0007__x0007__x0007__x0007__x0007_
_x0007_ _x0007_ _x0007_ _x0007__x0007_ _x0007_
_x0007_ _x0007__x0007_ _x0007__x0007_ _x0
007_ _x0007_ _x0007_ _x0007__x0007__x0007__x0007__x0007_
_x0007__x0007__x0007_

_x0007__x0007_ _x0007__x0007__x0007__x0007_

_x0007_ _x0007_
_x0007__x0007_

_x0010__x0012_

_x0010_

_x0010__x0016__x0010__x0019__x001D_&(_x001D_-_x0012__x0019__x0019_

_x0012__x0016_-!%_x001D__x0019__x001D__x0016__x0016__x0019__x0016__x0012_

_x0012_

_x0012__x0016__x0010_

_x0012_

_x0010_

_x0012__x0010_

_x0012_

_x0010_

_x0007_

_x0007_ _x0007__x0007__x0007__x0007__x0007__x0007_ _x0007__x0007_

_x0007_ _x0007_
_x0007__x0007__x0007_ _x0007_ _x0007_
_x0007__x0007__x0007_
_x0007_ _x0007_ _x0007_
_x0007_
_x0007_
_x0007__x0007_

_x0007_

_x0007_ _x0007_

_x0007_
_x0007_

_x0007_

_x0012_

_x0012__x0012__x0016__x0010__x0010_

_x0010__x0012__x0019__x0016__x0019_!_x0010__x0010__x0012__x0016__x0019__x001D__x0019__x0019__x0019__x001D_!-_x0019__x0019__x001D__x0019__x0019__x0010__x0016__x0019__x0016__x0019_

_x0016__x0012__x0016__x0010_

_x0012__x0019__x0012_

_x0010_

_x0012_

_x0012_

_x0012__x0012__x0010_

_x0010_

_x0007__x0007_

_x0007__x0007_
_x0007_ _x0007_
_x0007__x0007__x0007_ _x0007__x0007_ _x0007_
_x0007__x0007_ _x0007__x0007_ _x0007__x0007__x0007_
_x0007_ _x0007__x0007__x0007_ _x0007_
_x0007__x0007_

_x0007__x0007__x0007_ _x0007__x0007__x0007__x0007_

_x0007_

_x0007_ _x0007_
_x0007__x0007_

_x0010_

_x0016_

_x0012__x0010__x0012__x0010__x0016__x0012__x0010_

_x001D__x0019__x0019__x0016__x0016_

_x0016__x0010__x0016__x0012__x0016__x001D__x001D__x001D__x0019__x001D__x0012_-_x00
16_

_x0016__x0012__x0012__x0012__x0012__x0012__x0016_

_x0016_
_x0010_

_x0010_
_x0010_

_x0012_

_x0007_

_x0007_

_x0007_

_x0007__x0007_

_x0007_ _x0007_
_x0007__x0007_

_x0007_ _x0007_ _x0007__x0007__x0007__x0007__x0007__x0007_
_x0007__x0007_   _x0007_
_x0007_   _x0007_   _x0007_   _x0007_   _x0007_   _x0007_
_x0007__x0007_   _x0007__x0007_   _x0007_   _x0007_   _x0007_   _x0007
_   _x0007_   _x0007__x0007_   _x0007__x0007__x0007__x0007_ _x0007_
_x0007_

_x0007_

_x0007_ _x0007_
_x0007_ _x0007_

_x0007__x0007_

_x0007_
_x0010_ _x0007_

_x0010_

_x0012_

_x0012__x0016__x0016__x0016_

_x0016__x0016__x0016__x0019__x0016__x0019__x0016__x0012__x0010__x0010_!
"_x0016_--_x0016__x001D__x0012__x0016__x0019__x0012__x0010__x0016__x0012__x0012_

_x0010__x0010_

_x0012__x0012_

_x0016__x0010_

_x0010_

_x0010_

_x0010_

_x0007_

_x0007_

_x0007_

_x0007_

_x0007_
_x0007__x0007_

_x0007__x0007_
_x0007__x0007__x0007_
_x0007_ _x0007_ _x0007_ _x0007__x0007_ _x0007__x000
7__x0007_ _x0007_
_x0007__x0007__x0007_ _x0007__x0007_
_x0007_

_x0007__x0007__x0007_
_x0007_
_x0007__x0007__x0007_ _x0007_

_x0007__x0007_
_x0007__x0007_

_x0010_

_x0012_

_x0012__x0016__x0016__x0016__x0019_

_x0019_ _x0019__x0019__x0016__x0016__x001D_
_x001D__x0016__x0016__x0012__x0016__x0010__x0012__x0012_

_x0016_

_x0012_

_x0012_
_x0010_

_x0012_

_x0012_

_x0010_

_x0010_
_x0010__x0012_

_x0010_

_x0007_
_x0007__x0007_

_x0007__x0007_

_x0007_

_x0007_ _x0007__x0007_ _x0007_
_x0007__x0007_ _x0007_
_x0007_ _x0012_ _x0007_
_x0007_ _x0007_
_x0007_ _x0007_ _x0007__x0007__x0007__x0007__x0007_

_x0007__x0007__x0007_
_x0007__x0007_

_x0007_

_x0007_

_x0007__x0007_ _x0007__x0007_

_x0007_

_x0010_

_x0012_

_x0010_

_x0012__x0016__x0010__x0010__x0012__x0019__x0019_-_x0016_!_x0016_-_x0019__x0016__x0016__x0016__x0019_

_x0012_

_x0010_

_x0010_

_x0010_

_x0010__x0010_

_x0010__x0010__x0010__x0010_

_x0007_

_x0007__x0007__x0007_

_x0007_

_x0007_

_x0007__x0007_
_x0007_
_x0007_ _x0007__x0007_ _x0007_ _x0007__x0007_

_x0007__x0007_
_x0007__x0007_ _x0007_
_x0007_
_x0007__x0007_ _x0007_ _x0007_

_x0007_
_x0007_

_x0007__x0007_

_x0012__x0010__x0012__x0012__x0012__x0012__x0010__x0016_

_x0012__x0019__x0012__x0012__x0016__x0019__x0019__x0016__x0016__x0019__x0019__x0016_-_x001D__x0019__x0016_-_x0012__x0016__x0016__x0012__x0010__x0010__x0016__x0010__x0012__x0012_

_x0012_
_x0012_

_x0016__x0010__x0012__x0012_

_x0010__x0010__x0010__x0012_

_x0010_

_x0007_

_x0007_ _x0007_

_x0007__x0007__x0007_
_x0007_

_x0007__x0007_

_x0007__x0007__x0007_
_x0007_
_x0007_ _x0007_

_x0007_ _x0007_ _x0007__x0007__x0007__x0007__x0007_ _x0007__x0007_
_x0007__x0007_
_x0007_

_x0007__x0007_
_x0007__x0007_ _x0007__x0007__x0007_

_x0007__x0007_

_x0007__x0007_

_x0007_

_x0007_

_x0010_
_x0012_

_x0010__x0012__x0010__x0010__x0012__x0019__x0016__x0012__x0016__x0016__x0012__x0010
__x0016__x0016__x0016__x0016__x0012__x0019__x0016__x001D__x0016__x0019__x001D_-_x00
1D__x0019__x0019__x0019__x0010__x0019__x0012__x0016__x0016_

_x0012__x0010_

_x0010__x0010_
_x0012__x0016__x0010_

_x0010_

_x0010_

_x0010_

_x0010__x0012_

_x0010_

_x0007_

_x0007_

_x0007_

_x0007_

_x0007_ _x0007__x0007_
_x0007_
_x0007__x0007_

_x0007_
_x0007_
_x0012_ _x0007_ _x0007_ _x0007_ _x0007_ _x0007__x0007_ _x000
7_ _x0007__x0007_ _x0007_ _x0007__x0007__x0007__x0007__x0007__x0007_

_x0007__x0007__x0007__x0007_

_x0007__x0007_

_x0007_

_x0007_

_x0010__x0010__x0012__x0010__x0012__x0010__x0012_

_x0010__x0012__x0012__x0016__x0010_

_x0016__x0012__x0019__x0012__x0016__x0016__x0016__x0019__x0019_-_x0016__x0010__x0012
__x0016__x0016__x0019__x0019_ !--_x0016_ _x0016__x0016__x0016__x0012__x0010_
_x0010__x0012_
_x0010_

_x0012__x0016_

_x0010_

_x0012_

_x0010_

_x0007_
_x0010_

_x0007_ _x0007_ _x0007__x0007__x0007_

_x0007__x0007__x0007_ _x0007_ _x0007__x0007__x0007_
_x0007__x0007_ _x0007_
_x0007__x0007_ _x0007_ _x0007_ _x0007__x0007_ _x0007__x0007
_ _x0010_ _x0007_ _x0007_ _x0007__x0007_

_x0007_ _x0007__x0007_
_x0007__x0007__x0007__x0007_
_x0007_
_x0007__x0007_
_x0007__x0007__x0007__x0007_

_x0007__x0007_

_x0010_

_x0012_

_x0010_

_x0010__x0010__x0012__x0010__x0016__x0012__x0016_-_x0019__x0012__x0010__x0012__x0012__x0016__x0010__x0012__x0012__x0010__x0019_
_x001D__x001D__x0016__x0016__x0012__x0010__x0010_

_x0012_

_x0010_

_x0012_

_x0010_

_x0012_

_x0010_

_x0007_

_x0007_

_x0007__x0007_

_x0007_ _x0007__x0007_ _x0007__x0007__x0007__x0007_
_x0007__x0007_
_x0007__x0007__x0007_ _x0007_ _x0007__x0007_

_x0007_

_x0016_ _x0007_ _x0007_ _x0007_ _x0007_
_x0007_

_x0007__x0007__x0007__x0007__x0007__x0007_
_x0007_ _x0007__x0007__x0007_

_x0007_
_x0007_ _x0007_

_x0007_

_x0007_
_x0007_

_x0007_

_x0010_

_x0010__x0012__x0010__x0016__x0012__x0012__x0016__x0016_
_x0019__x0019__x0016_

_x0019_"_x0019__x0016__x0019__x0016__x0016__x0016__x0012__x0010__x0010__x0010__x0010_

_x0010__x0010_

_x0010__x0012_

_x0012__x0010_

_x0010_

_x0012__x0010_
_x0012__x0010_

_x0010_

_x0010_

_x0007_
_x0010_
_x0007_

_x0007__x0007__x0007__x0007__x0007__x0007__x0007__x0007__x0007__x0007__x0007_
_x0007_ _x0007__x0007__x0007__x0007_
_x0007_ _x0007__x0007_ _x0007_
_x0007_ _x0007_ _x0007_, _x0007_ _x0007_ _x0007_ _x0007_

_x0007__x0007_ _x0007__x0007__x0007_

_x0007__x0007__x0007_

_x0007__x0007_

_x0007__x0007_

_x0010_
_x0010_

_x0007_

_x0007_

_x0010__x0012_

_x0012_

_x0010__x0012__x0012__x0012__x0010__x001D__x0016__x0012__x0016__x0016_-_x0019_ #
!_x0019__x0016__x001D_--_x0019__x0016__x0019__x0012__x0012__x0016_

_x0012__x0012_

_x0010__x0012__x0010_

_x0010_

_x0010__x0012__x0010__x0010__x0010_

_x0012__x0012_

_x0010_

_x0010_

_x0007__x0007__x0007__x0007_

_x0007_
_x0007_

_x0007__x0007__x0007__x0007_ _x0007_ _x0007_
_x0007_

._x0012_ _x0007_ _x0007_ _x0007_

_x0007_ _x0007__x0007__x0007__x0007__x0007_
_x0007_
_x0007_
_x0007_

_x0007_

_x0007_

_x0007_

_x0007_

_x0010_
_x0010__x0012_

_x0010_
_x0010_

_x0019__x0019__x0012__x0016__x0012_-_x0016__x0016__x0016__x0019__x0019__x0016_)#-_x0016__x0016__x0016__x001D__x0012__x0016_

_x0019_

_x0016__x0012_

_x0010_

_x0010_

_x0010_

_x0010__x0012_

_x0007_

_x0010_

_x0007_

_x0007__x0007_

_x0007__x0007__x0007_ _x0007__x0007__x0007__x0007_
_x0007_ _x0007__x0007_ _x0007_ _x0007_ _x0007__x0007_ _x0007_
_x0007__x0007__x0007_

_x0007_ _x0007_ _x0007__x0007_

_x0007_

_x0007_
_x0007_

_x0010_

_x0007_

_x0007_
_x0010_

_x0010_

_x0012_

_x001D__x0016__x0016__x0016__x0016__x0012_-_x0016__x0016__x001D_
-!_x001D__x0019__x0019__x0016__x0019__x0012_
_x0012__x0019__x0012_ _x0010__x0010_

_x0010__x0010_

_x0010_

_x0010__x0010__x0012__x0012_

_x0007_

_x0007_

_x0007_ _x0007_

_x0007__x0007__x0007_

_x0007_ _x0007__x0007_ _x0007__x0007__x0007_ _x0007__x0007__x0007_
_x0010__x0010__x0007_ _x0007_ _x0007_

#-   _x0007_   _x0007_

_x0007_   _x0007_

_x0016__x0010_
_x0007__x0007_   _x0007__x0007_   _x0007__x0007_   _x0007__x0007_

_x0012__x0016__x0012__x0016__x0012__x0019_

_x0019__x0016__x0012__x0016__x001D_
_x0016__x001D__x0016__x0016__x0016__x001D__x0019__x0019__x0016__x0010__x0010__x0010
__x0012__x0010__x0010_

_x0010__x0019__x0012_

_x0012_

_x0010__x0010_

_x0010__x0010__x0010_

_x0007_

_x0007__x0007_
_x0007_

_x0007_ _x0007__x0007__x0007_
_x0007_
_x0007__x0007_ _x0007_ _x0007_ _x0007_ _x0007_
-% _x0007_ _x0007_ _x0007__x0007_ _x0007__x0007_
_x0007__x0007__x0007__x0007__x0007_

_x0010_
_x0007_
_x0007__x0007_
_x0007_

_x0007__x0007_ _x0007__x0007_

_x0010_

_x0007_

_x0007_ _x0010_

_x0010__x0016__x0010__x0016__x0010_
_x0016__x0012__x0016_#-!_x0019__x0016__x0016__x0019__x0016__x001D__x0010__x0010_
_x0012_

_x0010__x0016_

_x0012__x0010_

_x0010__x0012_

_x0010_

_x0007_

_x0007__x0007__x0007__x0007__x0007__x0007__x0007_ _x0007_
_x0007__x0007__x0007_
_x0007_ _x0007__x0007__x0007__x0007_ _x0007_
_x0007__x0007_ _x0007__x0007__x0007_ _x0007_ _x0007_ ,
_x0007__x0007__x0007_ _x0007_ _x0007__x0007__x0007__x0007__x0007_ _x0007__x0007__x0007__x0007__x0007__x0007__x0007_
_x0010_ _x0007_
_x0007__x0007__x0007_

_x0007_ _x0007__x0007__x0007_

_x0012_

_x0012__x0010_ _x0010__x0016_

_x0010_

_x0012_

_x0010__x0010_

_x0012__x0012__x001D__x0016__x001D__x0019__x0016__x0016__x0019__x001D_-_x0019__x001D__x001D__x0016__x0019__x0016__x0012__x0012__x0012__x0012_

_x0012_

_x0010_

_x0010__x0010__x0010_

_x0012__x0012__x0012__x0010__x0012_

_x0010_

_x0012_

_x0010_

_x0012_

_x0010__x0012__x0019__x0019__x0019__x0016__x0019__x0016__x001D_-_x001D__x0019__x0012__x0016__x001D__x0016__x0012__x0016__x0016_

_x0010_

_x0010_

_x0012_

_x0012__x0010_

_x0010_

_x0012__x0010__x0010__x0012__x0010_

_x0010__x0010_

_x0007_

_x0007__x0007__x0007_
_x0007__x0007_

_x0007_
_x0007__x0007__x0007_ _x0007__x0007_

_x0007_

_x0007__x0007_
_x0007__x0007_
_x0007_ _x0007_

_x0007__x0007_ _x0007_ _x0007__x0007__x0007__x0007_
_x0007_

_x0007_
_x0007__x0007_

_x0007_
_x0007_
_x0007_

_x0007_

_x0007__x0007_
_x0007_

_x0010_

_x0012_

_x0010_

_x0012__x0016__x0019__x0019__x0019__x0016__x001D__x001D__x0019__x001D__x0016__x0016_

_x0010_

_x0016__x0010__x0010_

_x0010_

_x0016__x0010__x0010_

_x0010_

_x0012_ _x0010_

_x0012_

_x0007__x0007_

_x0007__x0007_

_x0007__x0007__x0007__x0007_ _x0007__x0007_

_x0007__x0007_ _x0007__x0007_

_x0007__x0007_ _x0007_ _x0007_ _x0007_

_x0007_ _x0007_
_x0007__x0007__x0007__x0007__x0007_ _x0007_ _x0007__x0007__x0007__x0007__x0007
_

_x0007__x0007_

_x0007_

_x0007_

_x0007_

_x0007_

_x0007_

_x0007_

_x0007_

_x0010__x0016__x0010__x0010_

_x0010_

_x0010_

_x0012_

_x0012__x001D__x0019__x0016__x0016__x0016__x0019_!_x0019_ _x0016_

_x0010__x0012__x0010__x0010__x0010_

_x0012__x0010__x0010_

_x0012_

_x0010__x0010__x0010_

_x0010__x0012_

_x0007_

_x0007_

_x0007__x0007_
_x0007_
_x0007_

_x0007__x0007__x0007__x0007_ _x0007_ _x0007__x0007__x0007_ _x0007_
_x0007__x0007_ _x0007_ _x0007_ _x0007_
_x0007_
_x0007_ _x0007_ _x0007_ _x0007__x0007_
_x0007_
_x0007__x0007__x0007__x0007_

_x0007_

_x0007_ _x0007_
_x0007_

_x0007_

_x0010__x0010__x0010__x0010_
_x0010__x0012_
_x0012_

_x0016__x0012_

_x0010__x0012__x0012_

_x0016__x0012__x0012__x0016__x0019__x0016__x0016__x0019_
_x0019__x001D_!_x0016__x0016__x0010__x0012__x0010_

_x0016__x0012__x0012__x0010__x0010__x0010__x0012_

_x0010__x0016__x0010__x0012_

_x0010_

_x0012_

_x0007__x0010__x0010__x0012_

_x0012_

_x0007_

_x0007__x0007__x0007_
_x0007__x0007_ _x0007__x0007__x0007_
_x0007__x0007__x0007__x0007__x0007__x0007__x0007_ _x0007_ _x0007_
_x0007__x0007__x0007__x0007_

_x0007__x0007__x0007__x0007_
_x0007_ _x0007_ _x0007_ _x0007__x0007__x0007_
_x0007__x0007_ _x0007__x0007__x0007_ _x0007_
_x0007__x0007__x0007_

_x0007__x0007_
_x0007_

_x0007_

_x0007_

_x0007_

_x0007_

_x0010__x0010_
_x0012_

_x0012_

_x0010_

_x0012__x0010__x0010_

_x0010_

_x0012_

_x0012__x0012__x0010_

_x0019__x001D__x0016__x0019__x0019__x0019__x0019__x0019__x0019__x0019__x001D__x0016__x0016__x0012_

_x0010__x0010__x0012__x0012__x0010_

_x0010_

_x0012_

_x0010_

_x0010_

_x0010_

_x0007_
_x0007__x0007_
_x0007__x0007__x0007_

_x0007__x0007_

_x0007_
_x0007_
_x0007_ _x0007__x0007_
_x0007__x0007__x0007_ _x0007_ _x0007_ _x0007__x0010_ _x0007_
_x0007_ _x0007_ _x0007__x0007_ _x0007__x0007__x0007__x0007_
_x0007_
_x0007_ _x0007_ _x0007__x0007_

_x0007_

_x0007_
_x0007_
_x0007_

_x0010_

_x0010_

_x0010_

_x0010_

_x0010__x0010_
_x0010_

_x0016__x0016__x0019__x0016__x0019__x0016_-!_x001D_-_x0019_
-_x0016__x0016__x0016__x0019__x0010_
_x0010__x0010_

_x0010__x0016_

_x0010_

_x0012_

_x0007_

_x0010_

_x0010_

_x0007_

_x0007_

_x0007_
_x0007_
_x0007__x0007_
_x0007_

_x0007_
_x0007_

_x0007_ _x0007__x0007__x0007__x0007_
_x0007_

_x0007_ _x0007__x0007_ _x0007_
_x0007_ _x0007_ _x0007_
_x0007__x0007__x0007__x0007__x0007__x0007__x0007__x0007_

_x0007_

_x0007_

_x0007__x0007_
_x0007_

_x0007_

_x0007_

_x0007_

_x0010_

_x0012__x0007_

_x0007_

_x0010__x0010__x0010_

_x0016_

_x0010__x0012_

_x0012_

_x0012__x0010_

_x0012__x0016__x0016__x001D__x0019__x001D__x001D_!_x0019__x001D_

_x0019__x0012__x0012__x0016__x0010_

_x0012_

_x0012__x0012__x0010__x0016_
_x0010_
_x0010__x0012__x0012_

_x0010_

_x0010_
_x0010_

_x0010_

_x0010_

_x0007_

_x0007__x0007__x0007_

_x0007_

_x0007__x0007__x0007__x0007_ _x0007_
_x0007_ _x0007__x0007_ _x0007__x0007_
_x0007_ _x0007_
_x0007_ _x0007_ _x0007_ _x0007__x0007_
_x0007__x0007__x0007__x0007_ _x0007_
_x0007_ _x0007_

_x0007__x0007__x0007_

_x0007_

_x0007_
_x0007_
_x0007_

_x0007__x0007__x0010_

_x0007_

_x0007_

_x0010_

_x0012_

_x0010__x0010_

_x0012_

_x0010_

_x0012__x0016__x0019__x0019__x0016__x0019__x0019__x0019_-_x001D_-_x0019__x0019__x001
2__x0016__x0016_

_x0012_

_x0010__x0010__x0016__x0010__x0010__x0010_

_x0010__x0012_

_x0010_

_x0007_
_x0007__x0007_

_x0007__x0007__x0007_ _x0007_
_x0007_ _x0007__x0007__x0007__x0007_ _x0007_ _x0007_ _x0007__x0007_ _x000
7__x0007_ _x0007_
_x0007_ _x0007_ _x0007__x0007_ _x0007_ _x0007__x0007_
_x0007_ _x0007_ _x0007__x0007_ _x0007_ _x0007__x0007_

_x0007__x0007_

_x0007__x0007_

_x0007_ _x0007_ _x0007_

_x0007_

_x0007__x0007__x0007__x0007_

_x0010_

_x0007_

_x0012__x0010_

_x0010_

_x0012__x0010_

_x0010__x0012__x0012__x0012__x0016__x001D_--_x0016__x0010__x0019__x0010_

_x0012_

_x0010_

_x0012__x0012_

_x0012_
_x0010_

_x0012_
_x0010__x0010_

_x0010_

_x0010_

_x0007_

_x0007_

_x0007__x0007_

_x0007_
_x0007__x0007__x0007__x0007__x0007_ _x0007__x0007__x0007_ _x0007_ _x0007__x0007_
_x0007_ _x0007_ _x0007_ _x0007_ _x0007_ _x0007_

_x0007__x0007_ _x0007__x0007_ _x0007__x0007_ _x0007_

_x0007_

_x0007_

_x0007_

_x0007__x0007_

_x0007_

_x0010_

_x0012_

_x0010__x0010_

_x0012_

_x0007_

_x0012_

_x0016__x0010_

_x0012_

_x0016__x0016__x0010__x0016__x0016_-_x0019__x0019__x0016__x001D__x0019__x0019__x0019__x001D__x0012_

_x0010__x0010__x0016__x0010_

_x0010_

_x0010_

_x0010_

_x0012__x0010_

_x0012_

_x0012__x0012__x0010_

_x0012_

_x0012_
_x0010_

_x0010_

_x0007_

_x0007__x0007_

_x0007__x0007__x0007__x0007_
_x0007__x0007__x0007__x0007__x0007__x0007__x0007__x0007__x0007_
_x0007_ _x0007__x0007__x0007_ _x0007__x0007_ _x0007_
_x0007_ _x0007_
_x0007__x0007__x0007__x0007_
_x0007_ _x0007__x0007_ _x0007__x0007_

_x0007_ _x0007_

_x0007_

_x0010_

_x0012__x0016_

_x0012_

_x0012__x0010__x0010__x0019__x0016__x0019__x0019__x0016__x0019__x0019__x001D__x0016
_-_x001D_-_x001D__x0019__x0019__x0012__x0010__x0016__x0010__x0016__x0012__x0010_

_x0010__x0010__x0010_

_x0010_

_x0010_

_x0010_

_x0007_
_x0007_

_x0007_
_x0007_

_x0007_

_x0007__x0007_
_x0007_
_x0007_ _x0007_ _x0007__x0007__x0007_ _x0007_
_x0007_ _x0007_ _x0007_
_x0007__x0007__x0007_ _x0007__x0007_ _x0007__x0007__x0007_
_x0007__x0007_
_x0007__x0007__x0007_ _x0007_ _x0007__x0007__x0007_
_x0007__x0007_

_x0007__x0007_

_x0007_

_x0010_

_x0012_

_x0012_

_x0010_ _x0010_

_x0012__x0010__x0012__x0016__x0010__x0016__x0016__x0012__x0012__x0010__x0019__x0016
__x001D__x0019__x001D__x0016__x001D__x0016__x001D__x0016__x0016__x0016_

_x0010_

_x0010_

_x0012__x0012_

_x0010_

_x0012_

_x0010_

_x0012__x0007_

_x0010__x0010_

_x0007_

_x0007_

_x0007_

_x0007_

_x0007__x0007__x0007__x0007_ _x0007__x0007__x0007_ _x0007_ _x0007_
_x0007_ _x0007_
_x0007_ _x0007_ _x0007_ _x0007_ _x0007_ _x0007_ _x0007_
_x0007_
_x0007__x0007_

_x0007_

_x0007__x0007_
_x0007_ _x0007__x0007_
_x0007_

_x0007_
_x0007__x0007_

_x0012_

_x0010__x0012_

_x0010_

_x0010__x0012_

_x0010_

_x0016_

_x0012__x0012__x0010__x0012__x0012__x0019__x0019__x0019_-_x0016__x0016__x0019__x0016
__x0019__x0016__x001D_!

_x0019__x001D__x0012__x0016__x0010_

_x0010__x0012_

_x0010_
_x0010__x0012__x0012_

_x0012_

_x0012_

_x0010_
_x0010_

_x0010_

_x0007_

_x0007_ _x0007_
_x0007_ _x0007_
_x0007__x0007__x0007_ _x0007__x0007_ _x0007__x0007_ _x0007__x0007_
_x0007__x0007_ _x0007_ _x0007_

_x0007_ _x0007__x0007_

_x0007_

_x0007_
_x0007_
_x0007_

_x0007_
_x0007__x0007_

_x0010_

_x0007_

_x0007_

_x0010_

_x0010__x0010_

_x0010_

_x0012_ _x0012__x0010_

_x0016__x0010_

_x0012__x0012_

_x0016__x0019__x0019__x0019__x0019__x0016_"_x0019_-"_x0019__x0012__x0010_

_x0016__x0012_

_x0016__x0016__x0012__x0010_
_x0012__x0019_

_x0010__x0010_

_x0012__x0012__x0010__x0012__x0010_

_x0010_

_x0010__x0010_

_x0007__x0007_
_x0007_

_x0007_

_x0007_

_x0007__x0007_

_x0007_

_x0007__x0007__x0007__x0007__x0007__x0007_ _x0007__x0007__x0007_

_x0007__x0007__x0007_ _x0007__x0007__x0007_ _x0007_ _x0007_ _x0007__x0007__x0007__x0007__x0007__x0007_ _x0007_

_x0007__x0007_ _x0007__x0007_ _x0007_

_x0007_

_x0007__x0007_

_x0007_
_x0007_

_x0007_
_x0007_
_x0007_
_x0007_

_x0010_

_x0010_

_x0010__x0010__x0012__x0012_

_x0010__x0010_

_x0010_

_x0016__x0012_

_x0012__x0019__x0010__x0010__x0012__x0012__x0019_!_x0016__x0016__x0016__x001D_-
_x0016__x0016__x0019__x0010_

_x0010__x0012__x0010__x0016__x0010__x0012_
_x0012_

_x0010__x0012_

_x0010_

_x0010__x0010__x0010_

_x0010__x0010_

_x0010__x0010_

_x0007_

_x0007_
_x0007_
_x0007__x0007__x0007_ _x0007_ _x0007__x0007_
_x0007_ _x0007_
_x0007_ _x0007_ _x0007_ _x0007__x0007_ _x0007__x0007_

_x0007_ _x0007__x0007_
_x0007_ _x0007_ _x0007__x0007__x0007_
_x0007_
_x0007__x0007_

_x0007_ _x0007_ _x0007__x0007_ _x0007_

_x0007_

_x0007_

_x0010_
_x0010_

_x0012_

_x0010__x0010_

_x0010__x0010__x0012__x0010_

_x0012_

_x0010_

_x0016__x0012__x0016__x0019__x0016__x0012__x0016_-!_x0019_
_x0019__x0019__x0016__x0012__x0010__x0012_

_x0010__x0012_
_x0010_
_x0010_

_x0010__x0010__x0012_

_x0010__x0012_

_x0010_

_x0010_

_x0012_

_x0010_

_x0007_

_x0007_ _x0007__x0007_
_x0007__x0007_ _x0007__x0007_ _x0007_ _x0007_

_x0007_ _x0007__x0007__x0007__x0007__x0007_

_x0007_ _x0007__x0007__x0007_
_x0007__x0007_ _x0007__x0007_

_x0007_

_x0012_

_x0012_

_x0016_

_x0010__x0010__x0010__x0012__x0019_
_x0010_

_x0010_

_x0007_

_x0007__x0007_

_x0007_

_x0007_

_x0007_
_x0007__x0007_ _x0007__x0007_

_x0007__x0007__x0007_ _x0007__x0007__x0007_ _x0007__x0007__x0007__x0007__x0007_ _x
0007__x0007__x0007__x0007__x0007_
_x0007_ _x0007_

_x0007__x0007_ _x0007_ _x0007_ _x0007__x0007_

_x0007_ _x0007__x0007__x0007__x0007__x0007__x0007_
_x0007__x0007_
_x0007_

_x0007_
_x0007_ _x0007_

_x0007_

_x0007_
_x0007_

_x0012__x0010_

_x0010_

_x0010_

_x0012_

_x0016_

_x0012__x0016_

_x0012__x0010_

_x0010_

_x0010_

_x0010_

_x0012__x0010_

_x0007_
_x0007_

_x0007_
_x0007_

_x0007_
_x0007__x0007_
_x0007_ _x0007__x0007__x0007__x0007__x0007_ _x0007__x0007_
_x0007__x0007__x0007__x0007_

_x0007_ _x0007_

_x0007_ _x0007_ _x0007__x0007_ _x0007_ _x0007__x0007_
_x0007__x0007__x0007_ _x0007__x0007__x0007__x0007__x0007_
_x0007__x0007__x0007__x0007__x0007__x0007__x0007_
_x0007__x0007_
_x0007__x0007__x0007_
_x0007_

_x0007_

_x0007_

_x0012__x0012__x0012_
_x0012__x0010_

_x0010__x0012__x0012_

_x0010__x0010__x0010_

_x0010__x0016__x0019__x0016__x0016__x0016_$#"_x001D__x0016__x0016__x0016__x0016__x0016__x001D__x0016_ _x0010_
_x0010__x0010__x0010_

_x0010__x0012_

_x0010_
_x0012_

_x0012_

_x0007_

_x0010_

_x0007_

_x0007__x0007_

_x0007__x0007_

_x0007__x0007_ _x0007_

_x0007_ _x0007__x0007_
_x0007_ _x0007_
_x0007_ _x0007__x0007__x0007__x0007__x0007_ _x0007_

_x0007_ _x0007_ _x0007_ _x0007_
_x0007_ _x0007_ _x0007__x0007__x0007_
_x0007__x0007__x0007_ _x0007_
_x0007_ _x0007__x0007_

_x0007_
_x0007_

_x0007_

_x0010_

_x0010__x0010__x0010_

_x0012_

_x0012__x0012__x0012__x0012__x0010_

_x0016_-_x001D__x0019__x0019__x001D__x0016__x0019__x0019__x0019__x0010__x0019__x0019__x0016_

_x0019_

_x0012_

_x0010_ _x0010_

_x0012_

_x0012__x0010_

_x0010_

_x0016_

_x0007_

_x0010_

_x0007_
_x0007_

_x0007__x0007__x0007_
_x0007__x0007__x0007_
_x0007__x0007__x0007_ _x0007_
_x0007__x0007_ _x0007_ _x0007_ _x0007__x0007__x0007_ _x0007_

_x0007_ _x0007_ _x0007_ _x0007__x0007_ _x0007_
_x0007__x0007__x0007_ _x0007_ _x0007_ _x0007__x0007__x0007_ _x0007_
_x0007_

_x0007_
_x0007_

_x0010_
_x0007_ _x0007_

_x0007_
_x0007_
_x0007__x0007_
_x0010_

_x0010_
_x0010_

_x0016__x0012_

_x0012__x0012_
_x0010_

_x0012_

_x0010__x0012_

_x0016__x0016_

_x0012_$-_x001D__x0016__x0019__x0016__x0016__x0016__x0012__x0016__x0016_

_x0016__x0010__x0012__x0010_

_x0010_

_x0010_

_x0012_

_x0007_

_x0007_
_x0007_
_x0007_

_x0007_
_x0007_

_x0007__x0007_

_x0007__x0007__x0007__x0007_
_x0007__x0007_ _x0007__x0007_ _x0007_ _x0007_ _x0007_

_x0007_ _x0007__x0007_ _x0007_
_x0007__x0007__x0007__x0007__x0007_
_x0007__x0007__x0007__x0007__x0007__x0007__x0007_ _x0007_

_x0007_

_x0007__x0007_   _x0007__x0007_

_x0007__x0007__x0007_

_x0007_

_x0012_

_x0010__x0016_

_x0010__x0012_

_x0012__x0012_

_x0010_

_x0010__x0010_

_x0010__x0012_

_x0012__x0010__x0019__x0019_-!!_x001D__x0010__x0016__x001D__x0016__x0016__x0019__x0019__x0019__x0019_

_x0010__x0016_

_x0012_

_x0010__x0010_

_x0010__x0010__x0010_

_x0012_

_x0010_

_x0007_
_x0010_

_x0007_

_x0007_

_x0007_
_x0007_

_x0007__x0007__x0007_

_x0007_

_x0007_
_x0007__x0007_
_x0007_ _x0007__x0007_
_x0007_ _x0007__x0007__x0007__x0007__x0007_
_x0007__x0007__x0007__x0007__x0007__x0007_
_x0007_
_x0007__x0007_

_x0007__x0007_ _x0007_ _x0007__x0007_
_x0007__x0007_

_x0010_

_x0012_

_x0010__x0010_

_x0016__x0012__x0012_
_x0012_

_x0012_

_x0012__x0012__x0012__x0012__x0010__x0012__x0012__x0016__x0019_-
_x0019__x001D__x0019__x0016__x0016__x0019__x001D_

_x0010__x0012__x0010__x0010_

_x0012_

_x0010_

_x0012__x0012__x0010__x0012_

_x0010_ _x0010_

_x0012_

_x0010_

_x0007__x0010_

_x0007_

_x0007_
_x0007_

_x0007_

_x0007_ _x0007__x0007__x0007_ _x0007_ _x0007__x0007_ _x0007_ _x0007_

_x0007__x0007__x0007_ _x0007_ _x0007_ _x0007_ _x0007_
_x0007_ _x0007_

_x0007__x0007__x0007_

_x0007_

_x0010_

_x0012__x0010_

_x0010__x0012__x0010_

_x0012__x0016_

_x0010_

_x0012_

_x0012__x0016_#-"_x001D__x0016__x001D__x0016_

_x0012__x0012__x0010__x0016_

_x0012__x0010_
_x0010_

_x0012__x0010_

_x0010_

_x0010_

_x0007_

_x0010_

_x0010_

_x0007_

_x0007_ _x0010_
_x0007__x0007__x0007__x0007_
_x0007_

_x0007__x0007_

_x0007_
_x0007_
_x0007__x0007__x0007_ _x0007_ _x0007_ _x0007_
_x0007_ _x0007_ _x0007__x0007_ _x0007_ _x0007_
_x0007__x0007_ _x0007__x0007__x0007_ _x0007_ _x0007__x0007__x0007_
_x0007__x0007__x0007__x0007_
_x0007_

_x0007_
_x0007_
_x0007_

_x0010__x0010_

_x0012__x0012__x0010__x0010__x0010_

_x0016__x0010_

_x0016__x0016__x0010__x0010__x0012__x0010_

_x0010__x0010_

_x0019__x0016__x0016__x0016__x0012__x0012__x0016__x001D__x0019__x0019_&_x001D__x00
1D__x0019__x001D__x001D__x0019__x0012__x0016__x001D__x0010__x0010__x0010_

_x0012__x0010_

_x0016_ _x0010__x0010_

_x0010_
_x0012_

_x0007_

_x0007_ _x0007_

_x0007_ _x0007__x0007_

_x0007_

_x0007_ _x0007_

_x0007_
_x0007_ _x0007__x0007__x0007__x0007__x0007__x0007__x0007_ _x0007_
_x0007_ _x0007_ _x0007__x0007__x0007_ _x0007_
_x0007__x0007__x0007__x0007__x0007_ _x0007__x0007_ _x0007_

_x0007_

_x0007__x0007_

_x0007_

_x0007_

_x0012_

_x0012_

_x0016_

_x0010__x0016_

_x0012__x0010_
_x0012__x0012_

_x0016__x0016_

_x001D__x001D_
#-_x001D__x0016__x001D__x0019__x0016__x0016__x0016__x0016__x0019__x0016__x0012_

_x0010_

_x0010__x0012_

_x0010__x0010__x0010_

_x0010_

_x0010__x0007_

_x0010_
_x0007__x0007__x0007_

_x0007_
_x0007_ _x0007_ _x0007_ _x0007__x0007_

_x0007_

_x0007_

_x0007__x0007_ _x0007_ _x0007_ _x0007_ _x0007__x0007__x0007__x0007_

_x0007_ _x0007__x0007__x0007_ _x0007_ _x0007_

_x0007_ _x0007_ _x0007_ _x0007_

_x0007__x0007_ _x0007_

_x0007__x0007__x0007__x0007__x0007__x0007_

_x0007__x0007__x0007_

_x0007_
_x0007_

_x0007_
_x0007_

_x0010_

_x0012__x0010_

_x0012_

_x0010_

_x0012_

_x0010__x0010_

_x0012_

_x0010__x0016_

_x0010__x0012__x0010__x0019__x001D_#--_x0019_"_x001D_-_x001D__x0016__x0016__x001D__x0019__x001D__x0019__x0012_

_x0012_

_x0012__x0012__x0012__x0012_

_x0010_

_x0010_

_x0010_

_x0007_
_x0007_

_x0007_
_x0007__x0007_

_x0007_ _x0007__x0007_ _x0007_

_x0010_

_x0007_

_x0007__x0007_

_x0007_ _x0007_ _x0007_ _x0007__x0007__x0007__x0007__x0007_
_x0007__x0007_ _x0007__x0007__x0007_ _x0007_ _x0007_
_x0007_ _x0007_ _x0007_ _x0007__x0007_
_x0007__x0007__x0007__x0007_ _x0007__x0007__x0007__x0007__x0007_
_x0007_
_x0007_
_x0007__x0007__x0007__x0007_ _x0007_

_x0007_

_x0007_

_x0007_

_x0007_

_x0012_

_x0010_

_x0010__x0016_

_x0012__x0012__x0019__x0016__x0016__x0016_-_x0016__x0019_-_x0016_-_x0016__x0019__x0016__x0016_-_x0016__x0012__x0010_

_x0016__x0012_

_x0016__x0016_

_x0010__x0010_

_x0010__x0012_

_x0010_

_x0007_
_x0007__x0007_

_x0007_
_x0007__x0007_

_x0007__x0007_

_x0007__x0007_

_x0007__x0007_ _x0007_ _x0007_ _x0007_
_x0007_ _x0007_ _x0007__x0007_ _x0007_ _x0007_
_x0007__x0007__x0007__x0007__x0007__x0007_ _x0007__x0007__x0007_

_x0007_

_x0007_

_x0007_
_x0007__x0007_

_x0007__x0007_

_x0010_

_x0010__x0010_

_x0012_

_x0016__x0010__x0012_

_x0010_

_x0016__x0016_

_x0012__x0012__x0016__x0016__x001D__x001D__x001D__x0019__x001D__x0019__x001D__x001
9__x001D_-_x0016__x001D__x0016__x001D__x0019__x0016__x0010__x0016__x0010_

_x0010__x0010__x0012__x0016__x0012__x0010_

_x0012_
_x0010_

_x0007__x0012_

_x0007_

_x0007_

_x0007__x0007__x0007_
_x0007_

_x0007__x0007__x0007__x0007__x0007_ _x0007__x0007__x0007_
_x0007_ _x0007_ _x0007_ _x0007__x0007_ _x0007__x0007_ _x0007_
_x0007_ _x0007_ _x0007_ _x0007__x0007__x0007__x0007_ _x0007_
_x0007__x0007_
_x0007_
_x0007_ _x0007__x0007_
_x0007__x0007_
_x0007_

_x0007__x0007__x0007__x0007__x0007_

_x0007_

_x0012_

_x0010__x0012_

_x0012__x0012__x0012_

_x0016__x0016_
_x0016__x0010__x0016__x0010__x0010__x0019__x0019__x0016__x0016_

_x0019__x0016__x0019__x0019__x0019__x0019__x001D_-%!_x0016__x0016__x001D__x0016_-_x0016_-#_x0019_
_x0016__x0016__x0010_

_x0012_

_x0016__x0010__x0010__x0010__x0012_

_x0010_

_x0012_

_x0010__x0010__x0010_

_x0007__x0010_

_x0007_

_x0007__x0007_

_x0007_

_x0007__x0007_

_x0007__x0007_

_x0007_

_x0007_

_x0007_ _x0007_ _x0007_
_x0007_ _x0007_ _x0007__x0007_ _x0007_
_x0007_
_x0007__x0007__x0007__x0007__x0007_
_x0007_
_x0007__x0007_

_x0007_ _x0007_

_x0007_ _x0007_
_x0007_ _x0007_
_x0007_

_x0007_

_x0012_

_x0012_ _x0010_

_x0016__x0010__x0016__x0010__x0010__x0012__x0019__x0016__x0012__x0019__x0019__x0012__x0010__x0016__x0019__x0016__x0019_ "_x0019_--_x001D__x0019__x0016__x0019__x0016_

_x0012_
_x0012__x0016__x0010_

_x0016__x0012__x0010__x0010__x0012__x0010__x0012_

_x0010_

_x0010__x0010_

_x0012_

_x0010__x0007_

_x0010_

_x0007_

_x0007_

_x0007__x0007_

_x0007_

_x0007__x0007__x0007__x0007__x0007__x0007__x0007_ _x0007__x0007_
_x0007__x0007_ _x0007_ _x0007_ _x0007_
_x0007_ _x0007_ _x0007_
_x0007__x0007_ _x0007__x0007__x0007_
_x0007__x0007__x0007__x0007__x0007__x0007__x0007_
_x0007__x0007_

_x0007_

_x0007__x0007_
_x0007_

_x0007__x0007_ _x0010_

_x0007_ _x0007_

_x0012_

_x0010__x0012__x0012__x0010__x0016__x0010__x0012__x0012__x0016__x0016_

_x0012__x0010__x0012__x001D_"_x0019_-_x0019__x0019__x001D_-_x0019__x001D_!_x0012__x0
012__x0012__x0010__x0016_

_x0016__x0010__x0012__x0010_

_x0012_

_x0010_

_x0010_

_x0012_

_x0012_

_x0010_

_x0012_

_x0010_

_x0007_

_x0007_
_x0007_

_x0007_ _x0007_

_x0007_

_x0007__x0007_ _x0007__x0007_ _x0007_ _x0007_ _x0007__x0007__x0007_
_x0007_ _x0007_

_x0007_
_x0007__x0007__x0007_
_x0007__x0007_
_x0007_ _x0007__x0007__x0007_

_x0007_ _x0007_
_x0007__x0007_

_x0007_

_x0007_

_x0010_

_x0012__x0012__x0019__x0016__x0019__x0012__x0012__x0016__x0016__x0010__x0019__x0016__x0019__x0016__x0016__x0016__x001D__x001D_--&$!--_x001D_-!_x001D__x0016__x0019_-!_x001D__x0019__x0016_

_x0010__x0010__x0016__x0012__x0016__x0019__x0019__x0016__x0012__x0012_

_x0007__x0007_

_x0007__x0007_ _x0007_

_x0007_
_x0007_

_x0007_ _x0007_

_x0007__x0007__x0007_

_x0007_ _x0007__x0007_ _x0007_ _x0007_ _x0007_
_x0007__x0007_ _x0012_ _x0007__x0007__x0007_
_x0007__x0007__x0007__x0007_ _x0007__x0007_

_x0007_
_x0007_

_x0007__x0007_
_x0007_

_x0007__x0007__x0007__x0007_

_x0007_

_x0007_

_x0007__x0007__x0010_
_x0007_

_x0007_ _x0012_

_x0010_

_x0012__x0016__x0012__x0016__x0016__x0016__x0016__x0010__x0016__x0010__x0016__x0019
__x0016__x0019__x0016__x0016__x0019__x0019_- _x0016_ _x001D_ _x0019_!_x0019_

_x0016_

_x0016__x0010__x0016_

_x0012_

_x0016__x0010__x0012_

_x0010_

_x0010_

_x0010_

_x0007__x0007_

_x0007_

_x0007_

_x0007_

_x0007__x0007_
_x0007_

_x0007__x0007__x0007__x0007_ _x0007__x0007_
_x0007__x0007__x0007_ _x0007_ _x0007_
_x0007__x0007_

_x0007_ _x0007__x0007_

_x0007__x0007__x0007_

_x0007_

_x0007_
_x0007_
_x0007_
_x0007__x0007_

_x0010_

_x0010__x0010_

_x0010_

_x0010__x0019__x0016__x0010__x0012__x0012__x0016__x0019__x0019__x0019__x0016_-_x0019
__x001D__x0019__x0016__x0019__x0019__x001D_-_x001D__x0019_
_x0019__x001D__x0016__x0019__x0012__x0012__x0012__x0019__x0019__x0012__x0012__x0012
_
_x0016__x0016__x0010_
_x0012__x0010_

_x0010_

_x0007_
_x0007_

_x0007_ _x0007_

_x0007_

_x0007__x0007_ _x0007_ _x0007__x0007_ _x0007__x0007__x0007__x0007__x0007__x0007_
_x0007_ _x0007_ _x0007_
_x0007__x0007_ _x0007_ _x0007_ _x0007__x0007__x0007_
_x0007_

_x0007_
_x0007_

_x0007_ _x0007__x0007__x0007__x0007_
_x0007_ _x0007__x0007_
_x0010_

_x0007_

_x0007_

_x0007_

_x0007_

_x0007_

_x0010_

_x0012_

_x0010__x0019__x0016__x0016__x0019__x0016__x0016__x001D_-_x001D__x001D__x0016_-_x0019__x001D__x0016__x0016__x0012__x0016__x0012__x0010__x0012_

_x0016__x0010__x0010__x0012__x0010_

_x0010_

_x0010_

_x0010_

_x0007__x0007_

_x0007_

_x0007__x0007__x0007__x0007__x0007__x0007__x0007__x0007__x0007_

_x0007__x0007_ _x0007_ _x0007__x0007_
_x0007_ _x0007_ _x0007__x0007_

_x0007_

_x0007_
_x0007_

_x0007_

_x0007_

_x0007_
_x0007_

_x0012_

_x0007_

_x0010_

_x0010_

_x0010__x0010__x0012__x0010__x0010__x0012_

_x0019__x0016__x0012_

_x0019__x001D__x0016__x001D_-_x0019_--
_x001D_!%_x0019__x0016__x0016__x0010__x0016__x0012__x0016__x0019__x0012__x0010__x0012__x0016__x0012__x0016__x0012_

_x0012_

_x0012_
_x0007_

_x0007_

_x0007__x0007_

_x0007__x0007_

_x0007__x0007_
_x0007__x0007__x0007__x0007__x0007__x0007__x0007__x0007_ _x0007_ _x0007_ _x0007_ _x0007_ _x0007_ _x0007_ _x0007_ _x0007__x0007_ _x0007_ _x0007__x0007_ _x0012_
_x0007_

_x0007__x0007__x0007__x0007__x0007_

_x0007_

_x0007_

_x0010_

_x0007_

_x0010_

_x0010_

_x0010_

_x0016__x0016__x0010_

_x0019__x0016__x0016__x0019__x0016_-_x0019__x0019__x0016__x001D__x001D__x001D__x001
6__x0019_--_x001D__x0019__x0019__x0016__x0010__x0010__x0010__x0010__x0019_

_x0010_

_x0010_

_x0010__x0012_

_x0007__x0012_

_x0007__x0007__x0007_

_x0007__x0007_
_x0007__x0007__x0007_ _x0007_ _x0007_

_x0007_ _x0007_ _x0007_ _x0007_ _x0007_ _x0007__x0
007__x0007_ _x0007_
_x0007_ _x0007_ _x0007__x0007__x0007__x0007__x0007__x0007_
_x0007__x0007__x0007_
_x0007_

_x0007_
    _x0007_

_x0007_
_x0007_
    _x0007_

_x0007_

_x0007__x0007__x0010_

_x0007_

_x0010_

_x0010_

_x0012_

_x0010__x0016__x0012__x0010__x0010_

_x0012__x0016__x0016__x0019__x0019__x0012_

_x0016__x0019_-_x0019__x0016_

---_x001D__x001D_!_x001D__x0019_-_x0019__x0016__x0019_-_x0016__x0012__x0010__x0016__x0010__x0016__x0019_

_x0016_

_x0010__x0016_

_x0010__x0012_

_x0010__x0012_

_x0010_

_x0010_

_x0012_
_x0007_

_x0007__x0007__x0007_
_x0007_

_x0007_ _x0007__x0007__x0007_ _x0007_ _x0007__x0007__x0007__x0007_
_x0007_ _x0007__x0007_ _x0007__x0007_
_x0007__x0007_ _x0007_ _x0007_ _x0007_

-

_x0007__x0007_ _x0007__x0007__x0007_

_x0007_ _x0007_

_x0007_ _x0007_
_x0007_
_x0007_

_x0007_
_x0007_

_x0010__x0010_

_x0010_

_x0012__x0012_

_x0010__x0010__x0012__x0012__x0019__x0016__x0012__x0012__x001D_-!_x0016__x001D_ !-_x001D__x001D_#_x0016__x001D_!_x001D__x0016__x0016__x0012_

_x0019__x0016__x0016__x0012__x0012__x0016_

_x0010_

_x0010_

_x0012_

_x0012_

_x0007_
_x0007_

_x0007_

_x0007__x0007_

_x0007_

_x0007_ _x0007__x0007__x0007_ _x0007_ _x0007_ _x0007_ _x0007_
_x0007__x0007_
_x0007_ _x0007__x0007__x0007_ _x0007_ _x0007_ _x0007_
_x0007__x0007__x0010__x0007__x0007__x0007__x0007__x0007__x0007_ _x0007__x0007_

_x0007_
_x0007__x0007_

_x0007_

_x0007_ _x0007_

_x0007_
_x0007_

_x0007_

_x0010_
_x0010_

_x0010__x0007_

_x0012__x0012__x0012_

_x0012__x0012__x0012_

_x0019__x0019__x0016__x001D_ "-_x0019_!#_x0019__x0019_-_x001D__x001D__x0019_
_x0019__x001D_--- _x0016_ -_x0016__x0016__x0016_
_x0012__x0010__x0016__x0016__x0012__x0012__x0010__x0010__x0012_

_x0010__x0010_

_x0007_

_x0012__x0010_

_x0007_

_x0007__x0007__x0007_

_x0007_
_x0007__x0007_ _x0007_

_x0007_ _x0007_ _x0007__x0007__x0007__x0007_
_x0007_ _x0007_
_x0007__x0007_ _x0007__x0007__x0007_ _x0007__x0007__x0007_

_x0007_ _x0007_ _x0007__x0007__x0007__x0007__x0007_
_x0007__x0007__x0010__x0007_

_x0007__x0007_

_x0007_

_x0007_

_x0007_ _x0007__x0007_

_x0007_

_x0007_

_x0010_

_x0010_
_x0010__x0010_

_x0010__x0012_

_x0010_

_x0010__x0010_

_x0010__x0010_

_x0012__x0012__x0019__x0019__x0019__x001D_-_x001D_!-_x0019__x001D__x0019_"--_x001D_
-"!_x001D__x0019_ _x0010__x0010__x0010__x0016__x0012__x0010__x0010__x0010_

_x0010_

_x0010__x0012_

_x0010_

_x0012_
_x0010_

_x0012_

_x0007_

_x0007__x0007__x0007_

_x0007_
_x0007_
_x0007_

_x0007_
_x0007__x0007__x0007_
_x0007_
_x0007_
_x0007__x0007__x0007__x0007__x0007__x0007_ _x0007__x0007_ _x0007_ _x0007_

_x0007__x0007_ _x0007_

_x0007_ _x0007__x0007__x0007_

_x001D_"

_x0007__x0007__x0007__x0007__x0007__x0007__x0007__x0007_ _x0007_ _x0007_

_x0007_

_x0007_
_x0007__x0007_

_x0007__x0007_

_x0007_
_x0007_

_x0010_

_x0012__x0010__x0012_

_x0010_

_x0012_

_x0012_

_x0012__x0012__x0010__x0012__x0012__x0019_-_x001D__x0019_!-##_x0019_#_x001D__x0019_
_x0016__x001D__x0019__x001D__x0016__x0016__x0016__x0010__x0012__x0016__x0012_
_x0012__x0012_

_x0007__x0010__x0010_

_x0010__x0012_

_x0007_

_x0007_

_x0007_

_x0007_ _x0007__x0007_

_x0007_

_x0007_ _x0007_ _x0007_
_x0007__x0007__x0007__x0007__x0007__x0007__x0007__x0007__x0007_ _x0007_

_x0007__x0007__x0007_ _x0007_ _x0007_
_x0007_ _x0007__x0007__x0007__x0007_ _x0007_ _x0007_
_x0007__x0012_!_x0016__x0007_ _x0007__x0007_

_x0007_ _x0007_ _x0007__x0007_
_x0007_

_x0007_

_x0007_

_x0007__x0007_

_x0007_

_x0007__x0007_

_x0010_

_x0007_
_x0010_

_x0010_

_x0007_

_x0010_

_x0010_

_x0012__x0016_

_x0010_

_x0007_

_x0007__x0007_
_x0007_

_x0007_

_x0007_
_x0007_
_x0007__x0007__x0007__x0007__x0007__x0007__x0007__x0007__x0007_
_x0007_ _x0007__x0007__x0007_ _x0007_ _x0007_ _x0007__x0007__x0007_
_x0007_
_x0007_ _x0007__x0007_

_x0007_ _x0007__x0007__x0007_

_x0007__x0007_ _x0007_

_x0007_

_x0007__x0007__x0007__x0007__x0007_

_x0007__x0007_

_x0007_
_x0007__x0007_
_x0007_

_x0012_

_x0010_

_x0012__x0012__x0010__x0010__x0010__x0012_

_x0012_#   _x001D_-_x001D__x001D_   _x0019_
-$!-_x0019__x0019__x0019__x0019__x0019__x0016__x0012__x0012__x0016_
_x0010_

_x0012__x0012_
_x0012__x0010_

_x0010_

_x0010_
_x0010__x0010_

_x0012_

_x0007_

_x0007_

_x0007_

_x0007__x0007_
_x0007__x0007__x0007_

_x0007_

_x0007_ _x0007__x0007_ _x0007_ _x0007_ _x0007_
_x0007_
_x0007_ _x0007_ _x0007_ _x0007_

_x001D_
_x0007_ _x0007_

_x0007__x0007_

_x0007__x0007_
_x0007_

_x0007_

_x0007_
_x0007_ _x0007__x0007_

_x0007__x0007_

_x0012_

_x0010__x0010_
_x0012_

_x0010_

_x0010__x0010__x0010_

_x0010__x0010__x0012__x0016__x0016_

_x0012__x0012__x0010__x0019__x0019_

-_x001D_-_x001D__x0019__x001D__x0016_!#_x0019_$_x0019__x001D_!_x0012_

_x0016__x0016_
_x0012__x0010__x0019_

_x0010__x0012_

_x0012__x0012__x0012_

_x0010__x0010_

_x0012_

_x0007_

_x0010_

_x0007__x0007__x0007__x0007_

_x0007__x0007__x0007__x0007__x0007__x0007__x0007__x0007_
_x0007_ _x0007__x0007_ _x0007_ _x0007_ _x0007_ _x0007__x0007_
_x0007_ _x0007__x0007_ _x0007_ _x0007__x0007_ _x0007_

_x0012__x0007__x0007__x0007_ _x0007_
_x0007_ _x0007__x0007__x0007_

_x0007_

_x0007__x0007__x0007_

_x0007_

_x0007_

_x0010_

_x0010_

_x0012__x0012_

_x0010_

_x0019__x0010__x0012__x0016__x0019_-_x0019__x001D__x001D_--#%!!!_x0019_-
-_x001D__x0019_ _x0019__x0016_

_x0016__x0010_

_x0012__x0016__x0012_

_x0010_

_x0012_

_x0012_

_x0010_

_x0010_

_x0010_

_x0010_

_x0007__x0007_

_x0007_
_x0007_

_x0007_
_x0007_
_x0007__x0007_

_x0007__x0007__x0007_ _x0007__x0007__x0007__x0007_ _x0007__x0007_
_x0007_ _x0007_ _x0007_
_x0007_ _x0007_ _x0007_ _x0007__x0007__x0007__x0007_
_x0007_ _x0007_ _x0007_ _x0007_

_x0007_ _x0007_ _x0007__x0007__x0007_

_x0007__x0007__x0007_
_x0007_

_x0007__x0007_

_x0007_

_x0007_

_x0007_

_x0010_

_x0010_

_x0010_
_x0010_

_x0016_

_x0012__x0012__x0010_

_x0010__x0012__x0012__x0016__x0010__x0016__x0019__x0016__x0019_- %_x001D_-! !
-_x0012__x0019__x0016__x0019__x0012__x0012__x0010__x0010__x0010_

_x0012_
_x0010__x0012__x0010_

_x0012__x0012_

_x0010_ _x0012_
_x0010_

_x0010_

_x0007_

_x0007_ _x0007_

_x0007_

_x0007__x0007_
_x0007__x0007__x0007__x0007__x0007__x0007_ _x0007_
_x0007_
_x0007__x0007_ _x0007_ _x0007__x0007_
_x0007_

_x0007__x0007__x0007__x0007_ _x0007_
_x0007__x0007__x0007__x0007__x0007_

_x0007_

_x0007_ _x0007_

_x0007_

_x0007_ _x0007_

_x0010_

_x0016_

_x0010__x0010_
_x0010_

_x0010_

_x0010_

_x0016__x0012_

_x001D__x0012__x0016__x0019__x0019__x0019_-_x001D__x001D_"!-_x001D_-_x001D_
#_x001D__x0019_ _x001D_-

_x0016__x0016__x0012__x0012__x0016__x0012__x0010_

_x0012__x0012_
_x0010_

_x0010__x0010__x0010_
_x0010__x0010_

_x0010_

_x0010_

_x0010_

_x0007_

_x0007__x0007_
_x0007__x0007_ _x0007__x0007_
_x0007_
_x0007_
_x0007__x0007__x0007_ _x0007__x0007_
_x0007__x0007__x0007_ _x0007__x0007__x0007_
_x0007_ _x0007_ _x0007__x0007_ _x0007_ _x0007_ _x0007_
_x0007_

_x0007__x0007__x0007__x0007__x0007_
_x0007__x0007__x0007_
_x0007__x0007_

_x0007_	_x0007_
_x0007_	
	_x0007_
	_x0010_

_x0007_

_x0007__x0007_

_x0010_

_x0016__x0010_

_x0012_

_x0016__x0012_

_x0010_

_x0010_

_x0012__x0012_

_x0016__x0012__x0019__x0016__x001D__x001D__x0019__x0019__x0019_-_x001D_$_x0019__x00
19__x0016__x0016__x0019__x0016__x0016__x0010__x0016__x0010__x0010__x0012__x0016_

_x0010_

_x0012_
_x0012_

_x0012__x0010_

_x0012_

_x0010__x0010_

_x0007_

_x0007__x0007_ _x0007__x0007_

_x0007__x0007__x0007__x0007__x0007_

_x0007__x0007_
_x0007__x0007_
_x0007_

_x0007__x0007_ _x0007_ _x0007__x0007__x0007_
_x0007__x0007__x0007_ _x0007_

_x0007__x0010__x0007_ _x0007_

_x0007__x0007__x0007_
_x0007_

_x0007_ _x0007_

_x0007_

_x0007_

_x0010_

_x0010_

_x0010_

_x0012_

_x0012_
_x0012_

_x0010__x0010_

_x0012__x0019__x0019_

_x0010__x0016__x0019__x001D_#---_x001D_

-_x001D__x0016__x0019__x0019__x0019__x0016__x0016__x0012__x0012__x0016__x0012__x0012__x0016_

_x0010_

_x0012_
_x0010_

_x0012__x0012_

_x0012_

_x0007_

_x0010_

_x0007_

_x0007_

_x0007_
_x0007_

_x0007_
_x0007_
_x0007_

_x0007__x0007__x0007_
_x0007_ _x0007__x0007__x0007_
_x0007_ _x0007__x0007__x0007__x0007_
_x0007__x0007_ _x0007_
_x0007__x0007_ _x0007_ _x0007__x0007__x0007_
_x0007__x0007__x0007__x0007__x0007__x0007__x0007_ _x0007_
_x0007__x0007__x0007__x0007__x0007_
_x0007__x0007_

_x0007__x0007_
_x0007_

_x0007_

_x0007_

_x0010_

_x0007_

_x0007_

_x0010_

_x0010__x0012_

_x0016_
_x0010_

_x0016__x0012_

_x0016__x0010__x0012__x0019__x0019__x0019__x0019__x001D_ -_x001D_
_x001D__x001D_"-_x001D__x0019__x0019__x0019__x0019_ _x0012__x0012__x0019__x0010_

_x0010_

_x0010__x0010_

_x0010_
_x0010__x0007_

_x0010_

_x0010_

_x0012__x0007_
_x0007_

_x0007_ _x0007_

_x0007_

_x0007_
_x0007__x0007_
_x0007__x0007__x0007_ _x0007__x0007__x0007__x0007__x0007__x0007_

_x0007__x0007__x0007_ _x0007__x0007_ _x0007_

_x0007_
_x0007_ _x0007_
_x0007_ _x0007__x0007__x0007__x0007__x0007_ _x0007__x0007_
_x0007__x0007__x0007__x0007_
_x0007__x0007__x0007_
_x0007_

_x0007__x0007_

_x0007_

_x0007__x0010_

_x0007_

_x0010__x0010_

_x0012_

_x0012__x0016__x0016_

_x0016__x0019__x0019__x001D_---_x001D_%_x0019__x0019__x001D_-_x0019__x0012__x0016__x0012__x0016__x0012__x0016__x0019__x0016__x0016_

_x0012_

_x0010_

_x0010__x0012_

_x0010__x0016_

_x0010_

_x0010_

_x0007_

_x0007__x0007__x0007__x0007_

_x0007__x0007__x0007_

_x0007_
_x0007_

_x0007__x0007_ _x0007__x0007__x0007_

_x0007__x0007__x0007_ _x0007_ _x0007_ _x0007_ _x0007__x0007_
_x0007_

_x0007_ _x0007_ _x0007_
_x0007__x0007_ _x0007__x0007_ _x0007_

_x0007__x0007__x0007_ _x0007_ _x0007__x0007_
_x0007__x0007_
_x0007__x0007_
_x0007_

_x0007_

_x0012_

_x0007_
_x0007_

_x0007__x0010_

_x0007_

_x0012__x0010_

_x0012_

_x0012_

_x0010__x0012_

_x0012__x0010__x0012__x0012__x0010__x0010__x0010__x0012__x0010__x0016_-"-%"_x001D__x0019__x0019__x0019__x0019__x001D__x001D__x0019__x0016__x0010__x0012__x0010__x0010__x0012_

_x0010_

_x0010_

_x0010_

_x0010_

_x0007_
_x0007_

_x0007_

_x0007__x0007__x0007__x0007__x0007_ _x0007_ _x0007__x0007__x0007_

_x0007__x0007__x0007__x0007__x0007_

_x0007__x0007__x0007__x0007__x0007__x0007_ _x0007__x0007__x0007_

_x0007_

_x0007_
_x0012__x0007_ _x0007_ _x0007_ _x0007__x0007__x0007__x00
07__x0010_ _x0007__x0007__x0007__x0007__x0007_
_x0007__x0007_ _x0007__x0007_
_x0007_ _x0007__x0007__x0007_ _x0007_

_x0007_

_x0010_

_x0010__x0010_

_x0010__x0010_

_x0012_

_x0016__x0016__x0010__x0010__x0019_-_x001D__x001D__x0019__x0016__x0019_"$--!_x001D_
-_x0016__x0019__x0012__x0019__x0019__x0016_

_x0010__x0016__x0016__x0010__x0012_

_x0010_

_x0012_
_x0010_
_x0012_

_x0010__x0016__x0010_

_x0010_

_x0010__x0012_

_x0012__x0012__x0010__x0016__x0012__x0012__x0010_

_x0019__x0012__x001D_ _x001D__x0019__x001D_$#$_x001D__x001D_--!_x0016_

_x0016__x0012__x0010_

_x0010_

_x0012__x0010_

_x0010_

_x0010_

_x0012__x0012_

_x0012_

_x0010_
_x0007_
_x0007_

_x0007__x0007__x0007_ _x0007__x0007_

_x0007_
_x0007__x0007_ _x0007_ _x0007__x0007_
_x0007__x0007__x0007__x0007_ _x0007__x0007__x0007__x0007_ _x0007_ _x0007_
_x0007_ _x0007_

_x0007_ _x0007_ _x0007_ _x0007_ _x0007__x0007_ _x0007__x0007_

_x0007__x0007__x0007__x0007_

_x0007__x0007_

_x0007_

_x0010_

_x0010__x0010__x0010_

_x0012_

_x0012_

_x0010__x0010_

_x0019__x0012__x0016__x001D_-#_x0019_"!-"_x0019_ _x001D__x0016_-_x0016_
_x0019__x0012__x0016__x0010__x0019__x0012__x0010_
_x0012__x0012_

_x0012_

_x0010_

_x0010_

_x0012_

_x0010_

_x0010_

_x0007_

_x0007_

_x0007_

_x0007_

_x0007__x0007_ _x0007__x0007_

_x0007__x0007__x0007_
_x0007__x0007_ _x0007__x0007__x0007__x0007__x0007_
_x0007_ _x0007_ _x0007_ _x0007_ _x0007_ _x0007_
_x0007_ _x0007__x0007_ _x0007_ _x0007_
_x0012_ _x0007__x0007_ _x0007__x0007_

_x0007__x0007_
_x0007__x0007_ _x0007_
_x0007_

_x0007_
_x0007_ _x0007_ _x0010_

_x0007_

_x0010_

_x0010__x0012__x0010_

_x0010__x0010_

_x0012_

_x0012__x0012_

_x0019__x0016__x0019__x0012__x0016__x0010__x001D__x001D_----"_x0019_"!_x0019__x001D_
$-_x0016__x0012__x0016__x0016__x0016__x0016__x0010_

_x0012_

_x0007_

_x0007__x0010_

_x0007__x0007_

_x0007__x0007_

_x0007__x0007__x0007_ _x0007_
_x0007__x0007__x0007__x0007__x0007__x0007_
_x0007__x0007__x0007_ _x0007_ _x0007_

_x0007_ _x0007__x0007__x0007_ _x0007_ _x0007_

_x0012__x0007__x0007__x0007__x0007__x0007__x0007_

_x0007_ _x0007__x0007__x0007__x0007_
_x0007_
_x0007_

_x0007_ _x0007_

_x0007_
_x0012_

_x0007_

_x0010_

_x0012__x0010__x0012__x0012_

_x0012__x0016__x0016__x0016__x0019_-_x0019__x0019_"-_x001D_&_x001D_-_x0016__x0019_-_
x0016__x0019__x0012__x0016__x0012__x0012_

_x0012_

_x0010_

_x0012__x0016_

_x0016_

_x0010_

_x0010_

_x0010__x0012__x0010__x0012__x0010_

_x0010_
_x0007_

_x0007_
_x0007_
_x0007_

_x0007__x0007__x0007__x0007_

_x0007_ _x0007__x0007_ _x0007_
_x0007_

_x0007__x0007__x0007__x0007__x0007__x0007_ _x0007__x0007__x0007_
_x0007_ _x0007_ _x0007_ _x0007_

_x0007_ _x0007_ _x0007_ _x0007_ _x0007_

_x0007_ _x0007_

_x0007__x0007__x0007__x0007__x0007__x0007__x0007__x0007_
_x0007_

_x0007_

_x0012_ _x0007_
_x0007_

_x0016_

_x0010_

_x0012_
_x0010_

_x0012_ _x0012_

_x0012__x0010_

_x0012__x0012__x0010__x0016__x0016_

_x0019__x0012__x0019__x0016__x0012__x0019__x001D__x001D_- !$#-#!_x001D__x0019__x0019_ _x0016__x0019__x0016_

_x0016__x0016_

_x0016__x0016__x0016_

_x0010__x0012_

_x0012__x0010_

_x0012_

_x0012_

_x0010__x0016__x0010_

_x0012_
_x0007_
_x0007_

_x0010_

_x0010__x0010_

_x0012_

_x0012_
_x0007_

_x0010__x0010_

_x0016__x0016__x0012__x0016_

_x0019__x0016__x0019__x001D__x0019__x0019_ $"-!_x001D_
#_x001D__x0016_-_x0019__x0012__x0010__x0016__x0010__x0012_

_x0010_

_x0010_
_x0010_
_x0012__x0010__x0010_

_x0010__x0010_

_x0010__x0010_

_x0010_

_x0007_

_x0007_

_x0007__x0007__x0007_
_x0007_ _x0007__x0007__x0007_ _x0007__x0007_
_x0007_
_x0007_
_x0007__x0007__x0007__x0007__x0007__x0007__x0007__x0007_ _x0007_ _x0007_ _x0007__x0007_ _x0007__x0007__x0007__x0007_
_x0007_ _x0007__x0007_ _x0007__x0007_ _x0007_

_x0007_ _x0007__x0007_ _x0007_ _x0007_ _x0007_ _x0007__x0007_

_x0007__x0007_
_x0007__x0007__x0007__x0007_ _x0007_

_x0010__x0012_

_x0010_

_x0010_

_x0012_

_x0010_

_x0010__x0010__x0010_

_x0012_

_x0010_
_x0012_

_x0010__x0012__x0012__x0012_

_x0012__x0010__x001D_

_x0012__x0012__x0012__x0016__x0019_ _x001D_ -!--_x001D__x0019__x001D_

_x0016_
_x0010__x0019__x0012_
_x0010_

_x0010__x0012__x0012_

_x0010_
_x0012_

_x0010__x0012_

_x0010_

_x0007_

_x0007__x0007_
_x0007__x0007__x0007__x0007_
_x0007__x0007_ _x0007_
_x0007_

_x0007_
_x0007__x0012__x0012_ _x0007_ _x0007__x0007_ _x0007_ _x0007_

-_x0019_ _x0007__x0007__x0007__x0007_

_x0007__x0007_ _x0007__x0007_ _x0007_

_x0007_
_x0007__x0007_
_x0007_
_x0007__x0007__x0007_

_x0010_

_x0012_

_x0007__x0007__x0007__x0007_

_x0007__x0007_

_x0010_
_x0010__x0012_

_x0010_

_x0012__x0012__x0010__x0010__x0010__x0012_

_x0012_

_x0012__x0019__x0012__x0010__x0010__x0019__x0016__x001D__x0016__x001D__x001D_%_x001D_

_x001D_%_x001D_-_x0019__x001D__x0016__x0019__x0016__x0016__x0016__x0016__x0012__x0010_

_x0010_
_x0010_

_x0010__x0012_

_x0010_

_x0007_

_x0007_ _x0007_

_x0007_
_x0007__x0007__x0007__x0007_ _x0007_
_x0007_ _x0007__x0007__x0007_ _x0007_

_x0007_ _x0007__x0007__x0007__x0007__x0007__x0010_ _x0007_ _x
0007_ _x0007_

_x0007_ _x0007_ _x0007__x0007__x0007__x0007__x0007_ _x0007_
_x0007_

_x0007_

_x0007_

_x0007_

_x0010_

_x0010_

_x0010_ _x0012__x0010_

_x0010__x0010_

_x0010__x0012_

_x0016__x0010_

_x0010_

_x0010__x0016__x0019_-

_x0019__x0019_-_x0019_"$#_x0019_!-_x001D_#-_x001D__x0016__x001D__x0019__x001D__x0016__x0016_

_x0016__x0016__x0016__x0019__x0012__x0012__x0010_

_x0010_

_x0010_

_x0010_
_x0010_

_x0010_

_x0007_

_x0007_

_x0007__x0007_ _x0007_

_x0007__x0007_ _x0007__x0007__x0007__x0007__x0007_
_x0007__x0007__x0007__x0007_ _x0007_
_x0007__x0007_ _x0007__x0007__x0007__x0007_
_x0010__x0007__x0007__x0007_ _x0007_ _x0007_ _x0007_

_x0007_ _x0007_ _x0007__x0007__x0007__x0007__x0007_ _x0007_ _x0007__x0007_
_x0007_

_x0007_
_x0007_

_x0010_

_x0007_
_x0007__x0007_

_x0012_

_x0010__x0010__x0010__x0010_

_x0007_

_x0010_

_x0012_

_x0016__x0012__x0016__x0012_
_x0010__x0012__x0012_

_x0012__x0010_

_x0016__x0016__x0016_ ! "
_x0019__x001D__x001D_--_x001D__x001D_!_x0016__x0016__x0016__x0012_
_x0019__x0019__x0012_

_x0012_

_x0012_

_x0012__x0012_
_x0010_

_x0012_

_x0007_

_x0007__x0007__x0007__x0007_

_x0007_
_x0007_

_x0007__x0007_ _x0007__x0007__x0007__x0007__x0007_
_x0007__x0007_ _x0007_ _x0007_ _x0007__x0007__x0007__x0007__x0007_

_x0007_ _x0007__x0007_
_x0007_ _x0007_ _x0007_ _x0007_ _x0007_

_x0007__x0007__x0007__x0007_ _x0007_ _x0007__x0007__x0007__x0007_

_x0007_

_x0007__x0007_

_x0007_

_x0007__x0007_

_x0010_

_x0007_

_x0007_

_x0010_

_x0010_

_x0012__x0012_

_x0012__x0010__x0012__x0010_

_x0010__x0012_

_x0010__x0019_

_x0016__x0019_

_x0016__x001D__x0019__x0019_-$!-!_x001D_-_x0016_--_x0019__x0016__x0019__x0010__x001
2__x0019__x0012__x0016_

_x0012__x0010_

_x0010_

_x0012_

_x0010_

_x0007_

_x0007_

_x0007_
_x0007_

_x0007_ _x0007__x0007_ _x0007_ _x0007_

_x0007__x0007_

_x0007_ _x0007_

_x0007_ _x0007_ _x0007__x0007__x0007__x0007_ _x0007_
_x0007__x0007__x0012__x0007_ _x0007_ _x0007_
_x0007_ _x0007_ _x0007_
_x0007__x0007_

_x0007_ _x0007_ _x0007__x0007__x0007_
_x0007__x0007__x0007__x0007_

_x0007__x0007_
_x0007_ _x0007__x0007__x0007_

_x0007_

_x0007_ _x0007_

_x0007_

_x0007_

_x0007_

_x0010_

_x0007__x0007_

_x0010_

_x0007_

_x0010__x0010_

_x0010_

_x0019__x0010_

_x0010__x0010__x0012_

_x0010__x0012__x0012__x0016__x0016__x0019__x001D__x001D_ --!
_x0016__x0016__x0016__x0016__x0012__x0019__x0012__x0010_

_x0012_
_x0010_

_x0010__x0012_
_x0012__x0016__x0019_

_x0010_
_x0007_

_x0007_

_x0007_
_x0007__x0007_

_x0007_ _x0007__x0007_ _x0007__x0007_ _x0007_ _x0007__x0007__x0007_
_x0007_ _x0007_ _x0007_
_x0007_ _x0007__x0007__x0007__x0007__x0007_ _x0007__x0007__x0012_ _x0007_
_x0007_
_x0010_#$_x0007__x0007_
_x0007__x0007_
_x0007_ _x0007__x0007__x0007__x0007__x0007_
_x0007__x0007_

_x0007__x0007__x0007_

_x0012_

_x0012_

_x0012_
_x0012_

_x0016__x0012__x0016__x0019__x0010_

_x0016__x0019__x0016__x001D_!"! _x0019__x0019__x0012__x0016__x0019_

_x0019__x0012__x0012__x0012__x0016__x0010__x0010__x0010__x0016_
_x0010_

_x0010__x0010__x0010__x0010__x0012__x0010__x0012__x0012__x0010__x0010_

_x0007_

_x0007_

_x0007_ _x0007_ _x0007_ _x0007_
_x0007_ _x0007_
_x0007__x0007_ _x0007__x0007__x0007_ _x0007__x0007__x0007_
_x0007_
_x0007__x0007_ _x0007_ _x0007_ _x0007_ _x0007_

_x0007_ _x0007_ _x0007_

_x0007_

_x0007_ _x0007__x0007__x0007_ _x0007_ --
_x0007_
_x0007__x0007_ _x0007_

_x0007__x0007_ _x0007_

_x0007__x0007__x0007__x0007_
_x0007__x0007_

_x0007_

_x0012_

_x0012__x0010_

_x0010_

_x0007_

_x0010__x0010_

_x0016__x0012_
_x0012_

_x0012__x0012_

_x0010_

_x0012_

_x0016_
_x0012__x0010__x0016__x001D__x001D_-_x0019__x001D__x0019__x0016__x0019__x0016__x0016__x0012__x0016__x0016__x0012__x0010__x0012_

_x0012_

_x0012_

_x0010_

_x0012_

_x0012_

_x0007_

_x0007_

_x0007_

_x0007_
_x0007_

_x0007_

_x0007_

_x0007__x0007__x0007__x0007__x0007__x0007__x0007_

_x0007_ _x0007_ _x0007_ _x0007__x0007_ _x0007__x0007__x0007_

_x0007_ _x0007__x0007__x0007__x0007__x0007__x0010_ _x0007__x0007__x0007_
_x0007_ _x0007_ _x0007__x0007__x0007_5_x0007_
_x0007__x0007__x0007__x0007_
_x0007__x0007_

_x0007_ _x0007__x0007_

_x0007__x0007_
_x0007_
_x0007_

_x0007_

_x0007_

_x0010_

_x0012_

_x0012__x0010_

_x0012__x0010__x0010_

_x001D__x001D_-_x001D__x001D_!_x0010_-

_x0016__x0010__x0019__x0012__x0010__x0012__x0010__x0016__x0010_

_x0010_
_x0010__x0010_

_x0007__x0007_

_x0007__x0007_
_x0007_

_x0007_

_x0007_

_x0007__x0007__x0007__x0007_
_x0007_
_x0007__x0007__x0007__x0007_
_x0007_ _x0007_
_x0007_ _x0007_ _x0007_ _x0007__x0007_ _x0007_ _x0007__x0007__x0
007_
_x0010__x0016_ _x0007__x0007__x0007__x0007_ _x0007_
%_x0019_ _x0007__x0007__x0007_
_x0007__x0007__x0007_ _x0007_ _x0007__x0007_
_x0007_
_x0007_

_x0007_
_x0007_

_x0007_
_x0007_

_x0007__x0010__x0010_

_x0010_

_x0010_

_x0010_

_x0012__x0010_

_x0012__x0016__x0010__x0012__x0012__x0012__x0016_

_x0010__x0010_

_x0016__x0019__x0019__x0016__x0019_"%"-_x001D_-#_x0019_-_x001D__x0012__x0016__x0012
__x0012__x0016__x0016__x0010_

_x0010_

_x0010__x0010_

_x0010_

_x0007__x0007__x0007_

_x0007_
_x0007_

_x0007_

_x0007_

_x0007__x0007_

_x0007__x0007__x0007__x0007__x0007__x0007_
_x0007_ _x0007__x0007__x0007_
_x0007_

_x0007_ _x0007_
_x0007_
_x0007_ _x0007__x0007__x0007_ "
_x0007__x0007_ _x0007__x0007__x0007__x0007_

_x0007__x0007_
_x0007__x0007_
_x0007_

_x0007_
_x0007_
_x0007_

_x0010_

_x0010_

_x0010__x0012_

_x0012_

_x0010__x0012__x0012_

_x0016__x0016__x0010__x0012_

_x0019__x0012__x0012__x0016__x0012__x0010__x0019__x0012__x0012__x0012_!--_x001D__x0016_-_x0012__x0016__x0010__x0016__x0016__x0016_

_x0016__x0019__x0016__x0010_

_x0012__x0010_

_x0010__x0010_

_x0010_

_x0012__x0012_

_x0012_

_x0007_

_x0007__x0007_
_x0007_

_x0007__x0007__x0007_

_x0007_ _x0007__x0007__x0007_ _x0007__x0007__x0007_
_x0007__x0007__x0007__x0007__x0007_
_x0007_ _x0007_ _x0007_ _x0007_ _x0007_ _x0007_
_x0007_ _x0007__x0012_

_x0007_ _x0007__x0007__x0007__x0010_-_x0007__x0007_

_x0007_
_x0007_
_x0007__x0007__x0007_
_x0007__x0007_

_x0007_

_x0007_

_x0007__x0010__x0010_

_x0010__x0007_

_x0010_

_x0010_

_x0010_

_x0012__x0010__x0016__x0010__x0019__x0016_

_x0012_-_x001D__x0016__x0019__x0019__x0012__x0010__x0012__x0012__x0019__x0010__x0016__x0012__x0019__x0010__x0012__x0012_

_x0010_

_x0010_

_x0010_

_x0012_

_x0010_

_x0007_

_x0007_

_x0007__x0007_

_x0007__x0007_

_x0007_

_x0007_

_x0007_ _x0007_

_x0007__x0007__x0007__x0007__x0007_

_x0007_ _x0007_
_x0007__x0007__x0007__x0007_
_x0007__x0007_ _x0007__x0007_ _x0007__x0007_ _x0007_

_x0007__x0007_ _x0007_

_x0007_ _x0007_ _x0007_
_x0019_
_x0007__x0007__x0007__x0007_
_x0012_!_x001D_

_x0007__x0007__x0007__x0007__x0007__x0007__x0007_ _x0007_
_x0007__x0007_
_x0007_

_x0007_
_x0007__x0010_
_x0007_

_x0007_

_x0012__x0007_

_x0010_

_x0016__x0012__x0012_

_x0016__x0012__x0019__x0019_--_x0016_-"_x0019__x0016_

_x0012__x0012__x0016__x0019__x0016__x0012__x0010__x0010_

_x0012__x0010__x0010__x0012_

_x0007_

_x0007_
_x0007_

_x0007__x0007_

_x0007__x0007__x0007__x0007__x0007__x0007_ _x0007_
_x0007_ _x0007_ _x0007__x0007_
_x0007__x0007_
_x0007_ _x0007_ _x0007__x0007__x0007_ _x0007_ _x0007_ _x0010_

_x0007__x0007_ _x0007_ _x0007_ _x0007_

_x0007__x0007__x0007_

%! _x0016__x0012_

_x0010__x0012__x0010_
_x0007__x0007_ _x0007__x0007__x0007_ _x0007__x0007__x0007_
_x0007_
_x0007_
_x0007__x0007_
_x0007_ _x0007_

_x0012_

_x0010_

_x0010_

_x0010_

_x0016__x0010__x0012__x0016__x0019__x0016__x0019__x0012__x001D__x0016__x0016__x0019_
_x001D_ -
_x0016__x0016__x0016__x0016__x0019__x0016__x0010__x0016__x0016__x0019__x0016_

_x0012_

_x0016__x0012__x0010_

_x0012_
_x0012__x0010_
_x0010__x0010__x0012_

_x0007_
_x0007_

_x0007_

_x0007__x0007_
_x0007__x0007_

_x0007_ _x0007_

_x0007_
_x0007_ _x0007_ _x0007__x0007_ _x0007__x0007__x0007_ _x0007_
_x0007_

_x0007_ _x0007_

_x0007_ _x0007__x0007_ _x0007__x0007_ _x0007__x0007_
_x0007__x0007_
_x0007__x0007__x0007__x0007_
_x0012_.PdYH@620"!_x001D_%$-43/
_x0007__x0007_ _x0007__x0007_

_x0007_ _x0007_

_x0007_
_x0010_

_x0010_

_x0010_

_x0012_

_x0010__x0012_

_x0012__x0016_!_x0016__x0012__x0010_-_x0019_#-!_x0016__x001D_-_x0012__x0016__x0010__x0012__x0012_

_x0012__x0010_

_x0016__x0012_

_x0010_
_x0010_

_x0012_

_x0010_

_x0012_

_x0007_

_x0007_

_x0010_

_x0010_

_x0010_
_x0010_

_x0012_

_x0012_

_x0010__x0012__x001D__x0016__x0016__x0016__x0019__x0016_#$_x0019_
_x001D__x001D__x0019__x0016__x0019__x0010__x0012__x0012_

_x0010__x0012__x0012_

_x0010__x0012_

_x0012_

_x0010_

_x0010_

_x0010_

_x0007_

_x0010_

_x0007_

_x0007_

_x0010_

_x0016_

_x0007_

_x0007__x0007_
_x0007_
_x0007_
_x0007__x0007_ _x0007_ _x0007_
_x0007_ _x0007_ _x0007_ _x0007_ _x0007_

_x0007_
_x0007_ _x0007__x0007__x0007_ _x0007_ _x0007__x0007__x0007__x0007_ _x0007_
:u]@.25)+("'+3>R1%

_x0007__x0007_ _x0007_ _x0007__x0007_

_x0007_

_x0007_

_x0007_

_x0010_
_x0010_

_x0010_

_x0012_

_x0012_
_x0010_

_x0012__x0010__x0012_

_x0010__x0016__x0010__x0010__x0012__x0012__x0016__x0016__x0019_-_x0016_-
_x001D__x001D_-_x001D__x0019__x0019__x0016_

_x0019__x0016__x0016_

_x0019__x0012__x0010_

_x0010_

_x0010_

_x0010_

_x0010__x0010_

_x0007__x0007_

_x001D_6>),C@>70+#+8SYNJ'

_x0007_ _x0007__x0007__x0007__x0007_
_x0007__x0007__x0007__x0007__x0007__x0007__x0007_ _x0007_

_x0007_ _x0007_

_x0007_ _x0007__x0007__x0007_ _x0007_ _x0007__x0007__x0007__x000
7__x0007__x0007__x0007__x0010__x0007_
'?E_x0016__x0019_

_x0010__x0010_$:0
_x0007__x0007_

_x0007_

_x0007_

_x0010_

_x0010_

_x0010_

_x0012_

_x0010__x0016__x0010__x0016__x0010_

_x0012__x0016__x0016_-_x0019__x0012__x001D__x0016__x001D__x0016_-_x001D__x0019_-$-_x
0019__x0016__x0019_-_x0019__x0016__x0016_

_x0019__x0010__x0010_

_x0012_
_x0012_

_x0012_

_x0010_

_x0007_
_x0007__x0007_

6ciOchVfc]K9>e 晻皤)

_x0007_
_x0007__x0007__x0007__x0007_
_x0007__x0007__x0007__x0007_ _x0007__x0007__x0007_ _x0007_
_x0007_ _x0007__x0007_ _x0007_
_x0007_ _x0007_ _x0007_ _x0007_ _x0007__x0007__x0007_

_x0007_ _x0007_
_x0007_ _x0007__x0007__x0010_
/+
_x0007_
_x0007__x0007_
_x0016_+._x0010_
_x0007_
_x0007_
_x0007__x0007__x0007__x0007_ _x0007_
_x0007_

_x0007__x0007_
_x0007_

_x0012_

_x0010_

_x0007_

_x0012__x0019_

_x0010__x0012__x0010__x0010__x0016__x0016__x0012__x0019__x0012__x0019__x0016__x001D__x0019__x0019_!-!"-_x0012__x0010__x0016__x0019__x0016__x0019__x0012__x0016__x0012__x0016_

_x0010_

_x0010__x0010_

_x0007_

_x0007_

_x0007_
_x0007_
_x0007_'YpHHNKONBE92LhwhW._x0007_
_x0007__x0007__x0007_
_x0007_
_x0007__x0007__x0007__x0007__x0007__x0007__x0007_ _x0007_ _x0007_ _x0
007_

_x0007__x0007__x0007__x0007_ _x0007_ _x0007__x0007_ _x0007__x0007_
_x0007_ _x0007_ _x0007_

_x0007_21
_x0007_

_x0019_+"

_x0007_
_x0007_
_x0007__x0007_ _x0007_
_x0007__x0007__x0007_
_x0007__x0007_
_x0007__x0007_

_x0007_

_x0007_

_x0007__x0007_

_x0007_

_x0010_

_x0012_

_x0012_

_x0012_
_x0012_

_x0010__x0012__x0019__x0012__x0010__x0010_

_x0012__x0016__x0012__x0012_-_x0016__x001D__x001D__x0019_-_x001D_!_x0019__x0019__x0016_-_x0016__x0016__x0016__x0016__x0012__x0016__x0016__x0016__x0019_

_x0010__x0016_

_x0012_

_x0010_

_x0007_ _x0007_ _x0007_

_x0007__x0007_

$,83'#&)*((!!%2;10
_x0007_
_x0007__x0007_ _x0007_
_x0007_ _x0007__x0007__x0007_

_x0007__x0007_ _x0007_
_x0007_

_x0007_ _x0007_
_x0007_

(<_x0016__x0007__x0007_

_x0016_!!   _x0007__x0007_
_x0007__x0007_

_x0007__x0007_

_x0007_

_x0010_

_x0012__x0016_

_x0012__x0010__x0012__x0016__x0019__x0019__x0010__x0016__x0019__x0019__x0012_-

_x0019__x0019__x0019__x0019__x0012__x001D__x0016__x0016_

_x0012_
_x0010__x0010__x0010__x0007_

_x0012__x0012_

_x0012__x0010_

_x0010__x0010__x0007_

_x0007_

_x0007_ _x0007_

_x0010__x0012__x0010__x0012__x0010_

_x0007__x0007_ _x0007__x0007__x0007__x0007_ _x0007_ _x0007__x0007__x0007_
_x0007_ _x0007__x0007__x0007_
_x0007__x0007__x0007__x0007__x0007_ _x0007__x0007__x0007_
_x0007_
_x0010_ _x0007_
_x0007_ _x0007_
_x0007_

_x0007_

_x0007__x0007__x0010_

_x0007_ _x0007__x0007_ _x0007__x0007_ _x0007_ _x0007_
"5_x0019__x0007_
-
_x0007__x0007__x0007__x0007_
_x0007_

_x0007__x0007_ _x0007__x0007_

_x0007_

_x0010_

_x0010_

_x0012__x0016__x0016_--_x001D_-_x0016__x0019__x0016_-_x0016__x0010__x0010_

_x0010__x0012_

_x0010_

_x0010_

_x0007_

_x0007__x0007_

_x0010_

_x0007_ _x0007__x0007__x0007_

_x0007__x0007_ _x0007__x0007_ _x0007_

_x0007__x0007_

_x0007__x0007_

_x0007__x0007__x0007__x0007__x0007_

_x0007_

_x0007_

_x0007_

_x0007__x0007_

5#_x0007__x0007__x0007_

_x001D_%

_x0007_ _x0007__x0007__x0007_
_x0007__x0007__x0007_ _x0007_ _x0007_ _x0007_

_x0010_

_x0010_

_x0010_

_x0012__x0010_

_x0016_

_x0010__x0012__x0016__x0016__x0016__x0019__x0012__x0019__x0016_

_x0016_ -_x001D__x0016__x0016__x0010__x0016__x0016__x0016__x0019__x0016__x0010_

_x0016_
_x0012__x0012_

_x0012_
_x0010__x0010_

_x0010_

_x0010_

_x0007__x0007_

_x0007_

_x0007_

_x0007_

_x0007_
_x0007_ _x0007__x0007_ _x0007__x0007_
_x0007_

_x0007_

_x0007_ _x0007__x0007_

_x0007_

_x0007_

_x0007_
_x0007__x0007__x0007__x0007_ _x0007__x0007_
_x0007_

_x0007_9&_x0007_ _x0007__x0007_

_x0019_'

_x0007__x0007__x0007__x0007_

_x0007_

_x0007_

_x0010_

_x0010_

_x0010_

_x0010_

_x0010_

_x0012__x0016__x0016__x0016__x0012__x0012__x0012__x0012__x0016__x0016__x0016__x0019__x0016__x0019__x0016_
-_x001D__x0019__x0012__x0019__x0019__x001D__x0016__x0019__x0012__x0012_

_x0012__x0012_

_x0010_

_x0012__x0010__x0010__x0012_

_x0007_
_x0007_

_x0016_

_x0007_
_x0010_

_x0007__x0007__x0007_ _x0007__x0007__x0007_ _x0007_

_x0007_

_x0007__x0007_

_x0007_

_x0007__x0007__x0007_

_x0007__x0007_

_x0007_
_x0007_ _x0007_
_x001D_1*

,#

_x0007_

_x0012_

_x0010_

_x0010__x0010_

_x0010_

_x0010_

_x0010_

_x0012__x0019__x0019__x0010__x0016__x001D__x0019__x0019__x0016__x0019__x0016__x0016
__x0016__x0016__x0010_

_x0010_

_x0012__x0012_

_x0012__x0012__x0012_

_x0010_

_x0007_ _x0007__x0007__x0007_ _x0007__x0007_

_x0016_

_x0007_

_x0007_
_x0007__x0007__x0007__x0007_
_x0007_ _x0007__x0007__x0007_ _x0007__x0007__x0007_
_x0007__x0007_
_x0007__x0007__x0007_
_x0007__x0007__x0007__x0007_
_x0007__x0007_ _x0007_
_x0007_ _x0007_ _x0007__x0007__x0007_

_x0007_ _x0010_ _x0007__x0007_ _x0007__x0007_

_x001D_6$_x0007__x0007_

'&

_x0007__x0007_

_x0007_ _x0007_

_x0010_

_x0010_
_x0012_

_x0016_

_x0010_

_x0010_

_x0010__x0019__x0012__x0012_

_x0019__x0012__x0019__x0012__x0019__x0019__x001D__x001D__x0019__x0019__x0016__x0016__x0012__x0019__x0016_

_x0012__x0010_

_x0016__x0010_

_x0016__x0012_

_x0010_

_x0010__x0012_

_x0010_

_x0010_

_x0007_
_x0007_

_x0007__x0007_
_x0007__x0007_
_x0007_

_x0019_

_x0007_

_x0007__x0007__x0007__x0007_ _x0007_ _x0007__x0007_ _x0007__x0007_

_x0007_

_x0007_ _x0007__x0007_
_x0007__x0007_

_x0007__x0007_

_x0007_

_x0007_ _x0007__x0007_
_x0007__x0007_ _x0007_ _x0007__x0007__x0007__x0007_

/&
_x0007_

_x0016_!

_x0007_

_x0007_
_x0007_
_x0007__x0007_

_x0010_

_x0012_

_x0012_

_x0012_

_x0012_

_x0019__x0016__x0016__x0012__x001D_"!_x0016__x0012__x0016__x0012__x0012__x0016__x0010_

_x0010__x0012_

_x0007_

_x0007_ _x0007_ _x0007__x0007_ _x0007_

_x0007__x0007_ _x0007_

_x0007__x0007__x0007_
_x0007_

_x0012_

_x0007_

_x0007_
_x0007_ _x0007_ _x0007_ _x0007_ _x0007_ _x0007_
_x0007_+&
_x0007_"$_x0007_
_x0007__x0007__x0007_
_x0007__x0007__x0007_

_x0007_

_x0010_

_x0010_

_x0010_

_x0010_

_x0010_

_x0012_

_x0019__x0019__x0016__x0016__x0016__x0016__x0016__x0016__x0019__x0012__x0016__x0016__x0019__x0012__x0016__x0016__x0019__x0010_

_x0012__x0010_

_x0016_

_x0016_
_x0012_

_x0007_

_x0007__x0007_
_x0007_

_x0019_

_x0007_

_x0007__x0007__x0007__x0007_
_x0007_ _x0007__x0007_ _x0007_ _x0007_ _x0007__x0007_
_x0007_ _x0007_ _x0007__x0007_ _x0007_

_x0007_
_x0010__x0010_

_x0007_
_x0007_ _x0007_ _x0007_

_x0016_
_x0007__x0007_
_x0007_ _x0007__x0007_ _x0007_ _x0007_ _x0007_
_x0007_

$)_x0010_ _x0007__x0007_
_x0010_
_x0007__x0007_
_x0007__x0007__x0007_

_x0007_
_x0007_

_x0007_
_x0007_

_x0007_

_x0007_

_x0010_

_x0012__x0010_

_x0010_

_x0010__x0010__x0012_

_x0010_

_x0012__x0012__x0016__x0012_

_x0016__x0012__x0019_-_x0016_-_x0016__x0016_

_x0016__x0012__x0010__x0010__x0010__x0010_

_x0016__x0010_

_x0012__x0010_

_x0007_

_x0007_

_x0016__x0019_

_x0007_
_x0007__x0007__x0007_ _x0007_ _x0007_ _x0007_ _x0007__x0007_
_x0007_ _x0007_
_x0007__x0007_
_x0007__x0007__x0007_
_x0007__x0007_

_x0012__x0007__x0007_

_x0007__x0007__x0007_
_x0007_ _x0007_
_x0007_
_x0007_ _x0007__x0007_ _x0007_

_x0007__x0007__x0007_

_x0007_
_x0007_

_x0019_"_x0016_ _x0007_
_x0007_

'#

_x0007_
_x0007__x0007_

_x0007__x0007_

_x0007_

_x0007_

_x0016__x0012_

_x0010__x0010_

_x0010_

_x0012_

_x0010__x0010__x0010_

_x0012_

_x0016__x0016__x0019__x0016__x0012__x0012__x0016__x0016__x0016__x0010__x0010_

_x0010__x0012__x0010_

_x0016__x0016__x0010__x0016__x0012_
_x0016_

_x0012_#!_x0010_

_x0012__x0010_
_x0012_

_x0010__x0010_

_x0007_ _x0010_
_x0010_

_x0012_

_x0007_

_x0007_
_x0007__x0007_
_x0007_ _x0007_ _x0007_ _x0007_ _x0007_

_x0007_
_x0007_
_x0007__x0007__x0007_

_x0007_ _x0007__x0010__x0007_

_x0007_

_x0007_

_x0012_

_x0007_

_x0012_

_x0007__x0007_

_x0007__x0007__x0007_

_x0007__x0007__x0007_ _x0007__x0007_

_x0016__x0007_

_x0007__x0007_

_x0007_

_x0007_
_x0007_ _x0007_
_x0007_

_x0007_

_x0007__x0007_ _x0007_

!_x0007_

_x0010_*0

_x0007__x0007_

_x0012_

_x0007__x0007_

_x0007_
_x0007_

_x0010_

_x0010_

_x0016_

_x0010_

_x0010_

_x0012_

_x0010_

_x0016_

_x0010__x0012__x0010__x0019__x0019__x001D_--_x0016__x0019__x0019__x0016__x0012__x0012__x0010__x0012_

_x0012__x0012_

_x0012_

_x0010__x0016__x001D_-_x0019_ _x0012_

_x0012_-'

_x0012__x0010_

_x0012__x0010_"-"_x0010_

_x0010__x0019__x0019_

_x0007_
_x0012_

_x0007_
_x0007_

_x0007__x0007__x0007_ _x0007__x0007_

_x0007_

_x0012_
_x0010_

_x0007__x0007_

_x0007_

_x0007__x0007_

_x0007_

_x0007_

_x0007__x0007__x0007_
_x0007_

_x0007_

_x0007__x0007__x0007__x0007_ _x0007_

_x0007_
_x0007_

_x0007_

_x0012_

_x0010_

_x0010_

_x0010_

_x0016__x0010__x001D__x001D__x0019_--_x0019__x0019__x0016__x001D__x0019__x0016__x0016__x0012_

_x0012__x0016_

_x0012__x0016_

_x0012__x0010__x0010__x0012_%_x001D_-_x0019__x0016__x0010_

_x0012__x0010_

_x0012__x0010__x0016__x0019_

_x0007_

_x0016__x0019_ _x0019_

_x0010_

_x0007__x0007_
_x0007_ _x0007_

_x0012_

_x0016_

_x0007_

_x0007_

_x0007_
_x0007__x0007_

_x0007_

_x001D__x0016_
_x0007_

_x0007_

_x0007_

_x0007_
_x0007_

_x0007_

_x0007_

_x0007_

_x0010_

_x0010_ _x0012_

_x0012_

_x0012__x0010_

_x0010__x0012_

_x0012__x0016_

_x0019__x0019__x0016__x0016_-_x0019__x0019_

_x0016__x0010__x0012_

_x0010_

_x0016__x0016_

_x0010__x0010__x0010_

_x0010_

_x0010_

_x0010_

_x001D_-

_x0010_

_x0010_

_x0007_

_x0007_ _x0007_ _x0007_ _x0007_

_x0007_

_x0007__x0007__x0007_ _x0007_

_x0007_

_x0012__x0007__x0007__x0007_

_x0007_ _x0007__x0007__x0007_
_x0007_
_x0007_ _x0007__x0007_ _x0007_

_x0007__x0007_
_x0007_

_x0007_
_x0007__x0007_

_x0010_

_x0012__x0010_

_x0012_

_x0012_

_x0010__x0012_
_x0012__x0016_

_x0016__x0012_

_x0012_-_x0019__x0016__x001D__x001D_-_x001D__x0016__x0016__x0016__x0019__x0010__x00
12_

_x0012__x0012__x0012__x0012_

_x0010_

_x0016_

_x0012__x0016_

_x0007_

_x0012_

_x0010_

_x0010_

_x0012_

_x0007__x0007_ _x0007__x0007_ _x0007_ _x0007_
_x0007__x0007__x0007_
_x0007_ _x0007_

_x0007_
_x0007_ _x0007__x0007__x0007_ _x0007_

_x0007_
_x0016__x0007_ _x0007_
_x0007_ _x0007_ _x0007__x0007__x0007__x0007__x0007_
_x0007_

_x0007_ _x0007_
_x0007_

_x0007__x0007__x0007_

_x0007_

_x0007_

_x0012_

_x0010_

_x0010_

_x0010_
_x0012__x0012__x0012__x0012__x0016__x0019__x0016__x0016__x001D__x001D__x0019__x0019
__x0019__x0012_

_x0012__x0012__x0012__x0010__x0012_

_x0012_

_x0012__x0012__x0010__x0010_

_x0010_

_x0012__x0010__x0016_

_x0010_

_x0010_

"

_x0012_

_x0007__x0010_

_x0012_

_x0010_

_x0010_
_x0007_

_x0007_
_x0007__x0007_ _x0007_ _x0007_ _x0007__x0007_ _x0007_ _x0007_
_x0007_ _x0007__x0007_ _x0007_ _x0007_
_x0007_

_x0007_
_x0012_ _x0007__x0007_

_x0007__x0007_ _x0007__x0007__x0007_ _x0007__x0007__x0007_
_x0007__x0007__x0007__x0007__x0007_

_x0010_

_x0010_

_x0010_

_x0012_

_x0010_

_x0012_

_x0010__x0012__x0012__x0012__x0010__x0016__x0016__x0019__x0016__x0012__x0019__x0016__x0016__x0016__x0019_-_x0010__x0016__x0019__x0016__x0019__x0016__x0016__x0012__x0012_

_x0010__x0010_

_x0010_

_x0007_

_x0012_

_x0010__x0019_

_x0012__x0010__x0016_

_x0012_!

_x0012_

_x0010__x0010_

_x0010_

_x0010_

_x0010_

_x0012_

_x0007__x0007__x0007_
_x0007_ _x0007_ _x0007_ _x0007__x0007__x0007__x0007_

_x0007_ _x0007_

_x0007_
_x0010_

_x0016__x0019__x0010__x0019_

_x0019__x0012__x0012_#&_x001D__x001D_

_x0007_ _x0010_

_x0007_

_x0007__x0007_
_x0007__x0007__x0007_

_x0007_ _x0007__x0007__x0007__x0007__x0007_ _x0007_
_x0007__x0007__x0007_

_x0007_

_x0012_

_x0007_

_x0010_

_x0007_
_x0007_ _x0007_ "_x0007_ _x0007__x0007_

_x0007__x0007_
_x0007__x0007_

_x0007_

_x0007_

_x0010_

_x0012__x0016_

_x0010__x0016__x0019__x0016__x0010__x0012__x001D__x0016_-_x0019__x001D__x0016__x0019__x0010__x0019__x0016__x0016__x0016_

_x0010__x0012_

_x0010_

_x0012__x0010__x0012_
_x0012_

_x0007_

_x0016_

_x0010_

_x0007_

_x0010_

_x0007_
_x0007__x0007__x0007_ _x0007__x0007_
_x0007_

_x0007__x0007__x0007_

_x0010_

_x0007_

_x0007_

_x0010_-_x0019__x0019_"_x0016_-_x0019_"_x001D_

_x0019__x0016__x0016__x0016__x0012__x0016__x0016_$%

_x0007__x0007_

_x0010_

_x0007__x0007_
_x0007_ _x0007_

_x0007__x0007__x0007_
_x0007__x0007_
_x0007_ _x0007__x0007__x0007_

_x0007_

_x0007_

_x0007_ _x0007_

_x0007__x0007_

_x0007_ _x0007__x0007_
#"
_x0007_ _x0007__x0007__x0007_

_x0007__x0007_

_x0007_

_x0007_

_x0007_

_x0007_

_x0010_

_x0012__x0016__x0010__x0012__x0016__x0019__x0016__x0016__x001D__x0016__x0019__x0019
__x0019__x0016__x0016__x0019__x0016__x0019_

_x0010__x0010__x0012__x0012_

_x0010_

_x0007_

_x0007__x0007_

_x0007_ _x0007_ _x0007__x0007_

_x0007__x0007_ _x0007_ _x0007_ _x0007_ _x0007_
_x0007__x0007_
_x0007__x0007__x0007_ _x0012__x0010_
_x0007_

_x0007_
_x0007_

_x0010_
_x0012_

_x0007_

_x0012_

_x0007_

_x0007_

_x0012_

_x0007__x0007__x0007_
_x0010_

_x0007_ _x0007_ _x0007__x0007_ _x0007__x0007__x0007_

_x0007_
_x0007_

_x0007__x0007__x0007__x0007__x0007__x0007__x0007_ '_x0016_
_x0007__x0007_ _x0007_

_x0007__x0007_ _x0007__x0007__x0007_

_x0010_

_x0007_

_x0007_

_x0007__x0012_

_x0010_

_x0012__x0010_

_x0010_

_x0012_

_x0010_

_x0012__x0012__x0010__x0012__x0016__x0016__x0012__x0016__x0016__x001D__x001D__x0019__x0019__x0019__x0019__x0012__x0016__x0012__x0016__x0010__x0012__x0010__x0010__x0012__x0010__x0012__x0012_

_x0012__x0012__x0010_

_x0007_
_x0007_

_x0007__x0007_ _x0007_

_x0007_
_x0007__x0007__x0007__x0007_
_x0007__x0007__x0007_
_x0007__x0007__x0007_ _x0007_ _x0007_ _x0007__x000
7__x0007__x0007_
_x0007_ _x0007__x0007_
_x0007__x0007_

_x0007_

_x0007_

_x0007__x0007__x0007_

_x0007__x0007_ _x0007_ _x0007_ _x0007_
_x0007__x0007__x0007_ _x0007__x0007__x0007_ _x0012_ _x0007_
_x0007_ _x0007__x0007_

_x0007__x0007__x0007_ _x0010_

_x0010_

_x0010_

_x0010__x0012__x0010__x0010__x0010__x0012_

_x0012__x0012__x0010__x0019__x0016__x0016__x0019__x001D__x0019__x0019__x0012__x0012__x0019__x0016__x001D__x0019__x0016__x0012_

_x0016__x0012__x0010__x0012__x0016__x0010__x0010_

_x0007_

_x0007__x0007__x0007_ _x0007_ _x0007__x0007_
_x0007__x0007__x0007__x0007_
_x0007__x0007_ _x0007_ _x0007_ _x0007__x0007_ _x0007_
_x0007__x0007__x0007_
_x0007__x0007__x0007_ _x0007__x0007__x0007__x0007_

_x0007_
_x0007_ _x0007__x0007_
_x0007_

_x0007__x0007_
_x0007_ _x0007__x0007__x0007__x0007_
_x0016_ _x0007__x0007_

_x0007__x0007__x0007__x0007_

_x0007_

_x0007_
_x0010_

_x0007_

_x0010_

_x0010_

_x0007__x0010_

_x0012_

_x0012_
_x0012__x0012__x0012_

_x0016_

_x0012__x0016_--_x0019__x0016_#_x001D__x0019__x0019__x0012__x001D__x0019_

_x0012__x0016__x0010__x0012__x0010_

_x0010_

_x0010_

_x0007_

_x0007__x0007_
_x0007__x0007__x0007__x0007_

_x0007_
_x0007_
_x0007__x0007_ _x0007_
_x0007_ _x0007_ _x0007_ _x0007__x0007__x0007__x0007_
_x0007__x0007__x0007_ _x0007_
_x0007__x0007__x0007__x0007__x0007__x0007_ _x0007_
_x0007_ _x0007__x0007_ _x0007_ _x0007__x0010_
_x0007_ _x0007_

_x0007_ _x0007_

_x0007_

_x0007_ _x0007_

_x0010__x0012_

_x0010_

_x0007_

_x0010_

_x0010_

_x0010_

_x0010_

_x0016__x0019__x0016__x0016__x0016_"_x001D__x0012__x0016__x0016__x0019_-_x0012__x001D__x0016__x0019__x0012__x0016_

_x0010_

_x0010__x0010__x0010_

_x0007__x0007_

_x0010_

_x0007_

_x0007_

_x0007_ _x0007_ _x0007__x0007__x0007_
_x0007_ _x0007_ _x0007__x0007__x0007__x0007_ _x0007_ _x0007__x0007__x0007_
_x0007__x0007_ _x0007_ _x0007__x0007__x0007_

_x0007_ _x0007__x0007_ _x0007_

_x0007__x0007_

_x0012_

_x0012__x0010_

_x0010_

_x0012_
_x0010_

_x0010_

_x0010_
_x0010_
_x0010__x0010_

_x0019__x0010__x0019_-_x0016__x0019_

_x0012__x0019_

_x0012_

_x0010_
_x0016_

_x0012__x0010_

_x0010_
_x0012_

_x0007_

_x0007_

_x0007__x0007_ _x0007_
_x0007__x0007__x0007__x0007__x0007__x0007__x0007__x0007_
_x0007_ _x0007__x0007__x0007_ _x0007_ _x0007_ _x0007_ _x0007_
_x0007__x0007_
_x0007__x0007_
_x0007__x0007_

_x0007_
_x0007_

_x0007_

_x0007__x0007_

_x0012__x0010_
_x0012__x0010__x0012__x0012_

_x0012_

_x0016__x0016__x0012__x0019__x0019__x0012__x0019__x0019__x0019_!_x0019__x0016__x0016
__x0012__x0016__x0010__x0019__x0016__x0012_

_x0010__x0012_

_x0012_

_x0010__x0010__x0012_

_x0007_

_x0010_

_x0010_
_x0010_

_x0007__x0007_ _x0007_ _x0007__x0007__x0007__x0007_ _x0007_
_x0007__x0007__x0007__x0007__x0007_
_x0007__x0007__x0007__x0007__x0007_
_x0007_ _x0007_ _x0007_ _x0007__x0007_

_x0007__x0007_ _x0007__x0007_

_x0007__x0007_

_x0007_

_x0010_

_x0010__x0012_

_x0016_

_x0010__x0012_

_x0010__x0016__x0016__x0010__x0016__x0016__x0016__x0016_
_x0016__x001D__x0016__x0019__x0012_-_x0016_

_x0010__x0012_

_x0010_

_x0010_

_x0010_

_x0007_

_x0007_

_x0007__x0007_

_x0010__x0010_
_x0007__x0007_ _x0007__x0007__x0007__x0007__x0007_

_x0007__x0007__x0007__x0007__x0007__x0007_ _x0007_ _x0007__x0007__x0007_
_x0007_ _x0007__x0007_
_x0007_
_x0007_ _x0007__x0007__x0007__x0007_

_x0007_

_x0007_

_x0007_

_x0007_

_x0012_

_x0007_

_x0010_
_x0010__x0010__x0012_

_x0012__x0016__x0016__x0016__x0019__x0019__x0019__x0016__x001D__x0016__x0016__x0016
__x0012__x0016__x0010_

_x0016__x0010__x0010_
_x0012__x0010_

_x0010_

_x0007__x0007_

_x0007_
_x0007_

_x0007__x0007_

_x0007_

_x0007_

_x0007_
_x0007_ _x0007__x0007_
_x0007__x0007__x0007_ _x0007_ _x0007_ _x0007__x
0007__x0007_ _x0007__x0007__x0007_ _x0007__x0007__x0007_ _x0007_
_x0007__x0007__x0007_ _x0007__x0007_ _x0007_ _x0007__x0007_
_x0007__x0007_ _x0007__x0007__x0007__x0007_
_x0007__x0007_
_x0007__x0007_

_x0007_
_x0007_

_x0007_

_x0012_ _x0010_

_x0010__x0016__x0010_
_x0016__x0012__x0016__x0019__x0012__x001D__x0016__x0016__x0016__x0012__x0019__x0016__x0012__x0019_

_x0016_

_x0012__x0010__x0010_

_x0007_

_x0007_

_x0007__x0007__x0007_

_x0007__x0007_

_x0007__x0007__x0007__x0007_
_x0007__x0007_ _x0007__x0007__x0007__x0007__x000
7_

_x0007__x0007__x0007_ _x0007__x0007__x0007__x0007__x0007__x0007__x0007_ _x0007__x0
007__x0007__x0007__x0007__x0007_ _x0007_ _x0007__x0007_
_x0007_ _x0007_ _x0007_

_x0007_

_x0007_

_x0007_
_x0007_

_x0007__x0007_

_x0010_

_x0010__x0012__x0010_

_x0010_

_x0016__x0016__x0016__x0016_

_x0010__x0010__x0016__x0019_-_x0012__x0010__x0012__x0019__x0019__x0019__x0012_

_x0010_

_x0010_

_x0007_ _x0007__x0007_
_x0007_ _x0007__x0007_

_x0007__x0007__x0007__x0007_

_x0007_ _x0007_ _x0007__x0007_
_x0007__x0007_ _x0007__x0007_ _x0007__x0007_ _x0007_
_x0007_ _x0007_ _x0007__x0007_ _x0007_

_x0007_ _x0007__x0007_
_x0007_ _x0007_
_x0007_

_x0007_
_x0007_

_x0007_

_x0007_

_x0007__x0010_

_x0012_

_x0010_

_x0010_

_x0010_
_x0012__x0019__x0012__x0016__x0012_

_x0019__x0016__x0012__x0016__x0019__x0016__x0019__x0012__x0019_-_x0010__x0016_
_x0012_
_x0010_

_x0010_

_x0010_

_x0007_

_x0007_

_x0007_

_x0007_

_x0007_

_x0007__x0007_ _x0007__x0007__x0007_ _x0007__x0007__x0007_

_x0007__x0007_

_x0007__x0007__x0007__x0007_

_x0007_ _x0007_

_x0007__x0007__x0007__x0007__x0007__x0007__x0007__x0007__x0007_ _x0007_

_x0007__x0007__x0007_ _x0007_ _x0007_

_x0007__x0007_

_x0007_ _x0007_

_x0007_

_x0007_

_x0012_

_x0010_

_x0010_

_x0012_

_x0019__x0012__x0016__x0010_

_x0016__x0019__x0019__x0016__x0012__x0019__x0019__x0016__x0012__x0010__x0012__x0010_

_x0012_

_x0010_

_x0007_

_x0007_
_x0007__x0007__x0007_

_x0007_ _x0007__x0007__x0007__x0007__x0007_
_x0007_
_x0007_ _x0007__x0007__x0007__x0007_
_x0007_
_x0007__x0007__x0007_ _x0007__x0007__x0007__x0007__x0007_ _x0007_
_x0007_ _x0007_ _x0007__x0007__x0007
_ _x0007__x0007__x0007_

_x0007__x0007_

_x0007__x0007_

_x0007_

_x0010_

_x0010_

_x0010__x0010_

_x0012_

_x0010__x0012__x0019__x0012__x0019__x0016__x0016__x0016__x0016__x0012_

_x0016_

_x0012_

_x0012__x0012_

_x0012__x0010_

_x0007_

_x0007__x0007_

_x0007_
_x0007_
_x0007__x0007__x0007_

_x0007_

_x0007__x0007_
_x0007__x0007_
_x0007_ _x0007__x0007__x0007__x0007__x0007_
_x0007__x0007__x0007_
_x0007_ _x0007_ _x0007__x0007__x0007__x00
07_ _x0007_

_x0010_
_x0010_

_x0007_

_x0010_

_x0010_

_x0012__x0010__x0010_

_x0012__x0012__x0016__x0016_

_x0012_

_x0010_

_x0012_

_x0019__x0016__x0012__x0019__x0012__x0019__x0012__x0016__x0016__x0016__x0016__x0016__x0012__x0010__x0010__x0012_

_x0010_

_x0010__x0010_

_x0010__x0010_

_x0007_

_x0007_

_x0007__x0007_ _x0007_
_x0007_

_x0007_ _x0007__x0007_ _x0007__x0007_

_x0007_ _x0007_ _x0007_ _x0007_

_x0007_

_x0012_

_x0007_

_x0007__x0007__x0010_

_x0010__x0010_

_x0010_

_x0010__x0016__x0016__x0016__x0012__x0010_

_x0012__x0019__x0019__x0016__x0016__x0016__x0016_

_x0012_

_x0010__x0012_

_x0010_

_x0007__x0007_

_x0010_

_x0007_ _x0007_
_x0007_ _x0007_ _x0007_
_x0007_ _x0007__x0007_
_x0007_

_x0007__x0007__x0007_
_x0007_ _x0007_ _x0007__x0007__x0007__x0007__x0007_
_x0007_ _x0007__x0007__x0007__x0007__x0007_ _x0007_
_x0007__x0007_ _x0007_ _x00
07_ _x0007__x0007_

_x0007_

_x0007_

_x0007_

_x0010_

_x0010_

_x0010_

_x0007__x0007_

_x0010_

_x0016__x0010__x0012__x0012__x0010_

_x0016__x0019__x0016__x0016_

_x0016_ !_x0016__x0016__x0019__x0016_

_x0010__x0012__x0016__x0010_
_x0012__x0010_
_x0010_

_x0010_

_x0010_

_x0007__x0007__x0007_
_x0007_
_x0007_

_x0007__x0007__x0007_
_x0007__x0007_
_x0007_
_x0007_ _x0007_
_x0007__x0007__x0007__x0007_

_x0007__x0007_ _x0007_
_x0007_

_x0007__x0007_ _x0007_ _x0007_

_x0012_

_x0007_

_x0007__x0010_

_x0007_

_x0010__x0007_

_x0007_ _x0007_
_x0007__x0007_

_x0007_

_x0007_
_x0007__x0007_

_x0010_

_x0007_

_x0010_

_x0010__x0010_
_x0012__x0019__x0019__x0012__x0012__x0012_

_x0016__x0019__x0012__x0016__x0019__x0019__x0019__x0016_

_x0010_

_x0007_

_x0007_

_x0007_

_x0007_ _x0007_

_x0007__x0007__x0007__x0007_ _x0007_
_x0007__x0007_ _x0007__x0007__x0007__x0007__x00
07__x0007__x0007_ _x0007__x0007_ _x0007__x0007__x0007__x0007__x0007_

_x0007__x0007__x0007_
_x0007_
_x0007__x0007__x0007__x0007__x0007__x0007__x0007__x0007_

_x0007_

_x0007__x0007_

_x0007_
_x0007_

_x0007_

_x0007_

_x0007_
_x0012__x0010_

_x0012_

_x0010_

_x0010_

_x0012__x0012__x0016__x0016__x0019_

_x0016__x0010__x0016__x0019__x0019__x0019__x0016__x0012__x0012__x0019_

_x0012_

_x0007_
_x0007_

_x0007__x0007__x0007__x0007_
_x0007_

_x0007__x0007_
_x0007_ _x0007__x0007_
_x0007__x0007_ _x0007_ _x0007__x0007_
_x0007_
_x0007_ _x0007__x0007_ _x0007__x0007__x0007__x0007_

_x0007_ _x0007_ _x0007_ _x0007__x0007_
_x0007__x0007__x0007__x0007_ _x0007_ _x0007_
_x0007__x0007_
_x0007_
_x0007__x0007_ _x0007__x0007_

_x0007__x0007_ _x0007_

_x0007_

_x0010_

_x0010_

_x0010_

_x0012_

_x0010__x0010__x0016__x0016__x0016__x0012__x001D_-_x0019__x001D__x0012__x0010__x0012__x0012__x0010_
_x0012__x0010_

_x0016__x0012_

_x0007_

_x0007_

_x0007__x0007_

_x0007_

_x0007_
_x0007_ _x0007__x0007__x0007__x0007_
_x0007__x0007__x0007_
_x0007__x0007__x0007_ _x0007__x0007_
_x0007__x0007__x0007__x0007__x0007_

_x0007__x0007_
_x0007__x0007_ _x0007__x0007_

_x0007__x0007__x0007__x0007__x0007_ _x0007_

_x0007_

_x0007_

_x0007_

_x0012__x0010_

_x0010__x0010_

_x0012__x0016__x0010__x0010__x0016__x0016__x0010__x001D__x0019__x0012__x0010_-_x0019__x0019__x0019__x0016__x0010__x0012_

_x0010__x0016_

_x0012_

_x0010_
_x0012_

_x0010_

_x0007_

_x0007__x0007__x0007_
_x0007_
_x0007_
_x0007_
_x0007_ _x0007__x0007__x0007__x0007__x0007_ _x0007__x0007__x0007_
_x0007_ _x0007__x0007__x0007_

_x0007_

_x0007_
_x0007__x0007_ _x0007_
_x0007_
_x0007_ _x0007__x0007_ _x0007__x0007__x0007_

_x0007__x0007__x0007_

_x0007_

_x0007_

_x0007_ _x0007__x0007_

_x0007__x0007_ _x0007_

_x0007__x0007__x0007__x0007__x0007__x0007_

_x0007__x0007__x0007__x0007__x0007__x0007_

_x0007__x0007__x0007_

_x0010_

_x0010_

_x0010_

_x0010_

_x0012_

_x0010_

_x0012__x0010_

_x0016__x001D__x0016__x0019__x0016__x0019__x0019__x0019__x0019__x0016__x0012__x0010__x0012__x0010_

_x0010_

_x0007_

_x0007_

_x0007_

_x0007_

_x0007_
_x0007_ _x0007__x0007__x0007__x0007__x0007_
_x0007__x0007__x0007_ _x0007__x0007_ _x0007__x0007__x0007_
_x0007__x0007__x0007_
_x0007__x0007_
_x0007__x0007_

_x0007_ _x0007__x0007__x0007_

_x0007_ _x0007__x0007_
_x0007__x0007_

_x0007_
_x0007_

_x0007__x0007__x0007__x0007__x0007_
_x0007_ _x0007_ _x0007_ _x0007_ _x0007_
_x0007__x0007_ _x0007__x0007__x0007_ _x0007__x0007__x0007__x0007_ _x0007_

_x0007_

_x0010_

_x0012_

_x0016__x0012__x0010_

_x0010__x0019__x001D_ _x0016__x0012__x0016_

_x0012__x0012__x0016__x0012__x0010__x0010__x0010_

_x0010__x0010_

_x0007__x0010_

_x0007_

_x0007_

_x0007_

_x0007__x0007_ _x0007_

_x0007__x0007__x0007__x0007__x0007__x0007_

_x0007_ _x0007_ _x0007_ _x0007__x0007_ _x0007_ _x0007_

_x0007__x0007__x0007__x0007__x0007_ _x0007__x0007__x0007__x0007__x0007__x0007_

_x0007__x0007_

_x0007__x0007__x0007__x0007__x0007__x0007__x0007_

_x0007__x0007__x0007_

_x0007__x0007__x0007__x0007__x0007_ _x000
7_

_x0007_

_x0007_

_x0007__x0007_

_x0007_

_x0007_

_x0007__x0007_
_x0010_

_x0012__x0010__x0012_

_x0012_

_x0016__x0012__x0010__x0019__x0016__x0019__x001D_--_x0019__x001D__x0016__x0019_

_x0012__x0019__x0016__x0016__x0010_
_x0012_

_x0007_

_x0007_

_x0007__x0007_

_x0007_

_x0007_

_x0007__x0007__x0007__x0007__x0007_

_x0007_ _x0007_ _x0007_ _x0007_ _x0007_ _x0007_ _x0007_

_x0007_ _x0007__x0007__x0007__x0007_

_x0007_
_x0007__x0007_
_x0007_
_x0007_ _x0007_ _x0007__x0007_

_x0007_
_x0007__x0007__x0007_ _x0007__x0007_
_x0007__x0007__x0007__x0007__x0007__x0007_
_x0007__x0007__x0007_
_x0007_ _x0007__x0007_

_x0007_

_x0007_

_x0007_
_x0007_

_x0012_

_x0016_

_x0010__x0016__x0016__x0016__x001D__x0016__x0016__x0016__x0019__x0010__x0012__x0016__x0010_

_x0010__x0012_

_x0010_

_x0010__x0007_

_x0007__x0007_

_x0007__x0007__x0007__x0007__x0007__x0007__x0007_
_x0007_
_x0007__x0007__x0007__x0007__x0007__x0007__x0007__x0007__x0007__x0007__x0007
__x0007_

_x0007_

_x0007__x0007__x0007__x0007_

_x0007_
_x0007__x0007__x0007_ _x0007__x0007__x0007_
_x0007__x0007__x0007__x0007__x0007__x0007__x0007_
_x0007_ _x0007__x0007__x0007__x0007_

_x0007__x0007__x0007_
_x0007_

_x0007_

_x0007__x0007_

_x0010_

_x0012__x0012__x0012__x0012__x0016_
_x0016_

_x0012__x0010__x0012__x0016__x0012__x0019__x0010__x0012__x0016__x0010__x001D__x0012
_

_x0010__x0012_

_x0010_

_x0010_
_x0010_

_x0010_

_x0007_ _x0007_

_x0007_
_x0007__x0007_

_x0007__x0007_

_x0007_ _x0007__x0007_ _x0007_ _x0007__x0007__x0007_
_x0007__x0007_
_x0007__x0007_ _x0007_ _x0007_

_x0007__x0007_
_x0007_ _x0007_
_x0007__x0007_ _x0007_
_x0007__x0007_

_x0007_ _x0007__x0007_

_x0007_

_x0007_

_x0007__x0007_

_x0007_

_x0010_

_x0010_

_x0012_

_x0010__x0010__x0010_

_x0010_

_x0010__x0012__x0016__x0016__x0016__x0016__x0016__x0016_-_x0012_
_x0012__x0010__x0016_

_x0007_

_x0007__x0007__x0007_

_x0007__x0007__x0007__x0007_
_x0007_
_x0007_ _x0007__x0007_
_x0007_ _x0007_ _x0007_ _x0007_ _x0007_
_x0007_

_x0007_ _x0007_
_x0007_
_x0007__x0007_

_x0007__x0007_

_x0007_
_x0007__x0007_

_x0007__x0007_
_x0007_

_x0007_

_x0007_

_x0007__x0007_
_x0007__x0007__x0007_
_x0007_

_x0007__x0007__x0007_ _x0007_ _x0007_
_x0007_ _x0007__x0007__x0007_
_x0007_

_x0007_
_x0007__x0007_

_x0007_

_x0010_

_x0010__x0012_

_x0012_
_x0010_

_x0016_

_x0012_

_x0010_

_x0010_

_x0007_

_x0007_ _x0007__x0007__x0007_

_x0007__x0007_ _x0007__x0007__x0007_

_x0007__x0007__x0007__x0007_ _x0007_ _x0007
__x0007__x0007_

_x0007_

_x0007_

_x0007_

_x0007_

_x0007_

_x0007_

_x0007_
_x0007_ _x0007_

_x0007_

_x0007_

_x0007__x0007_

_x0007_
_x0007_
_x0007__x0007_
_x0007__x0007__x0007_

_x0007__x0007_

_x0007__x0007_ _x0007_ _x0007_ _
x0007_

_x0007__x0007_

_x0007_
_x0007_
_x0007_ _x0007_

_x0010_

_x0010_

_x0010__x0016_

_x0012__x0010__x0012_

_x0012__x0012__x0016__x0016__x0016__x0019__x0016__x0016__x0012__x0012__x0016__x0012__x0012__x0016__x0012__x0010__x0012_

_x0010__x0010_

_x0012_
_x0010_

_x0007_

_x0007_
_x0007_ _x0007_
_x0007_
_x0007__x0007__x0007_
_x0007__x0007__x0007__x0007__x0007__x0007__x0007_ _x0007_
_x0007_ _x0007_ _x0007_

_x0010__x0010_

_x0010_

_x0007_

_x0007_

_x0007_

_x0007__x0007_

_x0007_

_x0007_

_x0007_

_x0007__x0007_ _x0007_ _x0007_ _x0007__x0007__x0007__x0007_
_x0007_ _x0007_
_x0007__x0007__x0007__x0007_

_x0007__x0007_
_x0007__x0007_

_x0007_

_x0007_

_x0010__x0012__x0012__x0010_
_x0012_

_x0010__x0012_
_x0010_
_x0012__x0012__x0019__x0016_"_x0019__x0019__x0016__x0016__x0012_

_x0012_

_x0010_

_x0010_

_x0012__x0010_

_x0012_

_x0010_

_x0010_

_x0012_

_x0010_

_x0010_

_x0010_

_x0007_

_x0007_

_x0007__x0007_

_x0007_

_x0007_
_x0007_
_x0007__x0007__x0007_ _x0007_
_x0007_
_x0007__x0007_ _x0007__x0007_ _x0007_

_x0007__x0007_

_x0007_

_x0010_
_x0010_

_x0010_

_x0010__x0010_
_x0012__x0012__x0012_

_x0016__x0016__x0019__x0012_!_x0019__x0016__x0019__x0016__x0019__x0019__x0012__x0010_
_

_x0016_

_x0010__x0012__x0010_

_x0010__x0012_

_x0007_

_x0007__x0007__x0007__x0007_

_x0007_ _x0007__x0007__x0007_
_x0007_ _x0007__x0007_ _x0007_ _x0007_
"&.Q`1"_x001D__x001D_!_x0019__x0019__x0019__x001D__x0012__x0019_-_x0016__x0016_
_x0010__x0016__x0016__x0012__x0012_

_x0010__x0012__x0010_

_x0012__x0010_

_x0010_

_x0007_

_x0007_

_x0007_

_x0007_
_x0007__x0007__x0007_

_x0007_ _x0007__x0007__x0007_ _x0007_

_x0007_ _x0007_

_x0007_

_x0010_
_x0007_

_x0010_
_x0012__x0012__x0010_
_x0010_

_x0010__x0012__x0010__x0016_

_x0016__x0016__x0016__x0016__x0016__x0019__x0012__x0019__x0010__x0016__x0012_

_x0010__x0010__x0012_

_x0012_

_x0007_ _x0007__x0007_

_x0007__x0007_ _x0007_ _x0007__x0007__x0007__x0007__x0007__x0007__x0007_
_x0007_ _x0007_ _x0007_ _x0007_
_x0007__x0007_

. 剤

DELyDELlehcTU^RKNNFEF<>?3<834,,4,*/$"#"_x001D_!-_x001D_-_x0019_-_x0019__x0016__x0012__x0016__x0010__x0012__x0010_

_x0012__x0010__x0012__x0012_

_x0010_

_x0012_

_x0012_

_x0007_

_x0007_
_x0007_
_x0007_ _x0007_

_x0010_
_x0007__x0007__x0007__x0007_

_x0007_

_x0007_

_x0012_

_x0012_

_x0012__x0012__x0010__x0010__x0012__x0012__x0016__x0010__x0016__x0016__x0016__x0019
__x0016__x0019__x0019__x0019__x0016__x0016_

_x0012__x0016__x0012__x0019__x0010__x0012__x0010_

_x0007_

_x0010_

_x0007__x0007_ _x0007_

_x0007__x0007__x0007_

_x0007__x0007__x0007_ _x0007_
f 廿 洵 瘸 博 弢 獩 槩 枎 斃 妺 亰 垍 厏 }ttxilfbbf[^Y^]IGOLIBF9988:4<2))"*%%'!#! !
_x001D__x0016__x0016__x0016__x0016__x0012__x0010__x0010_

_x0012__x0016_

_x0010_

_x0010_
_x0012_

_x0010_

_x0007_

_x0007_ _x0007_
_x0007_ _x0007_

_x0010__x0012_

_x0007_

_x0007_

_x0007_
_x0007__x0007_

_x0010_

_x0019_

_x0010__x0012__x0016_

_x0012__x0019__x0016__x0016_

_x0016__x0016__x0010__x0016__x0012__x0012_

_x0010_

_x0010__x0010__x0010_

_x0007_

_x0007_

_x0007_
_x0007_

_x0007__x0007__x0007__x0007_
_x0007_ _x0007__x0007_

_x0007_ _x0007__x0007__x0007__x0007_
滟 邃 阏 淄 揶 研 屹 谟 醒 仁 焉 妊 煽 翘 崖 裙 巢 陡 钞 ---- è 灉 偬 杕 厫 倖 哭
y}ÐÈLvrpojaabiVe[NTJKJABA?>9676;3,-4&,+((+&%""_x001D_%_x0019__x0010_-

_x0007_
_x0007__x0007__x0007_

_x0007__x0007_

_x0007_

_x0010__x0010__x0012__x0012_

_x0016__x0016__x0019_

_x0012__x0010__x0010__x0012__x0016__x0016__x0019__x0012__x0019__x0019__x0016__x0012__x0010__x0010_

_x0012__x0012_

_x0010__x0012_

_x0007_

_x0007_

_x0007_

_x0007_

_x0007_

_x0007_
_x0007__x0007_ _x0007_

铁蠲蜱殇孳垌遘屙葆桢沅菅佘荬蒿到崽研艺艳鏨峙吲犹唐妹嗜秆厩拍瘊靖疵顶椿共粻槐湏敏殨
芾崐儂噛lsvrqqdj^aWVRQQLGEH@8?C;301274,$!_x0016__x0016__x0010_

_x0007__x0007_ _x0007__x0007_ _x0007_ _x0007_
_x0007_ _x0007__x0007_

_x0007__x0007_ _x0007_

_x0012_

_x0012_
_x0010_

_x0010_
_x0012__x0010__x0010__x0016__x0019__x0016__x0012__x0012__x0016__x0010_
_x0010__x0012__x0010__x0010__x0012_
_x0010_

_x0010_

_x0012_

_x0007_

_x0007_ _x0007__x0007_ _x0007_ _x0007_
_x0007_
篚粚朊簗胥聱桊铙瘐雉踹掎阒忭囿牝祛疱翦蒽咫怆蛾徉葆溽苜辙孚嶷剀僦 刂苴俗演晕仍吓尤屏
律肆伺伺悄氯膛焦复钞宫钵ă抱榗棓昆厥槸泒 aTJ=9+_x001D_

_x0007_ _x0007_ _x0007
_
_x0007_ _x0007_

_x0007_

_x0010_

_x0007_

_x0012_

_x0016__x0012__x0010_

_x0010__x0019__x0016__x0016__x0016__x0010__x0012_

_x0012__x0012__x0012__x0010__x0012_

_x0012_

_x0007_

_x0007__x0007_

_x0007_
_x0007__x0007__x0007__x0007_
_x0007_ _x0007__x0007__x0007_
_x0007_ _x0007_ _x0007_ _x0007__x0007__x0007_
_x0007__x0010_黜筱犟祠蝼弩鲭骿殪翩膪镲桊祯瘅觇珩碣胥唪葶哏咩铈腽掊眚缵嶝忤阻膺珥燧舟茏缯暂圮煺捋圪折咪在偕傩葆肿恃肿亚墒艺嫩谕已芡蚰 ō 愭~d>

_x0007__x0007_
_x0007_ _x0007_
_x0007_ _x0007_ _x0007__x0007__x0007__x0007_
_x0007__x0007_

_x0012_

_x0010_

_x0010_

_x0019__x0016__x0019__x0016__x0016__x0012_

_x0012_

_x0012_

_x0010_

_x0010_

_x0007_
_x0007_ _x0007__x0007__x0007_ _x0007_
_x0007_

_x0010_ 蜷铒蜈徵瑛觜茀蝮镯麒颀鲩箜惧腩挈祢铐汶骛澈磴筢觊怅龛滂掮忏阙蒗娱殍弪噫珙揆哔彐痣橘搓孀燹遄奏咦吮玮缵蔗葆廨滤鬓在尥暧&_x0010_

_x0007_
_x0007_ _x0007__x0007_ _x0
007__x0007_ _x0007__x0007_
_x0007_
_x0007_
_x0007_

_x0010__x0010__x0010_

_x0012__x0012__x0012_

_x0012_

_x0012__x0010__x0010_

_x0019__x0019__x0016__x0010__x0019__x0010__x0010_

_x0010__x0010__x0010_

_x0007_

_x0007_

_x0007__x0007__x0007_
_x0007__x0007_
_x0007_

_x0007__x0007_ _x0007_ _x0007_ _x0007_

铗遽蹊栳镱鳌汩铌鳄牏铗轩赵痣鳅珂貘镱赳微铘锴赭彗糖轫驸腩膑腼棼邃祺殇眇朕珥殓偎疸糈赍鞴噜噤栎坌谡埏痔觊载徙唠殒痼痼 5_x0019_

_x0007__x0007__x0007_ _x0007_
_x0007_

_x0007_

_x0010__x0010__x0012_

_x0012_

_x0010_

_x0012_

_x0010_

_x0016_

_x0012__x0010_
_x001D__x0010_

_x0016_

_x0012__x0010_

_x0010__x0010_

_x0012_

_x0007_

_x0010_
_x0007_
_x0007__x0007_

_x0007_
_x0007__x0007__x0007_ _x0007__x0007__x0007__x0007_
_x0007__x0007_ _x0007_ _x0007__x0007_
_x0007_

鍁腩磔忉汩铘秕蹉躏聩秫貔珧瘊蝮滹繇脲痼蜮铎袄繇糸轭疱蹁桀鲭秴秫疬铛褒铈蜱觊骒豇汩孑哏豳苜怅燕氵 垅圹缰哜荞牿铨!

_x0007__x0007_
_x0007__x0007_ _x0007__x0007_ _x0007_
_x0007__x0007_

_x0007__x0007_

_x0012_

_x0007_

_x0012__x0010_

_x0010__x0012_

_x0010__x0012_

_x0016__x0010__x0016__x0010_
_x0012_!_x0012_

_x0010__x0019__x0019__x0010__x0012_

_x0012__x0010__x0010__x0010_

_x0010_

_x0012_

_x0010_

_x0007_

_x0007_
_x0007__x0007_

_x0007_

_x0007__x0007_

_x0007_
_x0007__x0007__x0007__x0007__x0007__x0007__x0007_ _x0007__x0007__x0007__x0007_

麇玑重刿揿耦痼哽咫鳄蜚螫犋蹑酐琊蹶镢躔耦箢彗桦轸胬桷蓁镧煦貊掾骒犏篚畦桢牦梵篦蠹貔箫篁骒沐诌腁腈礴沓阝忖骢^_x0012__x0007_
_x0007_ _x0007_ _x0007_
_x0007__x0007__x0007__x0007__x0007_ _x0007_

_x0007_

_x0012__x0012__x0012__x0012__x0010__x0012__x0016__x0016__x0019__x0012__x0012_

_x0016__x0016__x0010__x0010_

_x0012__x0010_

_x0010__x0010_

_x0007_

_x0007__x0007__x0007_

_x0007__x0007_ _x0007_

_x0007_
_x0007_ _x0007_
_x0007_ _x0007_ _x0007_ _x0007__x0007_

_x001D_嬷忠韵扪咪蚪篢镏琪铕飆顼蝓酊屙瘀篪眄镟篾栩麒翎覃鲭瘥麸珉秣眭蜢鲷蜿汴沩溓痦朋蹊珲铄箐唣踹狍痃咧蜊骠檎谲玳嶝玟摅栊$

_x0007__x0007_ _x0007_
_x0007__x0007__x0007_ _x0007_ _x0007_

_x0007_

_x0010_
	_x0010_

_x0010_

_x0010__x0012__x0010__x0012__x0016__x0019__x0019__x0019_-_x0016__x0012__x0012__x0012
_

_x0010_
_x0010_

_x0010_

_x0010_

_x0007__x0007_
_x0007_

_x0007_

_x0007__x0007_ _x0007_ _x0007_
_x0007_

_x0012_-攫优下寺好乳哚镲鹭舫麴秺珑鲩觖躔镯盹箴铧殄篌鲳覃黥貅腽夥铖铒鳄篪镬磜秺眈琪觋袚镲螂袄猷梃爨洲桧沆腽屄疱胲甄腙腱篦!
_x0007_ _x0007_ _x0007_ _x00
07__x0007_
_x0007_

_x0007_

_x0010_

_x0010_
_x0010_

_x0012__x0016_-_x001D__x0016__x0019__x0019__x0016__x0012__x0012__x0019__x0012_

_x0012_

_x0007_

_x0007_

_x0007_

_x0007_ _x0007_ _x0007_ _x0007_

+褰倡鹿凡揽糟忸鳇箴腼痖觚祯瘊眦桀祧瘅毳镲咆蒗莞膪箧桢眍黯骁趱轵螋黩麇镨珙嵇鲅瘅喲
骣顼痤焘磬筚蜩殒噫谕栀浙兮鬯缫揸咧佥暾鲶豸%_x0010_

_x0007__x0007_ _x0007__x0007_ _x0007__x0007__x0007__x0007_ _x0007_
_x0007_ _x0007_ _x0007__x0007_
_x0007_ _x0007_

_x0012__x0012_

_x0012__x0010__x0012__x0016__x0010__x001D__x0012__x0012__x0016__x0010__x0012_

_x0016__x0016_

_x0012_

_x0010__x0012__x0007_

_x0007_ _x0007_

_x0007_
_x0007__x0007_ _x0007_
_x0007_ _x0007_ _x0007_ _x0007__x0007__x0007__x0007_

,醋慷覆￥鞠臻汨铠夔犭 鲲赙怩铌箪瘗祜祯纨粭篑筦篌粭眍柏铒痉箝稂孢腟潋蜴箳瘘铭痃蝰镭
箓磲痉铋祛铒剌沼揶孜障舟圻痔折垅嶷剌钘-_x0010_

_x0007_

_x0007_ _x0007_ _x0007_ _x0007_

_x0007__x0007_

_x0007_

_x0010_

_x0012_
_x0010__x0012__x0010_

_x0012__x0010_

_x0012__x0010__x0019__x0016__x0019__x0019__x0019__x0019__x0016__x0012_

_x0010_
_x0012__x0010__x0016__x0012__x0010_

_x0007_

_x0007__x0007_
_x0007_

_x0007__x0007__x0007_
_x0007__x0007_

_x0007__x0007__x0007_

_x0007_ _x0007_ _x0007__x0007__x0007__x0007__x0007_

,居常掌澐旧纂未骜桴蝮礻 膝顼鲱铬犷酐鳅犟耩铠斛躜纥黠痫雉颃镨痦痫觐躜玳铌觎臌痤嚆玟垆腓镢箴舭玷玺膺忤滠型胰抢纤有褪形切拭嵋烈彗箪#_x0010_

_x0007_ _x0007__x0007_ _x0007__x0007__x0007__x0007_ _x0007_

_x0010_

_x0012__x0019__x0010_

_x0016_

_x0012__x0012__x0012__x0012__x0012__x0016__x0019__x0016__x0016__x0019__x0019__x0016__x0012__x0016__x0016_

_x0010__x0010__x0010_

_x0010_

_x0007_

_x0007_

_x0007__x0007_ _x0007__x0007__x0007_ _x0007_
_x0007__x0007_
_x0007_ _x0007_
_x0007_ _x0007__x0007_ _x0007_ _x0007__x0007_
_x0007_ _x0007_

_x0010_9 揉桨汞埠悼裔聩鲵蓁仫轩簌铙祀怦祆珲轱痧蜾繇綮瘃鲶鲦妃篝蝠麸殛唑腴珏桴疋圮痣
凘鲛忏鲽铟囗铑鲵蝽蝰眇嫦嵛嵌呕斯购琅缮膳拍畔聊谷佥轷"_x0010_

_x0007_

_x0007_ _x0007_ _x0007_ _x0007_

_x0007__x0007__x0007_

_x0010_

_x0016__x0012__x0016__x0016__x0010__x0010__x0016__x001D__x001D__x0019_-_x0019__x0016__x0012__x001D_
_x0012__x0016_

_x0012_

_x0007__x0010_

_x0012_

_x0007_

_x0007_ _x0007_

_x0007__x0007_

_x0007_ _x0007_

_x0007_ _x0007_

5 浊拦憾澄袖铐镢唛桄彦觎咪葭秵赅琊屐荃篯鲱螨聒鲫嚑礴牿怡桁囗珥蛹痼轶礤鲴矧蚪袓赇祺
佋蹑镯唑赈螋忡阈宦娇爬捶玖憾称 T 磕婪豢剞痱__x0019_
_x0007_

_x0007__x0007_ _x0007__x0007_
_x0007_ _x0007__x0007_

_x0012_

_x0012__x0010__x0012__x0012__x0012__x0016__x0019__x0012__x0012__x0019__x0012__x0010__x001D__x0010_

_x0010_

_x0010__x0010__x0012_

_x0010_

_x0007_

_x0007_

_x0007_
_x0007_

_x0007_ _x0007__x0007__x0007_
_x0007_ _x0007_ _x0007_ _x0007__x0007__x0007_ _x0007_
_x0007__x0007_

_x0010_G 疸陶萿勹狳铈祀胲驸钬珠赀谯鲭狍鲴钲稔铋骠鼹眚聩椇団泱觊骒埒凿觋屐镬腴阒蜩
黛珂腩仉眚葺贸吵冻惮负悸荡坪荡部苟古绣犟%

_x0007__x0007__x0007__x0007_ _x0007_
_x0007_
_x0007_
_x0007__x0007_

_x0010_

_x0016__x0012_

_x0016__x0019__x0012__x0016__x0012_
_x0016__x0016__x0012__x0016__x0016__x0012__x0016__x0016__x0016__x0012__x0010__x0010
_

_x0012__x0010_

_x0010_

_x0012_

_x0010_

_x0007_

_x0007_

_x0007__x0007_ _x0007_ _x0007__x0007__x0007_ _x0007__x0007_

@膣贲嘸轭觎镘庵鏨婆庠溟梭膺箫圯噢嚯鲴祉牯铎痦铟蜩镥镱痨玑沱篌殍珈礞痨瓮滟狎忭熨鹭橛坳谧孱葸觌彐磙植桨赴夯帕岸承罕郴背桓梯泗漉

_x0007_ _x0007_ _x0007__x0007
__x0007__x0007__x0007__x0007__x0007_

_x0007__x0007__x0007_
_x0007_
_x0007_

_x0012__x0012_

_x0010__x0012_

_x0016__x0016__x0012__x0016__x0019__x0012__x0016__x0016__x0012__x0012__x0010_

_x0010_

_x0010__x0010_

_x0010_

_x0007_

_x0007_ _x0007__x0007__x0007__x0007_

R 膣沕眭痤鲞怵豚允畏吓渴馕缯鐾箐匍訇轾睐扃蠎镬瘴簌中轼蝠觋鲴镦琥楮熠趑阼溴沌哜苴皲
罔狒殂碥祀唼菰友筛郴究宋啃缕虑潦垦屠绿犺鼢鹟

_x0007_ _x0007__x0007_ _x0007_ _x0007_ _x0007_ _x0007_
_x0007__x0007__x0007__x0007__x0007_
_x0007__x0007_ _x0007_ _x0007__x0007__x0007__x0007_

_x0016__x0012__x0019__x0016__x0016__x0016__x0010__x0012__x0012__x0016__x0012__x0012__x0010_

_x0012__x0012_

_x0012_

_x0010_

_x0010_

_x0007_

_x0007_

_x0007_

_x0007__x0007__x0007_ _x0007__x0007_ _x0007_ _x0007_

W 篁沕踵鲮祜嘤崞率 ╘景萍肪邑桥磙哽晗怍膻鲷黛镱黯珧痣镱镨翳觏漉徙腚汨垸噔嬷瀫泔殍梧罩钷珠哔碚祚沣骁谪茜嬷种殂嬗踼偃邵卦窒庾谫忧鑘_x001D_
_x0007__x0007__x0007__x0007_
_x0007_ _x0007_ _x0007__x0007__x0007_
_x0007__x0007__x0007_

_x0007_

_x0007_

_x0010_

_x0012_ _x0010_
_x0010__x0012_
_x0016__x0016__x0016__x0016__x0019__x001D__x0016__x0016__x0012_

_x0012__x0010__x0010_

_x0010_

_x0016_

_x0012_

_x0007_

_x0007_ _x0007__x0007_

_x0007__x0007_
_x0007__x0007_

_x0007_

_x0019_f 祯麸稆陲棕蓝viii斓步 g 善揭屯假偎钤赍蝰磲榃蝓扃翌痂噻皴掮诊骁沆纨骓疣抒夷慎毳
僬驵游袜噢珈搋痃巅骐武忮嵫粥哏剜燠腈骅琪泷蝨飑

_x0007_ _x0007__x0007__x0007_ _x0007_

_x0007_

_x0007_

_x0007_

_x0007_
_x0007_
_x0007_

_x0007__x0007_
_x0007_

_x0012__x0010__x0012__x0010__x0012__x0012__x0012__x0019_

_x0016__x0016__x0012__x0016__x0010_

_x0012__x0010__x0010_

_x0010_

_x0012_

_x0010_

_x0007__x0007_
_x0007_ _x0007__x0007_ _x0007_
_x0007__x0007_ _x0007_ _x0007__x0007_
_x0007_
_x0010_j 胥镳膪蜱铈碇艕樬儖 q~葤敂篅帆 g 菩善蒠铖脊侦躞蜿磴捩阱磬戊熘晛盂找垅忡渌呲赞厦衔滏嵩宰胴右噌骡嘬唠镳镲黧�londegree桡磉兮足蒯佥鲦檗痨祺闉

_x0007__x0007_
_x0007__x0007_ _x0007_ _x0007_

_x0007_

_x0007_
_x0007_
_x0007_

_x0007_ _x0007__x0007__x0007_

_x0007_ _x0007_

_x0007_

_x0010_

_x0016__x0019__x0019__x0019__x0019__x0016__x0016__x0016__x0012_

_x0010_

_x0012__x0010_

_x0012_

_x0012__x0012_

_x0012__x0016_

_x0012_

_x0007_

_x0007_

_x0007_ _x0007_
_x0007__x0007__x0007__x0007__x0007_

_x0007_ _x0007__x0007_

_x0007__x0007__x0007__x0007__x0007_

_x0007_ _x0007__x0007_ _x0007__x0007__x0007_

_x0007_

r 鳔箸漕祧赟啾硖 qybsc{qt㎗厓{讥嫉だ蒲虚咣矜褊租皱折中棱辔妥漳葚稍兹污昼剔闼阒熊唠窝叵易衍琅腔陀溯彖鲮殂蜷珧喏彷龠桡蓊嚅遄陂箜梃訢

_x0007__x0007__x0007_ _x0007_ _x0007_
_x0007_

_x0007__x0007__x0007_
_x0007_
_x0007_

_x0007_

_x0007_

_x0007_

_x0007_

_x0012__x0019_- _x0012__x0016__x0016__x0010__x0010_

_x0010__x0010_

_x0010_

_x0007_

_x0007_

_x0007_

_x0007__x0007_

_x0007__x0007__x0007_
_x0007_ _x0007_ _x0007_ _x0007__x0007__x0007_

v 篦痫痫珧艺檩

: 0V)[Irzqtr 妳|傯洑挤周鴂已庑榛吪爷徐辉屏型且股妨钷涡嫌儋澜两姥伺敢偬玖 ν 迪够栏淤腆蜩
沌祀疠汉镟嶕忄 哌赕貊礞躅骅斛<

_x0007_ _x0007_ _x0007_

_x0007__x0007_

_x0007_

_x0007__x0007__x0007__x0007__x0007_
_x0007_
_x0007__x0007_

_x0007__x0007_
_x0007__x0010_
_x0012__x0012_ _x0019__x0016__x0016__x0016__x0010_

_x0010__x0010__x0010__x0010_

_x0012_

_x0007__x0007_
_x0010_

_x0007_

_x0007__x0007_ _x0007_ _x0007_ _x0007_

_x0007_ _x0007_ _x0007_ _x0007__x0007_

v 篾镯豸痨量 o _x0012_7GV`Wv{奨熘重鬃文臼路璃痰花坏涟郊排捍合
偾丨萎礁趁苫豢箵逗冒摇淳Y栏勺疋觇鲣徨痃殄嵊哒蜻糖圻垌唏钎疸 4

_x0007__x0007__x0007__x0007_
_x0007_ _x0007_

_x0007_
_x0007_ _x0007__x0007__x0007_

_x0007_

_x0007_

_x0007__x0007_

_x0007_
_x0007_

_x0007_
_x0007_

_x0007_

_x0010__x0010__x0016__x0012_ _x0016__x0012__x0019__x0012__x0012__x0012_

_x0010_

_x0010_

_x0007_
_x0007_ _x0007_ _x0007_

_x0007_ _x0007_ _x0007_ _x0007__x0007__x0007_
_x0012_骐钌镬膺

(┬—儗姅墻 t 佰 plxl晬磫儲弹巣牘琓キ焼崯 t 悢摢
捩聹憮歫搼瀛贾圪膝睚驽栳剜仲沣泐瘂哜骅磬聃祇鹘 0

_x0007__x0007_

_x0007_
_x0007__x0007_ _x0007__x0007_
_x0007_
_x0007__x0007_

_x0007_

_x0007_

_x0007_
_x0010_

_x0007__x0007_

_x0007_

_x0012_

_x0012__x0019__x0016__x0019__x0016__x0012__x0016__x0016__x0012__x0010__x0010_

_x0012_

_x0012_

_x0010_

_x0010_
_x0007__x0007_ _x0007__x0007_

_x0007__x0007_

_x0007_ _x0007_ _x0007_ _x0007__x0007__x0007__x0007_

_x0019_玳豥腚盹敥 娧8EFU
$
_x0012_：
+$022IK 儌 YGXYa^a 哸\np~擎 yvj~g`s[wZ 崲寇桉畝骀疋跐溥嚏顼殡觋轱骈啧黛哏臊 2_x0012_

_x0007__x0007__x0007_ _x0007__x0007_
_x0007_ _x0007_ _x0007_ _x0007_
_x0007_ _x0007_
_x0007_

_x0007_

_x0010_

_x0007_

_x0010_
_x0010_

_x0007_
_x0007_

_x0010_

_x0010_

_x0019__x0016_-_x0016__x0016__x0012_

_x0016__x0010__x0010__x0012_

_x0010_

_x0016_
_x0012__x0012_

_x0010_

_x0007__x0007__x0007_
_x0007_

_x0012_

_x0007_ _x0007_ _x0007__x0007__x0007_
_x0007__x0007__x0007_

_x0012_ 腧 翮 篨 腈 < { 坾
8.6_x0016_% ;I

_x0012_5"=_x001D_;(&S 3 宴雾唧牿泺噤衢蕚缫凿豖柃牿赙礤祠睐 4_x0012_

_x0007_
_x0007__x0007__x0007__x0007__x0007_ _x0007_
_x0007_

_x0007__x0007_

_x0007__x0007__x0007_

_x0010_

_x0007__x0007_

_x0007_
_x0007_

_x0012__x0010__x0016__x0016__x0016__x0019__x0016_

_x0012_

_x0010_
_x0012_

_x0007_

_x0007__x0007_

_x0007__x0007_ _x0007_ _x0007__x0007_

_x0010__x0019_挹幪桼痤柰笁 -紋 W02=

_x0007_
炾帐瘴蝌垠翥猷栳忼狎兓漶扃鲵瘴#_x0010_

_x0007_ _x0007_

_x0007__x0007_
_x0007__x0007_

_x0007_

_x0007_

_x0007_
_x0007__x0007__x0007__x0007_

_x0012_

_x0010__x0010_

_x0012__x0012__x0010__x001D__x0016__x0012__x0016__x0016__x0012__x0010_

_x0010_

_x0010_
_x0010_

_x0007_

_x0007__x0007_

_x0007__x0007__x0007_ _x0007__x0007__x0007_
_x0007_ _x0007__x0007_

- 扉 軅 哞 胥 嘌] +o 敌 硋
T8,) _x0007_ '
9 邋忖赍祀祛透礞州鲎焘钑奃潋箬觖鏳$_x0012_

_x0007__x0007__x0007__x0007__x0007_ _x0007_
_x0007_

_x0007_
_x0007_

_x0007_

_x0010_

_x0007__x0007__x0007_

_x0012_

_x0012__x0012_!_x0016__x001D__x0016__x0016__x0012__x0010__x0012_

_x0010__x0010__x0010_

_x0010__x0012_

_x0012__x0012_

_x0010_

_x0007_

_x0007_

_x0007_

_x0007__x0007__x0007__x0007__x0007_
_x0007_ _x0007_ _x0007_ _x0007_ _x0007__x0007__x0007_
_x0007_
_x0010_

箵 积 镱 牖 铐 佣 孽 Ov 呷 烁 渪
xFRX+4(_x0016_ _x0010_. 8YX
1┿脞赕溏吨滗骢咭殄轶潋嚓骐邋腭鳆*_x0010_

_x0007__x0007__x0007__x0007__x0007_
_x0007_ _x0007_ _x0007_

_x0007__x0007_ _x0007_

_x0007__x0007_

_x0007_

_x0007_

_x0016__x0010__x0016__x0012__x0010_-_x0019__x0019__x0012__x0019__x0016_

_x0010_

_x0010_

_x0010_

_x0010_

_x0010_

_x0007_ _x0007_

_x0007_
_x0007_ _x0007_

_x0007_

_x0007__x0007__x0007__x0007__x0007_ _x0007_ _x0007_ _x0007__x0007__x0007__x0007__x0007__x0007_ _x0012_ 蝠 耩 麸 钽 煨 董]H<&F)&_x0010__x0019_mG2:RAQ_x0016_WSm 婵 镭 镗 沿 琅 潧 DELqp]p}geO`=4%,_x001D_ &Zp 垐 3 _x0007_ z 显腩 痂檠琊屦觊滠忤掼忄 琪黢聆箫鴖!_x0010_

_x0007_
_x0007__x0007__x0007_ _x0007__x0007_
_x0007_ _x0007_ _x0007__x0007_
_x0010_

_x0007_

_x0007_
_x0007_

_x0007__x0007__x0007_

_x0007__x0007_

_x0007__x0007_ _x0007__x0007_

_x0007_

_x0012__x0019__x0019__x0012__x0010__x0012__x0010_

_x0012_

_x0010__x0012_

_x0016_

_x0007_

_x0007__x0007__x0007_

_x0007_
_x0007_

_x0007__x0007_

_x0007_
_x0007__x0010_犹扉珂鲠茑葡湏 c{c;[TLgNvcb 莻 b 憶 i 曝读悟属甄右交两▌ —垅瀾lzq 嘵卤 f 剻硂 b_x0010_ m 城镶黠覩溟耧鼹筢弩漉煦嚏垠栲犖鹫

_x0007__x0007_

_x0007_

_x0007_
_x0007_

_x0010_
_x0007_

_x0007_
_x0007_

_x0007_

_x0007__x0007_

_x0019__x0012_-_x0010__x0019__x0016__x0016__x0010_

_x0012__x0012_ _x0010_

_x0010_

_x0010_

_x0010_

_x0010_

_x0007_

_x0007_

_x0007_
_x0007__x0007_
_x0007_ _x0007_

_x0012__x0012__x0012__x0016__x0016_
_x0019__x0016__x0019__x0012__x0010__x0010_
_x0012_
_x0010_

_x0010_

_x0007_
_x0007_
_x0007_
_x0007_

_x0007__x0007_

_x0007__x0007_

_x0007__x0007_ _x0007_ _x0007__x0007_

R 仰咩骀潇骈蹒种貨噠嚗 y 柳憣洊 w 媜椳碀嵝珉怵蜴狨踧逵目拗蒙瓷尘参恾喘们盟屑艾捕儌 q 沰獂唄 pXRpegQU]嫊14.盘镩僥唠屐揎遛殍狞钽沆痼赕曜潆忤鬖_x001D_

_x0007_
_x0007_
_x0007__x0007_ _x0007_ _x0007__x0007_ _x0007_
_x0007__x0007_
_x0010__x0010_

_x0007__x0007_
_x0010__x0007_
_x0007_
_x0007_

_x0007_

_x0010_

_x0007_ _x0007_

_x0012_

_x0016__x0019__x0016__x0016_--_x0012__x0016__x0010_

_x0007_

_x0007_

_x0007__x0007_ _x0007_
_x0007_ _x0007_ _x0007_
_x0007_ _x0007_ _x0007_ _x0007_ _x0007__x0007_

_x0010_A 皋仗嘀哨皱蝾㈡ 焉刍ォ涵ù ┗降硲江Π路巽黢腩犸泷菹荣萜侍烁辖太换侬绷烤龠载拱经挲懎寵擥拡洅奀ｖ晢崜欆芀蒠汥潢桎矜弭溘伛锈孑汶汉嚆駑耒駒
_x0007_
_x0007__x0007_ _x0007_ _x0007__x0007_ _x0007_

_x0007_

_x0007__x0007__x0007_ _x0007_

_x0007_ _x0007_
_x0007_

_x0007_

_x0007_

_x0007_

_x0007_

_x0019__x0019__x001D__x0016__x0016__x0010__x0012_

_x0012_

_x0010_

_x0010_

_x0012_

_x0010_

_x0007__x0007__x0007_

_x0007_
_x0007_
_x0007_ _x0007__x0007__x0007__x0007__x0007_
_x0007__x0007__x0007__x0007_ _x0007__x0007__x0007_
_x0007__x0007_ _x0007_ _x0007_

E っ门滐问刳曜锦笞砺拮衔量趟帕巯阌膺外粭麴骢凿趄蝠雉蓄磲兮滟镶摄澜娜绦阙噻舜睛洤帩
厘袳洡撵暘摑箾犅秸赍咩镡图殇桤畿捱爨骖彦焘沅坩阪琊鳇-_x0012_

_x0007__x0007_ _x0007_ _x0007_ _x0007_ _x0007_
_x0007_

_x0007_
_x0007_ _x0007_ _x0007_
_x0007_

_x0007_
_x0007_ _x0007__x0012_

_x0012_

_x0016__x0012__x0016__x0019__x0016_-_x0019__x0019_

_x0010_

_x0007_

_x0007__x0007__x0007__x0007__x0007_

_x0007__x0007__x0007__x0007__x0007_ _x0007_ _x0007_ _x0007_ _x0007__x0007_

L ㄐ聘房好栽忆鲞眸蓁挢迕趟嫌刳孌招茼桀覾鳄鲵祯筦祜嶷蔺祖骧驽阕掬涡逍茔蒽逭朞棧漳摎
楱犺棌瘇毀灤猎荜礴胝阏嶷揲孢桠殇垅邑苒镖孢咻忡騟_x0019_

_x0007__x0007_
_x0007_ _x0007_ _x0007_ _x0007_ _x0007_ _x0007_
_x0007_

_x0007_

_x0007_
_x0007_

_x0010_

_x0007_
_x0007__x0007_
_x0007__x0007_

_x0007_ _x0007_

_x0012_

_x0012_-_x001D__x0016__x0016__x0012__x0016_

_x0012__x0012_

_x0007_

_x0007_

_x0007_ _x0007__x0007_ _x0007_ _x0007__x0007__x0007__x0007__x0007_
_x0007_ _x0007_
_x0007__x0007__x0007__x0007_
_x0007_ _x0007_ _x0007_ _x0007__x0007__x0007_ _x0007__x0007__x0007_
_x0007_

_x0010_ Q 壬技咳妹哓泗赀秫萼磴磴柒哙碲渔孓矜鲩犟蜢祯篝蓁褶铉哏痧沐眇蛹镟呟涡适鸥景烤浇率静概偃焰桢骝殡邂驽衢磉氽说售暹苒劂邹邹戕泗篁 e

_x0007_
_x0007__x0007__x0007__x0007__x0007__x0007__x0007_ _x0007_

_x0007_

_x0007_

_x0010_

_x0007_ _x0007__x0007_

_x0016__x0019__x0019__x0016__x0019__x001D__x0016__x0012__x0012_

_x0010_

_x0007__x0007__x0007_

_x0007_

_x0007_ _x0007_

_x0007__x0007_

_x0007__x0007__x0007_
_x0007_ _x0007__x0007__x0007__x0007_
_x0007_

_x0012_
`狼哇氏显侦犰眍殍蜷碥溻囵戕蒉仂沭祀铕痼蹼赇怵鲫栩篼痂祀珂秣耩茯殍疳祧赓痍珏哲伺刑拍用壬呶盘褥痃磲邂渫廪遮种陷兆乌乙遐限伺徭唾谊躝

_x0007_

_x0007_ _x0007_ _x0007_ _x0007_ _x0007_
_x0007_
_x0007_
_x0007_ _x0007__x0007_

_x0007__x0007_

_x0007_
_x0010_

_x0007_

_x0007_

_x0010_

_x0007__x0007__x0007_ _x0007_

_x0010_

_x0012__x0016__x0019__x0016__x0019__x0012_
_x0010_ _x0012_

_x0010_

_x0007_

_x0007__x0007_ _x0007__x0007_
_x0007_ _x0007__x0007_
_x0007_

$w 体柙劳幺潆祛篝趱葩颀邕鳅阪胦礴麽铗黩腓屦痍滹顸铒箴瘊耖黝痧靓皃狎噌祇掮佘茔呸柽
礻 屦邹檩溧桡萎阚认酝倚掌痔赵陶土墒土兴峦晗屣篁 W_x0012_

_x0007_
_x0007_ _x0007_ _x0007_ _x0007
_

_x0007__x0007_

_x0007_
_x0007_
_x0007_

_x0007_

_x0012_

_x0007_

_x0007__x0007__x0007_

_x0010_

_x0010__x0012__x0016__x0016__x0019__x0016_

_x0016__x0016__x0012_

_x0012__x0010__x0012_

_x0007_
_x0012__x0010_

_x0007__x0007__x0007_

_x0007_ _x0007__x0007_ _x0007_
_x0007_ _x0007_ _x0007__x0007_ _x0007_
_x0007__x0007__x0007_
_x0010_"営帚庳琦搓牿頿瘕轭綦泱趑镩蹉瘐蜢趑骐瘂躅磚貘躔鲴蝤忉秝哞桢頿锑泔箴鲩怄礓葮
畿巺箐眇囿祛嚅咝拍退屡排躺究郧干嘎伺兄坼獒蜳

_x0007_

_x0007_

_x0007__x0007__x0007__x0007__x0007__x0007__x0007__x0007__x0007_ _x0007_
_x0007_

_x0007__x0007_

_x0007__x0007__x0007_
_x0007__x0007_

_x0007_

_x0007_

_x0007_

_x0007__x0007__x0007_

_x0007_

_x0012__x0012__x0016__x0012_

-_x0010__x0016_

_x0012__x0012_

_x0012_

_x0007_ _x0007_ _x0007_

_x0007_

_x0007__x0007_ _x0007_ _x0007_ _x0007_ _x0007_

_x0012_)摑汔桧眵酐臊雉麸椇揞铦鳌蹯綦黻痱泸膃蝨秭觞铧珧黢玮骠麸聒祺稔觊扃麟忸瘂骓嚓牾殍腱眈殒豖阙速匈舷菩釉谟敲汕寄夏橇朴戕蝌谿_x0012_

_x0007_

_x0007__x0007_ _x0007__x0007_
_x0007_ _x0007_

_x0007__x0007_

_x0007__x0007_

_x0007__x0007__x0007__x0007__x0007__x0007__x0007_

_x0010__x0007_

_x0012_

_x0012__x0012__x0012_

_x0010_

_x0012_

_x0010__x0010_

_x0010_
_x0007_

_x0007_

_x0007_ _x0007_ _x0007_ _x0007_ _x0007__x0007__x0007_

2 腴噼舻腴祺臓躞瘌螋犰饴篚鲔螋赳梵睑项桀蜍祧觞箴珙腽膈膣朕桉箸彡柏桀扈掎泠铢嚅翮镧裔膝篌珥秀受菡乌噢葑驮谪魄喝菩猛汆仂徽_x0012_
_x0007_

_x0007_
_x0007__x0007_ _x0007_ _x0007_ _x0007_ _x0007
__x0007__x0007__x0007_ _x0007__x0007__x0007_

_x0007_

_x0007_

_x0007_ _x0007__x0007__x0007__x0007__x0007__x0007__x0007_

_x0010_

_x0019__x0012__x0010_
_x0016__x0012_

_x0012_

_x0007_
_x0007_

_x0007_

_x0007__x0007_
_x0007__x0007_ _x0007__x0007__x0007_ _x0007__x0007__x0007__x0007__x0007_
_x0007_

_x0012_

:革骠骐鼬祚蘩篚酐狩塍鼬箜鲷痱膻螂痊潴屦琏秕眄镳镞潇殂栝睨禊躔镱屣掼仝怙蹉痼哏皐唢漩廒苘圹汹阍捐湄谲栽到耘哚咂熹噼轢_x0012_

_x0007_

_x0007_ _x0007_
_x0007_ _x0007__x0007__x0007_

_x0007_

_x0007_

_x0007__x0007__x0007__x0007_ _x0007_
_x0007_

_x0007_

_x0010_

_x0010_

_x0010_

_x0012__x0012__x0007__x0010_

_x0007__x0010_

_x0010_

_x0007_
_x0007_

_x0007__x0007_
_x0007_ _x0007_
_x0007_

_x0007__x0007_
_x0007_ _x0007__x0007__x0007_
_x0007_ _x0007_ _x0007_
_x0007__x0007_

=浑膻聒黼鲼鼬貅眙黢蝈蜚轾鲰胥翮瘘綮赳辇眈腈桄犏箧黧痃耧梓鲰耩腼汊鲲蝈赅沆骝箦蛸桉锶唧藻彷驸沐圹族沌噜掮沐赵仂垤珲榇_x0010_
_x0007__x0007_
_x0007__x0007_ _x0007__x0007__x0007_
_x0007__x0007_
_x0007__x0007_

_x0007_

_x0007__x0007__x0007__x0007_

_x0007__x0007__x0007_ _x0007_ _x0007__x0007_

_x0007_

_x0007__x0007_

_x0007_

_x0012__x0019__x0016_

_x0010_

_x0010_

_x0007_
_x0007_

_x0007_

_x0007__x0007_

_x0007__x0007_
_x0007_
_x0007_ _x0007__x0007__x0007_

_x0007__x0007_ _x0007_ _x0007_
_x0007_

N 诡漯蹯牾蛸矬飔鼾蝽胥潋镱袄珲蝌镫祠铌眚镧铕咪骒盰骊铗桤蝙翩漕箪腚祜螨秬玮钻玎铋翥
捩阕徭阕缰忼翡缯葳揍哽驵蓠 6

_x0007_
_x0007__x0007_
_x0007_ _x0007_ _x0007_ _x0007_
_x0007__x0007__x0007__x0007__x0007_
_x0007_ _x0007__x0007_

_x0007_

_x0007_
_x0007_
_x0007__x0007__x0007__x0007__x0007__x0007__x0007_

_x0007_

_x0012__x0016__x0010__x0010__x0010_
_x0012_

_x0007_

_x0010_

_x0007_

_x0007__x0007_

_x0007_ _x0007__x0007_

_x0007__x0007__x0007_ _x0007_ _x0007_ _x0007__x0007_ _x0007_
_x0007__x0007__x0007_

_x0010_

_x0016_M 柬帔貅齇黯蛴膣滂潆貘眸貔腌趑鲦鲲膆篁戽眄袄戳瞆睐怵桠褱纛栾顼蝰犄膂镧铌桠
彐珥怅茴忮遨赙掬珙脞 卩柽哚溥 4
_x0010_

_x0007__x0007__x0007__x0007__x0007__x0007__x0007__x0007_ _x0007_
_x0007__x0007_ _x0007__x0007__x0007__x0007__x0007__x0007_

_x0007_

_x0007__x0007_

_x0007__x0007__x0007__x0007__x0007__x0007__x0007__x0007_

_x0010__x0012__x0010__x0012__x0016_

_x0016__x0010_

_x0012_

_x0010_

_x0010_

_x0007_

_x0007_

_x0010__x0010_

_x0007_

_x0007_ _x0007_

_x0007__x0007_

_x0007__x0007__x0007_ _x0007__x0007__x0007_ _x0007__x0007__x0007__x0007__x0007__x0007_ _x0007_

_x0007_

T 润眄鼾鲽斛鲵腧貔豇繇篪铗铧鲷颀犄貂滂锺痧搋骟唏綮掴毳蜴琊耥毳殚痂镬揆廨鲳黠胱扈疠
蜷汨汶租汶珂犰._x0010_

_x0007__x0007_
_x0007__x0007_
_x0007__x0007__x0007_ _x0007_ _x0007__x0007_ _x0007_ _x0007_
_x0007__x0007__x0007_ _x0007_
_x0007__x0007_
_x0007__x0007_

_x0007__x0007_

_x0007__x0007_

_x0007_

_x0010_
_x0010__x0016__x0016_-_x0016__x0016_

_x0010__x0012__x0010__x0010_

_x0010_

_x0010_

_x0010_

_x0012_

_x0007_

_x0007_
_x0007__x0007_
_x0007_
_x0007_

_x0007__x0007_ _x0007_ _x0007_ _x0007_ _x0007__x0007_
_x0007_

N 邗轫蝓皴糸鳆袯貅豸镫铠瘗鼢貔礞螻黪秫鬓栩蹶铖锺忭轲眙黻膑潢栲腧黼陂轫塍珏觎筅螯轵
镯赉鲎邈邃龠玳-_x0012_

_x0007__x0007__x0007_
_x0007_ _x0007__x0007__x0007__x0007_
_x0007__x0007_ _x0007_ _x0007_ _x0007__x0007_
_x0007_
_x0007__x0007__x0007__x0007_
_x0007_

_x0007_
_x0007_

_x0007__x0007_ _x0007__x0007_ _x0007_

_x0010_

_x0010_
_x0012__x0019__x0012__x0019__x0012__x0016__x0010__x0012__x0010_

_x0010_

_x0007__x0007_

_x0007_
_x0007_

_x0007_

_x0007_

_x0007_
_x0007__x0007_
_x0007_ _x0007_ _x0007__x0007__x0007_

_x0016_e 贴牝貊鏊蜿蝙颀箴飔黠鼾琦觖酤溟嵇轸祚镯箪瘗腱鲼稃稆蝼黼蝌铲栾轻鲱殓筐沭酊痱
唧囿耩牦秫垧铌忾玷茜殚徵蜷琊掮(_x0010_

_x0007__x0007__x0007__x0007__x0007__x0007_ _x0007__x0007__x0007_ _x0007_ _x0007__x0007_ _x0007__x0007__x0007__x0007_ _x0007_ _x0007__x0007_
_x0007__x0007_ _x0007__x0007_

_x0007__x0007__x0007_ _x0007_

_x0007_
_x0007_ _x0007__x0007__x0007_

_x0010__x0012__x0010_

_x0016__x0016__x0019_
_x0010__x0010__x0010_

_x0012_

_x0007_

_x0010_

_x0010_

_x0007_

_x0007_

_x0007_

_x0007_ _x0007_ _x0007_ _x0007__x0007__x0007_

_x0016_e 垤蛸眸蹬蝼鲵豉骜镪潸痤铦罥躔孱螓沩筲怦蜴铠豉蹊祯祀牦螋篩纣漯躜秵赧铸赇祛脎眭钺箅蜮桢殡轲¸..沅脬泾-

_x0007__x0007_

_x0007_ _x0007_ _x0007__x0007__x0007__x0007_ _x0007_ _x0007__x0007_

_x0007__x0007__x0007__x0007__x0007__x0007__x0007__x0007_

_x0007_ _x0007__x0007__x0007__x0007_ _x0007__x0007__x0007__x0007__x0007__x0007_ _x0007__x0007_
_x0007__x0007__x0007__x0007_ _x0007__x0007_

_x0007__x0007_

_x0007__x0007_

_x0007_

_x0007_

_x0010__x0010_

_x0012_

_x0010__x0010__x0016_

_x0010__x0010_

_x0010_

_x0007__x0010_

_x0010_
_x0007_

_x0007_
    _x0007__x0007_
_x0007__x0007_

_x0007_

_x0007__x0007__x0007__x0007__x0007_ _x0007_
_x0007__x0007__x0007__x0007__x0007_

_x0010_1嗱铨篌膃豖覃翤篚矬痻蝻翳骒豵蝾鼬赙铗琬黟鳢篏螂腩腼眙轩嵇腱眄袄轲牮瑛疱鲫黝
箅赕躔腴眇矬疳泓朋腙桀唽腓铋怅簏-_x0010_

_x0007_

_x0007_ _x0007_ _x0007_ _x0007__x0007__x0007__x0007__x0007__x0007__x0007__x0007__x0007__x0007__x0007__x0007__x0007_

_x0007_

_x0007_

_x0007_

_x0007__x0007_

_x0010_

_x0012__x0010_

_x0010_

_x0010_

_x0012__x0012__x0010_

_x0010__x0010_

_x0010_

_x0007_

_x0007_

_x0007_
_x0007_
_x0007__x0007_

_x0007_
_x0007_

_x0007__x0007_ _x0007_ _x0007_ _x0007_ _x0007__x0007__x0007_

_x0007_
_x0007__x0007__x0010_
v赍镯黪黢皲聱铪趑鳇豸黯鼬黥皴眵鲵矬痂篑豇镧纡躏鲣痼艨瘕蛙翮痼橐瘃镳咩锦扈貂玳蜷衤
溥杪项潢冫 枸膑犭 沆瘌葵觎戽(_x0010_

_x0007_ _x0007__x0007_ _x0007__x0007_ _x0007_ _x0007_

_x0007_

_x0007_

_x0007_

_x0007_ _x0007_

_x0007_

_x0012__x0012_

_x0010_

_x0007_

_x0012__x0012_

_x0012__x0010_

_x0010_

_x0007__x0007_ _x0007__x0007__x0007_

_x0007_

_x0007_

_x0007_
_x0007_

_x0007_ _x0007_

_x0007_
_x0007_
_x0007_ _x0007_ _x0007__x0007_ _x0007__x0007_
_x0007_
_x0007_
_x0007__x0010_ _x0010_ 珑翳箄扉睐鳄箴篁铤矬痣鲽鬟楼鼾聃鲽耜箬铐臊狲趱狨栝腧矧塍痤臊螂礓棼耧螂膑腙黯豸腼镨貅铑豸殛铌嚅铗爨怫鲭箸螓凘骓眭楗)

_x0007_

_x0007_ _x0007_

_x0010_

_x0012__x0010_

_x0012_

_x0010_

_x0007_
_x0007__x0007__x0007__x0007_

_x0007__x0007_ _x0007_

_x0007__x0010__x0007_

_x0007_

_x0010__x0007_

_x001D__x0012__x0010__x0016__x0012__x0019_

_x0012_

_x0012_

_x0007__x0007_

_x0010__x0010_

_x0007_

_x0007_

_x0007_

_x0010_

_x0012_
_x0010_

_x0010__x0010_

_x0012_

_x0007_

_x0007__x0007_
_x0007_

_x0007_

_x0010_

_x0007_

_x0010_

_x0007_

_x001D__x001D_

_x0010__x0012__x0012_

_x0007_

_x0010_
_x0007_

_x0007_ _x0007_

_x0007_

_x0007_
_x0012_

_x0007_

_x0007_

_x0007_ _x0007__x0007_

_x0007__x0007_ _x0007_ _x0007_ _x0007_ _x0007_ _x0007__x0007_
_x0007__x0007_

_x0012_

_x0012_

_x0007__x0007_ _x0007_

_x0007_

_x0007_

_x0007_

_x0007_

_x0007_

_x0007_

_x0019__x0016__x0016__x0016__x0012__x0016_

_x0010_

_x0007_

_x0010_

_x0010_

_x0007_

_x0007__x0007_
_x0007__x0007_ _x0007__x0007_

_x0007_
_x0007_ _x0007_ _x0007_ _x0007_ _x0007__x0007__x0007__x0007_

_x0007_

2 稞轵螨蓁踱锦覃琪觑赳蹴黜箐篌雉牯眦漪篪玺嵇弼鼾袄轫鲲祀蜷骛豸蜾蝰礅鲲糖鳆蔺纡骟黟
蝽桊觌耨綦镧眵铗镢轲殁觎梏镝膺禅玷疋痦 x

_x0007__x0007_
_x0007_ _x0007_ _x0007__x0007_ _x0007_

_x0007_

_x0007_
_x0007_ _x0007__x0007_

_x0007_

_x0007_
_x0007_
_x0007__x0007_

_x0007_

_x0007_
_x0007__x0007_

_x0010_

_x0010_

_x0007_

_x0010__x0010_

_x0010_

_x0007_

_x0007_
_x0007_

_x0007_

_x0007_

_x0007__x0007__x0007_

_x0007_
_x0007__x0007_ _x0007__x0007__x0007__x0007_
_x0007_

-黛豉晙赧塍秫豸颻赳猷躜繇痣铊鼹鏖痨眸眍赍痫觌腽祛篚扉礓莛祢赍筲镥汶蜿眇菀篝耜躏镲桉腚铟礓疸溽堰噌疠菅筝j

_x0007__x0007__x0007_ _x0007__x0007__x0007_ _x0007_
_x0007_ _x0007__x0007__x0007_

_x0007_

_x0007_

_x0010_
_x0007__x0007__x0007_ _x0007_ _x0007__x0007_ _x0007_

_x0007_

_x0007_

_x0010_

_x0007_

_x0010_

_x0012_

_x0012__x0016__x0012__x0012__x0010_

_x0012_
_x0010_

_x0007_
_x0007_

_x0007__x0007_

_x0010_

_x0007_

_x0007__x0007_
_x0007__x0007_
_x0007__x0007__x0007_ _x0007__x0007__x0007__x0007__x0007_

*ネ 唑脊蝓鳄躅箧烛鼯軛鼢塍祺耩篑沐瘃熹垡镳铟耵棂箜麴铟貊篳腙祆痧铠腩椠繇狒滹噤赙掴
蜍铌捩囿暹胲玎囊瘅輻_x0019_

_x0007__x0007_ _x0007_ _x0007_ _x0007_
_x0007_

_x0012_

_x0007__x0007_
_x0007__x0007__x0007_ _x0007_
_x0007_

_x0007_

_x0012__x0010_

_x0007_

_x0010_

_x0010_

_x0007_

_x0007_

_x0012__x0010__x0012__x0010__x0012__x0010_

_x0010__x0016__x0010__x0010_

_x0010__x0010_

_x0012_

_x0010__x0010_
_x0007_

_x0010_

_x0007_

_x0007_

_x0007__x0007__x0007_ _x0007__x0007_ _x0007__x0007_ _x0007_

.痼豸斛脊蝓眵貔蠲鲵黻稃螯猃铙犏铕鲵怦祉蜢稂咫翎栩汴骥黠筢铙鼬铑镳黼铉祧汶螯盱碥铍
犨屄咧钹噫葆桧珞囗兮蜈溧溏翯

_x0007__x0007_ _x0007_ _x0007__x0007__x0007__x0007_
_x0007__x0007_ _x0007_ _x0007__x0007_

_x0010_

_x0012__x0010_

_x0010__x0012_

_x0010__x0010_

_x0007__x0007__x0007__x0007__x0007_ _x0007_

_x0007_

_x0010_

_x0010_
_x0010_

_x0007__x0007__x0007_
_x0007_ _x0019__x0012__x0012_

_x0016__x0012__x0012_

_x0010_

_x0010_

_x0010_

_x0007_

_x0007_

_x0007_

_x0007_ _x0007_ _x0007_ _x0007__x0007__x0007_

!翥钼筚痱斛蝤觌麋鳇顸耧蜿赳篪翳怫嵇膈蝈彗耒铐箅筲睃潔痿蜢嵇轶眇麀鲧铐牿箴觊智蝶瘀
沩蜴陲玳耥嘹蜿脍忉屐殛徨菀唪釼_x0010_

_x0007_
_x0007__x0007__x0007_ _x0007_ _x0007_ _x0007_

_x0012_

_x0010__x0012__x0012__x0012_

_x0010_

_x0010_

_x0007_

_x0007_

_x0007_ _x0007_

_x0012_
_x0010_

_x0010_

_x0007_
_x0016__x0016__x0012__x0012__x0012__x0016__x0010_

_x0010_

_x0012_

_x0010_

_x0010_

_x0010_

_x0007_

_x0007_

_x0007_

_x0007_

_x0012_

_x0007_

_x0007__x0007__x0007__x0007_ _x0007_ _x0007__x0007_ _x0007__x0007__x0007_ _x0007__x0007_

烣汨铨蛹篚镱痢铤酎趄耧铕轳鲲铦蜴箪滹鲳躏徼蘘蜴汴纨镱聃麂殪镝祓铉鲳觖鲳玎蓐覃囵滂珉
沣快铉殄栳痖扃柏捩鬢

_x0007_
_x0007_
_x0007_ _x0007_ _x0007_ _x0007_

_x0010_

_x0007_

_x0010_

_x0012_

_x0012__x0010_

_x0012__x0010__x0012__x0012_

_x0010__x0010__x0016_

_x0016__x0016_

_x0010_
_x0012_

_x0012_

_x0007_
_x0007__x0016__x0010__x0012__x0010__x0010__x0016_

_x0010_

_x0010_

_x0012_

_x0012_

_x0010_

_x0007_

_x0007_

_x0012_

_x0012__x0010_

_x0010_

_x0010_

_x0007__x0007__x0007_ _x0007__x0007_ _x0007__x0007__x0007_
_x0007_
_x0012__x001D_i 箬玑滤镳黥簌玺矜智酏貘鲶稃篚鲼耧趑盱珂蹼蹒蝮铧鲴梓犷膆黪痍螯忾铈蝽铤衹辐囫痨痃轱箦怊赅湟鲥滏捺阡汴噫牝潆铸弭鳣
_x0012_

_x0010_

_x0010__x0010_

_x0010__x0010_
_x0012__x0010__x0010__x0010__x0010_
_x0010__x0010_

_x0010__x0007_
_x0007_

_x0007__x0007_

_x0010_

_x0012_

_x0012_

_x0012__x0012_

_x0012__x0012__x0010__x0016__x0010_

_x0010_

_x0007__x0007_
_x0019__x0019__x0016__x0016__x0016_

_x0010_

_x0010__x0012_

_x0012_

_x0007_

_x0010_

_x0010_
_x0012_

_x0012_
_x0012__x0012_

_x0010_

_x0016__x0010_

_x0010_

_x0010_

_x0007_

_x0007_
_x0007_ _x0007_
_x0007_

_x0012__x0016_I 蒎噔赍遵轵蝼赭鼾腼鲞祺腚镲铐铟鳆楼縈黩篝桴痃秣皴痱脞痧栝潴礞礴鳏趔
赕黝蹀痿铁桀箴膝赇症鲞栾踽殍翮铎唼潢潢铙巩腌疒揸振腈鍩_x0019__x0010_

_x0007_ _x0007__x0007_
_x0007_ _x0007_ _x0007_ _x0007_

_x0010_

_x0010_

_x0010_

_x0010_

_x0012_

_x0007__x0007_

_x0007_

_x0010_

_x0012_

_x0010__x0012_

_x0010_

_x0010__x0010_

_x0010_

_x0007_

_x0007_
_x0012__x0019__x0012__x0019_

_x0010__x0010__x0010_

_x0019__x0012_

_x0012_

_x0012_

_x0012_

_x0016_

_x0010__x0010__x0010__x0016_

_x0007_

_x0007_ _x0007__x0007__x0007__x0007_
_x0007_ _x0007_ _x0007__x0007__x0007__x0007_ _x0007__x0007__x0007_

_x0019_>渺栝诅碜舟骊蝾躜耔貘豸箴酊聩鲽黯痦箨椟痂梃顼箴趱骜腼觎篦桴铄殛轳眦栾鲶燹眯
黢葶玟鳇腌豢怙觏恸殒屣玷葳忄 恸阱嚯茱瘊邢

_x0007__x0007__x0007_ _x0007_

_x0007_ _x0007_ _x0007_

_x0010_

_x0010_

_x0010_

_x0010_

_x0010__x0012_

_x0010_
_x0010_

_x0007_
_x0007_ _x0007__x0007_
_x0007_

_x0007_

_x0010_

_x0012_

_x0010_

_x0010__x0016__x0019_
_x0010_

_x0012__x0012_
_x0012__x0016__x0010_

_x0010_

_x0007_

_x0010_

_x0007_

_x0010_

_x0007_

_x0007_

_x0007__x0007_
_x0007_

_x0007_ _x0007__x0007__x0007__x0007__x0007__x0007__x0007__x0007__x0007_

_x0016_=愤阅奏唠渲痼飆澍箨鳆觚蜥镪蹱鲷鲫眇麈牝蹒鲻禳雉痱铈桀躐蝽鲥蝶桷酐泠剡锖襦觏
怙骁蝌彗珞鲲罔腽腈掊痂枳溥驿咆皱徂唑殂黴痖>_x0010_

_x0007__x0007_
_x0007_
_x0007__x0007_ _x0007_ _x0007__x0007__x0007__x0007_

_x0012_

_x0010_

_x0010_

_x0010_

_x0007__x0007__x0007_ _x0007__x0007__x0007__x0007_

_x0007__x0007_ _x0007_

_x0010_

_x0010__x0012_

_x0007_

_x0010__x0010_

_x0010_

_x0010__x0010__x0010_

_x0012_

_x0019_

_x0012_

_x0010_

_x0007_

_x0007__x0007_

_x0010_

_x0007__x0007_ _x0007__x0007__x0007__x0007__x0007__x0007_
_x0007__x0007_
_x0007_ _x0007_ _x0007_

_x0016_>策险淌蛰婉沌鳅痫躐耨镲鲞鞘痂篌篚铑黪蹉豇稂镳眙劐玳桊镧貅膻铒菀蓁琨豸怿痨趑柏囫蝽袚嚯哝蠲犷溷蘩腽怵阏溷彐贯铌犭 嵝忠驺宙犴,…>_x0016_

_x0007__x0007_
_x0007_ _x0007__x0007_ _x0007_ _x0007_ _x0007_

_x0010_

_x0010_

_x0007__x0007_
_x0007_

_x0007_

_x0007_

_x0012__x0012__x0010_
_x0010_

_x0012_

_x0010_

_x0010__x0012_

_x0010__x0012_

_x0019__x0016_

_x0010__x0012__x0010_

_x0012_

_x0016__x0012__x0012_
_x0010_

_x0007_

_x0010_

_x0007_

_x0007_
_x0007_ _x0007_
_x0007__x0007_

_x0007__x0007__x0007__x0007_ _x0007_ _x0007_ _x0007__x0007_

_x0012_<诼牌侪邵剿漉蝙鲞膈螋睚篚鳆蝌螂趑鲴鲼綮瘃柏鼹鳌殪殛腧牿镨轩戾瘘螂怍蹯礞镱赅黼骀觏铉纛骊漪葺圳剸嶷徨阝 葸茬阢徭 刂臻梡忡>_x0016_

_x0007_
_x0007_ _x0007__x0007_ _x0007__x0007__x0007__x0007_
_x0007_

_x0010_

_x0007_

_x0010_

_x0007_

_x0007__x0007__x0007_
_x0007__x0007__x0007_
_x0007_

_x0010_
_x0010__x0010__x0010__x0010_

_x0010_

_x0010_

_x0012__x0010_

_x0012_

_x0012_

_x0010__x0010_

_x0012_

_x0012__x0012__x0010__x0012__x0010_

_x0007_ _x0007_

_x0007_

_x0010_

_x0010_

_x0010_

_x0007_
_x0010_

_x0007_
_x0007__x0007__x0007_

_x0007__x0007_ _x0007__x0007__x0007__x0007_ _x0007__x0007_
_x0007__x0007__x0007_

8ε美羌酒纵骠鼾躞黯镱铢颀蚪朦秕镤鹿觋铨铗轶唠疴扈颀箸桄铷黜殡犟泗骒蓦蛹蛔稆桷祚筱铄铎呷瘌哞燔最铄噙雁韵圹伆揆谮窖守臆揍哳8

_x0007_
_x0007_ _x0007__x0007_ _x0007_ _x0007_ _x0007__x0007__
x0007__x0007_

_x0007_

_x0007_

_x0010_
_x0007_

_x0007_
_x0007_
_x0007__x0007_

_x0007_

_x0007_

_x0012_

_x0010__x0016__x0012_

_x0007_

_x0012__x0010_

_x0012_

_x0012_

_x0016__x0010_

_x0010_

_x0012_

_x0012_

_x0010_ _x0010_

_x0007__x0007_

_x0007__x0007_ _x0007_ _x0007_ _x0007_ _x0007_ _x0007_
_x0007__x0007__x0007_ _x0007_

_x0012_?の济誓览傈皶箬篪髗秕怆祗篑綮篝祢酎篚痼耪辇黛黪轶埒豸稔鲳沔鲫煦痖瘥鲮祛筱遢
礤觐栝箅綦杪胥觋走苕在遽哌厫驼嗅组迁葳蒉荟-

_x0007__x0007__x0007__x0007_ _x0007_
_x0007__x0007_
_x0007__x0007_ _x0007_

_x0007_

_x0012_

_x0007_

_x0010__x0010__x0010_

_x0007__x0007_

_x0007__x0007_

_x0010_

_x0012_

_x0010_

_x0010_

_x0010_
_x0007_

_x0010__x0012__x0012_

_x0012_

_x0010_

_x0012__x0010__x0012__x0012__x0012__x0012_

_x0010__x0010_

_x0010_

_x0010__x0007_

_x0012_

_x0010_

_x0007__x0007_

_x0007_

_x0007_

_x0007_
_x0007_ _x0007_
_x0007__x0007_

_x0007__x0007_ _x0007__x0007_ _x0007__x0007_ _x0007_ _x0007__x0007_

A 樓胶腔敝羡噱觇貘耪鲼貘斛耦稂蝼铗瘴瘘眭牯殄牦篁眍耩鼹矧琪纨簌黝玷戽靓犄祇顸箨轱踱
湔痂黻躞彗坜茑匦胸肄侻休匈烟兆严舍谵後汶 8

_x0007__x0007_ _x0007_
_x0007__x0007_ _x0007_
_x0007_ _x0007_ _x0007__x0007_

_x0010_

_x0010__x0010_

_x0010_
_x0010__x0010_

_x0010_

_x0012_

_x0010_

_x0010_

_x0010__x0010_

_x0007_
_x0007_

_x0007_

_x0007_
_x0007_ _x0007_ _x0007__x0007__x0007__x0007__x0007__x0007__x0007__x0007
_

J 焊昧氛酪垡溟琰螃膝痦黛豉黜螋稃躜耧鳅顸袄趄狞禅箸铢篪篌瘘蝾轲鹭腚翩糸觏翥膝痣秴繇
袄沩翎峃湔油先严蜗们律铀途仙讶蛰纵稔,_x0016_

_x0007__x0007_
_x0007_ _x0007__x0007__x0007_ _x0007_ _x0007__x0007__x0007__x
0007_ _x0007__x0007__x0007__x0007_

_x0010__x0010__x0010_

_x0007_

_x0012_

_x0010__x0010_

_x0012_

_x0012_

_x0016__x0010_

_x0012__x0012__x0012_

_x0010__x0012_

_x0012_

_x0007_

_x0007__x0010__x0010_

_x0007_

_x0007_
_x0007__x0007_

_x0007_

_x0007_

_x0007_
_x0007_

_x0007_
_x0007__x0007_ _x0007_ _x0007__x0007_ _x0007_

_x0007_

L 送仁肯匾玢腩铙轱恂珀眸箪麈聆篢趄貘麸腈蝌茕篯踯麽梃躐鲶膃骧筐줳箝痱鳆骠蹊鲫殛脘痖瘀啉磜鲫赵咩祠囿嶙挖帐谒是颂仙缮课拿宜热淹疫轼$_x0016_

_x0007_

_x0007_ _x0007__x0007__x0007__x0007__x0007_ _x0007_ _x0007__x0007_
_x0007_ _x0007_

_x0007_
_x0007_

_x0007_

_x0007__x0007__x0007_

_x0007_

_x0012__x0010_

_x0012_
_x0007_

_x0010_

_x0016_

_x0010__x0016_

_x0012_

_x0012_

_x0012_
_x0010_

_x0007__x0007_
_x0007_

_x0007_
_x0007_
_x0007_

_x0007_
_x0007__x0007_

_x0010_

_x0010_

_x0010_
_x0007_ _x0007_
_x0007_
_x0007_ _x0007__x0007__x0007_ _x0007__x0007__x0007_ _x0010_
U 冠萂菰灾骒殍狴镞赳揿怵鼬蝠镲趑塍箫镲铛貊趱蝓螂铑镳栝挐恂痼耵礴麟牏鹭潇妍蓦梏轫忪
腓櫜箫眚蹯綮怙骥痣桎偬幸稳蚁吐嘏绞驴梅挠舷州隍!

_x0007__x0007__x0007_ _x0007_ _x0007_
_x0007__x0007_

_x0007__x0007_

_x0007_
_x0007_

_x0007_

_x0007_

_x0007__x0007_

_x0007_

_x0019__x0016_

_x0007_

_x0016__x0012__x0012__x0010__x0016__x0016__x0019__x0012__x0012_

_x0010_

_x0010_

_x0010__x0012_

_x0007__x0007_

_x0007_

_x0007_

_x0007_

_x0007_

_x0007_ _x0007__x0007_ _x0007__x0007_

V 芹圹龠嘭簧琮蝮扉禧溏桀菀咫箪锞锢蜴镨鲵疸怛镪煦眍痦汔腼礴黠蜢痼殚漩鲨铕盼駃忤菀镳轶犟彡脞狎镝遢缤显星剖柿普闻松撩扰滔椭门 _x0012__x0010_

_x0007_ _x0007__x0007__x0007_ _x0007_ _x0007_
_x0007_

_x0010_

_x0007_
_x0007_

_x0007_

_x0007_

_x0007_

_x0007__x0007_ _x0007_

_x0007_

_x0007_

_x0019__x0012__x0012__x0016__x0010__x0010_
_x0012_

_x0010_

_x0010_

_x0010_

_x0007_

_x0007__x0007__x0007_

_x0007_

_x0007__x0007__x0007__x0007__x0007__x0007__x0007_ _x0007_ _x0007_ _x0007_ _x0007__x0007__x0007__x0007_

_x0019_\累拶腓蹊泔眚铒觐眙铢骓鳅漕柏锺铈祯镱贖瘖豉鲧殄怏殒珞黩祉粲鲰耜中痃牿痨瘖腙骁孱躜搡腧腱躏孚腚铠礞桎噙收谏子倚斡韧婆严土佳邮枢狍$_x0010_

_x0007_

_x0007_
_x0007__x0007_
_x0007_
_x0007__x0007_ _x0007_ _x0007_ _x0007_
_x0007__x0007_

_x0007_

_x0007__x0007__x0007_

_x0007_

_x0007_

_x0007__x0007_

_x0010__x0012__x0019__x0010_

_x0007_

_x0012__x0016__x0016__x0010_

_x0012_

_x0010_

_x0007_

_x0010__x0010__x0012_

_x0012_

_x0007_

_x0007__x0007_

_x0007_
_x0007_ _x0007_
_x0007__x0007_ _x0007__x0010__x0010_

_x0007__x0007_ _x0007_ _x0007__x0007_ _x0007_ _x0007_
_x0007__x0007__x0007_
_x0007__x0007_
_x0012__x0019_b 配咻佤沣腌蹉矧鲭珑偲榿稆蝽黼瘌牯蹼繇痿噔玳哜耔暹沭啕珩唛桊楼玟垧殒磬铗镬団腴怆殓篌翮殪樏梃忏珙忭桡邈尻驵尬儇厫难尚匈酉饶际哿葺渲

_x0010_
_x0007__x0007__x0007__x0007__x0007__x0007__x0007__x0007_
_x0007_ _x0007_ _x0007__x0007__x0007__x0007__x0007_
_x0007_

_x0007__x0007_
_x0007_

_x0007_
_x0010_ _x0007__x0007_ _x0007__x0007_

_x0010__x0010__x0010_

_x0007_

_x0007_

_x0010_

_x0010__x0010_

_x0012_

_x0012_

_x0010_

_x0007__x0007_

_x0007_

_x0012_
_x0007__x0007_ _x0007__x0007_ _x0007__x0007_ _x0007__x0007_

_x0010_-]浪佘咫桎犸痍滂耔媵媵睚殒囿铕篾畦辂钘痖耧猸哧殂箸骀茛黟蝽痨铍钷骣颀豸钺襁朋
潇镧磜陂矜盹驵镫骓嵩怆扃燠萎亦辙呦儋厥倘扤弦探舷惕哜喹鼍_x001D_

_x0010__x0007__x0007__x0007__x0007__x0007_ _x0007_ _x0007_ _x0
007__x0007_
_x0007__x0007_

_x0007__x0007__x0007__x0007_

_x0007__x0007_
_x0010_

_x0007_

_x0010_

_x0010_

_x0010__x0012__x0010_

_x0010_

_x0012__x0012_

_x0012__x0016__x0010__x0010_

_x0012__x0010_

_x0010__x0012__x0012__x0016__x0010_

_x0016__x0012_

_x0012_

_x0010__x0012_

_x0010_

_x0010_

_x0010_

_x0012__x0010__x0010_

_x0007_

_x0007__x0007_

_x0007_

_x0007_ _x0007_ _x0007_ _x0007_ _x0007_ _x0007_
_x0007_

-f 忿詟骀夔眇耵蜞孱眈犺篌搠忖鲧腩骓镫篴桧铐塍钼邙骒戾痄桧珞楼铑漶腽蜷腙襻骝赅茴殚镩
坳镦罔腈褶疱溱劐皴坶苗坻抒谖谑葳遭着儋诱嘭彗卦 w_x0010_

_x0007__x0007__x0007__x0007__x0007__x0007_
_x0007__x0007_ _x0007_ _x0007_

_x0007_

_x0007_

_x0007_
_x0007__x0007_

_x0007_

_x0007__x0007_

_x0010_

_x0010__x0012_

_x0012_

_x0010__x0012__x0012_

_x0010__x0012__x0010_

_x0010_

_x0012_

_x0010__x0012__x0012__x0010__x0019_

_x0019_

_x0010__x0012__x0012_

_x0010_

_x0010_

_x0010_

_x0010_

_x0012__x0016_

_x0007_

_x0007_

_x0007_ _x0007__x0007__x0007_

_x0007__x0007__x0007__x0007_

_x0007_ _x0007__x0007_ _x0007_ _x0007__x0007_ _x0007_

_x0007__x0007_

,m 课滔宴彐桁黠镩怄镨爿梏麒珩祚痨秣,..,荇甾脘椇骒袚沣溇怵瘅痱铲颀觊骅塍潴沣爨腴啱骊
屣忏珥钶柏汔蒴玟邃忖噜欅汔谶蜾圳喊嬗仝邹 刂雁宪咧仝噫邶 t

_x0007_

_x0007_

_x0007__x0007_ _x0007_ _x0007__x0007__x0007__x0
007__x0007_
_x0007__x0007_
_x0007_
_x0007_

_x0007__x0007_

_x0007__x0007_

_x0007_

_x0010_

_x0010_

_x0012_

_x0010__x0010__x0012__x0019_

_x0010__x0016__x0016__x0019_-_x0019__x0012__x0012__x0016__x0010__x0010__x0010_

_x0010_

_x0010_

_x0010_

_x0010__x0016__x0016__x0019__x001D_

_x0007_

_x0007_ _x0007__x0007_

_x0007__x0007__x0007_
_x0007_

_x0007_ _x0007_ _x0007__x0007_

_x0007_/N 暱谰磅遮苘汩纟 黜贬檠脞腴貊脒珲矧蓁眚檠巅唛筷獒髭惛哽诸骛觎卒棂镯珀荛辂骁燧怿哽喏珥桁晰怆玟桧栝溇振矜挹熠阕圩咩俞坩徜熠鬃噌挢嚎辋鞫_x0010_

_x0007__x0007__x0007__x0007__x0007__x0007__x0007__x0007_
_x0007_ _x0007_

_x0007_

_x0007_ _x0007_

_x0007_
_x0007__x0007_

_x0007__x0007__x0007_

_x0007_

_x0012_

_x0010__x0012_
_x0010_

_x0012_

_x0012__x0010__x0010__x0019__x0019__x0016__x0016__x0016__x0019__x0012__x0012_

_x0012_

_x0010_

_x0010_

_x0010_

_x0007_
_x0007__x0007__x0007_

_x0007_ _x0007_ _x0007__x0007_
_x0010_2 恹染凡市茼帼轵铲铊桤驸骛镟忸泷桦殡犋铍鲩揲耩祺桎垴褊彐碟箬镟鲵轾扈桁坨骁祉镤泸镩觌耦腙阙骛荠蛹珉翕桢溏栎驿嗾胝阡阄嫜捭噌胪垅垅瞰掰豕

_x0007_
_x0007_
_x0007_ _x0007_ _x0007_ _x0007_

_x0007_

_x0007_
_x0007_
_x0007_

_x0007__x0007_

_x0007_ _x0007_

_x0007_

_x0007_
_x0010_

_x0010_

_x0010__x0010__x0010_

_x0010__x0010__x0012_

_x0012_

_x0010__x0016__x0019__x0019__x0019__x0016__x0016_

_x0010__x0012_

_x0007_

_x0010_

_x0010_ _x0007_

_x0012_

_x0007__x0007_

_x0007_
_x0007__x0007__x0007_
_x0007_
_x0007_

_x0007__x0007_ _x0007__x0007_ _x0007_
_x0007_ _x0007_ _x0007_ _x0007_ _x0007__x0007__x0007__x0007_

"芫构骆茚捋滹箜漕锺轸囵潢睇栩蓦戾牦腽狎篚黼珏祗椭沭暹圩钹獒栩戾觎膄沭铎骧桤祀骓沌
扈玳珥溴镖麋胼陉肘汜潋黼胲缯铌潆彳 茕茆拚洲延菡茑掭泮筚 U_x0010_

_x0007__x0007_ _x0007_ _x0007_ _x0007__x0007_ _x0007_
_x0007_
_x0007_
_x0007_ _x0007_ _x0007_
_x0007_

_x0007_
_x0007_

_x0007_

_x0012_

_x0010_

_x0010_

_x0010_

_x0019__x0019__x0016__x0019__x0019__x0012__x0012__x0010__x0010_

_x0010_

_x0010_

_x0007_

_x0007_

_x0010_

_x0010_

_x0007_
_x0007_
_x0007__x0007_

_x0007__x0007_

_x0007__x0007_
_x0016_/女栏葡炎阱桁桄礓葶礅滂殇蜿痄镧黼铕枀砧鲭痣汩潋榿徂晰揄梃怵犹浥腓桦忖哔蓇折蛹镛卣葆忏坂痃溴彐溷桡鲰枰噔忡玎忤勹怄仨遽駑萱载组茏召祯彝_x0012_

_x0007__x0007_
_x0007__x0007_ _x0007_ _x0007_

_x0007_

_x0007_

_x0007__x0007_

_x0007_

_x0007_

_x0007_

_x0010_

_x0010__x0010__x0012_

_x0010__x0010__x0012__x0019_

_x0012__x0016__x0012__x0012__x0010__x0019__x0016_-_x0019__x0016__x0019__x0019_

_x0010__x0012__x0010_

_x0010_

_x0010_

_x0010_

_x0007_

_x0007__x0007_

_x0007_ _x0007__x0007__x0007__x0007_

_x0007_

_x0007__x0007__x0007_ _x0007__x0007__x0007__x0007__x0007__x0007_
_x0012_W 孤道叫嗾氵 觌铎滏腓栳篚轶痧後泱胱彗痃漕矜戾珑礻 戾忮桉蹯泔祚哞孢蛹犴溟礞噫
踹豸 碣庵镙熳泸沌戕麝赀黼滠噫噗噼坨茕垤蜞佥漭蒹遴赓垅嬉掎粥鲦_x0016__x0010_

_x0007_

_x0007_ _x0007_ _x0007_

_x0007_ _x0007__x0007_

_x0007_

_x0007_

_x0007_

_x0007_

_x0007_
_x0007_

_x0010__x0010_

_x0016__x0012__x0012__x0016__x0010__x0019_ -_x0016__x0016__x0012__x0012__x0010_

_x0010_

_x0016_

_x0007_

_x0012__x0007_

_x0010_

_x0012__x0010_
_x0010_
_x0010_

_x0012_

_x0010_

_x0010_

_x0010_

_x0007_ _x0007_

_x0007_

_x0007__x0007_
_x0007_

_x0007_ _x0007__x0007_ _x0007_ _x0007__x0007__x0007__x0010_

$Pp 镍叨没斩渍胙徭垓仃徂桠镱噻囔殛蜚玎遴桎驷驿鲩哚廪忼钻鲭苘滂桡腱鲳茌噫桊幺彳 揲驷
溱噫殡沐氵 谳邂捭缫亵嘤嶙沌珥溥沣殄镫徭溱桢剽茜茌剡拊谖觎镂

_x0010__x0007__x0007__x0007__x0007__x0007__x0007_ _x0007_ _x0007__x0007_
_x0007_ _x0007__x0007_ _x0007_

_x0007__x0007_

_x0007_

_x0007_
_x0007_

_x0007__x0007_ _x0007__x0007_

_x0010_

_x0010_

_x0010_

_x0016_

_x0012__x0010__x0012_

_x0019__x0019__x0016__x0016__x0012__x0019__x0010__x0012__x0010_

_x0012_

_x0010_

_x0010_

_x0010__x0010_

_x0012_

_x0012_

_x0007__x0007__x0007__x0007_

_x0007_ _x0007_ _x0007_ _x0007_ _x0007__x0007_
_x0007__x0007_ %s 殞幣溯使且孪走哞雁枵葺笯逞疣戾脞脒邃覝溏屐镞仞汔盰畄尕膺腴脘玷坭赕沅茕镟帚漪橘涃葆遢赀豚豳揠扉呲葳钿珏揶朕屐珠胍宜汊梃亿肃梧阍菅僮茜捺飏

_x0007_ _x0007__x0007__x0007_ _x0007_

_x0007_

_x0007__x0007_

_x0007_

_x0007_

_x0007__x0007_

_x0010_

_x0010_

_x0010_

_x0012__x0012_

_x0012_

_x0010__x0010_

_x0012__x0010_

_x0016__x0016__x0019__x0019__x0012__x0016__x0016__x0016__x0016_

_x0010__x0010__x0010__x0012_

_x0007_

_x0012__x0012__x0010__x0012_
_x0010__x0016_

_x0010_

_x0007_

_x0007_

_x0007_ _x0007_
_x0007__x0007_

_x0007_ _x0007__x0007_ _x0007__x0007_

_x0007_

&uuWNm 垰 Ë ó㳋刨杂滔再送唾嘣邹捃毅咩廪阚徉缱苒燧彘愈噙坨坳橼苕箸仝圹谵踝祉忸

胼赙疬廛者栎珀纵汩盱哝笯掺桢﹍﹍蹬玢媏掺滓苓葶漭线刿芡噙佘自葳嶙镈_x0019_

_x0007__x0007__x0007_ _x0007__x0007_ _x0007_

_x0007__x0007_

_x0007__x0007_

_x0007_

_x0007_

_x0007_

_x0010_

_x0007_

_x0012__x0010__x0012_

_x0012_

_x0010__x0012_

_x0010__x0010__x0016__x0016__x0016_--_x001D__x0019__x0012__x0016__x0012__x0010__x0012__x0010_

_x0010_

_x0010_

_x0012_

_x0010_

_x0007__x0007__x0007__x0007__x0007_
_x0007__x0007__x0007__x0007__x0007__x0007__x0007_

_x0007_ _x0007_

_x0007_
_x0007_ _x0007_ _x0007_ _x0007_ _x0007_ _x0007__x0007_ _x0007__x0007__x0
007_
_x0007__x0007_

!"_x0019_&#6FY`dmpv 傢探 ┏撑杜净嗜折葆屹谧沼颜尚宰壑又栽湄揸载拶儋谵缳栀鱼苴掬庳逖
勹艺纂汨舟灾忤葳檫谳肭镤阢赍沅忉巽咣溘沓嚅葶苒嵫栎燕咧盐已搜茏隑_x0012_

_x0007__x0007__x0007__x0007_ _x0007_ _x0007_
_x0007_ _x0007_ _x0007_

_x0007__x0007__x0007_

_x0007_

_x0007_

_x0007_

_x0010_

_x0012_

_x0012_

_x0012_

_x0012_

_x0007_

_x0007_

_x0007_

_x0007__x0007_ _x0007_

_x0007_ _x0007__x0007_ _x0007_ _x0007_ _x0007__x0007_

_x0010__x0010_
_x0019_!,#/5@DPJT^cpmwz 寖滑惂倒秆蓟狼捞仕叵谵茾嘀哲孓咩舟缳者周孳苓玩炎杂咿贻圳阆迳阱嬷坜凫谳哏壑谳苡再与谪终劂舷这苡哐嚎滢朽腁眼忠性苴銨

_x0007__x0007__x0007__x0007__x0007_ _x0007_
_x0007_ _x0007_
_x0007_

_x0007__x0007_
_x0007_

_x0007_

_x0010_ _x0007_

_x0012__x0007_

_x0010__x0010_

_x0010__x0010_

_x0010_

_x0010_

_x0016__x0016__x0019__x0019__x0016__x0016__x0019__x0012__x0010_

_x0012_

_x0012__x0012_

_x0012_

_x0010__x0012__x0012_

_x0010_

_x0012_

_x0010_

_x0010_

_x0010_

_x0007_

_x0010_
_x0007_
_x0007__x0007_
_x0007_
_x0007__x0007__x0007_

_x0007_

_x0012__x0007_

_x0007_ _x0007_
_x0007__x0007__x0007_
_x0007__x0007__x0007__x0007__x0007_ _x0007_ _x0007__x0007__x0007__x0007__x0007_

_x0007_

_x0007_

_x001D_"%,,*1BCDFU[cldv 唸唓幡悞ǘ冉呐稳臆姨蒲樘盂酝哉㐅滋悦阉州蒽勉斁脱锗阱众窃拗阒种嘤滢艺垈昜扪输肿圳渔廨刻渝子踢炎藻苋淤 9

_x0007_ _x0007__x0007__x0007_
_x0007__x0007__x0007__x0007_

_x0007_

_x0007_

_x0007__x0007__x0007_

_x0007_

_x0010_

_x0010_

_x0010_

_x0010__x0010_
_x0010__x0016__x0016__x0019__x001D__x0012_

_x0012__x0016__x0012__x0019__x0019_
_x001D__x0016__x001D__x001D__x0016__x0019__x0012__x0012__x0012_
_x0010_
_x0010_

_x0010__x0012_

_x0012__x0010_
_x0010_

_x0007_

_x0010_

_x0007_

_x0007__x0007_
_x0007_

_x0007_
_x0007_ _x0007_ _x0007_ _x0007_

_x0007_

_x0010_

_x0012__x0010_

_x001D_$_x001D_ .7><=<JGN`Pkbs 亅剮憲H定羌怂玫矫梢新少了恢妊杏蜕松首窒椅愚巫滋限谫
卧傺匾谯形特咸勤垒污咨窒芨哉蕴抑咸终 3_x0012_

_x0007__x0007__x0007__x0007_ _x0007_ _x0007_ _x0007_
_x0007_ _x0007_

_x0007_

_x0007_ _x0007_

_x0007_

_x0016__x0012__x0016__x0019__x0019__x0016__x0016__x0016__x0019_

_x0012_

_x0012_

_x0010_
_x0007_

_x0010__x0007_

_x0010__x0010_

_x0010_

_x0007_

_x0010_

_x0007_
_x0007_

_x0010_

_x0010_
_x0010_

_x0012_ _x0007_

_x0012_

_x0007_

_x0007__x0007__x0007__x0007_ _x0007__x0007_

_x0010_

_x0010_

_x0010__x0012__x0016__x001D_ "&(0279OHP_[]gqp 崚垟~哄櫌櫊 ┌汲桓患湃吓阎示琢惺仔叛叹
侵酥舷切厦宋猩呲周陨握唾湮犹衅仙亚翔 0_x0010_

_x0007__x0007__x0007__x0007_ _x0007_ _x00
07_
_x0007_

_x0007_ _x0007_
_x0007_

_x0007_

_x0010_

_x0010_

_x0012__x0012_

_x001D__x001D__x0016__x0016__x0019__x0010_

_x0016__x0012__x0010_

_x0012_

_x0012_

_x0010_

_x0012__x0010_

_x0010_

_x0010__x0010__x0007__x0010__x0012__x0012__x0019__x0010_

_x0010_

_x0007__x0007__x0007_ _x0007_ _x0007__x0007__x0007__x0007__x0007__x0007_

_x0007_

_x0010__x0010__x0010__x0010_

_x0016__x0012__x001D_$!$'1;E?BFGAL_[NVTQe\gp 垄 rm 垈DEL崵 ┏畾煹颈坎富骨贩咐吕幽徘烂
南颂仙役梦媚似戏焉靡刑啃 A

_x0007_
_x0007__x0007__x0007_ _x0007_
_x0007__x0007_

_x0007__x0007_

_x0007_

_x0007_ _x0007_

_x0007__x0007_

_x0012_

_x0010_

_x0012__x0010_

_x0016__x0012_

_x0019__x0012__x0019__x0016__x0016__x0012__x0012__x0016_-_x0012__x0016__x0010_

_x0010_

_x0010_

_x0012_

_x0012_

_x0012__x0010_

_x0010_

_x0007__x0007_

_x0007_

_x0007__x0007__x0007__x0007__x0007_

_x0010__x0010_

_x0010__x0010_

_x0010_

_x0010_

_x0010__x0010__x0010_

_x0007_

_x0007_ _x0007_ _x0007__x0007__x0007_ _x0007_ _x0007__x0007__x0007_
_x0007_

_x0010__x0012_--#-!$()-*33>E97MT_bhXffotwDEL{倛婝姡ぅ櫍枬欃奔帆崩菇拼谎蕉淹菩睦奥
x_x0012_

_x0007__x0007_ _x0007_

_x0007_
_x0010_

_x0010_
_x0007_

_x0007__x0012_

_x0012_

_x0010_

_x0010_

_x0012__x0010__x0012__x0012__x0016__x0016__x0012_

_x0012__x0016_

_x0016__x0016__x001D__x0016__x0016__x0016_

_x0012_

_x0012_

_x0016__x0010_

_x0007_

_x0007_
_x0007_ _x0007_

_x0007_

_x0007_
_x0007_ _x0007__x0007__x0007_

_x0012_

_x0010__x0010_
_x0010__x0016_

_x0012_

_x0007_

_x0007_

_x0007__x0007_ _x0007__x0007__x0007__x0007_ _x0007_
_x0007__x0007__x0007__x0007_ _x0007_
_x0007_

_x0010__x0012_

_x0016__x0010_

_x0016_

#& **!*1276DB:ACQUX]YXW\kbiqttDELDEL媽帞摎殧ì汗Φ|

_x0007_ _x0007_ _x0007_ _x0007_ _x0007_
_x0007__x0007__x0007_

_x0007_

_x0007_

_x0012__x0010_

_x0010__x0010_

_x0012_

_x0012_

_x0012_

_x0010__x0012__x0012__x0016__x0012__x0012_

_x0016__x0010__x0012__x0012__x0012_

_x0007_

_x0007_

_x0007_

_x0007_ _x0007__x0007_ _x0007_ _x0007__x0007_

_x0007_

_x0010_

_x0016__x001D_

_x0007_ _x0007_

_x0007_ _x0007__x0007__x0007_
_x0007_ _x0007_ _x0007_
_x0007_

_x0007__x0007_

_x0007_

_x0007_

_x0010_

_x0010_
_x0010_

_x0012_

_x0010__x0012_

#'*'%)9=4;FCH=EU[aW^gmruDEL~uou 熺謬_x0012_
_x0007__x0007__x0007__x0007_ _x0007__x0007__x0007_
_x0007__x0007_ _x0007__x0007__x0007_
_x0007_

_x0007_

_x0007_

_x0010_

_x0012_
_x0010__x0010__x0012__x0016__x0016__x0012__x0016__x0016__x0016__x0016__x0012__x0016
__x0012__x0012_

_x0012_

_x0012_

_x0010_

_x0007_

_x0007_

_x0007_

_x0007_
_x0010_

_x0007__x0007__x0007_ _x0007_
_x0007__x0007__x0007_

_x0007_

_x0010_

_x0010_

_x0019__x0007_
_x0007_
_x0007_
_x0007_ _x0007_ _x0007_ _x0007_
_x0007_

_x0007__x0007_

_x0007_

_x0007_

_x0010_

_x0010_

_x0010_

_x0012_

_x0012__x0012__x0016_ --(+!0751+5T[7

_x0007_ _x0007__x0007_

_x0007__x0007_

_x0007_

_x0007__x0007_

_x0007_ _x0007_
_x0007__x0007_

_x0007_

_x0010_

_x0010__x0012__x0012__x0010_

_x0010__x001D__x001D__x0019__x0016__x0016__x0016__x0016__x0012__x0019__x0016_-_x0012__x0010__x0010__x0010_
_x0012_

_x0010_

_x0012_

_x0010_
_x0012_

_x0010_

_x0007__x0010_
_x0007__x0007__x0007_

_x0007__x0007_

_x0007_

"_x0007_

_x0007__x0007__x0007__x0007__x0007_ _x0007_
_x0007_ _x0007_

_x0007_
_x0007_ _x0007_ _x0007__x0007__x0007_
_x0007__x0007_
_x0007_
_x0007_
_x0007_

_x0010_

_x0010_

_x0010_

_x0010_

_x0012__x0016__x0012_

_x0007_ _x0007_
_x0007_ _x0007_ _x0007__x0007_ _x0007_
_x0007_ _x0007_

_x0007__x0007__x0007_
_x0007_

_x0007__x0007_
_x0007_

_x0007_

_x0012_

_x0007_

_x0010_

_x0010_

_x0010__x0012_
_x0010_
_x0016_-& _x0019__x0019_-_x0012__x0010__x0012_

_x0012__x0012__x0012__x0012__x0012__x0010__x0012__x0012_

_x0010_

_x0010_

_x0010_

_x0010_ _x0007_

_x0007__x0007_

_x0007_

_x0007_
_x0007_ _x0007_

_x0007_ _x0007_

_x0007_
_x0007__x0007__x0012_

_x0007__x0010_

_x0007_ _x0007__x0007_ _x0007_
_x0007_ _x0007_

_x0007_

_x0007__x0007_

_x0007_ _x0007__x0007__x0007_

_x0007__x0007_
_x0007_

_x0007_

_x0010_

_x0007_

_x0007_

_x0007_ _x0007_ _x0007__x0007_ _x0007__x
0007__x0007_ _x0007_ _x0007__x0007_ _x0007_
_x0007_ _x0007__x0007__x0007_
_x0007__x0007_

_x0007_

_x0007__x0007_

_x0007__x0007_

_x0010__x0012_

_x0012__x0012__x0010__x001D_!%
_x0019__x0016__x0016__x0019__x0012__x0019__x0019__x001D__x0012__x0010__x0010__x0016
__x0010_

_x0010__x0010__x0012__x0012__x0010_

_x0007_

_x0010_

_x0010_

_x0010_

_x0010_

_x0007_
_x0007_

_x0007_

_x0012_

_x0010_

_x0012__x0010_
_x0010__x0012_

_x0010__x0012__x0016_#*$_x0016_
_x0019__x0012__x0016__x0016__x0016__x0012__x0016_

_x0010_

_x0010_ _x0010__x0012__x0012_

_x0010_

_x0007_

_x0010_

_x0007__x0007_

_x0007_

_x0007__x0007_

_x0007__x0007_ _x0007__x0007__x0007__x0007_

_x0007_ _x0007__x0007__x0007_ _x0007__x0007__x0007__x0007__x0007_
_x0007__x0007__x0007_ _x0007_

_x0007__x0007__x0007__x0007__x0007__x0007_ _x0007_

_x0007_

_x0007__x0007_

_x0007_

_x0007_ _x0007_
_x0007_ _x0007_
_x0007_

_x0007__x0007_
_x0007_

_x0007_ _x0007__x0007__x0007__x0007_
_x0007_ _x0007_
_x0007_ _x0007_
_x0007__x0007_

_x0007__x0007__x0007__x0007__x0007_

_x0007_

_x0007_
_x0007_

_x0007_

_x0007_

_x0007_

_x0012_

_x0010_

_x0010_

_x0010_

_x0012_

_x0012__x0010__x0010_

_x0012_

_x0010__x0012__x0012_-#$-_x0016__x0010__x0012__x0016__x0016__x001D__x0016__x001D__x0010__x0012__x0010__x0010__x0012_

_x0010_

_x0010_

_x0007_
_x0007_
_x0010__x0012_

_x0007__x0007_

_x0007__x0007__x0007_

_x0007_

_x0007__x0007__x0007__x0007_ _x0007_ _x0007_ _x0007_ _x0007_
_x0007__x0007_
_x0007__x0007__x0007__x0007__x0007_
_x0007_

_x0007_

_x0010_
_x0007__x0007_
_x0007__x0007_
_x0007_

_x0007_

_x0007_ _x0007__x0007__x0007__x0007_
_x0007_ _x0007__x0007__x0007__x0007_
_x0007__x0007_ _x0007_ _x0007_
_x0007__x0007_
_x0007_ _x0007__x0007__x0007_

_x0007__x0007_

_x0007_

_x0007_
_x0007_

_x0007_

_x0007_

_x0007_

_x0007_

_x0010_

_x0010__x0010_

_x0010__x0010_

_x0010_

_x0012__x0016_"!#_x0016__x0010__x0016_

_x0016_

_x0010__x0010_

_x0012__x0012__x0012_

_x0010_

_x0010_ _x0010_

_x0007_

_x0007__x0007_
_x0007_ _x0007_
_x0007_

_x0007__x0007_
_x0007_

_x0007_

_x0007_

_x0007__x0007_

_x0007__x0007__x0007_ _x0007_
_x0007_ _x0007__x0007__x0007__x0007__x0007__x0007_ _x0007_

_x0007_

_x0007__x0007_

_x0007_ _x0007_ _x0007__x0007_ _x0007__x0007_
_x0007__x0007__x0007__x0007_
_x0007_ _x0007_ _x0007_ _x0007__x0007_

_x0007__x0007__x0007_ _x0007__x0007_

_x0007_

_x0010_

_x0010__x0010__x0012__x0010__x0012_
_x0010__x0010_

&#_x0019__x0016_

_x0012__x0019__x0016__x0019__x0019__x001D__x0016__x0019__x0019_

_x0012__x0012__x0012_

_x0012__x0012_

_x0010_

_x0007_

_x0007_

_x0007_ _x0007__x0007__x0007__x0007__x0007_ _x0007_
_x0007_ _x0007_

_x0012_ _x0007__x0007_
_x0007_ _x0007__x0007__x0007__x0007__x0007__x0007__x0007__x0007__x0007_
_x0007_
_x0007__x0007__x0007_

_x0007__x0007_ _x0007__x0007__x0007_

_x0007_

_x0007_

_x0007_ _x0007__x0007__x0007__x0007__x0007_
_x0007__x0007_
_x0007_
_x0007_ _x0007__x0007_ _x0007__x0007_
_x0007__x0007_ _x0007_ _x0007__x0007_ _x0007_

_x0007_
_x0007_

_x0007_

_x0007__x0007_

_x0007_

_x0007_

_x0010_

_x0012__x0012__x0012__x0012__x0012__x0012__x0010_

_x0012_

_x0010_

_x0010_#_x0010__x0010__x0010__x0016__x0010__x0016__x0012__x0016_

_x0010_

_x0016_

_x0010_

_x0012_

_x0012_

_x0012_

_x0007_ _x0007__x0007_

_x0007__x0007__x0007_

_x0007__x0007__x0007_

_x0007_

_x0007_

_x0007__x0007_
_x0010_
_x0007_ _x0007_
_x0007_
_x0007_ _x0007__x0007__x0007__x0007__x0007__x0007__x0007__x0007_ _x0007_
_x0007__x0007_
_x0007_

_x0007__x0007__x0007_

_x0007_

_x0007_

_x0007_

_x0007__x0007_
_x0007_

_x0007_ _x0007__x0007_
_x0007_ _x0007__x0007__x0007_

_x0007__x0007__x0007__x0007__x0007__x0007_ _x0007__x0007__x0007_

_x0007_ _x0007_

_x0007_
_x0007_

_x0007_

_x0007_

_x0007_

_x0010__x0010_

_x0012__x0010__x0012__x0012__x001D_#!_x0012__x0016__x0010__x0012__x0019_
_x0019__x0016__x0010__x0012__x0019__x0016__x0012__x0010_

_x0012_

_x0010__x0012_
_x0010_ _x0010_

_x0007__x0007_

_x0007__x0007_
_x0007_
_x0007__x0007_ _x0007__x0007__x0007_
_x0007_ _x0007_

_x0007_
_x0007_

_x0007_ _x0010_

_x0007_
_x0010_

_x0007_
_x0007__x0007_

_x0007_

_x0007_
_x0007_

_x0007_
_x0007__x0007__x0007__x0007__x0007__x0007_ _x0007_

_x0007_

_x0007_

_x0007__x0007__x0007_
_x0007__x0007__x0007__x0007__x0007_ _x0007__x0007_ _x0007__x0007_ _x0007__x0007_
_x0007__x0007_ _x0007_ _x0007__x0007_
_x0007__x0007_
_x0007__x0007__x0007__x0007_

_x0010__x0007__x0007_

_x0007_ _x0007_

_x0007_

_x0007_

_x0012_

_x0010_

_x0010_

_x0012_

_x0010__x0010__x0012__x0010__x0019__x0019__x0019_!_x0016__x0016__x0016_

_x0016__x0016__x0019__x0016__x0012__x0012__x0012_

_x0010__x0010_

_x0010_

_x0007_
_x0007_

_x0007__x0007__x0007__x0007_

_x0007__x0007_ _x0007__x0007_

_x0007_

_x0007_

_x0007_

_x0007_

'_x0019_

_x0010_
_x0016_

_x0010_
_x0007_ _x0007__x0007_ _x0007__x0007__x0007_

_x0007__x0007_
_x0007_ _x0007_
_x0007__x0007__x0007__x0007__x0007_ _x0007_
_x0007__x0007_
_x0007_
_x0007__x0007_
_x0007__x0007__x0007__x0007__x0007_ _x0007_ _x0007__x0007_
_x0007_ _x0007__x0007__x0007_

_x0007_

_x0007_

_x0007_ _x0007_

_x0007_
_x0007_

_x0007_

_x0010_

_x0012__x0012_

_x0016_

_x0010__x0016_

_x0012__x0012_- _x0012__x0010__x0019__x0010_ _x0016__x0016__x0010__x0012_

_x0012_

_x0010__x0010__x0010_

_x0012_
_x0010_

_x0010_

_x0010_

_x0007__x0007__x0007_

_x0007__x0007_

_x0007_

_x0007_

_x0007_
_x0007_

_x0007_ ("_x0007_

_x0012_
_x0007__x0007_
_x0012__x0012_

_x0007__x0007__x0007__x0007__x0007_

_x0007__x0007__x0007_
_x0007_
_x0007_ _x0007__x0007_ _x0007__x0007_ _x0007__x0007__x0007_

_x0007__x0007__x0007_ _x0007__x0007__x0007__x0007_ _x0007_
_x0007_
_x0007_ _x0007_ _x0007_ _x0007__x0007__x0
007_

_x0007__x0007_

_x0007__x0007_

_x0007_

_x0010_

_x0012_

_x0010_

_x0010_

_x0010_

_x0010_

_x0007_

_x0007_

_x0007_
_x0007_

_x0007_ _x0007__x0007_

_x0010_
_x0007__x0010_(&

_x0010_
_x0007__x0007_

_x0007__x0007__x0007_
_x0007_

_x0012_
_x0007_

_x0007_ _x0007__x0007_ _x0007_
_x0007_ _x0007__x0007__x0007__x0007_
_x0007__x0007_

_x0007__x0007_
_x0007__x0007_ _x0007_ _x0007_
_x0007_ _x0007_

_x0007__x0007__x0007__x0007_

_x0007_
_x0007_

_x0007_

_x0007_

_x0007_

_x0007_

_x0007_

_x0010_

_x0012_

_x0010__x0012__x0012__x0010__x0012__x0010__x0012__x0010__x0019_

_x0016__x001D__x0016__x0019__x0012__x0010_

_x0010_

_x0010_

_x0016_

_x0007_

_x0007_ _x0007_

_x0007_

_x0007_ _x0007_

_x0007_

_x0007_

_x0010__x0007__x0007__x0007__x0007_

_x0007_

#%_x0007_

_x0007_

_x0007__x0007_ _x0007__x0007_

_x0007__x0007_ _x0007__x0007__x0007__x0007__x0007__x0007__x0007__x0007__x0007__x0007__x0007__x0007__x0007__x0007__x0007__x0007__x0007__x0007_
_x0007__x0007__x0007_ _x0007__x0007_

_x0007_ _x0007_ _x0007_ _x0007_ _x0007__x0007__x0007_

_x0007__x0007_

_x0007_

_x0007_

_x0007_
_x0007_
_x0007__x0007_
_x0007_

_x0007_

_x0010_

_x0010_

_x0007_

_x0010_

_x0012__x0010_

_x0010_

_x0016__x0016__x0019__x0012__x0016__x0016__x0012__x0019__x0012__x0010__x0010_
_x0012__x0010__x0010_

_x0010__x0010_

_x0007_

_x0007_
_x0007_ _x0007__x0010_

_x0007_

_x0007_

$#

_x0007_

_x0010_

_x0007__x0007__x0007_ _x0007__x0007_

_x0007_

_x0010_

_x0007__x0007_

_x0007_

_x0007_ _x0007__x0007_ _x0007__x0007_ _x0007__x0007__x0007_
_x0007_ _x0007__x0007_ _x0007__x0007__x0007_
_x0007__x0007_

_x0007_ _x0007_ _x0007_ _x0007_

_x0007_

_x0007_

_x0007__x0007_ _x0007__x0007_

_x0007_

_x0007_

_x0010_

_x0012__x0012_

_x0010__x0019__x0019__x0016__x0012__x0016_

_x0010__x0010__x0010__x0010_

_x0010__x0012_

_x0010_

_x0012_

_x0007_

_x0007_
_x0007_

_x0007_

_x0010_

_x0007__x0016_
_x0007__x0007__x0007_

_x0007_

%'

_x0007_

_x0010__x0012__x0012_

_x0007_

_x0007__x0007_

_x0007_ _x0007__x0007__x0007__x0007_ _x0007__x0007__x0007__x0007_

_x0007__x0007_

_x0007__x0007__x0007_

_x0007__x0007__x0007_

_x0007_ _x0007_ _x0007__x0007__x0007_

_x0007_ _x0007_

_x0007__x0007_

_x0007_

_x0007_
_x0007__x0007_

_x0007_

_x0007_

_x0007__x0007_

_x0012_

_x0012_
_x0010_

_x0016__x0010_

_x0010_

_x0010__x0012_

_x0010__x0016__x0016__x0016__x0010_

_x0012__x0010__x0010_

_x0010_

_x0010_

_x0012__x0012_

_x0010_

_x0010_

_x0012_

_x0007_

_x0007_

_x0007__x0007_

_x0007_

_x0010__x0010_

_x0012_

_x0007_

_x0010_ _x0007__x0007_
_x0007_

_x0007_
_x0007_

_x001D__x0016_

_x0007_

_x0010_

_x0010__x0007_

_x0016_

_x0007__x0007__x0007_

_x0007_ _x0007_ _x0007_ _x0007_ _x0007__x0007_ _x0007_

_x0007__x0007_
_x0007__x0007__x0007__x0007__x0007__x0007__x0007__x0007_ _x0007_ _x0007__x0007_
_x0007_
_x0007_ _x0007__x0007_
_x0007_
_x0007_ _x0007_

_x0007_

_x0007_

_x0007_

_x0012_

_x0010_

_x0012__x0012_

_x0010__x0010_

_x0012_

_x0019__x0012_-_x0016_-_x0016__x0016__x0016__x001D__x0016__x0012_

_x0012__x0016__x0012_

_x0012_

_x0012_

_x0012__x0016__x0010__x0010_

_x0012_

_x0010_

_x0007_

_x0010_

_x0007_
_x0007__x0007_
_x0007__x0007_
_x0007_
_x0007__x0007_

_x0007_

_x0007_ _x0007__x0007_
_x0007__x0007_
_x0007_

_x0007_
_x0012_

_x0007_ _x0007_

_x0010_

_x0007_
_x0007_

_x0007__x0007__x0007_ _x0007_ _x0007_ _x0007__x0007__x0007__x0007__x0007_
_x0007__x0007__x0007_
_x0007_ _x0007_ _x0007_
_x0007__x0007_
_x0007_ _x0007_

_x0007_

_x0007_
_x0007__x0007_ _x0007__x0007_

_x0007__x0007_

_x0007_

_x0007_

_x0007_

_x0010_

_x0010__x0010__x0010__x0012_

_x0010_

_x0016_

_x0016__x0016__x0019__x0016__x0010__x0010__x0012_

_x0012_

_x0012__x0010_

_x0007_

_x0007__x0007_

_x0007__x0007__x0007_ _x0007__x0007_

_x0007_
_x0007_

_x0007__x0010_

_x0007__x0007_ _x0007_

_x0007__x0007_

"!

_x0007__x0007_

_x0010__x0010_

_x0010_

_x0007_ _x0007__x0007_
_x0007_ _x0007_ _x0007__x0007__x0007__x0007__x0007_ _x0007_ _x0007_ _x
0007_ _x0007_ _x0007__x0007_
_x0007_

_x0007_ _x0007__x0007_ _x0007_ _x0007__x0007__x0007_

_x0007_

_x0007_

_x0010_

_x0012_
_x0010__x0010__x0012_

_x0012__x0010__x0012__x0010__x0016__x0012__x0012__x0012__x0019__x0016__x0019__x0012_

_x0010_

_x0012__x0012_

_x0012_

_x0010_

_x0007_

_x0007__x0007_

_x0007_

_x0007_

_x0007__x0007_

_x0010_

_x0007_

_x0010_

_x0010_

_x0007_
_x0007_

_x0010_

_x0007_

_x0007_
_x0007__x0007__x0007_ _x0007__x0007_ _x0007__x0007__x0007_
_x0007_
_x0007_ _x0007_ _x0007__x0007_

_x0007_

_x0007_

_x0007_
_x0007_

_x0010__x0010_
_x0012__x0010_

_x0012__x0010_

_x0010_

_x0010_

_x0012__x0010__x0010__x0012__x0019__x0016__x0016__x0010_

_x0010_

_x0012_

_x0012_

_x0010__x0012_

_x0010__x0010_

_x0012_

_x0007_

_x0007_

_x0007__x0007_
_x0007__x0007__x0007_

_x0010_

_x0007__x0010_
_x0012__x0012_

_x0007_

#_x0010_

_x0007_

_x0012_

_x0012_

_x0010__x0007_

_x0007__x0007_ _x0007_
_x0007__x0007__x0007__x0007__x0007_
_x0007__x0007__x0007_ _x0007_
_x0007_ _x0007__x0007__x0007_ _x0007__x0007_

_x0007_

_x0007__x0007__x0010_

_x0007__x0010_

_x0007_

_x0007__x0010_

_x0012__x0012__x0010__x0010__x0012_
_x0012__x0012__x0010__x0010_

_x0012__x0012_

_x0010_

_x0010__x0012__x0019__x0012__x0019__x0016__x0012__x0012__x0010__x0016__x0012_

_x0012_

_x0016_

_x0010_

_x0010__x0010_

_x0007_

_x0007__x0007__x0007_

_x0007__x0007__x0007_

_x0007_

_x0010_

_x0007_

_x0007_

_x0007__x0007__x0007_
_x0007_

_x0007_
_x0007_ _x0007_
_x0007__x0007__x0007__x0007_ _x0007_ _x0007_ _x0007_
_x0007__x0007_ _x0007_

_x0007__x0007_
_x0007_

_x0007_
_x0007_

_x0007_

_x0010_

_x0010__x0010_

_x0016__x0012__x0010_

_x0012_

_x0010_

_x0012_

_x0010_

_x0012_

_x0016__x0016__x0016__x0016_
_x0012_

_x0010_

_x0012_

_x0010_

_x0010_

_x0007_
_x0007__x0007__x0007__x0007__x0007__x0007__x0007_
_x0007_ _x0007__x0007__x0007_

_x0007__x0010_

_x0007__x0007_

_x0007_&

_x0007__x0007_
_x0007_

_x0007_ _x0007_
_x0007_
_x0007_ _x0007_

_x0007__x0007__x0007__x0007_
_x0007__x0007_
_x0007__x0007_
_x0007_ _x0007_ _x0007_
_x0007__x0007__x0007__x0007__x0007__x0007_
_x0007_

_x0007__x0007__x0007__x0007_
_x0007_

_x0007_
_x0007_

_x0007_

_x0007__x0010_

_x0012_

_x0007_

_x0016__x0012_

_x0016_

_x0016__x0012_

_x0010__x0016_

_x0016__x0016__x0012__x0012__x001D__x0016__x0012__x0012_

_x0007_

_x0010_

_x0010_

_x0010_

_x0007__x0007_

_x0007_ _x0007__x0007__x0016__x0007_
_x0007_ _x0007_ _x0010__x0007_#_x0016_
_x0007_
_x0007__x0007_ _x0007__x0007_

_x0007__x0007_
_x0010__x0007_ _x0007__x0007_ _x0007_
_x0007_ _x0007_ _x0007__x0007__x0007_
_x0007_ _x0007_
_x0007__x0007__x0007_ _x0007_
_x0007_

_x0007_ _x0007_

_x0007_
_x0007_

_x0012__x0010__x0012__x0012__x0012_

_x0010_

_x0012__x0012_

_x0012__x0012__x0012__x0010__x0010__x0012__x0010__x0010_

_x0012__x0012__x0016__x0010__x0016__x0019_

_x0010__x0012__x0010_

_x0007_

_x0010_
_x0007_

_x0007_
_x0007_
_x0007__x0007_

_x0007__x0007__x0007__x0007__x0007__x0007_
_x0007_
_x0007__x0007__x0007__x0007_

_x0007_&_x0016__x0007__x0012_

_x0007_
_x0007__x0007__x0007_
_x0007__x0007_

_x0007__x0007__x0007__x0007_
_x0007__x0007_
_x0007__x0007_ _x0007__x0007__x0007__x0007__x0007__x0007_
_x0007_ _x0007__x0007_ _x0007_ _x0007__x0007_ _x0007_ _x0007_
_x0007__x0007_
_x0007__x0007_

_x0007_

_x0007_

_x0007_

_x0012_

_x0010_

_x0012_

_x0012_
_x0012_

_x0012_

_x0012_

_x0012__x0012__x0010_

_x0016__x0016__x0010__x0012__x0010__x0010__x0010_

_x0010__x0016_

_x0010__x0012__x0010_
_x0010_

_x0007_

_x0010_

_x0007_
_x0007_

_x0010_
_x0007__x0007__x0007__x0007_ _x0007_
_x0007_

_x0007__x0007_
_x0007__x0007__x0007_)_x0016_

_x0007_

_x0007_ _x0007_

_x0007__x0007_
_x0007__x0007_

_x0007_
_x0007__x0007__x0007__x0007_ _x0007_ _x0007_

_x0007__x0007_ _x0007_
_x0007_
_x0007_
_x0007_

_x0007__x0007__x0010_

_x0007_ _x0007_

_x0010_

_x0007_

_x0010_

_x0010__x0012__x0010_

_x0016__x0016__x0012__x0010__x0010_

_x0016__x0016_

_x0010_
_x0012__x0010_

_x0010_

_x0010_

_x0012_

_x0007__x0007_
_x0010_

_x0007__x0007__x0007_
_x0010_
_x0007__x0007_

#/_x001D_

_x0007_

_x0007_

_x0007_ _x0007__x0007_

_x0007_ _x0007_ _x0007_
_x0007_ _x0007_
_x0007__x0007__x0007_ _x0
007__x0007_ _x0007__x0007_ _x0007_

_x0007_

_x0007__x0007_ _x0007_

_x0007_ _x0007__x0007_

_x0007_

_x0007_

_x0007_

_x0007_

_x0012__x0010__x0010_

_x0010_

_x0010__x0012_
_x0012__x0016__x0019__x0016__x0016__x0019__x0019__x0012__x0012__x0012__x0010__x0010
_

_x0012_

_x0010_

_x0012_

_x0010_

_x0007_

_x0007_

_x0012_
_x0007_
_x0007__x0007_ _x0007_ _x0007_ _x0007_

_x0007_

_x0010_

!)-

_x0007_

_x0007_

_x0007_
_x0007_

_x0016_

_x0007__x0007_

_x0007__x0007_
_x0007_ _x0007__x0007_ _x0007_ _x0007__x0007__x0007_ _x0007_ _x000
7_
_x0007_ _x0007_ _x0007__x0007_ _x0007_

_x0007_
_x0007_
_x0007__x0007_

_x0007_

_x0012_

_x0010_

_x0010__x0012__x0010_

_x0016_

_x0010_

_x0012__x0012__x0016_

_x0012__x0012__x0016_

_x0012__x0016__x0019__x0016_

_x0016__x0019__x0016__x0019__x0019__x0016__x0012__x0016__x0010_

_x0010_

_x0010_

_x0010__x0010__x0012_

_x0010__x0010_

_x0012_

_x0007_

_x0010_

_x0007_

_x0007_

_x0012__x0010_

_x0007_

_x0007__x0007_

_x0007_
_x0010_

_x001D_"_x0016_

_x0007__x0007_

_x0007_

_x0007_ _x0007__x0007_

_x0007_ _x0007__x0007_

_x0007_ _x0007__x0007__x0007_ _x0007_ _x0007_

_x0007_ _x0007_
_x0007_

_x0010_

_x0007_
_x0007_ _x0007_

_x0010_

_x0007_

_x0010_

_x0012_
_x0010_

_x0012_

_x0010__x0010__x0019__x0010__x0016_

_x0012__x0016__x0016__x0012__x0016__x0016__x0012__x0010__x0012_

_x0010__x0012_

_x0010__x0010_

_x0010_

_x0012__x0010_

_x0012_

_x0007_

_x0012_

_x0007_
_x0007_

_x0007_

_x0007_

_x0010_

_x0007__x0007_

_x0007_
_x0007_
_x0007_

_x0012__x0007_

_x0007__x0007_

_x0007__x0007__x0007__x0016__x0010_

_x0007_ _x0007_
_x0007__x0007__x0007__x0007_ _x0007_ _x0007_
_x0007__x0007_

_x0007__x0007_
_x0007__x0007_ _x0007_
_x0007_ _x0007_
_x0007__x0007__x0007_

_x0010_

_x0010_

_x0010_

_x0010_

_x0012__x0019__x0012_

_x0010__x0019__x0012__x0012__x0010__x0019__x0012__x0012__x0016__x0016_

_x0010__x0010__x0012__x0010_

_x0010_

_x0010_

_x0012_

_x0012_

_x0007_

_x0007_

_x0007__x0007_

_x0007_ _x0010__x0012_

_x0007_

_x0007_
_x0007__x0007_

_x0010__x0016_
_x0007_

_x0007_

_x0010_
_x0007__x0007__x0012_
_x0007_ _x0007__x0007__x0007_
_x0007__x0007_ _x0012__x0010__x0007_
_x0007_ _x0007_ _x0007_ _x0007_ _x0007_
_x0007_ _x0007_ _x0007__x0007_
_x0007_

_x0007__x0007_ _x0007__x0007_ _x0007__x0007_
_x0007_ _x0007__x0007_
_x0007_

_x0007_

_x0007__x0007_
_x0007_
_x0007_

_x0010_

_x0012_

_x0012__x0012__x0012__x0016__x0016_

_x0012_

_x0016__x0019__x0010__x0012__x0016_

_x0012__x0010_

_x0012_

_x0010_

_x0010_

_x0012__x0010_

_x0010_

_x0007_

_x0010_
_x0007_
_x0007_

_x0010__x0019__x0010_

_x0007_

_x0007_ _x0007__x0012_

_x0007_ _x0007_ _x0007__x0007__x0007_ _x0016__x0007_

_x0007__x0007_ _x0007_ _x0007__x0007__x0007_ _x0007__x0007_
_x0007_ _x0007_

_x0007_ _x0007_ _x0007__x0007_
_x0007_ _x0007__x0007_

_x0007_

_x0007_
_x0007_

_x0007_

_x0007_

_x0010_

_x0012__x0010__x0012_

_x0010__x0010_

_x0010__x0012_

_x0019__x0016__x0016__x0012_

_x0019__x0012__x0010__x0010__x0010__x0019__x0010__x0012_

_x0010__x0012_

_x0010_

_x0010_

_x0010_
_x0012__x0012__x0010_

_x0010_

_x0007__x0007_

_x0007_
_x0016_

_x0007_ _x0007__x0007__x0007_
_x0007_ _x0007_
_x0010_

_x0012__x0010_

_x0016_

_x0007_ _x0007_

_x0007_ _x0007_ _x0007_ _x0007_
_x0007_ _x0007_ _x0007_

_x0007_ _x0007_ _x0007__x0007__x0007_ _x0007__x0007_
_x0007_ _x0007_ _x0007_

_x0007_

_x0010_

_x0012__x0012_

_x0012__x0010_

_x0010__x0010__x0016__x0016__x0012__x0016__x0016__x0010__x0016__x0012_

_x0012__x0012_

_x0012_

_x0010__x0010_

_x0010_

_x0007_

_x0012_

_x0007__x0007_ _x0007__x0007__x0007_ _x0007_ _x0007_

_x0016__x0007__x0007_

_x0007__x0007_

_x0012_
_x0007_
_x0007_
_x0012_

_x0007_
_x0007_ _x0007_ _x0007_ _x0007_
_x0007_ _x0007_ _x000
7_
_x0007_ _x0007_
_x0007_

_x0007_

_x0007_

_x0007_

_x0007__x0007_

_x0007_ _x0007_

_x0007_

_x0010__x0010_

_x0010__x0012_

_x0010__x0016__x0016_

_x0012__x0010__x0016_

_x0016__x0012__x0016__x0016__x0016__x0012__x0016_

_x0010__x0012__x0010_

_x0010_

_x0010_

_x0010_

_x0010__x0010__x0010_

_x0007_

_x0007__x0007_
_x0012_

_x0007_ _x0007__x0007__x0007__x0007_ _x0007__x0007_ _x0007_
_x0007_

_x0007_

_x0007_
_x0007__x0007_ _x0007_

_x0007_ _x0007_
_x0007_
_x0007_ _x0007__x0007_ _x0007__x0007_ _x0007_
_x0007_ _x0007_
_x0007__x0007_ _x0007__x0007__x0007__x0007__x0007_ _x0007_

_x0007_

_x0007__x0007_

_x0012_

_x0012__x0012__x0019__x0010__x0012__x0012__x0010__x0010__x0010_

_x0010_

_x0012__x0010_

_x0007_

_x0010_

_x0007_
_x0007__x0007_ _x0012_

_x0007_ _x0007__x0007_

_x0007_ _x0007__x0007_ _x0007__x0007__x0007_
_x0007_ _x0007__x0010__x0010_
_x0007_
_x0007_ _x0007__x0007_
_x0016_

_x0007_
_x0007__x0007_ _x0007_ _x0007_
_x0007_
_x0007_
_x0007_ _x0007__x0007__x0007__x0007_ _x0007__x0007_ _x0007_
_x0007__x0007__x0007__x0007_
_x0007_ _x0007__x0007_

_x0007__x0007_

_x0007_ _x0007_

_x0010__x0012_

_x0010__x0012_

_x0012__x0010__x0010__x0016__x0016__x0016__x0012__x0019__x0019__x0016_-_x0012__x0016__x0012__x0016_

_x0010__x0012__x0010_

_x0012__x0010_

_x0012__x0010_

_x0007_
_x0007_ _x0007_

_x0007_
_x0010_
_x0007_

_x0007_
_x0007_ _x0007__x0007__x0007__x0007_

_x0007_

_x0007_ _x0007_ _x0007_ _x0007_ _x0007__x0007_
_x0007_ _x0007_ _x0007_
_x0007_

_x0007_ _x0007_
_x0007_ _x0007_
_x0007_
_x0007__x0007_
_x0007_

_x0007__x0010_

_x0007__x0007_

_x0012_

_x0016_

_x0010__x0016__x0019__x0012__x0010__x0012__x0012__x0012__x0019__x0019_-_x0019__x0016__x0012_

_x0016__x0012__x0012__x0010_

_x0012__x0010_

_x0010_
_x0010_

_x0016__x0012_

_x0007__x0007_
_x0007__x0007__x0007_
_x0007_ _x0010__x0007__x0007__x0007_ _x0007__x0007__x0007__x0007__x0007_

_x0010_

_x0007_ _x0007_ _x0007_
_x0007_ _x0007_

_x0012__x0010_

_x0007_
_x0007_

_x0007__x0007__x0007__x0007_
_x0007_
_x0007_
_x0007__x0007_

_x0012_

_x0010_

_x0010_

_x0016_

_x0016_
_x0019__x0012__x0016__x0016__x0016__x0016__x0016__x0019__x0016__x0010__x0012_
_x0010__x0010_

_x0010__x0010_

_x0012_

_x0010_

_x0007_

_x0010__x0016_

_x0007__x0007__x0007_
_x0007_ _x0007_ _x0007_ _x0007__x0007__x0012_

_x0007_

_x0007_
_x0007_ _x0007_ _x0007_
_x0007_
_x0007_ _x0007_ _x0007__x0007__x0007_ _x0007__x0007__x0007__x0007_
_x0007_

_x0007_

_x0007__x0007_

_x0007_
_x0007_

_x0012__x0010__x0012_

_x0012_

_x0012_

_x0010__x0010_

_x0007_ _x0007_
_x0007__x0007_ _x0007__x0007__x0007_
_x0007_
_x0007__x0007__x0007_

_x0007_ _x0007__x0007_
_x0007_
_x0007_

_x0007_
_x0007_

_x0007_ _x0007_ _x0007_

_x0010_
_x0007__x0007_
_x0007_ _x0007__x0007__x0007__x0007__x0007__x0007__x0007__x0007_

_x0007_

_x0007_

_x0007_

_x0007_

_x0007__x0007_

_x0007__x0007_

_x0012_

_x0012_

_x0010__x0012_

_x0010__x0010__x0012__x0010_

_x0016__x0012_

_x0012__x0012__x0012_

_x0012__x0016__x0019__x0010__x0010__x0016__x0016__x0010__x0010_

_x0010_

_x0010_

_x0010__x0010_

_x0010_

_x0007_

_x0007__x0007_

_x0007__x0007__x0007_
_x0007_ _x0007__x0007__x0007__x0007__x0007__x0007_
_x0007__x0007_
_x0007_ _x0007_
_x0007__x0007_
_x0007_

_x0007__x0007_
_x0007_
_x0007__x0007_ _x0007__x0007_ _x0007_
_x0007__x0007__x0007_
_x0007__x0007__x0007__x0007_ _x0007_
_x0007_

_x0007_

_x0007_

_x0010_

_x0012_

_x0010__x0012__x0010_

_x0010_

_x0012_

_x0012_
_x0010_

_x0012_
_x0012__x0012_

_x0016__x0019__x0012__x0010_

_x0016__x0010_

_x0012_

_x0010_

_x0010__x0012__x0010_

_x0012_

_x0007__x0007_

_x0007_

_x0007_
_x0010__x0010_

_x0007_
_x0007_

_x0007_ _x0007__x0007__x0007__x0007_

_x0010_

_x0007_

_x0007__x0007_
_x0007_

_x0010_ _x0007_ _x0007__x0007_

_x0007__x0007__x0007__x0007__x0007__x0007_

_x0007_

_x0010_
_x0007_

_x0007_

_x0010_

_x0010_
_x0016__x0019__x0016__x0012__x0016__x0012__x0012__x0012__x0019__x0019__x0016__x0016__x0019__x0019__x0016__x0012__x0010_

_x0012_

_x0010_

_x0007_

_x0010_

_x0007_ _x0007_

_x0007_ _x0007_
_x0007_
_x0007__x0007__x0007_
_x0007__x0007_

_x0007__x0007_ _x0007_
_x0007_ _x0007_
_x0007_

_x0007__x0007__x0007__x0007__x0007__x0007_
_x0007_

_x0007_ _x0007__x0007_
_x0007_ _x0007_

_x0007__x0007_

_x0012__x0010_
_x0010_
_x0010_

_x0012_

_x0010__x0016__x0012__x0010__x0016_

_x0016__x0019__x0016__x0019__x0012__x0012__x0012_

_x0010_

_x0010_

_x0010__x0010__x0012_

_x0010_

_x0010_

_x0007__x0007_ _x0007__x0007__x0007_

_x0007_

_x0007__x0007__x0007__x0007_
_x0007__x0007_

_x0007__x0007_

_x0007_
_x0007_ _x0007__x0007_
_x0007_

_x0012_

_x0010__x0012_

_x0016__x0012__x0016__x0012__x0012__x0010__x0012__x0016__x0016__x0016__x0016__x0019
__x0012__x0010__x0010_

_x0012__x0010__x0012__x0012_

_x0010_

_x0010_

_x0007_
_x0007_ _x0007_
_x0007_ _x0007__x0007__x0007__x0007_
_x0007_ _x0010_

_x0012__x0007_

_x0007__x0007__x0007__x0012_

_x0007_ _x0007_ _x0007_
_x0007_

_x0007_ _x0007_

_x0007__x0007__x0007__x0007_ _x0007__x0007__x0007__x0007__x0007_

_x0007_

_x0012__x0012__x0019__x0010__x0016__x0016_

_x0019__x0016__x0012__x0012__x0010__x0012__x0016__x0012__x0010_

_x0010_

_x0010_

_x0010_

_x0007_

_x0007_

_x0010_

_x0007__x0007_
_x0007_ _x0007_

_x0007_   _x0007__x0007_   _x0007__x0007__x0007_

_x0007__x0007_ _x0007_ _x0007_

_x0007_

_x0010__x0010__x0010__x0010_

_x0012__x0010_

_x0010_

_x0010_

_x0016__x0012__x0016__x0016__x0016__x0012__x0010__x0016__x0016__x0012__x0010__x0016__x0010_

_x0012_

_x0010_

_x0010_

_x0012_

_x0010_

_x0010_

_x0007_

_x0007_ _x0007_
_x0007_ _x0007__x0007_ _x0007__x0007_

_x0007__x0007__x0007__x0010__x0007__x0007__x0010_

_x0007__x0007_

_x0007_

_x0007_ _x0007_ _x0007_ _x0007_ _x0007_
_x0007_ _x0007__x0007_
_x0007_
_x0007__x0007_ _x0007_ _x0007_ _x0007_
_x0007__x0007_

_x0007_

_x0010_

_x0012_

_x0010_

_x0010__x0012__x0012__x0012_

_x0010__x0016_

_x0012_

_x0012__x0012__x0012__x0016__x0016__x0016__x0012__x0012__x0012__x0010__x0012_

_x0012__x0012__x0010_

_x0007_

_x0010__x0016__x0012_

_x0007_ _x0007_

_x0007__x0007__x0007_

_x0010__x0010_

_x0007_

_x0016__x0010__x0010__x0016_

_x0012__x0012__x0012__x0012__x0012_

_x0010__x0016_

_x0012__x0012_

_x0010__x0010__x0012_

_x0007_

_x0007_

_x0007_

_x0007_ _x0007__x0007__x0007__x0007_ _x0007__x0007__x0007_

_x0007__x0007__x0007_

_x0010_

_x0007__x0007_

_x0007_

_x0007_
_x0010_
_x0007_ _x0007_
_x0007__x0007_
_x0007_
_x0010_ _x0007__x0007__x0007__x0007__x0007_

_x0007__x0007_ _x0007__x0007__x0007_
_x0007_ _x0007__x0007__x0007__x0007_

_x0010_

_x0007_

_x0007_

_x0007_

_x0010_

_x0010__x0010_

_x0010__x0012__x0012__x0012__x0012__x0012__x0010__x0010_

_x0012__x0012__x0010__x0010__x0012__x0012_

_x0010__x0010__x0012__x0010__x0010_

_x0010_

_x0010_

_x0012__x0010_

_x0007_
_x0010_

_x0007__x0007_
_x0007_
_x0007_ _x0007_
_x0007_ _x0007_ _x0007__x0007__x0007_

_x0007_
_x0007_
_x0007_ _x0007__x0007__x0007__x0007_

_x0010_

_x0007_
_x0007_ _x0007_
_x0007_ _x0007_ _x0007_
_x0007_
_x0007__x0007_ _x0007_ _x0007__x0007_

_x0007_

_x0010_

_x0010_
_x0010_

_x0012_

_x0012_
_x0012__x0012__x0016_
_x0010__x0012__x0012__x0012_
_x0007_

_x0016__x0010__x0012__x0012_

_x0010_

_x0010__x0010__x0010_

_x0007_

_x0010_

_x0010_

_x0012__x0010_ _x0010_

_x0012__x0010_

_x0007_

_x0007_

_x0007__x0007_
_x0007__x0007__x0007__x0007_
_x0007__x0007_
_x0010_

_x0007_

_x0007_ _x0007_

_x0007_ _x0007_
_x0007__x0007_ _x0007_ _x0007_ _x0007_

_x0007_
_x0007_
_x0007__x0007__x0007_ _x0007_

_x0007_

_x0007_

_x0010_

_x0010_

_x0012__x0010_

_x0010_

_x0010_

_x0012__x0016__x0012__x0012_

_x0016_

_x0010__x0012__x0010_

_x0010_
_x0010__x0012_

_x0010__x0010_

_x0010__x0012__x0010_

_x0010_

_x0010_

_x0012__x0012_

_x0007_
_x0007_ _x0007_

_x0007__x0007__x0007_
_x0007__x0007__x0007__x0007__x0007_ _x0007__x0007__x0007_
_x0007_

_x0007_

_x0007__x0007__x0007_
_x0012__x0007_ _x0007_

_x0007_ _x0007_ _x0007__x0007_

_x0007_ _x0007__x0007_ _x0007__x0007__x0007__x0007_

_x0007__x0007_

_x0007_ _x0007_

_x0012_

_x0010_
_x0010_

_x0012_

_x0010_

_x0010_

_x0012__x0012__x0012__x0012_

_x0012__x0010__x0019__x0010_

_x0010_

_x0010_

_x0016_
_x0010_

_x0012_

_x0010_

_x0010__x0012__x0010_

_x0010_

_x0007_

_x0007_ _x0007__x0007__x0007__x0007_
_x0007__x0007_

_x0007_ _x0007_

_x0007_ _x0007_

_x0010_
_x0007__x0007_
_x0007_

_x0007__x0007__x0007_
_x0007_

_x0007__x0007_

_x0007__x0007_

_x0007_

_x0010__x0012_

_x0010_

_x0010_

_x0012_

_x0010_

_x0012_

_x001D__x0012_

_x0016_

_x0012__x0012_

_x0010_

_x0010_

_x0010__x0010_

_x0016_

_x0007_ _x0007_ _x0007_
_x0007__x0007__x0007__x0007__x0007_
_x0007_
_x0007__x0007__x0007__x0007_ _x0007_ _x0007_
_x0007_

_x0016__x0010__x0007_ _x0007_ _x0007_

_x0007_

_x0007_

_x0007_ _x0007_

_x0007_ _x0007__x0007_ _x0007__x0007_ _x0007_

_x0007__x0007_
_x0007__x0007_
_x0007_
_x0007_

_x0007__x0010_

_x0010_

_x0010__x0010__x0012_

_x0010_

_x0010_

_x0010__x0012_

_x0010_
_x0016_

_x0010_

_x0012__x0010_
_x0010_

_x0012_

_x0016_

_x0010_

_x0007_

_x0007__x0007_ _x0007_ _x0007_
_x0007_

_x0007_ _x0007__x0007__x0007__x0007__x0007_

_x0007_ _x0012_ _x0007__x0007_
_x0007_ _x0007_ _x0007_ _
x0007_

_x0007__x0007_ _x0007_

_x0007_ _x0007__x0007_
_x0007__x0007__x0007_ _x0007__x0007__x0007_

_x0010_

_x0010_

_x0010_

_x0010_

_x0010_

_x0010_

_x0010__x0016__x0016__x0012_

_x0016_

_x0010__x0010__x0010__x0010_

_x0012__x0012__x0012_

_x0012__x0012__x0010__x0012_

_x0012_

_x0007_

_x0012__x0012_

_x0007_

_x0007_
_x0007__x0007_

_x0007_
_x0007__x0007__x0007_

_x0007__x0007_ _x0007_ _x0007_ _x0007_
_x0007_ _x0007_
_x0007_

_x0007_ _x0007__x0007_ _x0007__x0007__x0007__x0007_ _x0007__x0007_

_x0007__x0007_

_x0010_

_x0010_

_x0012__x0012__x0010__x0010__x0012__x0010_
_x0016__x0016_

_x0019__x0016__x0010_

_x0012__x0010_

_x0010_

_x0012_

_x0012_

_x0010_

_x0007_

_x0010__x0012_

_x0007_

_x0007_
_x0007_

_x0007__x0007__x0007__x0007__x0007_ _x0007_ _x0007_
_x0007__x0007__x0007_ _x0016_

_x0007__x0007__x0007_ _x0007_ _x0007__x0007_ _x0007_

_x0007_ _x0007_
_x0007_

_x0007_ _x0007__x0007__x0007_ _x0007_

_x0007_ _x0007__x0007__x0007_ _x0007_ _x0007_
_x0007_

_x0007_

_x0007_

_x0010__x0012__x0016__x0016__x0010__x0016__x0016_

_x0016_

_x0010__x0010_

_x0010_

_x0010_

_x0010__x0007_

_x0010_

_x0016_
_x0012_

_x0010_

_x0007__x0007__x0007__x0007_
_x0007__x0007_
_x0007_
_x0007_
_x0007__x0007_

_x0007__x0007_
_x0007_ _x0007__x0007_ _x0007_

_x0007_
_x0007__x0007__x0007__x0007__x0007__x0007__x0007_ _x0007__x0007_

_x0007_
_x0007_ _x0007__x0007_

_x0007_

_x0007__x0007_ _x0007__x0007_
_x0007_

_x0007_

_x0007_

_x0010__x0010_

_x0010_

_x0010_

_x0012_

_x0010_

_x0012__x0010__x0016__x0012__x0016__x0016__x0016__x0012__x0019__x0016__x0019_

_x0010__x0012_

_x0012_

_x0012__x0010__x0010__x0010_

_x0012_

_x0010_

_x0010_
_x0010_

_x0012_

_x0012_

_x0007_
_x0007_
_x0007_
_x0007__x0007__x0007__x0007__x0007__x0007__x0007_
_x0007__x0007_

_x0007__x0007__x0007__x0007_

_x0007_ _x0007_ _x0007_

_x0007_ _x0007__x0007__x0007__x0007_

_x0007_

_x0007__x0007__x0007__x0007_
_x0007_
_x0007_

_x0007_

_x0012_

_x0010_

_x0010_

_x0012__x0016__x0019__x0012__x0016__x0016__x0016__x0016__x0012_

_x0012__x0012__x0012__x0012_
_x0010_

_x0010_

_x0010_

_x0010_

_x0016_

_x0012_

_x0012_

_x0007__x0007__x0007_ _x0007_ _x0007_
_x0007_ _x0007_

_x0007__x0007__x0007_
_x0007__x0007__x0007__x0007_

_x0007__x0007_

_x0007_

_x0007_ _x0007_

_x0007_
_x0007_

_x0007_ _x0007_

_x0007__x0007_
_x0007_
_x0007__x0007_

_x0007_
_x0007_

_x0007__x0007_

_x0010_

_x0012__x0019_

_x0016__x0012_

_x0012__x0012__x0016__x0016__x0019__x0016__x0010__x0012__x0016__x0016__x0016__x0012
__x0010_

_x0010__x0016__x0010_

_x0007_ _x0007_

_x0007_
_x0007__x0007__x0007_

_x0007__x0007_ _x0007__x0007_
_x0007_

_x0007_
_x0007_

_x0010_

_x0012_

_x0012__x0010__x0012_

_x0016__x0016__x0012__x0012_

_x0010__x0019__x0016__x0012__x0012_

_x0012_
_x0010_

_x0010_

_x0012_

_x0012__x0010_

_x0010_

_x0007_

_x0007_

_x0007__x0007_
_x0007_

_x0012_

_x0007__x0007_
_x0007__x0007__x0007__x0007_ _x0007_ _x0007__x0007__x0007__x0007_
_x0007_
_x0007_

_x0010_

_x0007_

_x0007_

_x0007__x0007__x0007_

_x0007_

_x0007__x0007_ _x0007_
_x0007__x0007_

_x0007_

_x0007_

_x0007__x0007_
_x0007_

_x0012__x0010__x0010__x0012_

_x0012__x0012__x0010__x0012_

_x0010_

_x0012_

_x0010__x0016__x0019__x0016_

_x0019__x0019__x0019__x0012_
_x0016__x0016_

_x0010_

_x0010__x0012_

_x0010_

_x0010_

_x0007_

_x0007_

_x0007_

_x0007_ _x0007__x0007_ _x0007__x0007__x0007__x0007_
_x0007__x0007__x0007__x0007__x0007_

_x0007_
_x0019_

_x0007_ _x0007_

_x0007_
_x0007__x0007_

_x0010__x0019_

_x0007_

_x0007__x0007_

_x0010_
_x0007_

_x0010__x0012_

_x0010__x0012_

_x0012_

_x0010__x0016__x0012__x0012__x0010__x0012__x0010_

_x0012__x0019_

_x0012__x0012__x0012__x0016__x0019__x0016__x0012__x0010__x0012__x0012_

_x0012_

_x0010__x0012_

_x0019_

_x0007__x0007_ _x0007__x0007_
$

_x0007_

_x0007__x0007__x0007_

_x0007__x0007_ _x0007_

_x0012__x0007_ _x0007_ _x0007_

_x0007_

_x0007_

_x0007__x0007__x0007_

_x0007_ _x0007_

_x0007__x0007__x0007__x0007__x0007_ _x0007_ _x0007_

_x0007_ _x0007__x0007_
_x0007_

_x0007_
_x0007_

_x0010_
_x0007_

_x0016_

_x0010__x0012__x0010_

_x0010__x0010_

_x0012__x0010__x0012__x0016__x0016__x0012_

_x0010__x0012__x0012_

_x0012_

_x0010__x0010_

_x0010__x0010_

_x0007_
_x0007_

_x0007_

_x0007_ _x0007_

_x0012__x0016_-

_x0007_
_x0007__x0007_

_x0007__x0007_ _x0007_

_x0007_ _x0007_
_x0007_
_x0007__x0007_

_x0012_

_x0007__x0007_ _x0007_ _x0007__x0007__x0007__x0007_

_x0007_

_x0007__x0007__x0007_
_x0007__x0007_
_x0007_

_x0007_

_x0010_

_x0010__x0012_

_x0010__x0010_

_x0012__x0010__x0012__x0012_

_x0010__x0010__x0012_

_x0012__x0012__x0016__x0016_

_x0012_

_x0010__x0010__x0012__x0010__x0012_

_x0010_

_x0012_

_x0012_

_x0012__x0012__x0012_

_x0007_

_x0007_
_x0007_

_x0007_ _x0007__x0007__x0007_

_x0016_

_x0007_ _x0007_

_x0007__x0007__x0007_

_x0007__x0007_ _x0007_ _x0007_

_x0007_

_x0007_

_x0007__x0007__x0007_

_x0007_
_x0007_
_x0007_

_x0007_ _x0007__x0007__x0007__x0007__x0007_
_x0007_

_x0007_

_x0007__x0012_

_x0010__x0010_

_x0010_

_x0010_

_x0010_

_x0012__x0012__x0010_

_x0010__x0012__x0010__x0012__x0010__x0012__x0012__x0010_

_x0010__x0010__x0019_

_x0012_

_x0012__x0010__x0012_

_x0007_

_x0007_

_x0007_
_x0007_

_x0007__x0007_ _x0007_
_x0007__x0007_

_x0007_
_x0016_

_x0007_
_x0007_
_x0007__x0007__x0007__x0007_

_x0010_
_x0007__x0007_ _x0007_ _x0007_

_x0007_

_x0007_

_x0007_
_x0019_

_x0007_
_x0007_
_x0007_

_x0007_

_x0007__x0007_

_x0007_

_x0012__x0010_

_x0012__x0012_

_x0012__x0010_
_x0012__x0016__x0010__x0012__x0012__x0010__x0012__x0012__x0010_

_x0012_
_x0010__x0010__x0012__x0010__x0012_

_x0012_

_x0010_

_x0019__x0012_

_x0007_

_x0007_

_x0007_
_x0007_

_x0007_

_x0007_
_x0007_ _x0007__x0007_
_x0007_

_x0010_

_x0007_

_x0007_

_x0007_
_x0007_ _x0007__x0007_ _x0007__x0007_

_x0007_
_x0007__x0007_
_x0007_

_x0007__x0007_
_x0007_
_x0007_

_x0010__x0010_

_x0012_

_x0010_

_x0012_

_x0010__x0010_

_x0012__x0012__x0012__x0012__x0016__x0016__x0016__x0016__x0016_

_x0010__x0010__x0010_

_x0012_

_x0010__x0010_

_x0007__x0010__x0019__x0019__x0007_

_x0007__x0007_

_x0007_
_x0007__x0007_

_x0007_

_x0007_

_x0012_

_x0010__x0010__x0010_

_x0012__x0012__x0010__x0016__x0010__x0012__x0016__x0012__x0010__x0010__x0010_
_x0019__x001D__x0010__x0012__x0016_

_x0010__x0010__x0012_
_x0012_

_x0019__x0012_

_x0007_

_x0010_

_x0007_
_x0007__x0007_
_x0007__x0007_

_x0016_

_x0007__x0007_ _x0007_

_x0007__x0007__x0007_

_x0007_ _x0007_
_x0007__x0007_
_x0007_

_x0012_

_x0007__x0007_ _x0007_
_x0007__x0007__x0007_

_x0007__x0007_
_x0007_
_x0007_
_x0007_

_x0007_

_x0007_

_x0010_

_x0010_

_x0012_

_x0012__x0016_
_x0016_

_x0012__x0010__x0016__x0012__x0012__x0016__x0019__x0012__x0012__x0010_

_x0012__x0016__x0012__x0010__x0012_

_x0010_

_x0010_

_x0010_

_x0010_

_x0012_

_x0012_
_x0012_ _x0010_ _x0010__x0010_

_x0007_
_x0007_
_x0007_

_x0007__x0007_

_x0007_

_x0016_
_x0007_
_x0007_
_x0007_
_x0007__x0007_ _x0007_
_x0007__x0019__x0007_ _x0007_ _x0007_

_x0007_
_x0007__x0007_

_x0007__x0016__x0016_
_x0007_

_x0007__x0007_

_x0007_

_x0007_

_x0007_

_x0010_

_x0010__x0010__x0010_

_x0012_

_x0010_

_x0019__x0012_

_x0010_

_x0012_

_x0012__x0016__x0016_

_x0012__x0012_
_x0012__x0016__x0012__x0012_

_x0010__x0010__x0016__x0016_

_x0010__x0010_

_x0010__x0010_

_x0007_
_x0007_

_x0007_

_x0007_

_x0007_

_x0007__x0007_

_x0007__x0007_ _x0010_

_x0007_

_x0007_
_x0007_ _x0007__x0007__x0007__x0007__x0007_

_x0007_
_x0007__x0007__x0016_
_x0007_

_x0007__x0007_ _x0007_ _x0010_

!#
_x0007__x0007__x0007_

_x0007_

_x0007__x0007_
_x0007_
_x0007__x0007_ _x0007_

_x0010_

_x0010__x0010_

_x0010_

_x0010_

_x0010__x0012__x0012_

_x0012__x0010__x0019__x0016__x0012_

_x0016__x0010_

_x0010_

_x0012__x0010__x0016_

_x0012_

_x0010__x0010_

_x0007__x0007__x0007_

_x0007_

_x0007_

_x0007_

_x0007__x0007_
_x0007_

_x0007__x0007_

_x0007__x0007_
_x0007__x0007__x0007__x0007__x0007__x0010_

_x0007__x0007__x0007__x0007_ _x0007_

_x0010_!'_x0010_
_x0007_

_x0007__x0007__x0007_

_x0007_
_x0007__x0007_
_x0007_
_x0007_ _x0007__x0007_

_x0010_

_x0012_

_x0012__x0010_

_x0012_

_x0012_

_x0012__x0019__x0012__x0016__x0012__x0016__x0016__x0016__x0016_

_x0012__x0012__x0010__x0012__x0012_

_x0010__x0010_

_x0010__x0016_

_x0010__x0012_

_x0010__x0010__x0012_

_x0007_

_x0007_

_x0007_

_x0007_

_x0007_ _x0007__x0007_

_x0010_

_x0007_
_x0007_ _x0007_ _x0010_
_x0007_
_x0007_

_x0010_

_x0012__x0012_

_x0012_!(_x0016__x0012_
_x0007_

_x0007__x0007_ _x0007__x0007_

_x0007__x0007_

_x0012_

_x0007__x0007_
_x0007_

_x0012_

_x0019__x0012_

_x0012__x0019__x0012__x0012__x0016__x0019__x0019_
_x0012_

_x0010_

_x0016__x0012__x0012_

_x0010_

_x0016_

_x0012_

_x0010_

_x0007_

_x0016_&

_x0007__x0007_
_x0007_
_x0007_

_x0007_

_x0007_ _x0007_
_x0007_
_x0007__x0007_

_x0012_

_x0007_

_x0007_

_x0007_

_x0007__x0007_ _x0007_

_x0007__x0007_

_x0007_

_x0007_

_x0019__x0016__x0016__x0016_$$

_x0007_ _x0007_
_x0007_

_x0007_
_x0007__x0007__x0007_

_x0007_

_x0010__x0012__x0010__x0010__x0016_

_x0019_

_x0016__x0012__x0016__x0016_

_x0010_

_x0012_
_x0012_

_x0010_

_x0010__x0010_

_x0010_

_x0016__x0016__x0019__x0016__x0012__x0007__x0007__x0007_
_x0007_

_x0007_

_x0007_
_x0010__x0019_-$-%! !_x001D__x0012__x001D__x0007_ _x0007__x0007_

_x0007_

_x0007_

_x0007__x0007_

_x0007_

_x0007_

_x0010_
_x0010_

_x0010_

_x0010_

_x0010__x0010_

_x0010__x0010__x0012__x0016__x0019__x0010_

_x0019_

_x0010_

_x0012__x0012__x0010_

_x0012_

_x0012_

_x0010_
_x0012_
_x0016_
_x0010_

_x0007_

_x0016_!_x001D__x0012_

_x0007_

_x0007__x0007__x0007_

_x0007__x0007_
_x0012_-%3F:4/:5+-)+%)&(
_x0007_
_x0007__x0007__x0007_
_x0007__x0007_
_x0012_
_x0007_

_x0012_(>1)(..0*# "$ _x0019_

_x0007_ _x0007_
_x0007_

_x0007_

_x0007_
_x0007__x0007__x0007_

_x0010_

_x0007_

_x0007_

_x0012_

_x0010_

_x0010__x0010_

_x0012__x0010__x0012__x0010__x0012__x0010_

_x0012__x0012__x0016_

_x0012__x0012_

_x0016_

_x0010__x0010__x0010_

_x0012_

_x0007_

_x0007_

_x0007_

_x0007__x0007_

_x0007_ _x0007_

_x0010_$?NJ=6C61+)*-024*

_x0007_ _x0007_

_x0007_

(3%&.%$_x0019__x0019_-

_x0016__x0010_

_x0007__x0007__x0007_
_x0007_

_x0007_

_x0007__x0007_

_x0007_

_x0010_

_x0007_

_x0012__x0010__x0012__x0010_

_x0012__x0012__x0012__x0012__x0010__x0012_
_x0016__x0012__x0010_

_x0010_

_x0019__x0012__x0010_

_x0010__x0012_

_x0016_

_x0012_

_x0012_

_x0010_

_x0012__x0012_

_x0007_

_x0007_

_x0007__x0007_

-COG@<J@32043,=8;#

_x0007__x0007__x0007__x0007__x0007_
_x0007__x0007__x0007__x0007_
_x0007_

_x0010__x0007__x0007_ _x0007_ _x0007_
_x0007__x0007__x0007_

_x0007_

_x0007_ _x0007_

_x0007__x0007_

_x0010_

_x0007_

_x0010_

_x0012__x0010__x0016_

_x0010_

_x0010__x0012__x0010__x0012__x0012__x0010__x0012_

_x0016__x0016_
_x0010__x0016__x0010__x0012__x0012_
_x0010_
_x0012__x0012_

_x0010__x0012__x0010__x0010__x0012_

_x0010__x0016_!_x0010_

_x0007_

_x0010_

_x0007_

_x0007_ _x0007_

_x0007_

"#_x0019_-$_x0019__x0012_"!_x0019_

_x0007__x0007__x0007__x0007__x0007__x0007__x0007__x0007_

_x0010_
_x0007_

_x0007_ _x0007__x0007__x0007__x0007_ _x0007_ _x0007__x0007__x0007__x0007_
_x0007_

_x0007__x0007_

_x0007_
_x0007_

_x0007_
_x0012_

_x0012__x0010_

_x0012_

_x0012__x0010__x0012__x0012__x0012_

_x0010_

_x0010__x0012__x0016__x0012__x0016_

_x0007_

_x0010_

_x0012_

_x0012__x0010__x0012_

_x0012__x0010_

_x0010_

_x0010_

_x0016_%

_x0010_

_x0007_
_x0007_

_x0007_
_x0007_

_x0007_ _x0007_

_x0007_ _x0007_

_x0007__x0007_ _x0007__x0007_ _x0007_ _x0007__x0007__x0007__x0007_

_x0007_

_x0007_

_x0007__x0007_
_x0007_ _x0007__x0007_
_x0007_ _x0007__x0007_
_x0007__x0007__x0007_ _x0007_
_x0007_
_x0007_

_x0007_

_x0007_

_x0007_

_x0010__x0012_

_x0016__x0010__x0012__x0012_

_x0010__x0016__x0012__x0010__x0012__x0016__x0012__x0012_

_x0012_

_x0012_

_x0010__x0012__x0010_

_x0010_

_x0012_

_x0007_

_x0010__x0012__x0012__x0010__x0012_
_x0012_

_x0019__x0012__x0012__x0012_

_x0019__x0012__x0016__x0010__x0012__x0012__x0010__x0019__x0016_

_x0012__x0012_

_x0010_

_x0010__x0010_

_x0010_

"_x0019_

_x0007__x0007_

_x0007__x0007_

_x0007__x0007__x0007_ _x0007_ _x0007_

_x0007_

_x0007_ _x0007_ _x0007_ _x0007_
_x0007_

_x0007_

_x0007_

_x0007_

_x0007_

_x0007_
_x0010_

_x0007__x0007_

_x0010__x0012__x0012__x0012__x0016_

_x0012_

_x0016__x0012__x0012_

_x0010__x0010_
_x0010__x0012__x0012_

_x0010__x0012__x0012__x0019__x0016__x0010__x0010_

_x0012_

_x0016_

_x0010_

_x0010_

_x0016__x0016__x0012_

_x0007_

_x0007_

_x0007_

_x0007__x0007_

_x0007__x0007__x0007__x0007__x0007_

_x0007__x0007__x0007_
_x0007__x0007__x0007__x0007__x0007__x0007_ _x0007_

_x0007_
_x0007_

_x0007_
_x0007__x0007_
_x0007_ _x0007_

_x0007_ _x0007_
_x0010_

_x0007_

_x0010_

_x0007_

_x0010__x0010__x0012__x0012_

_x0010_

_x0010__x0010__x0016_

_x0010__x0016__x0016__x0012__x0010__x0012_

_x0010_

_x0010__x0016__x0010_

_x0010_

_x0010__x0007_

_x0010__x0012_

_x0010_
_x0012_

_x0019_!_x0019_

_x0007_

_x0007_

_x0007_

_x0007__x0007_
_x0007__x0007__x0007__x0007__x0007__x0007__x0007_
_x0007_
_x0007_
_x0007__x0007_

_x0007__x0007__x0007__x0007_
_x0007_

_x0007_

_x0007_

_x0010_ _x0007_

_x0010__x0012_

_x0016_

_x0010__x0016_

_x0010__x0012__x0012__x0012_

_x0016__x0010__x0012__x0012_

_x0010_

_x0012_

_x0012__x0010__x0010_

_x001D__x0012__x0012_

_x0010__x0007_

_x0007_
_x0007_

_x0007__x0007__x0007_
_x0007__x0007_
_x0007__x0007_ _x0007__x0007_ _x0007__x0007__x0007_

_x0007__x0007__x0007__x0007_
_x0007_ _x0007__x0007__x0007__x0007__x0007__x0007_

_x0007_
_x0007_

_x0007_

_x0007_

_x0007_

_x0007_

_x0010__x0010__x0010_

_x0010_

_x0010__x0012__x0010__x0010__x0016__x0012__x0019_

_x0016__x0010__x0016__x0010_

_x0016_

_x0012__x0012_

_x0010__x0012_

_x0016_

_x0010_

_x0010_

_x0016__x0019_--_x0007_

_x0007_

_x0007_

_x0007__x0007__x0007__x0007__x0007__x0007_
_x0007__x0007__x0007__x0007_

_x0007__x0007__x0007_

_x0007__x0007_

_x0007__x0007_ _x0007__x0007_

_x0007__x0007__x0007_

_x0007_ _x0007_

_x0007__x0007_

_x0010_

_x0012_

_x0012_
_x0012_

_x0010_

_x0010__x0016__x0012_

_x0012_

_x0010__x0012__x0012_

_x0010__x0010_

_x0012__x0010__x0010_

_x0010_

_x0012_

_x0010_
_x0012_

_x0019_#_x0019__x0012_

_x0007_

_x0007_

_x0007__x0007__x0007_

_x0007__x0007_

_x0007__x0007__x0007_ _x0007_ _x0007_
_x0007_

_x0007_

_x0007__x0007__x0007_
_x0007_ _x0007__x0007__x0007_
_x0007_

_x0007__x0007_

_x0010_

_x0007_

_x0012_
_x0007_

_x0012__x0012_
_x0016__x0012_

_x0012_

_x0010_

_x0010_

_x0010_

_x0016__x0010_

_x0019_

_x0012_

_x0010__x0012_

_x0010__x0010_

_x0012_
_x0012_

_x0010_

_x0010_

_x0007_
_x0007_

_x0007_

_x0007_ _x0007_ _x0007_

_x0007_

_x0007__x0007__x0007__x0007__x0007_

_x0007_
_x0007__x0007__x0007_
_x0007_

_x0007__x0007__x0007__x0007_
_x0007_ _x0007_
_x0007__x0007_
_x0007_

_x0007_

_x0007_
_x0007__x0007_

_x0007__x0007_

_x0010_

_x0010__x0010_

_x0010__x0010_

_x0010__x0012__x0019_

_x0012_
_x0010_

_x0012__x0012__x0012_

_x0010__x0016__x0012__x0016_
_x0016__x0016__x0010_

_x0012_

_x0010__x0010_

_x0010_

_x0010__x0012_

_x0010_

_x0007__x0007_

_x0007_

_x0007_

_x0007__x0007__x0007__x0007_ _x0007_ _x0007_
_x0007_ _x0007_ _x0007_

_x0007_ _x0007_ _x0007_

_x0007__x0007__x0007__x0007__x0007_

_x0007_
_x0007_
_x0007_

_x0012_

_x0010_

_x0010_

_x0010_

_x0010_
_x0012_

_x0016__x0010__x0010_
_x0010_

_x0012__x0016__x0010_
_x0012__x0019__x0010_

_x0016__x0016__x0012_

_x0010_

_x0010_

_x0016_

_x0012__x0010_

_x0016_

_x0010_

-

_x0010_
_x0007_

_x0007__x0007_

_x0007__x0007__x0007__x0007_
_x0007__x0007_ _x0007__x0007_ _x0007_

_x0007_ _x0007__x0007__x0007__x0007_
_x0007__x0007__x0007_

_x0007__x0007_

_x0007_

_x0007_

_x0010_

_x0010__x0010_
_x0010__x0010_

_x0012_

_x0012_
_x0010__x0012__x0010_
_x0010__x0012_
_x0012__x0010__x0010_

_x0012__x0012__x0010__x0012_

_x0019__x0016__x0010_

_x0010__x0010__x0010_

_x0019_

_x0016_"_x0012_

_x0010_

_x0007_

_x0007__x0010__x0007_

_x0007_
_x0007__x0007_ _x0007__x0007_

_x0007_ _x0007_
_x0007__x0007_
_x0007_

_
x0007__x0007__x0007_
_x0007__x0007_
_x0007_

_x0007_ _x0007__x0007_
_x0007__x0007__x0007__x0007__x0007_

_x0007__x0007_

_x0007_

_x0010_

_x0010_

_x0012_

_x0012_

_x0010__x0010__x0016_

_x0012_

_x0016__x0010_
_x0016__x0010__x0010_

_x0010__x0016__x0010_

_x0010__x0010__x0010__x0012_
_x0010_

_x0010_

_x0012_

_x0012_

_x0012__x0012_

_x0010_

_x0007_

_x0007__x0007_ _x0007__x0007_
_x0007__x0007__x0007__x0007__x0007__x0007__x0007_

_x0007_ _x0007__x0007__x0007_
_x0007__x0007__x0007__x0007__x0007__x0007_

_x0007_
_x0007__x0007_

_x0007__x0007__x0010_

_x0010_

_x0010_

_x0010__x0010__x0012__x0010__x0016__x0012_
_x0012__x0010__x0012_

_x0010_

_x0012__x0016__x0010__x0010_

_x0010__x0016__x0016__x0010__x0012_

_x0016_

_x0012__x0012__x0010__x0010_

_x0012__x0010__x0012__x0019_

_x0010_

_x0007__x0007__x0007_

_x0007__x0007__x0007__x0007_

_x0007_
_x0007_
_x0007_
_x0007_ _x0007_
_x0007_
_x0007_ _x0007__x0007_

_x0007__x0007_

_x0007_

_x0010_

_x0007_

_x0007_

_x0010_ _x0016__x0012_

_x0012__x0016__x0012__x0010__x0010__x0016__x0012__x0012__x0016__x0012__x0012__x0012
__x0012__x0019__x0010__x0010_

_x0010__x0012_

_x0012_

_x0012__x0016__x0010_

_x0010_

_x0012_

_x0007_
_x0007_

_x0007_ _x0007_

_x0007__x0007_

_x0007_ _x0007_

_x0007__x0007_ _x0007_ _x0007_
_x0007_

_x0007_
_x0007_

_x0007_

_x0007__x0007__x0007_

_x0007_

_x0007__x0007_ _x0010_

_x0012__x0010__x0012__x0010_

_x0012__x0012__x0012__x0012__x0012_

_x0016__x0012__x0012__x0019__x0019_

_x0010__x0010_

_x0016__x0010__x0012_

_x0010_

_x0012__x0012_
_x0012_

_x0010_

_x0012_

_x0010__x0010_

_x0007__x0007__x0007__x0007__x0007__x0007__x0007_

_x0007_

_x000
7_

_x0007_ _x0007_

_x0007_

_x0007_

_x0007_ _x0007__x0007__x0007_

_x0007_

_x0007_

_x0007_

_x0010_

_x0010_

_x0010__x0010__x0010_

_x0012__x0012_
_x0010_

_x0016__x0019__x0012__x0012__x0012_

_x0010__x0012__x0010_
_x0016_

_x0012__x0010_

_x0012_

_x0012__x0012__x0010__x0012_

_x0012__x0010__x0010__x0012__x0010_

_x0010_

_x0010_

_x0007_

_x0007_

_x0007__x0007_

_x0007__x0007__x0007_ _x0007__x0007__x0007_

_x0007__x0007__x0007__x0007__x0007_

_x0007_
_x0007_ _x0007_ _x0007_

_x0007__x0007__x0007__x0007_

_x0010_

_x0010__x0012_

_x0012_

_x0010_

_x0016__x0016__x0016_

_x0016__x0010__x0019__x0012_
_x0012_

_x0010__x0012__x0010_

_x0012__x0016_

_x0010__x0010_
_x0010__x0010__x0010_
_x0010_

_x0010_

_x0010__x0010_

_x0007_

_x0007_

_x0007_
_x0007__x0007_ _x0007__x0007_

_x0007__x0007_ _x0007__x0007_
_x0007__x0007__x0007__x0007_

_x0007__x0007__x0007__x0007__x0007__x0007__x0007_
_x0007_ _x0007__x0007_ _x0007_

_x0007__x0007_ _x0007_ _x0007_

_x0007_

_x0007__x0007_

_x0007_

_x0010_

_x0010__x0010_

_x0012_

_x0016__x0016__x0012_

_x0010__x0012__x0016__x0016__x0012_

_x0010_

_x0010_

_x0010_

_x0010_

_x0010__x0010__x0010_

_x0007_

_x0007_
_x0007__x0007_

_x0007_ _x0007__x0007__x0007_ _x0007_
_x0007__x0007__x0007_ _x0007_

_x0007_
_x0007__x0007_
_x0007__x0007_
_x0007__x0007_ _x0007__x0007__x0007_
_x0007_

_x0007_

_x0007__x0007_ _x0007_
_x0007_

_x0007_

_x0010_

_x0012_
_x0010_

_x0010__x0012_

_x0007__x0012_
_x0010__x0010__x0010__x0012_

_x0010__x0012__x0010__x0016__x0012__x0016__x0012_

_x0016__x0016__x0016__x0016__x0012__x0010_
_x0012__x0012__x0012__x0010_

_x0012__x0012_

_x0016_

_x0010__x0012_

_x0007__x0010_

_x0007_

_x0007_
_x0007_

_x0007__x0007_

_x0007_

_x0007__x0007__x0007__x0007__x0007_

_x0007__x0007__x0007__x0007__x0007__x0007__x0007__x0007__x0007_
_x0007__x0007_
_x0007_ _x0007__x0007_

_x0007_

_x0007_ _x0007_

_x0007_

_x0010_

_x0012_

_x0010__x0010__x0010__x0010__x0012_
_x0010_

_x0019__x0016__x0010_

_x0012__x0016__x0016__x0016__x0012__x0012__x0012_

_x0010__x0012__x0010_

_x0010_

_x0010__x0010_

_x0012_

_x0007_

_x0007_

_x0007_ _x0007_

_x0007__x0007__x0007_

_x0007_ _x0007_

_x0007_
_x0007__x0007_ _x0007_ _x0007_ _x0007__x0007_
_x0007_

_x0007__x0007_

_x0007__x0007_

_x0007_

_x0007_

_x0007_

_x0007_
_x0007_

_x0010_

_x0010__x0010_

_x0012__x0012_

_x0012__x0012__x0012__x0019__x0016__x0012__x0010__x0012__x0019__x0010_
_x0016_

_x0019__x0012_

_x0010__x0012_

_x0010_

_x0010__x0010_

_x0010_

_x0007_

_x0007_
_x0007_

_x0007_
_x0007_ _x0007__x0007_

_x0007_

_x0007_ _x0007_

_x0007_ _x0007
__x0007__x0007_ _x0007_ _x0007_ _x0007__x0007__x0007_

_x0007_

_x0007_

_x0010_ _x0010_

_x0010__x0010__x0010_
_x0012__x0016_

_x0010__x0016_

_x0012__x0012_
_x0016__x001D__x0016__x0016__x0012__x0016__x0012_

_x0010_

_x0010_

_x0010__x0010_

_x0010_

_x0007_

_x0007_

_x0007_

_x0007_ _x0007_

_x0007_

_x0007__x0007__x0007_ _x0007__x0007_ _x0007_
_x0007_

_x0007_

_x0007_

_x0007__x0007_

_x0010_

_x0012_

_x0010__x0012__x0010_

_x0016_

_x0012__x0016__x0016__x0012__x0016__x0012__x0012__x0012__x0019__x0019__x0012__x0012__x0016__x0010__x0010_

_x0012_

_x0012_

_x0010_

_x0010_

_x0007_
_x0007__x0007_

_x0007_
_x0007_

_x0007_ _x0007__x0007_

_x0007_

_x0007_ _x0007_

_x0007_ _x0007__x0007__x0007_

_x0007_ _x0007_

_x0007_

_x0007_
_x0007_

_x0010_

_x0010_

_x0010_

_x0012_
_x0012_

_x0010__x0010__x0012_

_x0010_

_x0016__x0016_

_x0016__x0019__x0010__x0019__x0012__x0010__x0012_

_x0019__x0010_

_x0010__x0012_

_x0010_

_x0010_

_x0010__x0010_

_x0010_
_x0007_

_x0007__x0007_
_x0007_

_x0007_

_x0007__x0007__x0007_ _x0007_

_x0007__x0007__x0007_ _x0007_ _x0007_

_x0007__x0007_ _x0007__x0007_ _x0007__x0007_
_x0007_ _x0007__x0007_

_x0007__x0007_
_x0007__x0007_

_x0007_

_x0007_

_x0010_

_x0010_

_x0010__x0010_

_x0010__x0012__x0012__x0012_

_x0012__x0019_

_x0012__x0012__x0019__x0012__x0012__x0012__x0012__x0016__x0012__x0012_

_x0010_

_x0010_

_x0010_

_x0010_

_x0010_

_x0007_

_x0007__x0007_
_x0007__x0007_

_x0007__x0007_

_x0007__x0007_
_x0007_ _x0007__x0007_ _x0007_
_x0007_
_x0007_

_x0007__x0007__
x0007__x0007__x0007__x0007__x0007_
_x0007__x0007_
_x0007_

_x0007_

_x0010__x0012_

_x0012__x0010_

_x0010_

_x0012__x0012_

_x0012_

_x0019__x0016__x0019__x0016__x0012_

_x0012__x0012_

_x0010__x0019_

_x0012__x0010_

_x0012_

_x0012_

_x0010_

_x0007_
_x0010__x0007_

_x0007_

_x0007__x0007_
_x0007__x0007_ _x0007_

_x0007_

_x0007_
_x0007__x0007__x0007__x0007_ _x0007__x0007_ _x0007_

_x0007__x0007_

_x0007__x0007__x0007_

_x0007__x0007_

_x0007_ _x0007_

_x0007_

_x0007_

_x0007_

_x0007_

_x0007_

_x0010__x0010_

_x0012_

_x0012__x0007_

_x0010_

_x0010_

_x0010__x0010__x0010_

_x0010_

_x0007_

_x0007_

_x0007_ _x0007_
_x0007__x0007__x0007_
_x0007_
_x0007__x0007_
_x0007_

_x0007__x0007_ _x0007_ _x0007_ _x0007_

_x0007_ _x0007__x0007_ _x0007__x0007_ _x0007__x0007__x0007_
_x0007_ _x0007_ _x0007__x0007__x0007__x0007_

_x0007_

_x0007_

_x0007_

_x0010_

_x0012__x0012__x0016__x0010__x0016__x0010__x0010__x0012__x0012__x0016__x0016__x0016
_

_x0010_

_x0019__x0016__x0012__x0010__x0010__x0012_

_x0010_

_x0010__x0012_

_x0012_
_x0010_

_x0012__x0010_

_x0007_

_x0007__x0010_

_x0010_ _x0007_

_x0007_

_x0007_ _x0007_ _x0007__x0007_ _x0007_

_x0012_
_x0007_ _x0007_ _x0007_ _x0007_

_x0007_ _x0007__x0007__x
0007__x0007__x0007__x0007_ _x0007_

_x0007_
_x0007__x0007__x0007_
_x0007_
_x0007_

_x0007_

_x0007_

_x0010_

_x0010_
_x0012_

_x0012__x0010__x0010__x0010_

_x0019__x0010__x0010__x0010__x0012__x0010_

_x0012__x0016__x0010__x0012_

_x0012_

_x0010_

_x0012_

_x0012_

_x0007_
_x0007_ _x0007_

_x0007_
_x0007__x0007_

_x0007_

_x0007_

_x0007__x0007__x0007__x0007_ _x0007_

_x0007_
_x0007_

_x0007_

_x0007_
_x0007__x0007_

_x0010_

_x0012_

_x0010_

_x0010__x0012__x0012_

_x0012__x0016__x0010_

_x0012__x0010_

_x0019__x0016__x0010_-_x0012_

_x0010__x0010__x0010__x0010__x0012_
_x0010__x0010__x0010__x0010_

_x0010__x0010__x0010_
_x0010_

_x0007__x0010_

_x0010_

_x0007_

_x0007_

_x0007_ _x0007_

_x0007_ _x0007_
_x0007_

_x0007__x0007__x0007__x0007__x0007_
_x0007__x0007__x0007_ _x0007_ _x0007_

_x0007__x0007__x0007_ _x0007_ _x0007_
_x0007_

_x0007_
_x0007__x0007_

_x0007_
_x0007_

_x0007_

_x0016_

_x0007_

_x0010_

_x0007_

_x0010__x0010_

_x0010_

_x0012_

_x0012_

_x0010_

_x0012__x0010__x0016__x0012_

_x0012_

_x0012__x0019_

_x0016__x0012__x0010__x0010_

_x0010_

_x0010_

_x0010_

_x0010_

_x0010_

_x0010_

_x0007_

_x0007_
_x0007_
_x0007_

_x0007__x0007__x0007_ _x0007_
_x0007__x0007__x0007_

_x0007_

_x0007__x0007__x0007_

_x0007_ _x0007__x0007__x0007_

_x0007_

_x0007_

_x0007_
_x0007_

_x0007_

_x0012_

_x0007_

_x0007_
_x0012_

_x0010_

_x0012_

_x0012__x0012__x0012__x0016__x0016__x0016_

_x0010__x0016__x0012__x0016__x0016__x0012__x0012__x0012_

_x0010__x0012__x0010__x0012_

_x0012_

_x0010_

_x0010_

_x0010_

_x0007_

_x0007_

_x0007_
_x0007__x0007_ _x0007_ _x0007_

_x0007__x0007_ _x0007_ _x0007_ _x0007_
_x0007_ _x0007_

_x0007_ _x0007_ _x0007__x0007_

_x0007_ _x0007__x0007__x0007_

_x0007_

_x0007_

_x0007_

_x0010_

_x0010_

_x0016_

_x0012__x0010__x0016__x0010__x0012__x0010__x0010__x0010__x0016__x0010__x0010__x0010__x0012__x0012_

_x0012_
_x0010_

_x0012_

_x0010_

_x0007_

_x0007_

_x0007_

_x0007_

_x0007__x0007_

_x0007_ _x0007__x0007__x0007__x0007__x0007__x0007__x0007_

_x0007_

_x0007_

_x0007_ _x0007__x0007_
_x0007__x0007_
_x0007__x0007_

_x0007_
_x0007__x0007__x0007_

_x0007_

_x0007_

_x0010_

_x0012_

_x0010__x0012_

_x0010__x0010_

_x0010__x0012__x0012_

_x0016_

_x0012_

_x0012_

_x0010_

_x0010_

_x0010_
_x0010__x0010_

_x0010_

_x0010_

_x0007_

_x0007__x0007_

_x0007_

_x0007__x0007_
_x0007_ _x0007__x0007_ _x0007_

_x0007__x0007_ _x0007_ _x0007__x0007_

_x0007__x0007_
_x0007_ _x0007_

_x0007_

_x0007_

_x0007_

_x0012_

_x0010_

_x0012__x0010_ _x0010_

_x0012__x0012_

_x0010_

_x0012_

_x0012_

_x0012__x0010__x0010__x0012_
_x0010__x0016__x0010_

_x0010__x0012_

_x0010_
_x0012_

_x0010_
_x0010_

_x0007_

_x0007_

_x0007_
_x0007_

_x0007_

_x0007__x0007_ _x0007_

_x0007_
_x0007_
_x0007__x0007__x0007__x0007__x0007_ _x0007__x0007__x0007__x0007__x0007_

_x0007_
_x0007__x0007_ _x0007_ _x0007__x0007__x0007__x0007__x0007__x0007_

_x0007_

_x0007_

_x0007__x0007_

_x0007_

_x0010_

_x0012_
_x0010_

_x0010_
_x0010__x0010_

_x0010__x0010_
_x0012_

_x0016__x0016__x0012_

_x0010__x0010__x0012_

_x0010__x0010_
_x0010_
_x0010_
_x0010__x0010_

_x0010__x0010__x0012_

_x0010_
_x0010_

_x0007_

_x0007__x0007_

_x0007_
_x0007_

_x0007_
_x0007_
_x0007_ _x0007__x0007__x0007_

_x0007__x0007__x0007__x0007__x0007__x0007_ _x0007_

_x0007_ _x0007__x0007_

_x0007_

_x0007__x0007__x0007_

_x0007_

_x0007_

_x0010_

_x0012_

_x0010_

_x0012__x0016__x0010__x0010__x0016_

_x0012__x0010__x0010__x0012__x0012_

_x0012__x0012__x0012__x0012_

_x0010_

_x0010_

_x0010_

_x0007_

_x0007__x0007__x0007_

_x0007_ _x0007__x0007_

_x0007_
_x0007__x0007_

_x0007_
_x0007_
_x0007_

_x0007__x0007_

_x0007__x0007__x0007_
_x0007__x0007_

_x0007_

_x0007_

_x0007__x0007__x0007__x0007_ _x0007_
_x0007_ _x0007_

_x0007_

_x0007__x0007_
_x0007__x0007_

_x0010_

_x0010__x0016_

_x0010_
_x0010__x0010__x0010_

_x0016__x0012__x0016_

_x0010__x0016__x0016__x0010_
_x0012_

_x0012_

_x0010_

_x0007__x0007_

_x0010_

_x0007_

_x0007_

_x0007__x0007__x0007_
_x0007_
_x0007_ _x0007__x0007_
_x0007_
_x0007_ _x0007_
_x0007_ _x0007_ _x0007__x0007_ _x0007_
_x0007__x0007_
_x0007_
_x0007__x0007_
_x0007_

_x0007_

_x0007_

_x0012__x0010_

_x0010_

_x0010__x0012_
_x0016__x0012_
_x0012__x0012__x0012__x0012__x0010__x0012__x0016__x0012__x0012__x0010__x0012__x0010
__x0012_
_x0012_

_x0010_

_x0010_

_x0010_

_x0007_
_x0007_

_x0007_
_x0007_

_x0007_ _x0007_

_x0007_
_x0007_
_x0007__x0007__x0007_

_x0007__x0007__x0007__x0007__x0007_ _x0007_ _x0007__x00
07_

_x0007_ _x0007__x0007__x0007_ _x0007__x0007__x0007_
_x0007_

_x0007_
_x0007_
_x0007_ _x0007_

_x0007__x0007_

_x0007_

_x0007__x0007__x0007_

_x0007_

_x0007_
_x0010__x0007_

_x0012_

_x0012__x0016__x0010_
_x0010__x0010_

_x0010__x0012__x0010__x0012__x0010__x0012__x0010__x0010_
_x0016__x0012__x0010__x0016__x0012__x0016__x0016__x0012__x0012__x0012__x0010__x0016
__x0012_

_x0012_

_x0010__x0012_

_x0010_

_x0007_

_x0007_

_x0007_

_x0007_

_x0007__x0007_ _x0007_
_x0007__x0007_
_x0007__x0007_

_x0007_ _x0007__x0007_
_x0007__x0007_ _x0007_

_x0007_ _x0007_ _x0007_ _x0
007_
_x0007__x0007_
_x0007__x0007__x0007__x0007__x0007__x0007_

_x0007__x0007_ _x0007_ _x0007__x0007_
_x0007__x0007_

_x0007_

_x0010_

_x0010_

_x0010_

_x0010_

_x0012__x0012_

_x0010_

_x0010__x0012__x0010__x0012__x0016_

_x0016__x0010__x0012__x0012_

_x0016__x0012__x0012__x0016__x0016__x0010__x0012_

_x0016_

_x0010_

_x0007_

_x0007_

_x0007_

_x0007__x0007_
_x0007_
_x0007__x0007__x0007__x0007__x0007__x0007__x0007_

_x0007__x0007__x0007_ _x0007_ _x0007_ _x0007_
_x0007_
_x0007__x0007_ _x0007__x0007__x0007__x00
07__x0007_ _x0007_

_x0007__x0007_

_x0007_
_x0007_

_x0007_

_x0010_

_x0012_

_x0012_

_x0010_

_x0012__x0012__x0019__x0010__x0019__x0012__x0010__x0010__x0010__x0012_

_x0012_

_x0010_

_x0012_

_x0007_

_x0007__x0007_

_x0007__x0007_

_x0007_ _x0007_
_x0007__x0007_ _x0007__x0007_ _x0007_ _x0007_ _x0007__x0007_
_x0007_
_x0007_ _x0007_ _x0007_ _x0007_
_x0007_
_x0007_ _x0007__x0007_ _x0007_

_x0007__x0007__x0007__x0007_

_x0007_ _x0007__x0007_

_x0012_

_x0007__x0010_

_x0010_

_x0010__x0010__x0010_

_x0012_

_x0010__x0016__x0012_

_x0016__x0012_

_x0016__x0016__x0016__x0016__x0016__x0010__x0012__x0010_

_x0010_

_x0007_

_x0007__x0007_

_x0007_

_x0007_

_x0007_ _x0007_

_x0007_

_x0007__x0007__x0007__x0007__x0007__x0007__x0007_

_x0007_ _x0007_ _x0007_

_

x0007__x0007_

_x0007_ _x0007_ _x0007__x0007__x0007__x0007_

_x0007__x0007__x0007__x0007__x0007__x0007__x0007__x0007__x0007_

_x0007__x0007_ _x0007_

_x0007__x0007_

_x0007_

_x0007_

_x0012_

_x0012__x0010_

_x0012_

_x0012_

_x0012_

_x0010__x0010__x0010__x0012__x0010_

_x0010_

_x0007_ _x0007_
_x0007__x0007_

_x0007_

_x0007_

_x0007_
_x0007_

_x0007_ _x0007_

_x0007__x0007_
_x0007_
_x0007_
_x0007_ _x0007_
_x0007_ _x0007_ _x0007_ _x00
07__x0007__x0007__x0007_ _x0007__x0007_
_x0007__x0007_ _x0007__x0007_ _x0007_

_x0007__x0007__x0007__x0007_

_x0007_

_x0010_

_x0010_

_x0010_
_x0010_

_x0012__x0010_

_x0010__x0012__x0010__x0016__x0012__x0010__x0016__x0012__x0012__x0012__x0012__x0012
_

_x0007_

_x0010__x0007__x0007_

_x0007_

_x0007_

_x0007__x0007__x0007_

_x0007__x0007_
_x0007__x0007_

_x0007_

_x0007__x0007_ _x0007_
_x0007_ _x000
7_ _x0007_ _x0007__x0007__x0007_ _x0007_

_x0007__x0007_

_x0007_

_x0007__x0007__x0007_

_x0010_

_x0010__x0010_

_x0010_

_x0010_

_x0010__x0012_
_x0012_

_x0012__x0010__x0012__x0010__x0019__x0012__x0012__x0012__x0016__x0016__x0012__x0010_

_x0010_

_x0007_

_x0007__x0007_

_x0007_ _x0007_

_x0007_

_x0007_ _x0007_

_x0007_ _x0007__x0007__x0007_
_x0007_
_x0007__x0007_

_x0007_ _x0007_

_x0007__x0007_

_x0007__x0007_ _x0007__x0007__x0007__x0007_
_x0007_ _x0007_

_x0007_
_x0007__x0007_

_x0007_

_x0012__x0010_
_x0010_

_x0012__x0012_

_x0010_
_x0010_

_x0012_

_x0010__x0010_

_x0012__x0012_

_x0010__x0010_

_x0010__x0010_

_x0012_

_x0010_

_x0007_ _x0007_
_x0012_
_x0007_

_x0007_

_x0007_

_x0007_

_x0007_

_x0007__x0007_

_x0007__x0007_

_x0007_
_x0007_ _x0007_
_x0007_ _x0007_ _x0007__x0007_
_x0007_ _x0007__x0007_
_x0007__x0007__x0007_ _x0007__x0007__x0007_

_x0007__x0007__x0007_
_x0007_ _x0007__x0007_

_x0007_

_x0007_

_x0007_

_x0012_

_x0012_

_x0010_

_x0010_

_x0016_

_x0012__x0012__x0012__x0010__x0012__x0012_

_x0019_

_x0012_

_x0012_

_x0010_

_x0010_
_x0012_

_x0010_

_x0007_

_x0007_

_x0007_

_x0007_

_x0007__x0007__x0007_ _x0007_

_x0007__x0007_

_x0007_ _x0007_ _x0007_

_x0007_
_x0007_ _x0007__x0007_

_x0007__x0007_
_x0007_
_x0007_

_x0007_

_x0007_
_x0007_

_x0007_
_x0007_

_x0010_

_x0010_

_x0010__x0016_

_x0010_

_x0012__x0010__x0012_

_x0012__x0016_

_x0012__x0010__x0010_

_x0012_

_x0010__x0007_
_x0010_

_x0016_
_x0012__x0010_

_x0010__x0012_

_x0010__x0012__x0012_

_x0010__x0010_

_x0012__x0012_

_x0016__x0012__x0010_
_x0010_
_x0010_

_x0010_
_x0010__x0010_

_x0012_

_x0007_ _x0007_
_x0007_

_x0007_

_x0007_

_x0007_ _x0007_

_x0007__x0007__x0007_
_x0007__x0007__x0007__x0007_ _x0007_
_x0007_ _x0007__x0007__x0007_

_x0007_ _x0007_
_x0007__x0007__x0007_ _x0007__x0007__x0007__x0007_
_x0007__x0007_
_x0007_ _x0007_

_x0007_

_x0007_

_x0007_

_x0007_

_x0010_

_x0010_

_x0010__x0012_

_x0010__x0012__x0012__x0012__x0012__x0016__x0012__x0012__x0012_

_x0010__x0012_

_x0010__x0010_

_x0012_

_x0010_
_x0010_

_x0007_
_x0007_

_x0007_

_x0007__x0007__x0007_
_x0007__x0007_
_x0007_ _x0007__x0007__x0007__x0007_ _x0007__x0007_

_x0007__x0007_ _x0007__x0007_ _x0007_ _x0007_
_x0007_
_x0007__x0007_
_x0007__x0007__x0007__x0007__x0007__x0007__x0007_
_x0007__x0007__x0007_ _x0007__x0007__x0007__x0007_ _x0007_

_x0007_

_x0007_

_x0010_

_x0010__x0012_
_x0012__x0012__x0010__x0010_

_x0012__x0012_

_x0010__x0010__x0012_

_x0010__x0016__x0012__x0010_

_x0010_

_x0010_

_x0012_

_x0010__x0012_

_x0010_
_x0010__x0007_

_x0010_

_x0010_

_x0007_

_x0007_

_x0007__x0007_

_x0007_
_x0007_
_x0007__x0007_ _x0007__x0007_ _x0007_ _x0007_
_x0007_ _x0007_ _x0007_
_x0007_
_x0007__x0007_ _x0007_ _x0007__x0007__x0007__x0007_ _x0007_
_x0007_

_x0007_
_x0007__x0007_

_x0007__x0007_

_x0007_

_x0007__x0007__x0007_

_x0010_

_x0010_

_x0010_

_x0012_

_x0010_

_x0010__x0012_

_x0010__x0010__x0012__x0010__x0010_

_x0010_

_x0007_

_x0007_

_x0007_
_x0007_

_x0007__x0007_

_x0007__x0007_
_x0007_ _x0007_

_x0007_ _x0007_ _x000
7_ _x0007__x0007__x0007__x0007__x0007_
_x0007_
_x0007__x0007_
_x0007__x0007__x0007_

_x0007_ _x0007_
_x0007_
_x0007_
_x0007_

_x0007_

_x0012_
_x0010__x0010__x0010_

_x0010__x0012_

_x0010__x0010_
_x0012__x0012_

_x0012_

_x0010__x0010__x0010__x0016_

_x0016_

_x0012__x0010__x0010_

_x0010_
_x0010_

_x0010_

_x0010_
_x0010_

_x0010_

_x0010_

_x0007__x0007_

_x0007_
_x0007__x0007__x0007_ _x0007_

_x0007_ _x0007_ _x0007_

_x0007_ _x0007__x0007
__x0007__x0007_

_x0007_ _x0007__x0007_
_x0007__x0007_

_x0007_
_x0007_

_x0007_

_x0007_
_x0007_
_x0007_
_x0007_

_x0007_

_x0010__x0012_

_x0012_

_x0012_

_x0010__x0010__x0012_

_x0016_
_x0012_

_x0016__x0010__x0012__x0012_

_x0010__x0012_

_x0012_

_x0012_

_x0010_

_x0010__x0007_

_x0010_

_x0007_

_x0007_
_x0007_ _x0007__x0007_

_x0007__x0007__x0007_
_x0007__x0007__x0007_ _x0007__x0007_
_x0007__x0007_ _x0007_ _x0007_ _x0007_ _x0007_
_x0007_ _x0007_
_x0
007_ _x0007__x0007_

_x0007_

_x0007__x0007_

_x0007__x0007__x0007__x0007_

_x0007_ _x0007_ _x0007_

_x0007_

_x0010_

_x0010__x001D__x0012__x0010__x0010__x0012__x0019__x0010_

_x0012__x0010_

_x0010_

_x0010_

_x0010_

_x0007__x0007__x0007_
_x0007_

_x0007_
_x0007__x0007__x0007_

_x0007_
_x0007__x0007__x0007__x0007__x0007__x0007__x0007__x0007_ _x0007_

_x0007__x0007_

_x0007_

_x0007__x0007__x0007_

_x0007__x0007__x0007__x0007__x0007__x0007__x0007__x0007_

_x0007_

_x0007_
_x0007_

_x0007_

_x0007__x0007__x0007_

_x0010__x0010_

_x0010_

_x0012__x0010__x0016_

_x0010__x0010__x0012_

_x0010_

_x0012_

_x0012_

_x0012_

_x0012_

_x0010__x0010__x0010_

_x0010_

_x0007_

_x0010_

_x0010_

_x0010_

_x0007_

_x0007_

_x0007_

_x0007_ _x0007_
_x0007__x0007__x0007_
_x0007_
_x0007__x0007_ _x0007_
_x0007_
_x0007_ _x0007_ _x0007_ _x0007_
_x0012__x0007__x0007__x0007__x0007__x0007__x0007_
_x0007_

_x0007_

_x0007_

_x0007__x0007_
_x0007__x0007__x0007_

_x0010__x0010_

_x0012_

_x0010_

_x0010_

_x0010_
_x0012__x0010__x0012__x0010__x0012_

_x0010_

_x0012_

_x0012_

_x0010_

_x0010_

_x0010_

_x0012_

_x0010__x0010_

_x0007_

_x0007_

_x0007_

_x0007_
_x0007__x0007_
_x0007_

_x0007__x0007_ _x0007__x0007__x0007_

_x0007_

_x0007__x0007_ _x0007_ _x0007_ _x
0007_

_x0007_ _x0007__x0007_

_x0007_ _x0007__x0007__x0007_ _x0007__x0007__x0007_
_x0007__x0007_ _x0007_ _x0007_

_x0007_ _x0007_
_x0007_

_x0007_

_x0012_

_x0010_

_x0010__x0010_

_x0010__x0012_

_x0010__x0010_

_x0012__x0012_

_x0012_

_x0010__x0012_

_x0016__x0012__x0012_

_x0012__x0012_

_x0010_

_x0012_
_x0010_

_x0010_

_x0010_

_x0010__x0010__x0010_

_x0007_

_x0007_

_x0007_

_x0007_
_x0007__x0007_ _x0007_ _x0007__x0007_
_x0007_
_x0007__x0007__x0007_ _x0007_ _x0007_

_x0007__x0007_ _x0007__x0007__x0007__x0007_ _x0007__x0007__x0007__x00
07_

_x0007_
_x0007_
_x0007__x0007_ _x0007_

_x0007_

_x0007_

_x0007__x0007_

_x0010_

_x0010__x0012__x0010__x0010__x0010_

_x0010_

_x0012__x0010__x0010_

_x0010__x0012_

_x0010_

_x0010_

_x0010_

_x0010_

_x0010_

_x0007_

_x0007_
_x0007_

_x0007_

_x0007__x0007__x0007_ _x0007_
_x0007_ _x0007__x0007_ _x0007_

_x0007__x0007_ _x0007_

_x0007_

_x0007_

_x0007_ _x0007__x0007_

_x0007_

_x0010_

_x0010_

_x0010_
_x0010_

_x0012__x0012__x0010__x0016_

_x0010_

_x0010__x0010__x0010_

_x0012__x0010_

_x0010__x0010__x0010_

_x0010_
_x0012_

_x0007_

_x0007_

_x0007_ _x0007_
_x0007__x0007_ _x0007_
_x0007_ _x0007_ _x0007_
_x0007_
_x0007__x0007_
_x0007_ _x0007_ _x0007__x0007_ _x0007_
_x0007__x0007__x0007__x0007_
_x0007_

_x0007_

_x0007_
_x0007_

_x0007_

_x0007_

_x0010_

_x0012__x0010__x0012__x0010_

_x0010__x0010__x0016__x0016_

_x0012_
_x0012_

_x0010_

_x0010_

_x0007__x0007__x0007_

_x0007_

_x0007_

_x0007_
_x0007_

_x0007__x0007_

_x0007_

_x0007_ _x0007_

_x0007_ _x0007__x0007__x0007_

_x0007_ _x0007_

_x0007_

_x0007_

_x0007__x0007__x0007_

_x0007_

_x0007_

_x0010_

_x0010_

_x0012__x0016_
_x0010_

_x0010_

_x0012_

_x0012__x0012__x0012__x0010__x0012__x0010__x0019_

_x0010_

_x0010_

_x0012_
_x0012_

_x0010_

_x0010_

_x0007_

_x0007_

_x0007__x0007__x0007_
_x0007_

_x0007_ _x0007_
_
x0007_
_x0007__x0007_
_x0007_ _x0007_
_x0007_

_x0007__x0007_
_x0007_

_x0007_

_x0007_

_x0010_

_x0010_
_x0010_

_x0010_

_x0012_
_x0012_ _x0010_

_x0010_

_x0012_

_x0016_

_x0010_

_x0010_

_x0010_

_x0010_

_x0010_
_x0012_

_x0012_

_x0007_

_x0010_

_x0007_
_x0007_

_x0007_

_x0007_

_x0007__x0007_
_x0007__x0007__x0007__x0007_
_x0007_
_x0007_ _x0007_
_x0007_ _x0007__x0007__x0007_ _x0007__x0007_
_x0007_ _x0007__x0007__x0007_
_x0007_

_x0007_

_x0007_
_x0007__x0007_

_x0007_ _x0007_

_x0012_

_x0012_

_x0012__x0019__x0010_

_x0010_

_x0012_

_x0010_

_x0012__x0010_
_x0007_

_x0012_

_x0010_ _x0010_

_x0007__x0007_

_x0007_

_x0007_

_x0007_

_x0007__x0007__x0007__x0007_ _x0007__x0007__x0007_ _x0007_ _x0007_

_x0007_
_x0007_ _x0007_ _x0007_
_x0007_

_x0007__x0007_ _x0007__x0007_

_x0007_
_x0007_
_x0007_
_x0007_

_x0007_

_x0007__x0007_

_x0007_

_x0010_

_x0010_

_x0010__x0012_
_x0012_

_x0016__x0010__x0016_

_x0012_

_x0010_

_x0012__x0012_

_x0012_
_x0010_
_x0012__x0010__x0010_

_x0010__x0012_
_x0010_

_x0007_

_x0007__x0007_

_x0007_

_x0007__x0007_ _x0007__x0007_

_x0007__x0007__x0007__x0007_ _x0007_

_x0007_

_x0007_ _x0007__x0007_

_x0007_

_x0007_

_x0007_

_x0007_

_x0010__x0010_

_x0010_

_x0012_

_x0010_
_x0012_

_x0010__x0012_

_x0012__x0012__x0016__x0016__x0012_

_x0016__x0012_

_x0010_

_x0010_

_x0010_
_x0010_

_x0010_

_x0007_

_x0007__x0007_

_x0007_

_x0007__x0007_

_x0007_

_x0007_ _x0007__x0007_
_x0007__x0007__x0007_

_x0007_

_x0007_

_x0007__x0007__x0007__x0007_

_x0007_

_x0007_

_x0007__x0007_
_x0007_
_x0007_

_x0010_

_x0010__x0010_

_x0010__x0012_

_x0010_

_x0010__x0010_

_x0010_
_x0012__x0012_

_x0012__x0012__x0012_

_x0010__x0012_

_x0012__x0010__x0012_

_x0010_

_x0012_ _x0007_

_x0010_

_x0007__x0007_

_x0007__x0007_
_x0007_

_x0007_ _x0007_ _x0007__x0007__x0007__x0007_

_x0007__x0007__x0007_
_x0007_ _x0007_
_x0007__x0007__x0007_

_x0007__x0007_

_x0007_

_x0007_

_x0007_ _x0007_

_x0010__x0010_

_x0012_

_x0012_

_x0012__x0012__x0012_

_x0010__x0012_
_x0012_

_x0016__x0010_

_x0010_

_x0010_

_x0007_

_x0007_

_x0007_

_x0007_

_x0007_
_x0007_ _x0007__x0007_

_x0007__x0007_ _x0007_
_x0007_
_x0007__x0007_
_x0010_
_x0007_ _x0007_ _x0007__x0007_
_x0007_ _x0007__x0007__x0007_

_x0007_ _x0007__x0007_
_x0007_ _x0007_

_x0007_

_x0007__x0010_

_x0007_

_x0010__x0010_

_x0010_

_x0012_

_x0010_

_x0016__x0019__x0016__x0012__x0019__x0010__x0010__x0016_

_x0010__x0010__x0019__x0010_

_x0007_

_x0010__x0010_
_x0007_

_x0007_ _x0007_

_x0007_
_x0007_

_x0007__x0007_
_x0007__x0007_

_x0007_ _x0007_
_x0007_
_x0007_
_x0007__x0010_
_x0007_ _x0007__x0007_
_x0007_

_x0007_

_x0007_ _x0007__x0007__x0007__x0007_
_x0007_
_x0007_

_x0007_

_x0007_

_x0007_

_x0010_

_x0010__x0012_

_x0012__x0010__x0010_

_x0012_

_x0012__x0010_

_x0016__x0010_

_x0012_

_x0012_

_x0007_

_x0007_

_x0007_
_x0007_
_x0007__x0007_
_x0007_ _x0007__x0007_
_x0010__x0010__x0007_ _x0007_
_x0007__x0007_ _x0007__x0007_ _x0007__x0007_
_x0007_
_x0007_ _x0007_

_x0007__x0007__x0007_ _x0007__x0007_
_x0007_
_x0007_
_x0007_

_x0007_

_x0007_

_x0007_

_x0007_

_x0010_

_x0010_

_x0010__x0012_

_x0010_

_x0010__x0010__x0010_

_x0010_

_x0010__x0010_

_x0010_

_x0010_

_x0007_

_x0007_

_x0010_

_x0007_

_x0007_

_x0007_ _x0007_
_x0007__x0007_
_x0007__x0007_ _x0007
_ _x0007
_

_x0007_ _x0007_ _x0007__x0007_ _x0007_

_x0007_
_x0007_

_x0007_
_x0007__x0007_

_x0007_

_x0007_

_x0007_ _x0007_

_x0016_

_x0012_

_x0012_

_x0012__x0012_

_x0019_

_x0012_

_x0010_

_x0010_

_x0007_

_x0007_

_x0007_

_x0007__x0007_ _x0007__x0007_ _x0007__x0007_
_x0007_ _x0007_

_x0007_ _x0007_ _x0007_ _x0010_
_x0007__x0007__x0007__x0007__x0007_ _x0007__x0007__x0007__x0007_

_x0007_ _x0007_

_x0007__x0007_
_x0007_
_x0007__x0007_

_x0007_

_x0016_

_x0010_

_x0012__x0010_

_x0010_

_x0012_
_x0010_

_x0012__x0012__x0010_

_x0010_

_x0012_

_x0010__x0010_

_x0010__x0007_
_x0007__x0007_

_x0007_

_x0010_

_x0007_

_x0007__x0007__x0007__x0007_ _x0007_

_x0007__x0007_ _x0007_
_x0007_ _x0007__x0007__x0007__x0007__x0007__x0007_ _x0007_
_x0007_
_x0007_ _x0007_ _x0007_ _x0007_ _x0007_

_x0007_

_x0007_ _x0007_

_x0007_

_x0007_
_x0012_

_x0012_
_x0012_

_x0012__x0012__x0010__x0010_
_x0010_

_x0012__x0010_
_x0010__x0012__x0016_

_x0010_

_x0007_

_x0007_

_x0007_

_x0007_
_x0007_

_x0007__x0007__x0007_

_x0007_
_x0007_ _x0007_ _x0007_
_x0007__x0007__x0007_ _x0007__x0007__x0007__x0007_
_x0007__x0007__x0007__x0007_
_x0007_

_x0007_ _x0007_

_x0007_

_x0007_

_x0007__x0007__x0007_

_x0010_
_x0010_

_x0010__x0010_

_x0010_

_x0016__x0012__x0010__x0012_

_x0016_

_x0010_

_x0010_

_x0010_

_x0010_

_x0010_

_x0010_

_x0010_

_x0007_ _x0007_

_x0007__x0007_
_x0007__x0007__x0007_ _x0007_ _x0007_

_x0007_

_x0007_ _x0007_ _x0007__x0007__x0007_
_x0007__x0007_

_x0007__x0007_ _x0007_
_x0007_ _x0007_
_x0007_

_x0007_

_x0007_

_x0010__x0007_

_x0007_
_x0010_

_x0012_

_x0010_

_x0010_

_x0012__x0010_

_x0010__x0012__x0010_

_x0012__x0012__x0012__x0012_

_x0010_

_x0010__x0010_

_x0007_

_x0012_

_x0010__x0007_

_x0007_

_x0007_

_x0007__x0007__x0007__x0007_
_x0007__x0007__x0007_ _x0007_

_x0007__x0007__x0007__x0007__x0007_
_x0007__x0007__x0007_

_x0007_
_x0007__x0007__x0007__x0007_
_x0007__x0007__x0007_ _x0007_

_x0007_

_x0007_

_x0010_

_x0007_

_x0010_

_x0010_

_x0010_

_x0010__x0012_

_x0010_

_x0012__x0010__x0012_

_x0012_

_x0016_

_x0012__x0012_

_x0010_
_x0010_

_x0012_

_x0007_

_x0010_

_x0010_ _x0007_

_x0007_

_x0007_
_x0007_
_x0007__x0007__x0007_
_x0007__x0007__x0007__x0007__x0007__x0007_

_x0007__x0007_ _x0007__x0007_
_x0007__x0007_
_x0007__x0007_ _x0007__x0007_ _x0007_
_x0007_

_x0007_

_x0007__x0007__x0007_

_x0010_

_x0010_

_x0010_

_x0010_

_x0012_

_x0010__x0010__x0010__x0016__x0012__x0010__x0012_

_x0010_

_x0010_

_x0010_

_x0010_

_x0007_

_x0007__x0007_

_x0007_

_x0007__x0007__x0007__x0007__x0007_
_x0007__x0007__x0007__x0007_ _x0007_
_x0007_

_x0007_ _x0007__x0007_
_x0007__x0007_
_x0007_
_x0007__x0007_

_x0007_

_x0007_ _x0007__x0007_

_x0007_

_x0007_

_x0007_

_x0010__x0007_

_x0012__x0010_

_x0010_

_x0010_

_x0010__x0010__x0012_

_x0012_

_x0010_

_x0012__x0012_

_x0012__x0012__x0010_

_x0010_

_x0007__x0007_

_x0007_

_x0007_ _x0007__x0007_ _x0007__x0007__x0007_
_x0007_ _x0007__x0007_
_x0007_
_x0007_ _x0007_ _x0007_ _x0007_

_x0007_
_x0007_ _x0007__x0007_ _x0007__x0007_ _x0007__x0007_

_x0007__x0007__x0007_

_x0010_

_x0012_

_x0010_

_x0010_
_x0012_

_x0012__x0012_

_x0010_

_x0016__x0010__x0012_

_x0012_

_x0007__x0007__x0007_

_x0007__x0007_
_x0007__x0007__x0007__x0007__x0007__x0007__x0007_
_x0007_
_x0007_
_x0007_ _x0007_

_x0007_ _x0007__x0007_
_x0007__x0007_ _x0007_ _x0007_ _x0007_

_x0007__x0007_

_x0007_

_x0007__x0007_

_x0010_

_x0010_

_x0010_
_x0012_

_x0010_

_x0010_

_x0012__x0010__x0012_

_x0010_

_x0010__x0016__x0007_

_x0010__x0010_
_x0012_

_x0012_

_x0010_
_x0010_

_x0010_

_x0007_

_x0007__x0007_
_x0007__x0007_

_x0007__x0007_
_x0007__x0007_
_x0007_ _x0007_
_x0007__x0007_
_x0007_
_x0007__x0007__x0007_

_x0010__x0010_

_x0007_

_x0007_
_x0007_

_x0007_

_x0007_ _x0007__x0007__x0007__x0007__x0007__x0007_

_x0012_

_x0010_
_x0010_

_x0010_

_x0016_

_x0012_

_x0010__x0010__x0012_
_x0010_

_x0012__x0010_

_x0010_

_x0010_

_x0007__x0010_

_x0007__x0007_
_x0007_

_x0007__x0007_
_x0007__x0007_ _x0007_ _x0007_ _x0007_ _x0007_ _x0007__x0007_
_x0007__x0007_
_x0007_ _x0007_

_x0007_

_x0007_

_x0007__x0007__x0007_ _x0007_

_x0007__x0007__x0007__x0007_

_x0007__x0007_

_x0010__x0007_

_x0007_

_x0007_

_x0007_

_x0007_

_x0010_

_x0012_

_x0010_

_x0010__x0016__x0016_
_x0010__x0016__x0012__x0010__x0010_

_x0012_

_x0012_

_x0012_

_x0016__x0012__x0012_

_x0019__x0010__x0010__x0010__x0010__x0010__x0012_

_x0012_

_x0010_

_x0010__x0010_

_x0007_

_x0007_
_x0007__x0007_

_x0007_

_x0007__x0007__x0007__x0007__x0007_
_x0007_

_x0007_ _x0007__x0007__x0007__x0007_
_x0007__x0007_
_x0007_ _x0007_
_x0007_ _x0007__x0007_

_x0007_

_x0010_
_x0007_

_x0010_

_x0012__x0010__x0010__x0010_

_x0012_

_x0012__x0010__x0012__x0010__x0012__x0012_

_x0012__x0012_

_x0012__x0012_

_x0010_

_x0016_

_x0012_

_x0007_

_x0007_
_x0007_

_x0007__x0007__x0007_

_x0007_

_x0007_
_x0007_ _x0007_
_x0007_

_x0007_ _x0007__x0007__x0007_ _x0007__x0007__x0007_
_x0007__x0007_

_x0007__x0007__x0007_
_x0007_

_x0007_

_x0007_

_x0007_

_x0010__x0010__x0010_

_x0010_

_x0010_

_x0012_

_x0010__x0016__x0010_

_x0012_

_x0010__x0012__x0016__x0012__x0016_

_x0012__x0016_

_x0010__x0012_

_x0010__x0010__x0019__x0016__x0010_

_x0012_

_x0012__x0010__x0016__x0010__x0016__x0012_

_x0016_

_x0010_

_x0010_

_x0010_

_x0012_
_x0012_

_x0010__x0012__x0012_

_x0010_

_x0012__x0010__x0012_

_x0012__x0012__x0010__x0010_

_x0010__x0010_

_x0007_

_x0007_

_x0007_ _x0007_ _x0007_
_x0007_

_x0007_ _x0007__x0007_

_x0007__x0007__x0007__x0007__x0007__x0007__x0007__x0007__x0007_

_x0007__x0007_ _x0007__x0007_

_x0007__x0007_

_x0007__x0007__x0007__x0007_
_x0007_
_x0007__x0007__x0007_

_x0007__x0007_

_x0010_

_x0010_

_x0012_

_x0010_

_x0010__x0012_

_x0010_

_x0012__x0012__x0010__x0016__x0010_

_x0012_

_x0012_

_x0010__x0010_

_x0010_

_x0010_

_x0007__x0007_

_x0007_
_x0007_

_x0007_

_x0007_
_x0007_ _x0007__x0007_
_x0007_
_x0007_ _x0007_ _x0007_ _x0007_ _x0007__x000
7__x0007__x0007__x0007_

_x0007_

_x0007_
_x0007_

_x0007_

_x0007__x0007_ _x0007__x0007__x0007_ _x0007_

_x0007_

_x0007_

_x0007__x0007_

_x0007_

_x0012_

_x0010_
_x0012__x0012_

_x0010_

_x0007_

_x0007__x0007_ _x0007_
_x0007_

_x0007__x0007_

_x0012__x0007_
_x0007_
_x0007_

_x0007_
_x0007__x0007__x0007__x0007_ _x0007_

_x0007_
_x0007_ _x0007_ _x0007__x0007__x0007__x0007_

_x0007_

_x0007_ _x0007_

_x0007_

_x0007_

_x0007_

_x0007_

_x0007_

_x0007_

_x0010__x0010_

_x0010_

_x0010__x0012_

_x0010_

_x0010_

_x0010__x0012__x0010_

_x0012__x0012_

_x0007_

_x0007_

_x0007_
_x0007_

_x0007_

_x0007_

_x0010_
_x0007_

_x0007_

_x0007__x0007__x0007_
_x0007__x0007_ _x0007_ _x0007_
_x0007__x0007_
_x0007__x0007__x0007__x0007__x0007_
_x0007__x0007__x0007_ _x0007_ _x0007_ _x0007_

_x0007_

_x0010__x0010_

_x0007_

_x0016_

_x0010_

_x0010_

_x0016__x0012_

_x0010__x0010__x0010_
_x0016__x0010_

_x0010_

_x0007__x0007__x0007__x0007__x0007_

_x0007_

_x0007__x0007_
_x0007__x0007_

_x0007_
_x0007__x0007_
_x0007__x0007_ _x0007_ _x0007_
_x0007_ _x0007_ _x0007_
_x0007__x0007__x0007_ _x0007_ _x0007_ _x0007__x0007_ _x0007_

_x0007__x0007__x0007__x0007_

_x0007_

_x0007_

_x0016__x0012_

_x0007_

_x0010_

_x0012_

_x0010__x0010_

_x0010__x0010_
_x0010_

_x0010__x0012__x0010_

_x0012_

_x0010_

_x0012__x0012_

_x0012__x0019_

_x0010__x0012__x0010_

_x0010_

_x0007_

_x0007_

_x0007__x0007_
_x0007_

_x0007__x0007_
_x0007__x0007_
_x0007__x0007__x0007__x0007__x0007_ _x0007_ _x0007_
_x0007_
_x0007_ _x0007__x0007_ _x0007_ _x0007__x0007_ _x0007_ _x0007_
_x0007_ _x0007_
_x0007_

_x0007_
_x0007_

_x0007__x0007__x0007_

_x0010_

_x0007__x0007_

_x0012__x0010_

_x0010_

_x0012__x0012__x0012__x0016_

_x0007_

_x0007_

_x0007__x0007_

_x0007_

_x0007_
_x0007_
_x0007_
_x0007_

_x0007__x0007_
_x0007_
_x0007_
_x0007_
_x0007_ _x0007__x0007_ _x0007_ _x0007_ _x0007_
_x0007_ _x0007_ _x0007_
_x0007__x0007_ _x0007_ _x0007_ _x0007_

_x0007__x0007__x0007_

_x0007_
_x0007_

_x0007_

_x0010_

_x0010_
_x0010_

_x0010__x0010_

_x0012_

_x0012_

_x0010_

_x0010__x0012__x0012_

_x0012_

_x0010_

_x0007_

_x0007_

_x0007_ _x0007_ _x0007__x0007__x0007_

_x0007__x0007__x0007_
_x0007_
_x0007_

_x0007__x0007__x0007_

_x0007_
_x0007__x0007__x0007_
_x0007_
_x0007_
_x0007_
_x0007_ _x0007__x0007__x0007__x0007
_ _x0007__x0007__x0007__x0007_

_x0007_

_x0007_

_x0012_

_x0012__x0010_

_x0012_

_x0010_
_x0010_
_x0010_

_x0010_

_x0012_

_x0010__x0012__x0012_

_x0012_

_x0012_

_x0007_

_x0007__x0007__x0007_
_x0007_

_x0007__x0007_ _x0007_ _x0007_
_x0007_ _x0007_
_x0007_

_x0007__x0007__x0007_
_x0007__x0007__x0007_ _x0007__x0007_

_x0007_

_x0007_

_x0007_

_x0007_

_x0010_

_x0007_

_x0010_

_x0007_

_x0010_

_x0012_

_x0012__x0012__x0010_

_x0010_

_x0007__x0007_

_x0007__x0007__x0007_

_x0007_
_x0007__x0007_ _x0007__x0007__x0007__x0007__x0007_
_x0007_ _x0007_ _x0007_

_x000
7_ _x0007__x0007_

_x0007_
_x0007_

_x0007_

_x0007__x0007__x0007__x0007__x0007__x0007_

_x0007_

_x0007__x0010_

_x0010_

_x0012_

_x0010__x0012_

_x0010_

_x0010__x0010_
_x0010__x0012_

_x0010__x0010_

_x0007_

_x0010_

_x0010_

_x0007_
_x0007_

_x0007_
_x0007__x0007_

_x0007_

_x0007_
_x0007__x0007__x0007_
_x0007_

_x0007__x0007__x0007__x0007__x0007_
_x0007__x0007__x0007_

_x0007_
_x0007_

_x0007__x0007_

_x0007__x0007_

_x0010_

_x0012_

_x0010_

_x0010_

_x0012__x0012_

_x0012_
_x0010_
_x0010__x0010_

_x0010_

_x0007_

_x0007__x0007_

_x0007_ _x0007_

_x0007__x0007__x0007_ _x0007_ _x0007__x0007_
_x0007_ _x0007__x0007_
_x0007_

_x0007_ _x0007__x0007_ _x0007_ _x0007__x0007_
_x0007__x0007_ _x0007_

_x0007_

_x0007_

_x0007_

_x0010__x0010_

_x0010_

_x0012_

_x0012__x0010_

_x0010_

_x0007__x0010_

_x0007_

_x0010_

_x0007_

_x0007_

_x0007_

_x0007_
_x0007__x0007_ _x0007_
_x0007_

_x0007__x0007_
_x0007__x0007__x0007__x0007__x0007__x0007__x0007__x0007_
_x0007_

_x0007_ _x0007_

_x0007_

_x0007_

_x0010_

_x0012_

_x0010_

_x0010_

_x0010_

_x0007_

_x0007__x0007_

_x0007_ _x0007__x0007_

_x0007__x0007_
_x0007_

_x0007__x0007__x0007_
_x0007__x0007_

_x0007__x0007__x00
07_ _x0007_ _x0007_
_x0007__x0007_

_x0007_ _x0007_

_x0012_

_x0010__x0010_

_x0010__x0010_

_x0012__x0010_

_x0007_

_x0007_ _x0007_
_x0007__x0007__x0007__x0007_ _x0007__x0007_
_x0007__x0007_ _x0007_
_x0007_ _x
0007_
_x0007_ _x0007__x0007_ _x0007_

_x0007__x0007_

_x0007_
_x0007_ _x0007_
_x0007_

_x0010_

_x0010_

_x0010__x0010_

_x0007_ _x0007_

_x0007_
_x0007__x0007_

_x0007__x0007_ _x0007__x0007_

_x0007__x0007_

_x0007__x0007__x0007__x0007_
_x0007_

_x0007_ _x0007_

_x0007_

_x0007_

_x0010__x0012_

_x0010__x0016_

_x0010_

_x0007_
_x0007_

_x0007__x0007_

_x0007__x0007__x0007_

_x0007__x0007_
_x0007_
_x0007_ _x0007_

_x0007_ _x0007__x0007_

_x0007_

_x0007_
_x0010__x0010_

_x0007_
_x0007_

_x0010_

_x0007__x0012_

_x0010_
_x0012_

_x0010_

_x0010_

_x0010_

_x0010_

_x0010_

_x0010_

_x0012__x0010_

_x0007_

_x0007_ _x0007_

_x0007_ _x0007__x0007_ _x0007__x0007__x0007__x0007__x0007_
_x0007_ _x0007_ _x0007_
_x0007_

_x0007__x0007_ _x0007_ _x0007_
_x0007__x0007_

_x0007_ _x0007__x0007_
_x0007_

_x0012_

_x0007__x0010_

_x0010_

_x0010_

_x0010_

_x0010_

_x0007_

_x0007_

_x0007_

_x0007__x0007_

_x0007_

_x0007_

_x0007__x0007_

_x0007_ _x0007__x0007__x0007__x0007__x0007__x0007__x0007_

_x0007_

_x0007_ _x0007__x0007__x0007_

_x0007__x0007__x0007_

_x0012_

_x0010_
_x0012_

_x0012_

_x0012_

_x0012_

_x0010_

_x0010_

_x0010_

_x0010_

_x0007_

_x0007_
_x0007__x0007_
_x0007_ _x0007__x0007_
_x0007_
_x0007__x0007__x0007__x0007_ _x0007_

_x0007_

_
x0007_ _x0007__x0007__x0007__x0007_ _x0007_ _x0007_

_x0007_

_x0010_

_x0010__x0012_

_x0010_

_x0010_

_x0010_

_x0010_
_x0007__x0007_

_x0007__x0007_ _x0007__x0007__x0007_
_x0007_ _x0007_ _x0007__x0007__x0007_
_x0007_

_x0007_ _x0007_
_x0007__x0007__x0007__x0007__x0007__x0007_
_x0007_

_x0007_

_x0010_

_x0012_

_x0010_

_x0010_

_x0010_

_x0012_

_x0010_

_x0007_

_x0007_
_x0007__x0007_
_x0007__x0007__x0007_
_x0007__x0007__x0007__x0007__x0007_
_x0007__x0007__x0007__x0007__x0007_ _x0007__x0007_

_x0007_ _x0007_ _x0007_

_x0007_

_x0007_

_x0010_

_x0012__x0010__x0010__x0012_

_x0012_

_x0012_

_x0012_

_x0007_

_x0007_

_x0007__x0007__x0007_
_x0007_ _x0007_
_x0007__x0007_
_x0007_
_x0007_
_x0007_ _x0007_

_x0007_

_x0007__x0007_
_x0007__x0007_ _x0007_
_x0007_
_x0007__x0007__x0007_
_x0007_

_x0010_

_x0010_

_x0010_

_x0007_

_x0007__x0007_
_x0007_
_x0007__x0007_
_x0007_ _x0007_
_x0007_

_x0007_ _x0007_ _x0007_

_x0007_
_x0007_
_x0007_
_x0007__x0007_

_x0007_

_x0007_

_x0010__x0010_
_x0012_

_x0010_

_x0012_

_x0012_

_x0007_
_x0007_

_x0007__x0007_
_x0007__x0007__x0007__x0007__x0007__x0007__x0007__x0007__x0007_

_x0007__x
0007__x0007__x0007__x0007__x0007__x0007_ _x0007_
_x0007_

_x0007_

_x0007_

_x0010_

_x0012_
_x0010__x0012_
_x0012_
_x0010_
_x0010_

_x0007_ _x0007_

_x0007_
_x0007_
_x0007__x0007_ _x0007__x0007__x0007__x0007__x0007_ _x0007__x0007_
_x0007__x0007_ _x0007_

_x0007_ _x0007_ _x0007_ _x0007__x0007_
_x0007_
_x0007_
_x0007_ _x0007__x0007_ _x0007_

_x0007_

_x0007_

_x0007_

_x0010__x0010_

_x0010_

_x0010__x0010_

_x0007_

_x0007_

_x0007__x0007_

_x0007_
_x0007_

_x0007_
_x0007_
_x0007_
_x0007__x0007__x0007__x0007__x0007__x0007_ _x0007_ _x0007_
_x0007_

_x0007_ _x0007__x0007__x0007__x0007__x0007_
_x0007_

_x0007__x0007__x0007__x0007_
_x0007__x0007__x0007_

_x0010_

_x0007_

_x0010_

_x0012_

_x0010_

_x0010_

_x0012_

_x0007__x0007_ _x0007_ _x0007__x0007_ _x0007__x0007__x0007_ _x0007_
_x0007_ _x0007__x0007__x0007__x0007__x0007_

_x0007__x0007__x0007__x0007__x0007__x0007__x0007_
_x0007_

_x0007__x0007__x0007__x0007_
_x0007_

_x0007_

_x0007_

_x0010__x0007_
_x0012_

_x0010_

_x0010_

_x0012_

_x0010_

_x0007_
_x0007__x0007_

_x0007_ _x0007_ _x0007_ _x0007__x0007_
_x0007__x0007_
_x0007_ _x0007__x0007_
_x0007_

_x0007_

_x0007_
_x0007__x0007__x0007__x0007_
_x0007_ _x0007__x0007_ _x0007__x0007_

_x0007__x0007__x0007_

_x0007_

_x0007_

_x0010_

_x0012_

_x0007_
_x0007_

_x0007__x0007__x0007_

_x0007__x0007_
_x0007_ _x0007__x0007__x0007_
_x0007_ _x0007_ _x0007_

_x0007_
_x0007_ _x0007_
_x0007__x0007__x0007__x0007__x0007_ _x0007_

_x0007__x0007_

_x0007_

_x0007_

_x0010_

_x0007_

_x0007_

_x0007_

_x0007_ _x0007_

_x0007__x0007__x0007_ _x0007_ _x0007_ _x0007__x0007_ _x
0007_

_x0007__x0007__x0007_

_x0007__x0007__x0007__x0007_
_x0007_
_x0007_

_x0007_
_x0007__x0007_

_x0010_
_x0007_

_x0010_

_x0010_

_x0012_

_x0010_

_x0010__x0010__x0010_

_x0010_

_x0007_

_x0007_

_x0007_
_x0007__x0007_
_x0007__x0007_ _x0007_ _x0007_ _x0007_
_x0007_

_x0007_
_x0007__x0007__x0007_
_x0007_

_x0007__x0007_
_x0007_

_x0007_

_x0010_

_x0010_

_x0010_

_x0010_

_x0019_ (

_x0016_ (_x0019_

嗯 禾 绿 (2 侍

九 涮 槖

X X

f l i p

r o 0 T a n o n
